# Supplementary material for: Analysis of Greek prehistoric combat in full body armour based on physiological principles: A series of studies using thematic analysis, human experiments, and numerical simulations
Source: PLoS One. 2024 May 22;19(5):e0301494. doi: 10.1371/journal.pone.0301494 (PMC11111059; doi:10.1371/journal.pone.0301494)
Supplement: S1 File — (ZIP) [file pone.0301494.s001.zip › Details on the rationale, methods, and results of the study.docx]

# Analysis of Greek prehistoric combat in full body armour based on physiological principles: a series of studies using thematic analysis, human experiments, and numerical simulations

Andreas D. Flouris,^1^* Stavros B. Petmezas,^1^ Panagiotis I. Asimoglou,^1^ João P. Vale,^2,3^ Tiago S. Mayor,^2,3^ Giannis Giakas,^4^ Athanasios Z. Jamurtas,^4^ Yiannis Koutedakis^4,5^ Ken Wardle,^6^ Diana Wardle^6^

^1^FAME Laboratory, Department of Exercise Science, University of Thessaly, Greece;

^2^Transport Phenomena Research Centre (CEFT), Engineering Faculty, Porto University, Rua Dr. Roberto Frias, 4200-465 Porto, Portugal;

^3^Associate Laboratory in Chemical Engineering (ALICE), Engineering Faculty, Porto University, Rua Dr. Roberto Frias, 4200-465 Porto, Portugal;

^4^School of Exercise Science, University of Thessaly, Greece;

^5^Research Centre for Sport, Exercise and Performance, Faculty of Education, Health and Wellbeing, University of Wolverhampton, UK;

^6^Department of Classics, Ancient History and Archaeology, University of Birmingham, UK.

## Corresponding author

Andreas D. Flouris

FAME Laboratory

Department of Exercise Science, University of Thessaly

Karies, Trikala, 42100, Greece

Tel: +30 2431 047 072; e-mail: andreasflouris@gmail.com / aflouris@uth.gr

## Section 1. Background concept

The bronze panoply discovered in 1960,[^1^](#_ENREF_1)^,^[^2^](#_ENREF_2) in a small Mycenaean chamber tomb at Dendra in the Greek Argolid is not just the only *complete* suit of armour from Europe but also among the earliest yet known, dating c 1450 BC.[^3-5^](#_ENREF_3) Until the discovery of the panoply, it was regularly assumed that the references to bronze armour in the Iliad were all later interpolations. We now know that the Dendra armour is neither unique nor a prototype. Its sophistication and the refinements in its functionality show it to be the product of a period of development. Elements of similar armour dating from c 1500 – 1200 BC have been recognised from earlier excavations and found more recently.

Before the discovery, a shoulder piece had been found in another chamber tomb at Dendra,[^6^](#_ENREF_6) but was assumed to be a helmet. Soon after, a group of plates was found in the ‘arsenal’ at Thebes in Central Greece,[^2^](#_ENREF_2) ^and also in Illustrated London News, Dec. 5, 1964^ and later another in the Municipal Conference Centre site in the same town.[^7^](#_ENREF_7) Fragments have been identified in tombs at Phaistos in Crete,[^8^](#_ENREF_8) Nichoria[^9^](#_ENREF_9) and in the ‘Tomb of the Griffin Warrior’ at Pylos[^10^](#_ENREF_10) in the south west Peloponnese, Kallithea in Achaea,[^11^](#_ENREF_11) and at Mycenae in Chamber Tomb 15.[^12^](#_ENREF_12) Other pieces were found in the ‘Poros Wall Hoard’ close to the Clytemnestra tholos at Mycenae (observed in archive photographs by Diana Wardle, currently under study by D. Mason and S. Aulsebrook).[^13^](#_ENREF_13) Armour of the Dendra type also inspired a stone vase in the shape of a panoply with shoulder pieces found in a tomb at Knossos.[^14^](#_ENREF_14) Thus, there can be no doubt that armour of the Dendra type was well-known throughout the Aegean area (Figures s1-2) with the earliest known example (Phaistos) dating to c 1500 BC.

As we were putting together the final queries and edits to this paper, a valuable new series of studies became available in *Brill’s Companion to Bronze Age Warfare in the Aegean* edited by L. Kvapil and K. Shelton (Brill, 2023). These provide important information about the context and nature of Mycenaean warriors and warfare but do not address the question of the utility of the armour in any great detail. We have referenced it where appropriate.

**Figure s1.** Map of the Late Bronze Age Aegean region showing sites mentioned in text, find spots of plate armour and Linear B tablets listing armour.


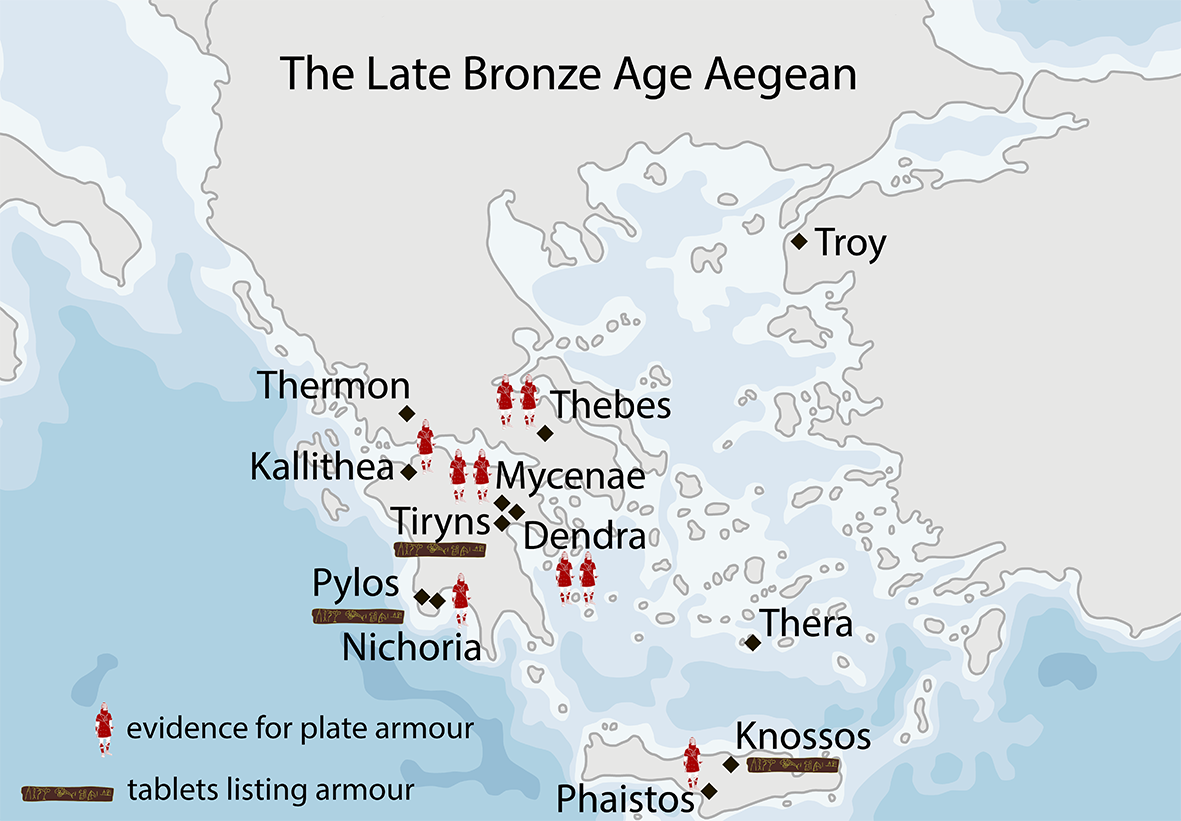


**Figure s2.** Transcript of the ‘Chariot tablet’ from Knossos No. 226, Sc32, showing chariot, suit of armour and horse, exemplifying their direct connection.


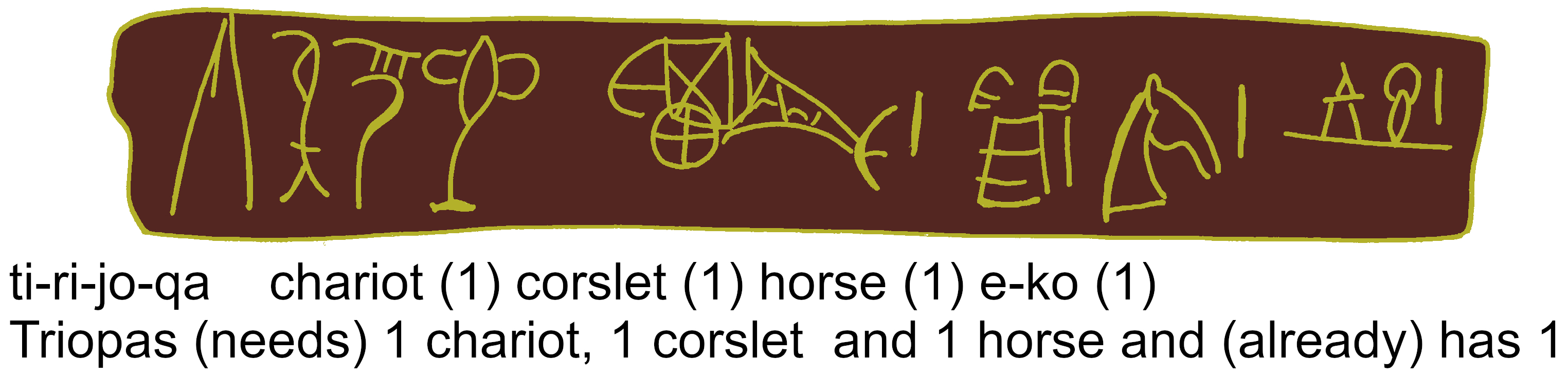


### Section 1.1. Thematic evidence for armour in the early Greek Linear B script

Mycenaean administrators were compulsive record-keepers, writing on soft unbaked clay tablets for initial lists of many different types of goods and supplies, including sheep and goats, grain or cloth, everything from people to figs but also weapons and armour. In ‘Documents in Mycenaean Greek’ published the year before the discovery of the Dendra armour, Ventris and Chadwick had reported at least 140 suits of armour at Knossos in Crete and another 20 at Pylos in the Southwest Peloponnese, complete or in parts, listed on the Linear B tablets.[^15^](#_ENREF_15) Chadwick, however, continued to assume that these tablets were ‘school room’ exercises rather than records of real armour since the evidence for bronze armour was still limited.[^16^](#_ENREF_16)

Since some tablets list armour, chariot and horse or horses attributed to a named individual, we can be sure that their nature is military. Other parts of the ‘uniform’ are also listed, including leather gambeson and linen tunics. We do not know the exact context of the lists: are they an inventory of items present or a list of those required? Do they record the state of readiness for war or battle or the deficiencies at the end of the campaign? The number of suits of armour recorded at Knossos suggests a large group of those known to Homer as χαλκοχίτωνες (men with brazen tunics).

Recent analysis[^17^](#_ENREF_17)^,^[^18^](#_ENREF_18) shows that the archives of Linear B tablets at the Palace of Knossos, in the ‘Room of the Chariot Tablets’ alone, indicate the existence of at least 250 chariots, each with provision of armour to equip two men, as well as the horses to draw them. This demonstrates that the palace authorities must have maintained a mobile and well-armoured fighting force of 500 or more men. Similar records from the ‘Palace of Nestor’ at Pylos refer more to the maintenance and refurbishment of armour than the numbers present. A single instance of the armour ideogram has been recognised on a tablet discovered at Tiryns[^19^](#_ENREF_19) whilst several cheek-pieces are recorded at Thebes.[^18^](#_ENREF_18) Survival of these temporary records is accidental – only a consequence of the fires which destroyed the palaces – and they may well be an underestimate of the quantity of armour which existed.

Assuming that each suit of armour weighed around 18 kg (see analysis below, Section 4.5), the minimum estimates of numbers demonstrate enormous resources available to the palatial centres. At Knossos this set of tablets on its own implies that nine tonnes of bronze would have been needed, of which ~10 %, 900 kg, was tin. In this quantity, tin had to be sourced from very distant places – Afghanistan and probably even Britain[^20^](#_ENREF_20) – and it demonstrates the scale of ‘international’ trade in the 2^nd^ Millennium BC. For comparison, the ship that sank off the South coast of Turkey at Uluburun near Kaş, c 1320 BC was loaded with copper ingots of around 30 kg each, totalling 10 tonnes and one tonne of tin ingots – the right proportions for bronze.[^21^](#_ENREF_21)^,^[^22^](#_ENREF_22)

Up until the discovery of the Dendra panoply, it was assumed that the ideograms named to-ra-ka – θώρακα – represented armour made from layers of linen or canvas to provide protection.[^15^](#_ENREF_15)^,^[^23^](#_ENREF_23)^,^[^24^](#_ENREF_24) This concept was based on the two references in the epics to the use of the λινοθώρηξ (linen corslet), regardless of the existence at Knossos of several examples where the ideograms were ‘overwritten’ with the representation of a bronze ‘oxhide’ ingot – surely the record of an instance where the raw material was available to make a required suit of armour. The Knossos ideogram shows the shoulder pieces and the lower bands very clearly, whilst those from Pylos show the neck-guard and a conical helmet. These differences are probably no more than the local practices of the scribes in each palatial centre.

It is clear from all this evidence that bronze armour was in regular use in Mycenaean Greece, even if the majority was recycled to leave only a few surviving fragments in addition to the complete Dendra panoply. This frequency matches that in Homer’s Iliad, where the elite warriors are almost all armour-clad (see Section 2.5).

### Section 1.2. Combat in Mycenaean Greece and after

Warriors are familiar themes in Mycenaean art, whether in single combat,[^25^](#_ENREF_25)^(p. 41, fig 29)^ in battle[^26^](#_ENREF_26) or conducting a siege.[^25^](#_ENREF_25)^(p. 224, fig 178; p. 242, fig 203)^ Their equipment – swords and spears, bows and arrows, shields, greaves and helmets - is all clearly shown, though sometimes without great detail. Curiously only the armour is absent from these illustrations – perhaps, as in Classical Art where warriors are often depicted naked, Mycenaean heroism was indicated by the lack of protection. Chariots are also well represented, but usually in hunting scenes[^25^](#_ENREF_25)^(p. 41, fig 30)^ or ‘processions’[^27^](#_ENREF_27) rather than in battle. Finds of examples of most of this equipment – or at least the durable elements – are regular throughout the Mycenaean period. All of them are attested in the Linear B record. Apart from a group of warriors drawn up on the shore in the ‘ship fresco’ from Akrotiri[^28^](#_ENREF_28)^(p. 58, fig 26 and 28)^ there is no indication of fighting ‘in formation’. As time passes the actual practice of combat must have changed: long rapiers give way to shorter, stouter, slashing swords; smaller spearheads suggest more are thrown as javelins.[^5^](#_ENREF_5) Smaller shields, as shown on the ‘warrior vase’ from Mycenae[^25^](#_ENREF_25)^(p. 149, fig. 117)^ suggest a shift from protection to mobility. The sophistication and prosperity of Mycenaean civilization is compatible with military campaigns of the kind described in part in the Iliad, even if the ten-year scale of that conflict is poetic exaggeration. (Note: a vast collection of images of Mycenaean military equipment and combat entitled “The Greek Age of Bronze” has been assembled by Andrea Salimbeti at http://www.salimbeti.com/micenei).

There is no shortage of examples of weapons from Iron Age graves from c 1000 BC onwards. Warriors were still important members of society, but much of their equipment was now of iron not bronze.[^29^](#_ENREF_29)^,^[^30^](#_ENREF_30) Whether this change reflects changes in economic circumstances and the difficulty of accessing the metals required for bronze – copper and tin – or whether the eventual superior quality of iron weapons rendered them preferable, even if less effective when first created, remains debated.[^31^](#_ENREF_31) One of the Bronze Age sword types (Naue II),[^32^](#_ENREF_32)^,^[^33^](#_ENREF_33) originally in bronze, continues to be made in the same form but now in iron. With the loss of most figurative art in the Early Iron Age, we lose the images which would provide greater enlightenment about warriors and warfare. Occasional finds such as a bronze panoply from Argos c 750 BC[^34^](#_ENREF_34) provide hints of continuing practices.

In contrast, the Homeric Epics are full of details of equipment and the actions of individual heroic warriors or small groups. There is no question that bronze armour was part of the heroic tradition preserved in the Iliad. The Achaeans, as Homer calls the Greeks (Mycenaeans refers only to inhabitants of Mycenae itself), are described as wearing ‘brazen tunics’ – χαλκοχίτωνες – in over 20 passages. On six more occasions the epithet is used for other groups – Argives, Boeotians, Cretans, Epeians – and even once for the Trojans. The words θώρηκας for the cuirass itself and τεύχεα for the armour as a whole set of equipment are regularly used (20 and 114 examples respectively) and their metallic character is made specific when described as bronze, as gleaming or as clanging when a warrior falls to the ground. Two warriors have golden armour and several have it elaborately decorated. Stripping armour from a fallen opponent is regular too – a clear indication of its value. In only two cases is the armour described as linen (λινοθώρηξ – Ajax the Less, Il. 2.529 and the Trojan Amphios, Il. 2.829) and obviously exotic, as are the occasional references in Classical literature, by Herodotus, for example, of the gifts by the Pharaoh Amasis (Ahmose II) to Athena of Lindos and to the Spartans (Herodotus Histories 2.182; 3.47).

The regular use of the term χαλκοχίτωνες (brazen tunics) for groups gives the impression that armour was relatively common, whilst the detailed descriptions are always for individual heroes – the elite warriors who each led their own bands of followers. As mentioned above, until the discovery of the Dendra panoply and consequently the demonstration that the Mycenaeans had full suits of metal armour (not just greaves and occasional helmets) scholars had regularly suggested that all references to bronze in the epics were later interpolations designed to show the heroic use of the ‘hoplite’ armour which, in fact, only came into use after 700 BC.[^35^](#_ENREF_35)^,^[^36^](#_ENREF_36) This view also coloured the interpretation of the ‘armour’ ideograms in the linear B texts as representations of linen rather than bronze armour.

Nowhere is there any sign of the formations developed from the 6^th^ century on of the hoplite warriors typical of the Archaic and Classical Greek city states such as Argos, Athens and Sparta. It is unlikely that a combined Greek expedition such as the Trojan War could have been mounted in the centuries between 1200 and 700 BC, given the apparently fragmented nature of Greek civilization. Tradition, however, maintained the memory of such a major event and of many of the details which allow us to place it in a Bronze Age context. Intriguingly there is one more recent event preserved by ancient Greek historians – the so-called ‘Lelantine War’ between Eretria and Chalkis on Euboea, in which many Greek communities supported one side or the other (Thucydides, History of the Peloponnesian War, 1.15). The details preserved of this event, dated around 700 BC are, however, meagre to say the least[^37^](#_ENREF_37) but there is the first reference to the use of cavalry in battle, conspicuously absent from the Iliad (Plutarch, Amatorius 17. (Moralia 760ε-761β).

Despite the evident changes in social order from the Mycenaean to the Archaic period, there is nothing to suggest that the character of warfare had changed substantially. Bronze armour may well have been more common in the Late Bronze Age than in Homer’s own time, but the heroes in the Iliad are certainly equipped with it. Their actions, as described there and analysed in Section 2, provide a sound basis for the development of a Late Bronze Age combat simulation protocol created for the experiments described in Section 3.

### Section 1.3. Research question

The discovery of the Dendra panoply in 1960 immediately raised the issue of its function. Could it have been worn in battle[^38^](#_ENREF_38) – as repeatedly demonstrated by the exploits of the warriors in the Iliad? Was its use restricted to those who rode to battle in chariots?[^39^](#_ENREF_39) – again, a regular practice in the epic and indicated in the Linear B archives by the association of armour and chariots, or was it too cumbersome to be worn except on ceremonial occasions[^40-42^](#_ENREF_40) as stated for example in the caption to the pieces of armour from Thebes on display in the Archaeological Museum of Thebes? Only experiment – bound by strict criteria and content – with a replica could answer these questions since the metal of the panoply itself is too fragile for use.

Understanding the location, the environment, and the physical effort exerted in the associated activities are essential to an analysis of the physiology involved in Late Bronze Age warfare which, in turn, will answer the research question of this study: was the armour found at Dendra regular equipment suitable for extended use in battle or was it purely ceremonial?

## Section 2. Analysis of Late Bronze Age warfare and battle tactics (Study 1)

The Iliad describes a small part of the war between the Achaeans (a coalition of Greek city-states from mainland Greece, Crete, and the Aegean islands) and the Trojans (inhabitants of the Troad aided by other city-states in Thrace and Asia Minor).[^43-46^](#_ENREF_43) Homer describes military activities in detail from dawn to dusk and provides vivid descriptions of single combat between heroes as well as of the action on the battlefield as a whole. Homer’s epic story of heroism and slaughter, of anger and comradeship, had been passed down from generation to generation as oral poetry preserving the legends of Late Bronze Age heroes and their names which have, ever since, resounded through the history of Europe.

Homer’s oral poetry of the 8^th^ century BC, which was first written down hundreds of years later than the events it describes, is undoubtedly based on distant memories of an age whose citadels and monuments were familiar parts of his audiences’ experience. Its details of military action had also to be familiar to them. Although the feats of ancient warriors were exaggerated and embellished, their stories were told to live audiences – both aristocrats and their retainers – of those who would understand the practices of war, practices of their own day and their own experience.[^47-49^](#_ENREF_47) Whilst the actions of the heroes are sometimes impossibly heroic, the general context of the conflict and the practical needs and capabilities of the less exalted warriors seem entirely realistic.

To suggest, as Schliemann did in the 1870s, that Homer’s epics were accurate accounts of events which took place some five hundred years earlier, is to invite both censure and disbelief. To follow Thucydides’ (I.2-12) approach that the traditions about the remote past of Greeks contain truth and suggest that they provide a reasonable basis for evaluating the nature of early warfare in Greece and, by extension, the nature of Late Bronze Age warfare, is, we believe, a rational starting point. We have, therefore, based our analysis on the circumstances and actions of the fighting outside the walls of Troy, as described in Homer’s Iliad, and calibrated it on the basis of the environmental conditions there in the Late Bronze Age, as far as they can be reconstructed for the period during later phases of the Late Bronze Age (between 1300 and 1200 BC). This is a date range generally accepted as the likeliest for the Trojan War to have taken place (see Section 2.1), on the assumption that the events related in it, whether one or many separate ones, were a historic reality. Accordingly, we are confident that this analysis of the Iliad has provided a logical sequence of parameters for understanding Late Bronze Age armament and warfare, i.e. for the weapons available and the actions, both offensive and defensive, of individual warriors.[^1^](#_ENREF_1)^,^[^29^](#_ENREF_29)^,^[^38^](#_ENREF_38)^,^[^47^](#_ENREF_47)^,^[^50^](#_ENREF_50)^,^[^51^](#_ENREF_51) In this context, it was necessary to derive information along the following seven topics:

1. The characteristics of the physical environment in which battles took place.
2. The typical start and end time of daily army operations.
3. The typical activities performed by warriors during one day.
4. The typical food and water intake during a day of battle.
5. The physical characteristics and the level of combat experience of most warriors.
6. The main types of combat.
7. The typical techniques, movements, and weapons used by combatants.

Given that the Trojan War, as described in the Iliad, was used as the paradigm for Late Bronze Age warfare, we conducted a review of the scholarly literature as well as two thematic analyses of Homer’s Iliad to explore all the aforementioned seven topics. For the literature review, we searched the Google Scholar and PubMed databases from their date of inception to January 2020 using search terms related to each of the seven topics. We included all types of peer-reviewed papers, conference proceedings, and books. Manuscript titles and abstracts were subsequently screened for relevance. Thereafter, full texts (retrieved via the University of Thessaly library) were screened independently by three reviewers (ADF, SP, and PA).

The 1^st^ thematic analysis of the Iliad addressed topics 1 through 5 (Study 1), while the 2^nd^ thematic analysis addressed topics 6 and 7 (Study 2). Each analysis included a careful study of the Iliad performed independently by two reviewers who were either study investigators or had an extensive background in classical studies. To eliminate inter-reviewer bias due to interpretation of ancient Greek, the thematic analyses were performed using the Iliad translation into Modern Greek by Prof. Dimitrios N. Maronitis,[^52^](#_ENREF_52) a well-accepted translation that received the 2011 State Award for Interlanguage Translation. Notes addressing each topic were transcribed verbatim by a third investigator and were proofread by the original reviewer. Thematic analysis of transcripts was conducted independently by three investigators (ADF, SP, and PA). Thereafter, each investigator reviewed the others’ results, and then they established the final themes by consensus. The results from the two thematic analyses of the Iliad combined with the results from the review of the literature are summarised in the following subsections 2.1-2.7, addressing all the above-mentioned seven topics characterizing Late Bronze Age warfare and battle tactics. The data collected from the 2^nd^ thematic analysis has been placed in an online data repository and is freely available (<https://doi.org/10.6084/m9.figshare.12090831.v1>).[^53^](#_ENREF_53)

### Section 2.1. The characteristics of the physical environment in which battles took place (topic 1)

The location and the date that an event took place are the two most essential elements from which to derive information about the physical environment in which it transpired. Late Bronze Age Troy was likely the capital of a city-state that controlled the surrounding landscape, the Troad.[^43^](#_ENREF_43) It was situated south of the southwest mouth of the Dardanelles strait and northwest of Mount Ida (Latitude: 39.957616°; Longitude: 26.238851°), in the far northwest of the region known in late Classical antiquity as *Asia Minor*, now known as Anatolia in modern Turkey.[^38^](#_ENREF_38)^,^[^54^](#_ENREF_54) The paleogeography of Homeric Troy – dominated by the rivers Scamander and Simois – has intrigued scholars for more than two millennia. In antiquity, Strabo devoted several passages to it in his Geography.[^55^](#_ENREF_55) Much more recently, Kraft et al.[^54^](#_ENREF_54) conducted an in-depth analysis of the areas’ sedimentology and geomorphology, mapping the paleoenvironments and testing phrases in the Iliad against the observed data to specify areas that could have served as harbours for ancient Troy, for the Greek encampment, and for other landforms described in the Iliad. Their analyses demonstrated that the area surrounding Troy during the time of Homer’s Iliad was characterized by “*an extremely irregular delta coastline with constantly varying…swamps, brackish to saline lagoons, and bypassed flanking ponds or lakes progressing from saline to brackish to freshwater. Water depths…were frequently 1 m and could vary to 3-4 m. As the delta coast approached the Dardanelles, littoral currents and increased wave action sorted sands into nearshore shoals and possibly thin beaches*”.[^54^](#_ENREF_54) By combining the paleogeographic analyses and the maps of Kraft et al.,[^54^](#_ENREF_54) the locations of morphology and historic features of Iliad from Luce,[^56^](#_ENREF_56) as well as the geographic indicators and distances provided by Strabo,[^55^](#_ENREF_55) we used Azgaar's Fantasy Map Generator, a free web application, to create our interpretation of the geomorphologies in the area surrounding Troy at the time of the events which are recorded in the Iliad (Figure 2 in main text). A video showing the map in 3D can be freely viewed online (<https://youtu.be/jvQ9YTt6yzA>). The video has been also placed in an online data repository and is freely available (<https://doi.org/10.6084/m9.figshare.12961463.v1>).[^57^](#_ENREF_57)

Naturally, the battle ground morphology was different for every battle during the Late Bronze Age and was an important aspect of military tactics. The information provided above suggests that the Trojan War took place in a ~4 km^2^ area characterized by the Scamander river plain, with meandering river levees and backswamps, and a coastline with irregular shaped bays and pervasive coastal marshes. The morphology of the battle ground was characterised by flat ground with low hills of not more than 30 m altitude.

Dating the Trojan War was outside the scope of the present study. However, determining the time period and the month in which the events described in the Iliad took place was essential to deriving information about the physical environment (season, temperature, day-night cycle, etc.). Our thematic analysis of the Iliad suggested that the events described took place in the summertime, as Homer provides many references to increased heat, especially during mid-day. Yet, providing a date for the Trojan War is a complex challenge which has been a subject of frequent discussion since antiquity.[^58^](#_ENREF_58) Eratosthenes provided an estimate of ca. 1184 BC, Douris of Samos proposed that the War took place in 1334 BC,[^59^](#_ENREF_59)^,^[^60^](#_ENREF_60) while an estimate of ca. 1250 BC was provided by both Herodotus[^61^](#_ENREF_61) and Blegen.[^62^](#_ENREF_62) In total, ancient authors have placed the Trojan War within a long period between 1334 and 1129 BC.[^58-62^](#_ENREF_58) A recent series of studies analysing in detail some of Iliad’s descriptions of physical phenomena covered by “divine interventions” (considering that physics at that age was entangled within *mythos* and *theology*) suggested that the events described in the Iliad may have taken place in June, 1218 BC.[^63-66^](#_ENREF_63) However, this conclusion is predicated on the assumption that, despite the distance in time, the described phenomena are precise and accurate memories of events that occurred, rather than being introduced for dramatic effect. Overall, it is not clear whether later narratives are reliable evidence for the dating of the Trojan War.[^67^](#_ENREF_67) Taking all this information and approaches into account, we hypothesised that the events described in the Iliad took place in June during the later phases of the Late Bronze Age (between 1300 and 1200 BC).

Based on the estimated date of June during the later phases of the Late Bronze Age, paleoclimate data using radiocarbon dating of stable oxygen and carbon isotopes in samples from the surrounding region (temperature: Aegean and Hellespont; humidity: Greece, Aegean Sea, Turkey)[^68^](#_ENREF_68) as well as palaeoceanographic research using marine sediment core from the central Aegean Sea[^69-71^](#_ENREF_69) suggest that the average annual temperature was 18-20 °C and the average annual relative humidity was 70-80 % in this period. To our knowledge, no data exist on monthly variability of temperature or humidity in the Aegean region during the Late Bronze Age. Therefore, we assumed a monthly distribution similar to the present (1980 to 2017). Based on publicly available weather station recordings from the United States National Oceanic and Atmospheric Administration[^72^](#_ENREF_72) for the Canakkale weather station (the closest weather station to Troy), the current (1980 to 2017) average annual temperature is 15.1 °C and the average temperature in June is 22.5 °C. Thus, the aforementioned average annual temperature of 17.8 °C in the later phases of the Late Bronze Age suggests that the average temperature in June was probably 26.4 °C. Given a 10 % day-to-day variability, the temperature in June during this period would have ranged from 23.8 °C to 29.1 °C. Similarly, the current (1980 to 2017) average annual humidity is 75.1 % and the average humidity in June is 68.6 %. Thus, the aforementioned average annual humidity of 84.8 % in the later phases of the Late Bronze Age suggests that the average humidity in June was probably 77.4 %. Given a day-to-day variability of 10 percentage points, humidity in June during this period would have ranged from 69.6 % to 85.1 %.

### Section 2.2. The typical start and end time of army operations (topic 2)

The Sunrise/Sunset and Solar Position Calculators from the United States National Oceanic and Atmospheric Administration[^73^](#_ENREF_73) suggest that the average sunrise and sunset during June in the later phases of the Late Bronze Age were 04:37 and 19:37, respectively, ±10 minutes. Our thematic analysis of the Iliad suggested that armies moved out of their camps after the sunrise following a large breakfast, equipment set-up and check, and council meetings of the group leaders. The same analysis showed that the battle ended near sunset and was followed by burying the dead, preparing and having dinner, and council meetings of the group leaders. Based on references provided by Homer as well as consultation with experts in classical and modern warfare, we estimated that armies moved out of their encampment approximately 2.5 hours after sunrise, and that fighting ended about 1.5 hours before sunset. Based on these sun-based time points and the aforementioned sunrise and sunset times during June in the later phases of the Late Bronze Age, we concluded that the army operations started at about 07:00 and ended at about 18:00, making each day an 11-hour operation. This 11-hour time-length was adopted for the purposes of the current investigation. These estimates are in line with previous analyses of Homeric[^48^](#_ENREF_48) and classical Greek[^74^](#_ENREF_74)^,^[^75^](#_ENREF_75) warfare.

### Section 2.3. The typical activities performed by warriors during one day (topic 3)

A physiological analysis of warfare requires information regarding the duration and timing at which different tasks are carried out. According to Strabo, the Greek ships were beached and the army encamped was close to Sigeum, 20 stades (4 km) from Troy (Figure 2 in main text).[^55^](#_ENREF_55) This is supported by the environmental lithosome distributions and the radiocarbon dates performed by Kraft et al.[^54^](#_ENREF_54) Our 1^st^ thematic analysis of the Iliad indicated that armies typically returned to their camp at the end of the day in the vast majority of cases. This would imply that – in addition to the actual fighting – the Greek army marched ~8 km each day. However, the Trojans camped in the valley in one of the nights described in the Iliad to continue pushing the Greeks towards the sea. Therefore, we analysed the battle tactics used during the four days of battle (out of the 10 days in total) described in the Iliad to derive a reasonable estimate of the typical distance covered by armies during one day.

The marching speed of the Greek army in the Classical period has been estimated as 3-4 km/h for typical marching and 6-8 km/h for maximal speed.[^75^](#_ENREF_75)^,^[^76^](#_ENREF_76) An exhausted army returning to camp at the end of the day while carrying the dead and the wounded was hypothesized to march at 3 km/h. Therefore, it would have taken the Greek army approximately three hours to reach Troy, assuming 90 min to cross the river Scamander and regroup, as well as 30 min to assume battle formation (Figure s3, panel A). The time needed to return to camp was estimated at three hours and 20 min, assuming 30 min for disengagement and 90 min to cross the river Scamander and regroup (Figure s3, panel B). In the 2^nd^ day of the Iliad when the duel between Menelaus and Paris takes place (Iliad III), the battle ground is outside the walls of Troy (Figure s3, panel C). In the 4^th^ day of the Iliad where Hector leads the Trojans, the battle starts in the morning outside the walls of Troy and the Trojans advance throughout the day (Iliad VIII) forcing the Greeks to fall back to their camp by the evening (Figure s3, panel D). In the 5^th^ day of the Iliad when the death of Patroclus takes place (Iliad XVI), the battle ground is outside the walls of the Greek camp and at the coast (Figure s3, panel E). In the 7^th^ day of the Iliad where Achilles ends his refusal to fight and takes the field pushing the Trojans back from the beaches and into Troy (Iliad XIX), the battle starts in the morning outside the walls of the Greek camp and their army advances throughout the day forcing the Trojans to fall back to their city by the evening (Figure s3, panel F).

The above analysis and the times shown in Figure s3 provide reasonable estimates for the typical time spent for deployment of the army and for returning to camp. The time that each warrior spent in combat has been estimated from the 2^nd^ thematic analysis of the Iliad described in Sections 2.6-2.7 (see below). Our 1^st^ thematic analysis of the Iliad indicated that the majority of battles lasted the entire day. For instance, Odysseus warns Achilles at Il. 19.157-161 that “*…the combat will last no little while once the masses of men meet in battle and the god breathes strength into both sides*”. This prolonged duration of fighting was typical in times of ancient Greece.[^74^](#_ENREF_74)

| **Figure s3.** Characteristics of the march between the Greek camp and Troy (panels A and B) and analysis of the battle tactics used in the four days of battle (out of the 10 days in total) described in the Iliad (panels C-F). The maps were created using Azgaar's Fantasy Map Generator, a free web application, under a CC BY license, with permission from Max Haniyeu, original copyright 2017-2021. | |
| --- | --- |
| 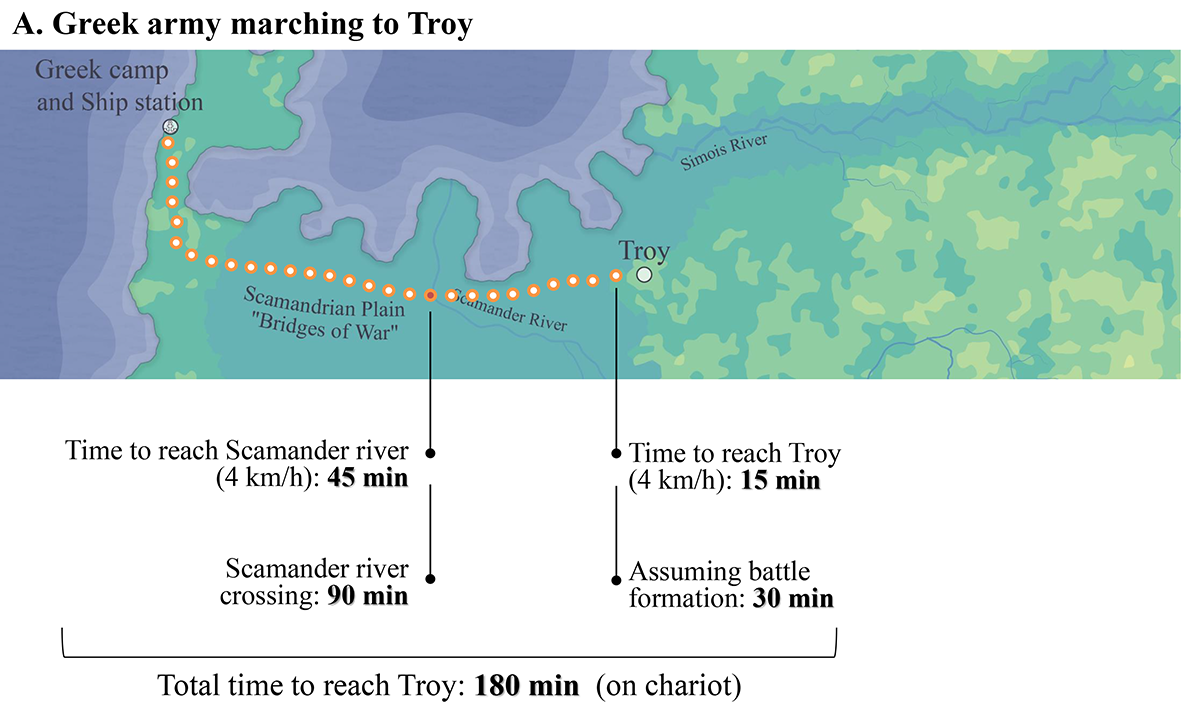 | 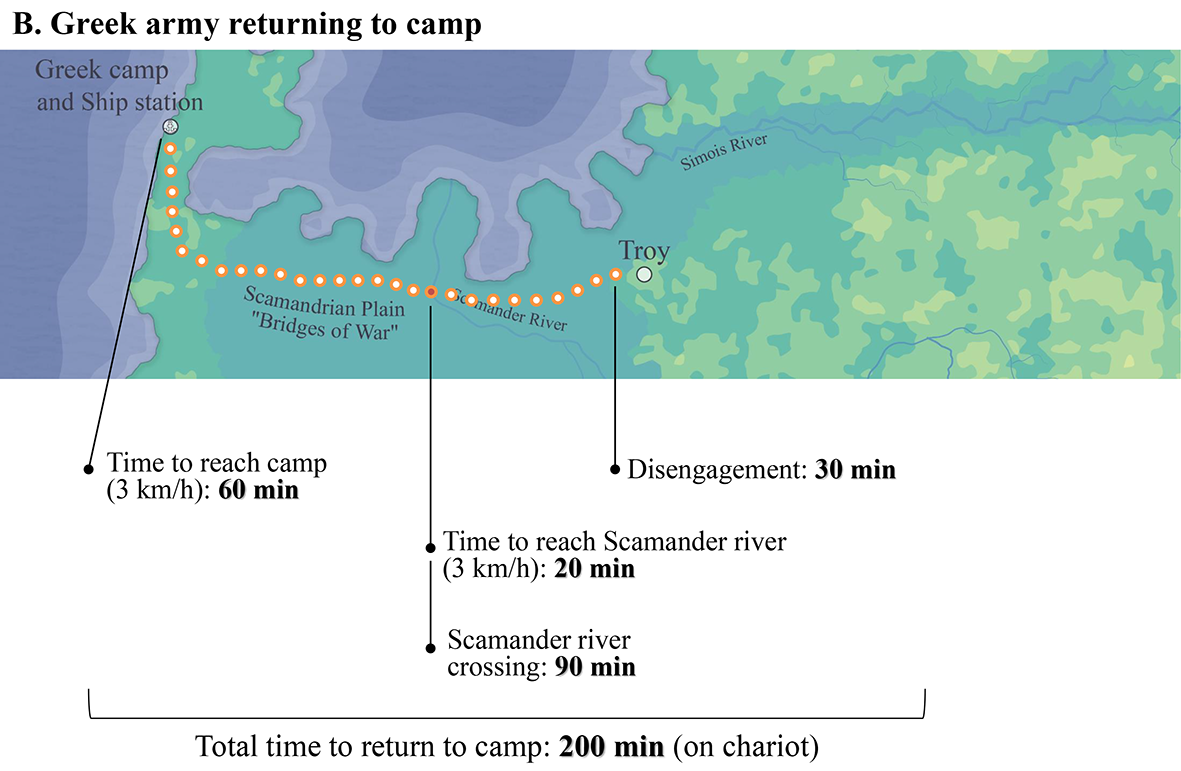 |
| 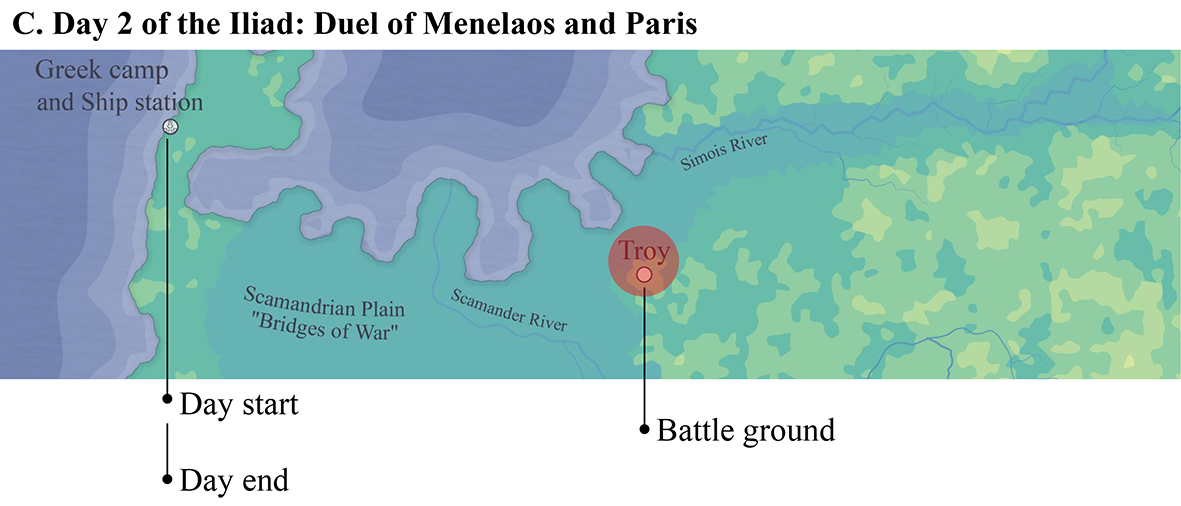 | 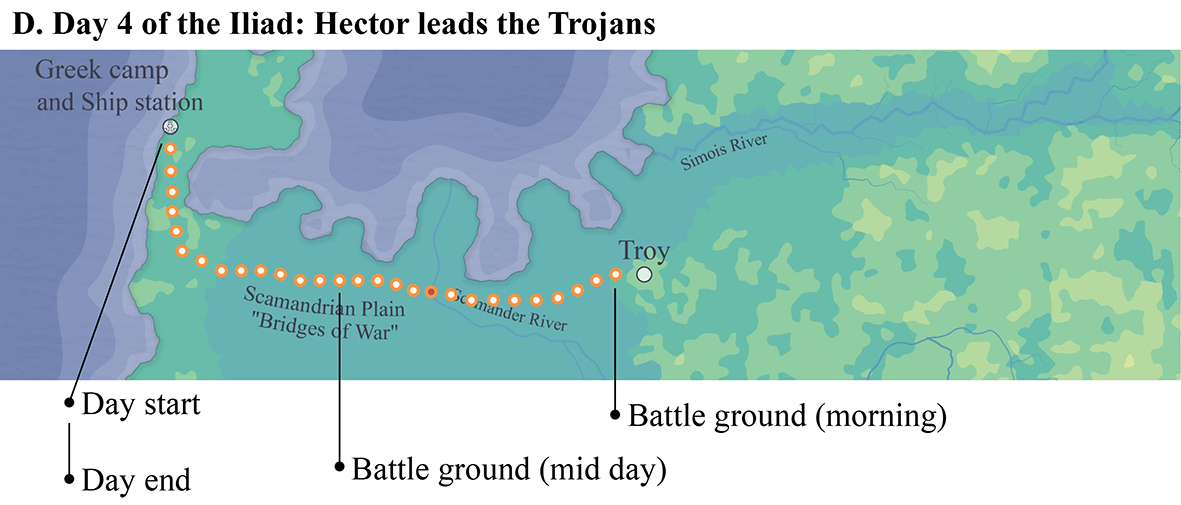 |
| 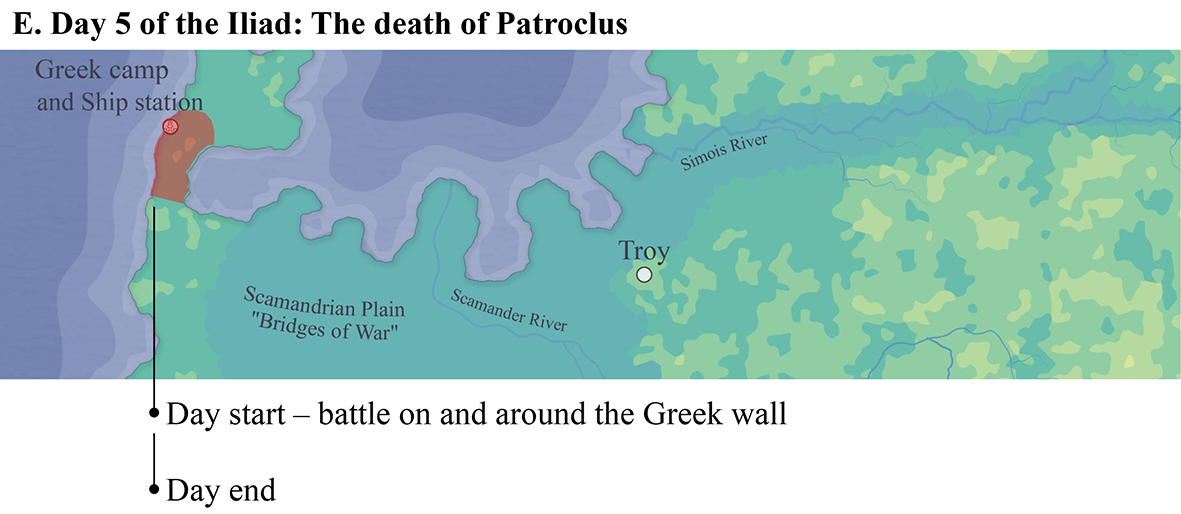 | 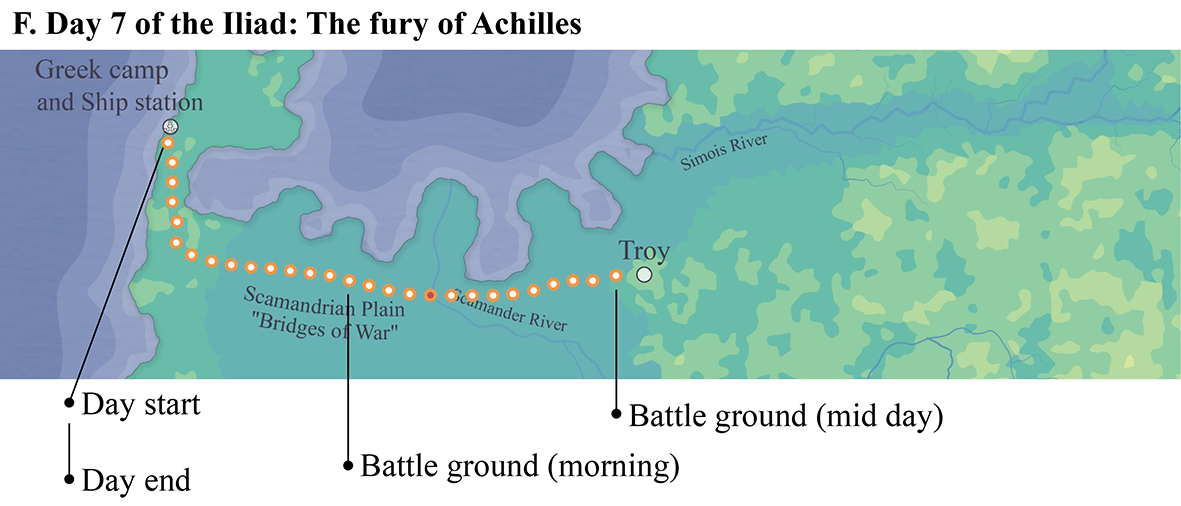 |

The army was organised in groups / bands, where elite warriors were surrounded by their “*hetairoi*” (followers) who accompanied them when they moved to another part of the battlefield or leave and re-enter battle. Thus, elite warriors with their bands were able to freely move across the battlefield, pick their targets and engage them using long- and / or short-range weapons. An elite warrior with his group – which can be assumed to include anywhere from just a few up to several dozen men – could move through the ranks of army and join the “*promachoi*” (foremost fighters) for as long as he was willing and able to effectively engage the enemy. He could also choose to refrain from combat and be part of the “*plithos*” (multitude) of men, staying out of immediate danger. Thus, in some cases warriors would take a break – by either moving to the back of the army or by returning to camp – to rest, eat, drink, pick up weapons, mend their armour, or consult with comrades.

Based on the information from the 1^st^ thematic analysis of the Iliad as well as physiological principles, we postulated that the break time of elite warriors was 50 % more than that spent on combat. Also, we assumed that any time during the 11-hour long daily operation (see Section 2.2) not spent on deployment, returning to camp, combat, or break was, instead, spent on manoeuvring. This was an important battle tactic since elite warriors were separated by considerable distance from one another. The aim of manoeuvring, therefore, was to relocate from the back of the army formation towards the front (e.g., when a warrior picked an adversary and approached him to shoot an arrow, cast a spear or stone), or from the front lines to the rear (e.g., when a warrior quickly retreated to safety after a one-on-one combat), or sideways (e.g., to find a weak point in the enemy lines or support comrades in danger). Given that the present physiological analysis is focused on elite warriors such as the one wearing the Dendra armour (see Section 4) who were, undoubtedly, high-ranking officers,[^1^](#_ENREF_1)^,^[^67^](#_ENREF_67)^,^[^77^](#_ENREF_77) we hypothesised that 70 % of the manoeuvring time was on a chariot,[^38^](#_ENREF_38) and 30 % was on foot. Based on these assumptions and on the above time estimates for the movement of the armies (Figure s3, panels A and B), the analysis of the battle tactics used in the four days of battle described in the Iliad (Figure s3, panels C through F), as well as information derived from the 2^nd^ thematic analysis addressing topics 6 and 7 (see Sections 2.6-2.7), our estimated typical battle activities performed by warriors during one day are shown in Table s1.

| **Table s1.** Estimated time (hours:min) spent on typical activities performed by elite warriors during the four days of battle in the Trojan War, as described in the Iliad. | | | | | | |
| --- | --- | --- | --- | --- | --- | --- |
| **Day in Iliad** | **Deployment^1^** | **Return to camp^1^** | **Combat^2^** | **Break^3^** | **Manoeuvring (on chariot)^4^** | **Manoeuvring (on foot)^5^** |
| 2 | 3:00 | 3:20 | 0:54 | 1:21 | 1:41 | 0:44 |
| 4 | 3:00 | 0:00 | 0:10 | 0:15 | 5:18 | 2:17 |
| 5 | 0:00 | 0:00 | 1:31 | 2:17 | 5:02 | 2:09 |
| 7 | 0:00 | 3:20 | 0:21 | 0:32 | 4:45 | 2:02 |
| **Average** | **1:30** | **1:40** | **0:44** | **1:06** | **4:11** | **1:48** |
| Note: ^1^: derived from the review of the literature and the thematic analysis; ^2^: derived from the thematic analysis (see Sections 2.6-2.7); ^3^: estimated as 150 % of combat time; ^4^ and ^5^: estimated as the time in the 11-hour operation (see Section 2.2) not spent on deployment, return to camp, combat, and break (70 % on chariot; 30 % on foot; due to the high-ranking of the individual wearing the Dendra-type armour). | | | | | | |

### Section 2.4. The typical food and water intake during a day of battle (topic 4)

According to legend, the Trojan War lasted 10 years. However, even in a campaign lasting a few months it is logical to assume that warriors needed to remain fit and healthy for active duty during prolonged periods of time. Therefore, they would need to maintain energy balance, consuming enough food to account for the energy requirements of the daily and repeated battle tasks and tactics. Odysseus’ warning to Achilles at Il. 19.161-162 that “*…No man unnourished by food will be able to fight his foe all the livelong day to the sun's setting*” confirms this hypothesis.

Based on the estimated time spent on different activities performed by warriors during one day of the Trojan War (Section 2.3) and on published standards for the energy cost of similar activities performed in athletic and occupational settings,[^78-80^](#_ENREF_78) we derived estimates for the energy requirements of warriors during a day of battle described in the Iliad (Table s2). It should be stressed here that the estimated energy requirements depend on the weight of the warrior (heavier individuals require more energy for the same activities), and that more precise estimations for the energy cost of these activities are provided below (see Section 4) based on indirect calorimetry measurements from the human study conducted as part of this physiological analysis.

| **Table s2.** Estimated time spent on different activities performed by elite warriors during a typical day in the Iliad and associated energy cost for carrying out these activities for a 75-kg individual. | | | | | |
| --- | --- | --- | --- | --- | --- |
| **Activity** | **Activity description** | **Tactics** | **Energy cost (METS)** | **Total activity time (hh:min)** | **Total energy cost (kcal)** |
| Army movement | Deployment | NA / OC | 3.0^a^ | 1:30 | 337.5 |
|  | Battle formation | AO / OC | 6.5^b^ | 0:30 | 243.8 |
|  | Disengagement | AO / OC | 6.5^b^ | 0:22 | 178.9 |
|  | Return to camp | NA / OC | 3.0^a^ | 1:40 | 375.8 |
| Warrior manoeuvring | Manoeuvres on chariot | AO / OC | 6.5^b^ | 3:19 | 1,618.5 |
|  | Manoeuvres on foot | AO / OF | 6.5^b^ | 1:48 | 877.5 |
| Combat | 1-on-1 combat | AO / OF | 10.3^c^ | 0:31 | 401.7 |
|  | Foot warrior vs chariot | AO / OF | 10.3^c^ | 0:10 | 131.3 |
|  | Chariot vs chariot | AO / OC | 10.3^c^ | 0:02 | 23.2 |
|  | Chariot vs warrior on ship | AO / OC | 10.3^c^ | 0:01 | 15.5 |
| Break | Standing or sitting | AN / OF | 3.0^a^ | 1:06 | 247.5 |
|  |  |  | **TOTAL** | **10:59** | **4,451.2** |
| Note: NA: warrior not wearing armour (cf [^81^](#_ENREF_81)); OC: warrior on chariot; AO: warrior armour on; OF: warrior on foot; ^a^: examples of relevant activities at this energy cost include walking at 4 km/h on level and firm surface, walking and carrying in munition industry, hunting / butchering small animals, playing with animals at light effort (e.g., walking the dog), walking at light effort while gathering tools, standing tasks at light/moderate effort (e.g., assemble/repair heavy parts, welding, stocking parts, packing boxes, nursing patient care), or performing tai chi / qi gong; ^b^: examples of relevant activities at this energy cost include manual or unskilled labour at moderate to vigorous effort (e.g., digging, shovelling, loading/unloading boxes, walking while carrying objects about 22.5 to 33.5 kg), or race walking; ^c^: examples of relevant activities at this energy cost include different types of martial arts (e.g., judo, jujitsu, karate, kick boxing, tae kwon do, tae-bo, Muay Thai boxing), or working with an axe. | | | | | |

Based on the hypothesis that warriors consumed enough food to account for the energy requirements of the daily battle tasks and tactics, one of the aims of our thematic analysis was to derive information on the typical food intake during a day of battle. Our findings (e.g., Il. 1.597, also 7.465-466, 8.229-232, 9.71-72, 9.89-92, 9.220, 9.215, 19.316-317) suggest that the main constituents of elite warrior diet were roasted meat (from sheep, goat, pig, or cow), bread, and wine.[^82^](#_ENREF_82) Warriors consumed about 40 % of their daily dietary intake in the morning breakfast, 10 % of their intake during breaks throughout the day, and about 50 % of their caloric intake in the evening dinner. Breakfast was comprised primarily of dry bread, goat’s cheese, olives, red wine, and water. The small snacks consumed during the day included dry bread, honey, goat’s cheese, onions, and water. Finally, meat and red wine were the main components of the dinner at the day’s end, but bread, cheese, and water were also included. These findings from our thematic analysis are in line with previous analyses on the dietary and culinary practices described in the Iliad,[^83-86^](#_ENREF_83) with biomolecular archaeology data of stable isotope analysis of relevant human and faunal remains,[^87^](#_ENREF_87) and with archaeological data on food production and the consumption of stock-farming animal species in the region.[^88-90^](#_ENREF_88)

Taking into account the above-mentioned requirement for energy balance and the food available / consumed at the time of Homer’s Iliad[^83-86^](#_ENREF_83) in conjunction with the findings of our 1^st^ thematic analysis and those performed by others,[^83-85^](#_ENREF_83) we estimated the daily nutrition plan of warriors during one day of the Trojan War (Table s3).

| **Table s3.** Daily nutrition plan of warriors during one day of the Trojan War and associated example for a 75-kg individual. | | | | | |
| --- | --- | --- | --- | --- | --- |
| **Meal** | **Food** | **Energy**  **(kcal / 100 g)**[**^91^**](#_ENREF_91) | **Percent of the meal** | **Nutrition for a 75 kg warrior** | |
|  |  |  |  | Food quantity (g) | Meal energy (kcal / meal) |
| Breakfast | Dry bread | 350 | 50 | 254 | 1,777  (40 %) |
|  | Goat cheese | 365 | 30 | 146 |  |
|  | Green olives | 145 | 18 | 221 |  |
|  | Red wine^1^ | 85 | 2 | 42 |  |
|  | Water^2^ | 0 | --- | --- |  |
| Snack | Dry bread | 350 | 55 | 70 | 444  (10 %) |
|  | Goat cheese | 365 | 35 | 43 |  |
|  | Onion^3^ | 40 | 10 | 111 |  |
|  | Water^2^ | 0 | --- | --- |  |
| Dinner | Red meat^4^ | 332 | 50 | 335 | 2,222  (50 %) |
|  | Dry bread | 350 | 31 | 197 |  |
|  | Goat cheese | 364 | 15 | 92 |  |
|  | Red wine^1^ | 85 | 4 | 105 |  |
|  | Water^2^ | 0 | --- | --- |  |
|  |  |  |  | **TOTAL** | **4,443** |
| Note: ^1^: a typical portion of wine is 175 ml;[^92^](#_ENREF_92) ^2^: *ad libitum* water consumption is assumed; ^3^: allium cepa; ^4^: from sheep, goat, pig, or cow. | | | | | |

### Section 2.5. The physical characteristics and the level of combat experience of elite warriors (topic 5)

Homer describes the majority of the warriors in the Iliad as strong, fast, and skilled middle-aged (probably between 20 and 40 years old) men. Also, many of the elite warriors are described as physically impressive and tall. While it is logical to suspect that the physique of ancient Greek and Trojan warriors was exaggerated and embellished in Homer’s oral poetry, archaeological evidence[^93-95^](#_ENREF_93) indicates that army warriors in the Achean army were likely to be larger and stronger than the average population. Indeed, skeletal remains from the Late Bronze Age suggest that the mean height of the average “Ancient Greek male” was 1.62 to 1.67 m,[^94^](#_ENREF_94)^,^[^96^](#_ENREF_96)^,^[^97^](#_ENREF_97) yet the height of the average warrior in the Iliad may have been 1.67 to 1.75 m,[^90^](#_ENREF_90)^,^[^97^](#_ENREF_97) while the height of the wearer of the Dendra armour was >1.77 m.[^1^](#_ENREF_1) This may be due to natural selection of warriors with larger and stronger bodies, or because the wealthy elite warriors ate better than the poor in ancient societies.

In regards to combat experience and armour usage, our 1^st^ thematic analysis suggested that the elite warriors had extensive fighting experience and wore well-made and functional full armour. The majority of their followers were experienced warriors (though having varying levels of skill, training, and fighting experience) and wore light or no armour. These conclusions concur with previous analyses of Homeric warfare.[^48^](#_ENREF_48)^,^[^74^](#_ENREF_74)^,^[^98-101^](#_ENREF_98)

### Section 2.6. The main types of combat (topic 6)

In agreement with available data on Homeric warfare,[^48^](#_ENREF_48)^,^[^74^](#_ENREF_74)^,^[^98-101^](#_ENREF_98) our 1^st^ thematic analysis suggested that, at a given time during battle, a number of elite warriors with their followers fought at the front line as “*foremost fighters*”, while others (referred to as “*multitude*”) would seek safety at the rear of the battle line or in the camp (see Section 2.3). Elite warriors and their groups did not permanently belong to either the *foremost fighters* or the *multitude*. Instead, the structure of both groups was constantly in flux, as elite warriors and their followers took turns at fighting. Some warriors spent more time fighting and others spent most time in safety, but everyone participated in fighting. Even the least capable fighters (such as Thersites) were worthy of taking prisoners in battle.[^52^](#_ENREF_52)

The cooperating groups which comprised the army – each with one or two leaders and their followers – would attack, retreat, recover, gain ground, and retreat again in a serial manner throughout the day.[^48^](#_ENREF_48)^,^[^98^](#_ENREF_98)^,^[^101^](#_ENREF_101) Attackers would use striking weapons or missiles to pursue their escaping enemies, yet they would retreat if their opponents turned to defend themselves. When victorious, individual warriors ceased fighting to loot their victims, reducing the pace of the offensive action. Leaders sometimes encouraged their men to maintain the pace of the advance which, despite these efforts, typically remained slow and dispersed. In total, fighting activity in the Iliad was characterised by hit-and-run tactics,[^48^](#_ENREF_48)^,^[^101^](#_ENREF_101) a form of physical effort described in physiology as “high-intensity interval exercise”.[^102^](#_ENREF_102)

To delve deeper into the main types of combat fighting in the Iliad and obtain the information required for a physiological analysis of Late Bronze Age warfare, our 2^nd^ thematic analysis included a detailed study of the fighting described by Homer. Based on this work, the Iliad described 152 encounters (Figure 3, main text, panel A). Comparisons using chi-square analysis showed that the vast majority (78 %) of the encounters described were “1-on-1” combats (i.e., foot warrior vs foot warrior) (χ^2^=487.2, p<0.001), which were typically between two group leaders. Encounters between a foot warrior and a chariot represented 12 % of the encounters, while the remaining 10 % of combats comprised of “chariot vs warrior on ship”, “chariot vs chariot”, “duels”, and “distance encounters”. The “1-on-1” combats were the most frequent type of encounter described across all days (χ^2^=46.9, p<0.001), ranging from 33 % of the encounters described in Day 4 to 82 % of the encounters described in Day 5 (Figure 3, main text, panel B). Encounters between a foot warrior and a chariot ranged from 10 % of the total in Days 2 and 5 to 20 % of all the encounters described in Day 7 (note: out of the 10 days described in the Iliad, only four days include combat fighting).

### Section 2.7. The typical techniques, movements, and weapons used by combatants (topic 7)

For the 152 encounters described in the Iliad, Homer mentions 359 combat moves in total, 95 of which were not described in detail (i.e., only the end-result was provided; Figure 3, main text, panel C). When analysing combat moves, we considered any kind of attempted / successful physical attack with either a part of the human body or with an inanimate object (such as a sword, spear, rock, or other ranged weapon) intended to inflict damage or harm to an enemy. All kinds of hand-to-hand combat (e.g., grabbing, choking, strangling, joint locking) were also considered. Of the 264 combat moves for which a detailed description is provided, two out of three (65 %) were ranged combats (where spears or even stones were thrown), while one out of three (35 %) was hand-to-hand combat (χ^2^=24.2, p<0.001; Figure 3, main text, panel C). This analysis showed that encounters in the Iliad were decided after 1 to 12 combat moves (Figure s4, panel A). The majority (53 %) of encounters ended after a single fatal move (χ^2^=402.9, p<0.001), while 80 % of the encounters ended within three combat moves. Chi-square analysis showed that the number of combat moves made in each encounter was similar across the days described in the Iliad (χ^2^=41.9, p=0.07; Figure s4, panel B). The encounters describing “chariot vs warrior on ship”, “chariot vs chariot”, and “distance encounters” ended within three combat moves (Figure 3, main text, panel D). In contrast, some of the “foot warrior vs chariot” and “1-on-1” encounters ended after 11 and 12 combat moves, respectively. Finally, the fate of “duels” was determined in four to six combat moves (Figure 3, main text, panel D).

| **Figure s4.** Combat moves in each encounter in total (panel A) and across the four days of battle (out of the 10 total days) described in the Iliad (panel B). Panel B is a two-level scatterplot with data points randomly distributed within each rectangle at the crossing of the horizontal and vertical axes. The colour of rectangles in the scatterplot indicates the frequency of data points included (darker shades indicate more data points). | |
| --- | --- |
| 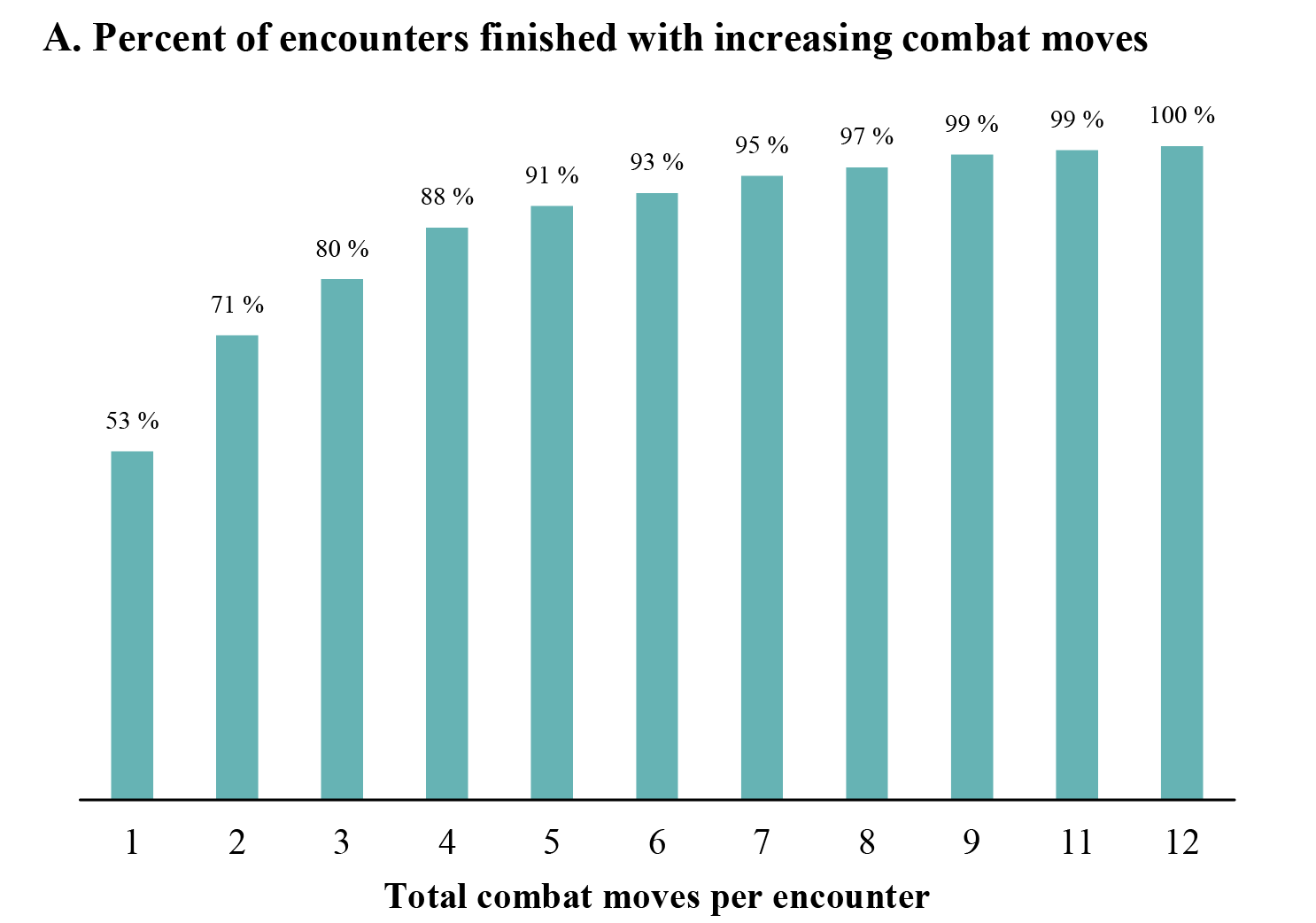 | 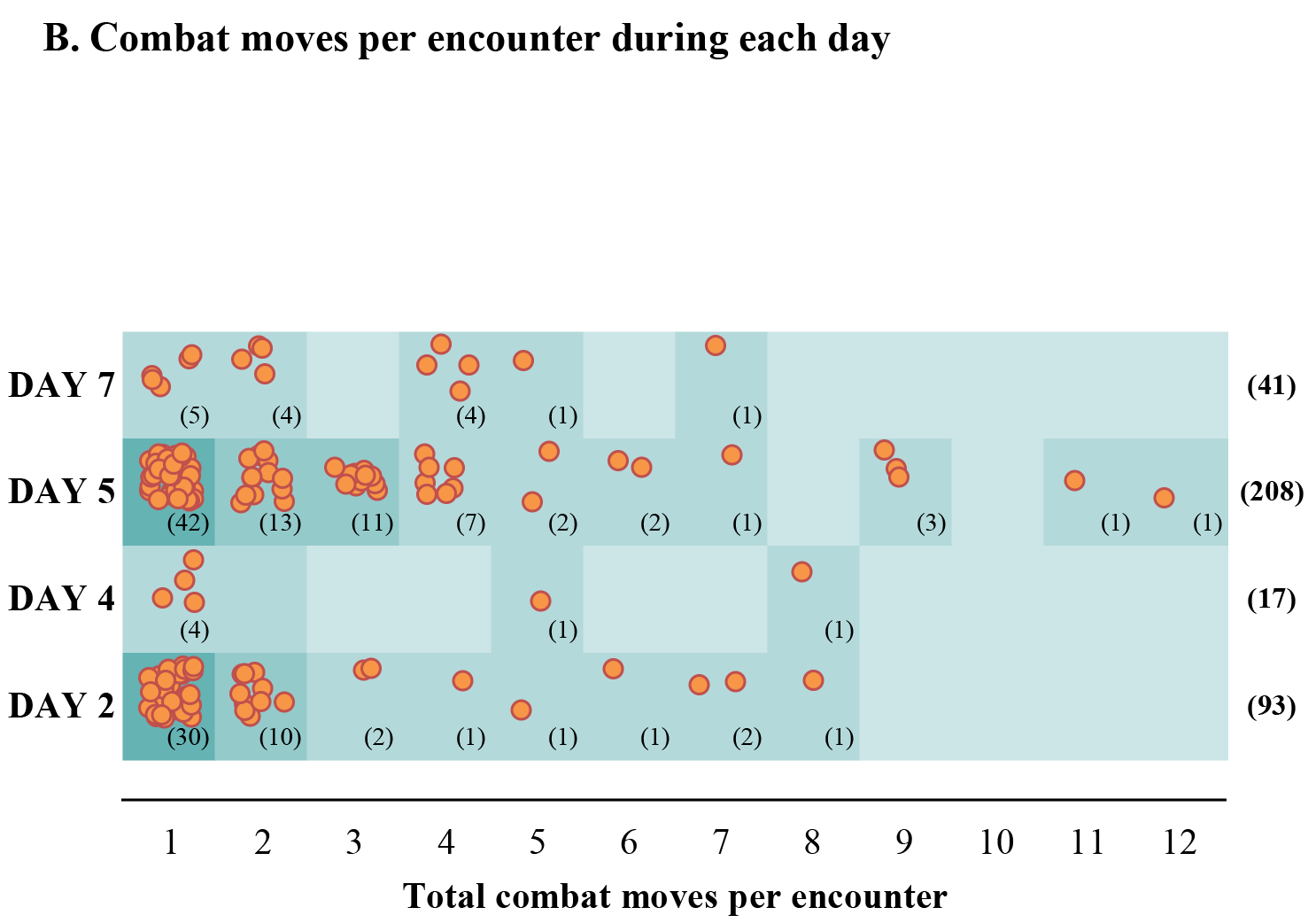 |

A total of 248 deaths and 31 non-fatal injuries were inflicted in the encounters described in the Iliad (Figure s5, panel A), the majority of which took place during Day 5 (χ^2^=17.9, p=0.04; Figure s5, panel B). Homer does not describe the area of the body targeted in half of the combat moves (Figure s5, panel C). In cases where such information is available, the chest, the head, and the upper limbs were the most commonly-targeted areas. This is probably because injuries to the chest and the head were the most likely areas to cause fatalities (Figure s5, panel D). The upper limbs were probably a frequent target due to their contribution in hand-to-hand combat, yet the vast majority of combat moves targeting this area were unsuccessful (Figure s5, panel D). The majority of fatalities were caused by hits on the chest (28 %), the head (24 %), the abdomen (16 %), and the neck (15 %) (Figure s5, panel E). On the other hand, non-fatal injuries were mostly caused by combat moves aiming at the upper limbs (32 %), the lower limbs (25 %), and the chest (21 %) (Figure s5, panel F).

In conclusion, our thematic analyses maintain that typical fighting took place at the front lines where a man selected an opponent and walked through the army lines as near as need be to unleash an attack, typically via ranged or close combat attack. He would then retreat to safety at the rear of the army to rest and recuperate. This pattern was characteristic in almost every encounter, suggesting that individual warriors were separated by marked distance. This is also supported by the fact that the majority of the attacks used ranged weapons.

| **Figure s5.** The outcome of the combat moves used by warriors (panels A and B), the areas of the body targeted by combat moves (panels C-D), as well as the areas of the body wounded when the victim died (panel E) or suffered an injury (panel F). Panels B and D are two-level scatterplots with data points randomly distributed within each rectangle at the crossing of the horizontal and vertical axes. The colour of rectangles in the scatterplots indicates the frequency of data points included (darker shades indicate more data points). | |
| --- | --- |
| 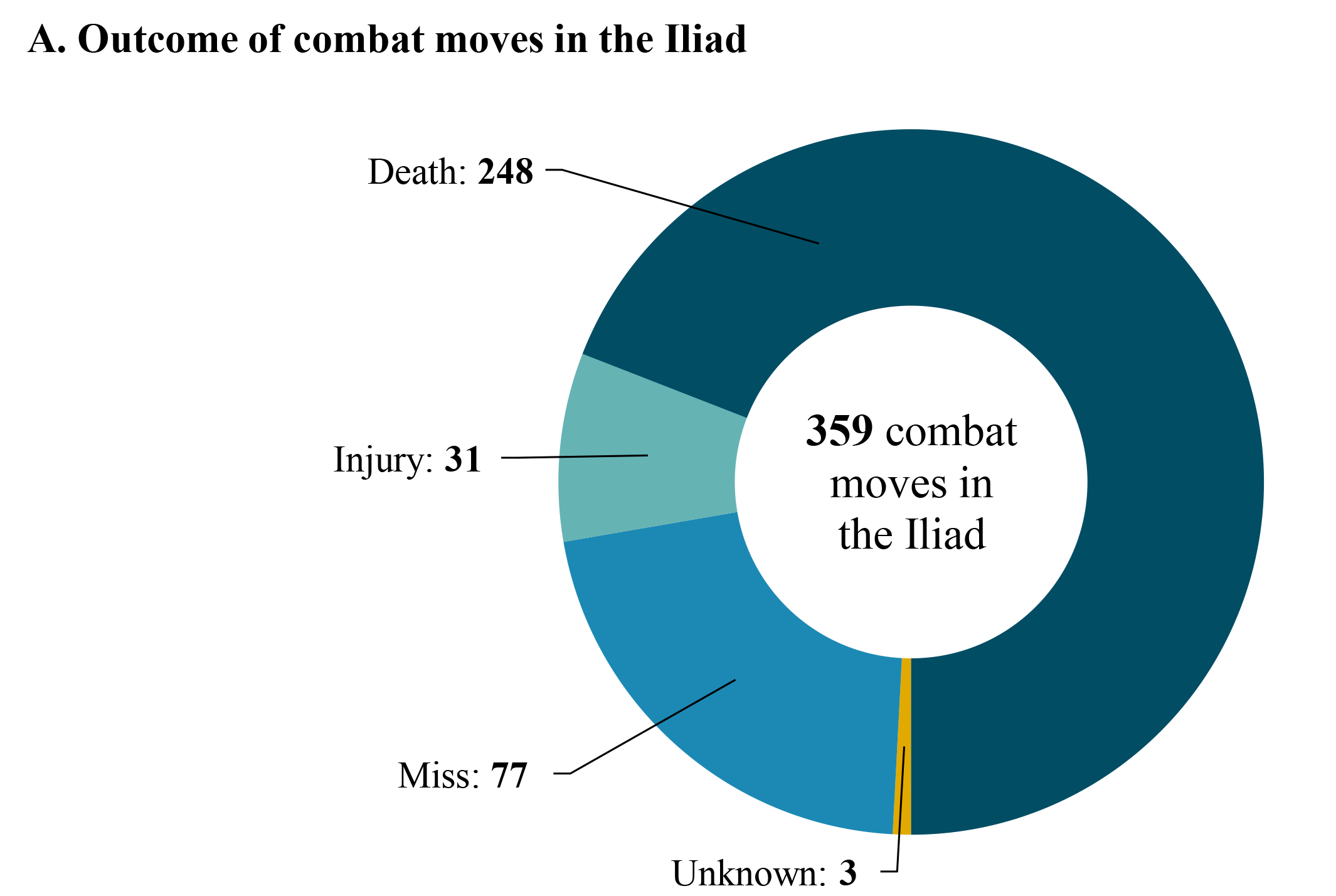 | 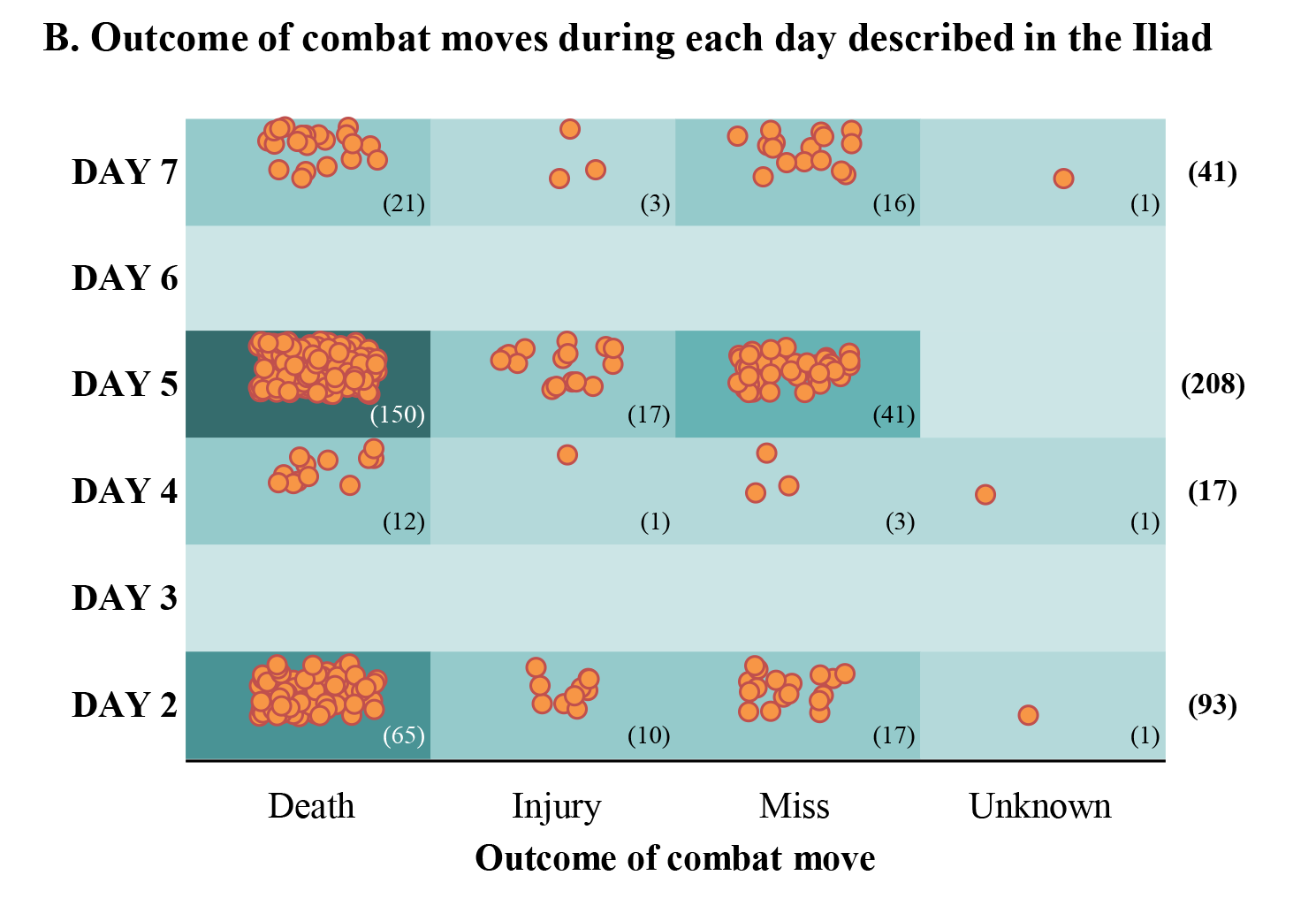 |
| 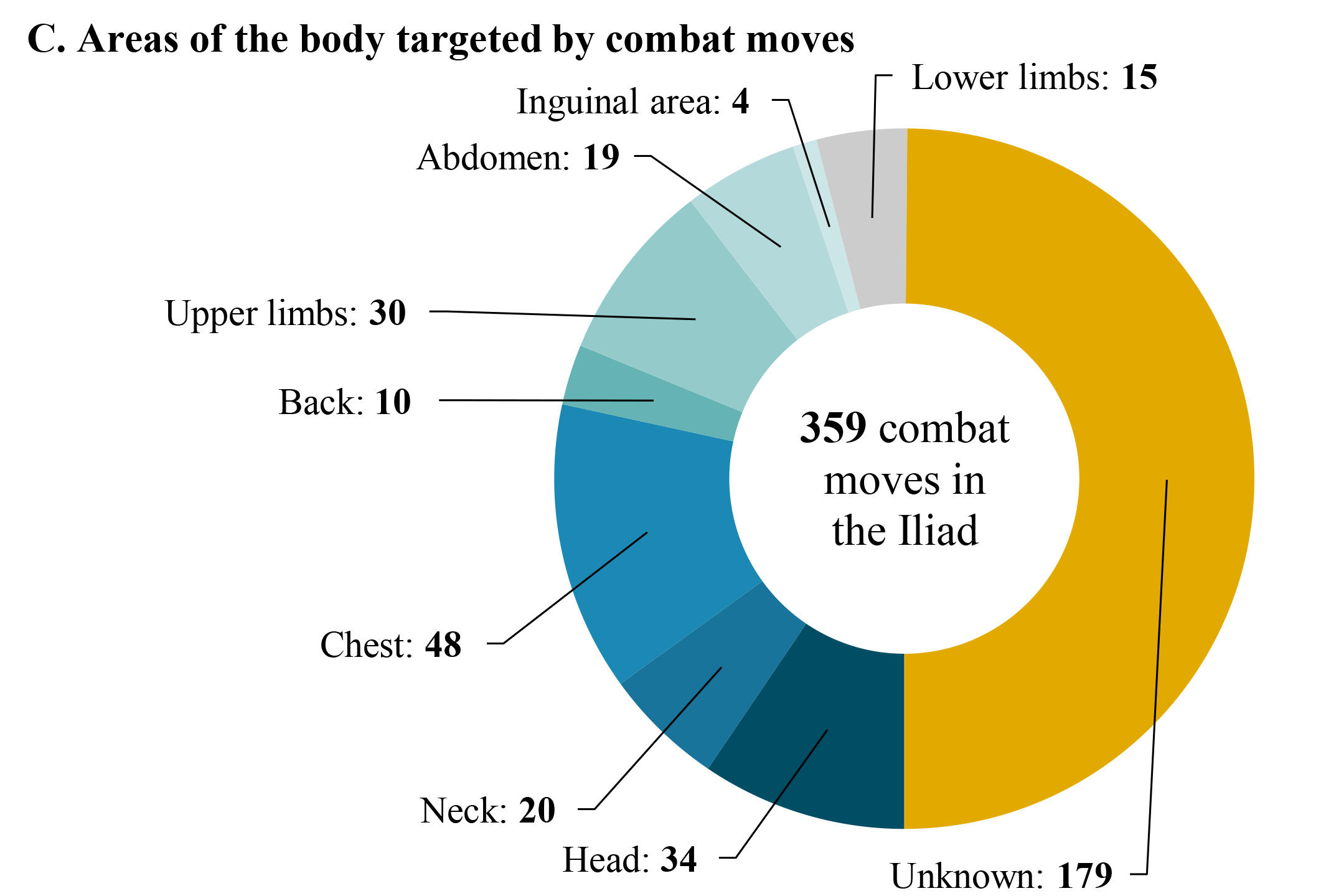 | 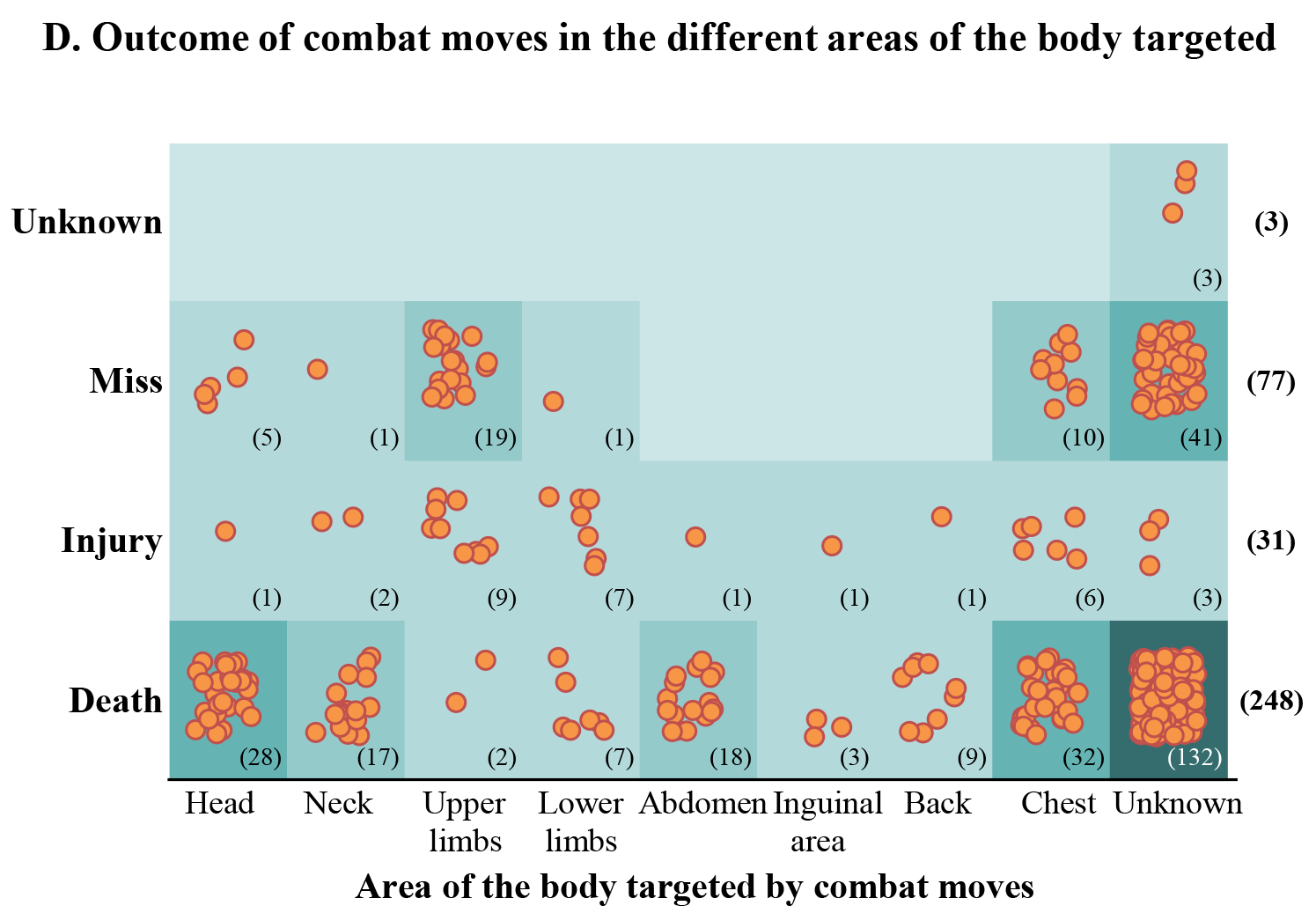 |
| 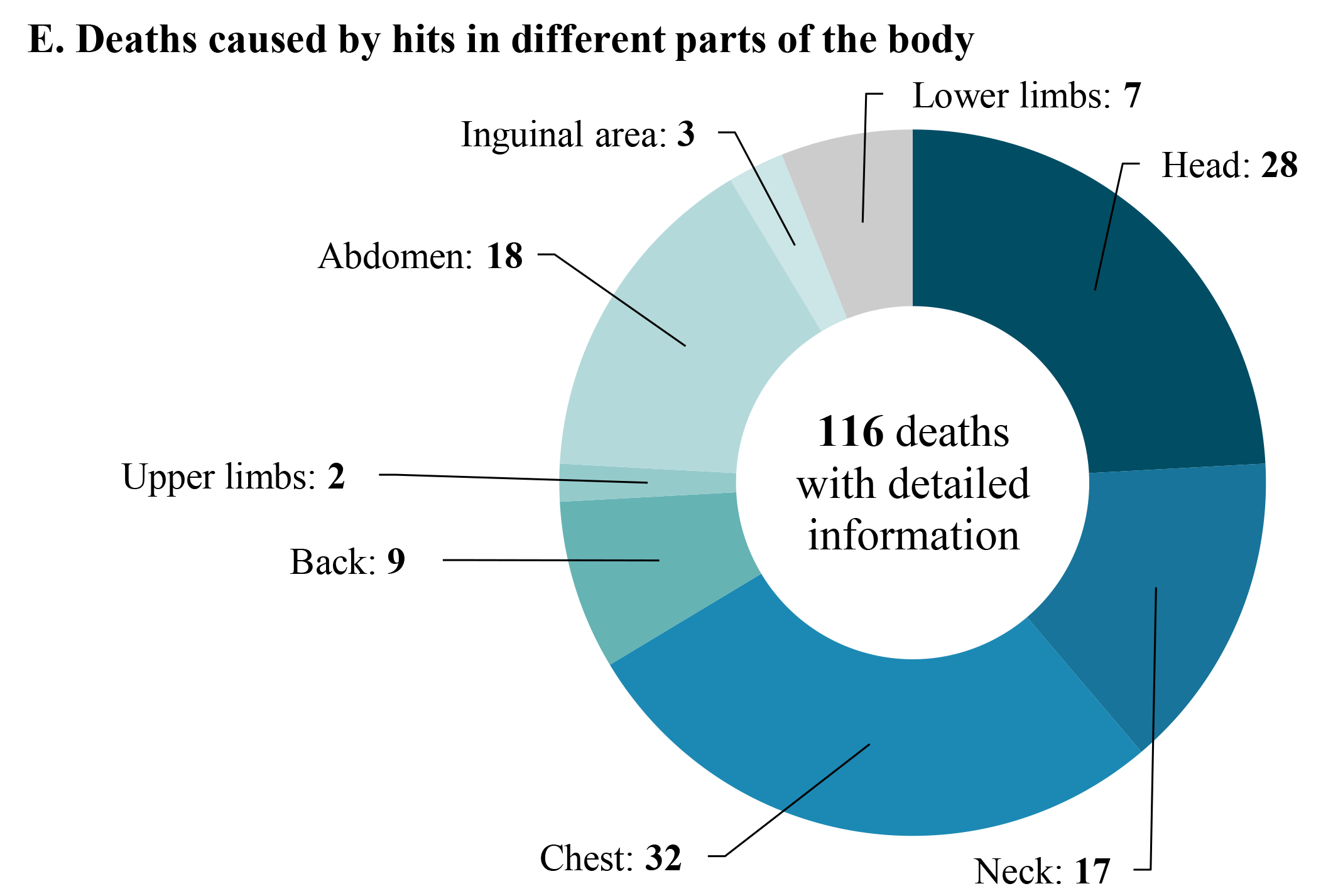 | 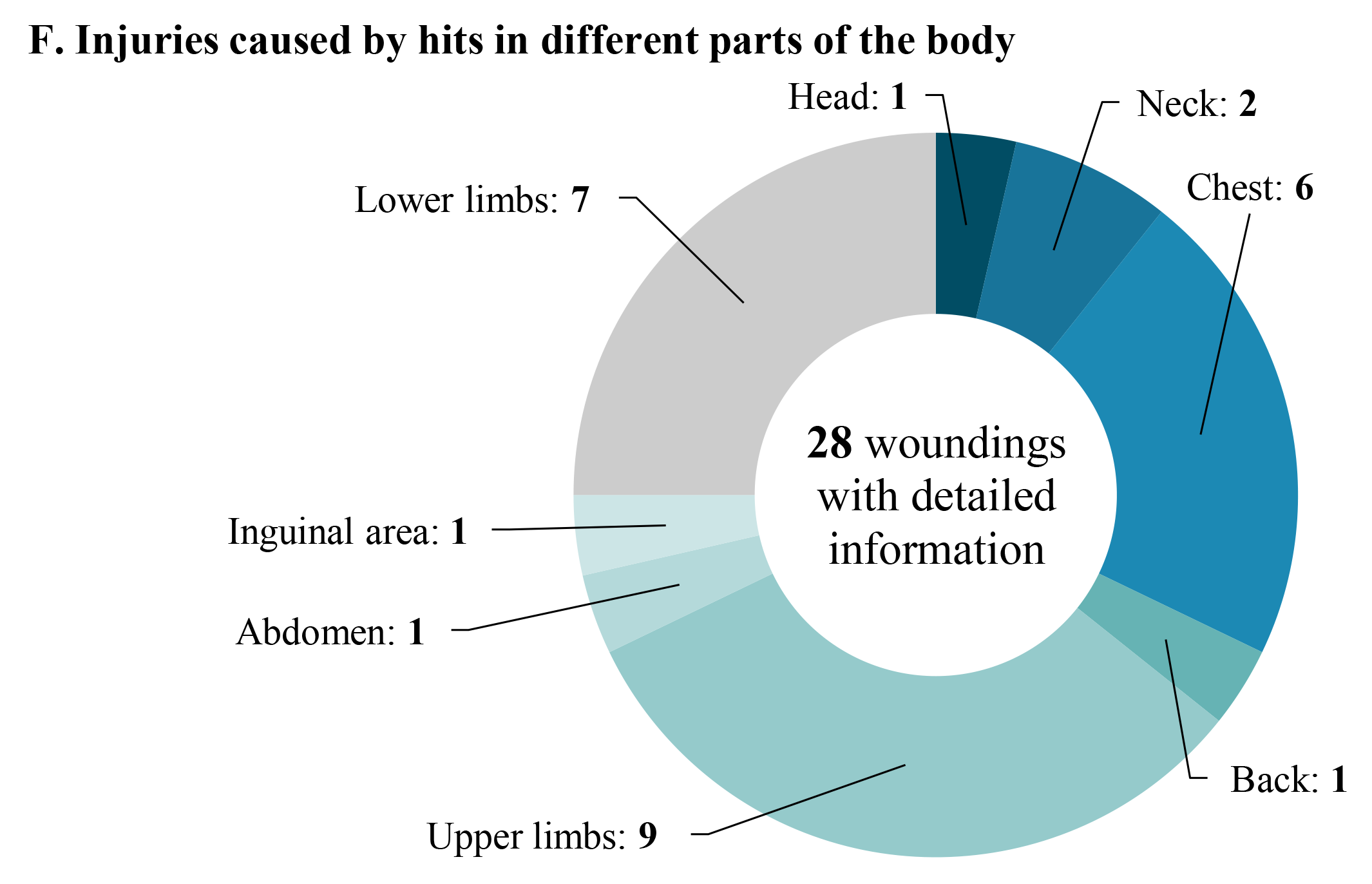 |

## Section 3. The Late Bronze Age combat simulation protocol of elite warriors (Study 2)

We used the information presented in Section 2 [made freely available in an online data repository (<https://doi.org/10.6084/m9.figshare.12090831.v1>)[^53^](#_ENREF_53)] to develop a combat simulation protocol of elite warriors during Late Bronze Age warfare. The information presented in Section 2.3 for the estimated time spent on typical activities performed by warriors during an average day of battle described in the Iliad provided the basis of the Late Bronze Age combat simulation protocol. To determine the activities during the time labelled as “combat” in Table s1, we used information which appears in Section 2.6-7. Specifically, the vast majority (96 %) of the encounters described in the Iliad were four types: (1) “1-on-1”, (2) “foot warrior vs chariot”, (3) “chariot vs warrior on ship”, and (4) “chariot vs chariot”. Accordingly, the combat portion of the Late Bronze Age combat simulation protocol included:

- 31 “1-on-1” encounters,
- 10 “foot warrior vs chariot” encounters,
- 2 “chariot vs chariot” encounters,
- 1 “chariot vs warrior on ship” encounter.

For each of these four types of encounter, we used the data derived from the 2^nd^ thematic analysis (see Section 2) and a two-step cluster analysis to identify the typical number of combat moves, weapons used, encounter phases (attack-defence), and areas of the body targeted (Table s4). To simulate the slow and dispersed pace of the fighting activity as well as the characteristic hit-and-run tactics (see Section 2.6), the four main types of encounters were interspersed within manoeuvring (on foot or on chariot) and break activities. The resulting Late Bronze Age combat simulation protocol is presented in Table s5. For the simulated elite warrior, “Deployment” and “Return to camp” means riding on a chariot at a speed of ~4 km/h towards the battlefield or the camp, respectively.[^75-77^](#_ENREF_75) “Battle formation” and “Disengagement” involves walking at a speed of 4 km/h to different areas of the battlefield.[^75-77^](#_ENREF_75) “Manoeuvring (on chariot)” means riding on a chariot at about 8 km/h,[^103^](#_ENREF_103) while “Manoeuvring (on foot)” involves walking at about 6 km/h.[^75^](#_ENREF_75)^,^[^76^](#_ENREF_76) Finally, “Break” involves resting seated.

| **Table s4.** Number of combat moves, weapons used, encounter phases, and areas of the body targeted in the four main types of encounters described in the Iliad. | | | | |
| --- | --- | --- | --- | --- |
| **Encounter type** | **Combat move** | **Weapon** | **Encounter phase** | **Targeted area** |
| 1-on-1 | 1 | Spear throw | Attack | Chest |
|  | 2 | Spear strike | Defence | Head |
|  | 3 | Spear strike | Attack | Upper limbs |
|  | 4 | Sword strike | Defence | Abdomen |
| Foot warrior vs chariot | 1 | Spear throw | Attack | Chest |
|  | 2 | Spear throw | Attack | Chest |
|  | 3 | Spear throw | Attack | Abdomen |
|  | 4 | Spear throw | Attack | Abdomen |
|  | 5 | Spear throw | Attack | Inguinal |
|  | 6 | Spear strike | Attack | Head |
|  | 7 | Spear strike | Attack | Head |
|  | 8 | Sword strike | Defence | Neck |
|  | 9 | Stone throw | Attack | Chest |
| Chariot vs chariot | 1 | Spear throw | Attack | Chest |
|  | 2 | Spear strike | Attack | Back |
| Chariot vs warrior on ship | 1 | Bow | Attack | Neck |
|  | 2 | Spear throw | Defence | Neck |
|  | 3 | Spear throw | Attack | Chest |

| **Table s5.** Late Bronze Age combat simulation protocol with activities performed by elite warriors during one day described in the Iliad. | | | | | |
| --- | --- | --- | --- | --- | --- |
| **#** | **Activity description** | **Tactics** | **Duration**  **(hh:min:sec)** | **Cumulative time (hh:min:sec)** | **Real time (hh:min:sec)** |
| 1 | Deployment | NA / OC | 1:30:00 | 1:30:00 | 7:00:00 |
| 2 | Battle formation | AO / OC | 0:30:00 | 2:00:00 | 8:30:00 |
| 3 | Manoeuvring (on foot) | AO / OF | 0:01:00 | 2:01:00 | 9:00:00 |
| 4 | Foot warrior vs chariot | AO / OF | 0:01:00 | 2:02:00 | 9:01:00 |
| 5 | Manoeuvring (on foot) | AO / OF | 0:01:30 | 2:03:30 | 9:02:00 |
| 6 | 1-on-1 | AO / OF | 0:01:00 | 2:04:30 | 9:03:30 |
| 7 | Manoeuvring (on foot) | AO / OF | 0:02:00 | 2:06:30 | 9:04:30 |
| 8 | Manoeuvring (on chariot) | AO / OC | 0:05:00 | 2:11:30 | 9:06:30 |
| 9 | Manoeuvring (on foot) | AO / OF | 0:01:00 | 2:12:30 | 9:11:30 |
| 10 | 1-on-1 | AO / OF | 0:01:00 | 2:13:30 | 9:12:30 |
| 11 | Manoeuvring (on foot) | AO / OF | 0:01:30 | 2:15:00 | 9:13:30 |
| 12 | 1-on-1 | AO / OF | 0:01:00 | 2:16:00 | 9:15:00 |
| 13 | Manoeuvring (on foot) | AO / OF | 0:02:00 | 2:18:00 | 9:16:00 |
| 14 | Manoeuvring (on chariot) | AO / OC | 0:05:00 | 2:23:00 | 9:18:00 |
| 15 | Break | NA / OF | 0:05:00 | 2:28:00 | 9:23:00 |
| 16 | Manoeuvring (on chariot) | AO / OC | 0:05:00 | 2:33:00 | 9:28:00 |
| 17 | Manoeuvring (on foot) | AO / OF | 0:01:00 | 2:34:00 | 9:33:00 |
| 18 | 1-on-1 | AO / OF | 0:01:00 | 2:35:00 | 9:34:00 |
| 19 | Manoeuvring (on foot) | AO / OF | 0:01:30 | 2:36:30 | 9:35:00 |
| 20 | 1-on-1 | AO / OF | 0:01:00 | 2:37:30 | 9:36:30 |
| 21 | Manoeuvring (on foot) | AO / OF | 0:02:00 | 2:39:30 | 9:37:30 |
| 22 | Manoeuvring (on chariot) | AO / OC | 0:05:00 | 2:44:30 | 9:39:30 |
| 23 | Manoeuvring (on foot) | AO / OF | 0:01:00 | 2:45:30 | 9:44:30 |
| 24 | Foot warrior vs chariot | AO / OF | 0:01:00 | 2:46:30 | 9:45:30 |
| 25 | Manoeuvring (on foot) | AO / OF | 0:01:30 | 2:48:00 | 9:46:30 |
| 26 | 1-on-1 | AO / OF | 0:01:00 | 2:49:00 | 9:48:00 |
| 27 | Manoeuvring (on foot) | AO / OF | 0:02:00 | 2:51:00 | 9:49:00 |
| 28 | Manoeuvring (on chariot) | AO / OC | 0:05:00 | 2:56:00 | 9:51:00 |
| 29 | Break | NA / OF | 0:05:00 | 3:01:00 | 9:56:00 |
| 30 | Manoeuvring (on chariot) | AO / OC | 0:05:00 | 3:06:00 | 10:01:00 |
| 31 | Manoeuvring (on foot) | AO / OF | 0:01:00 | 3:07:00 | 10:06:00 |
| 32 | 1-on-1 | AO / OF | 0:01:00 | 3:08:00 | 10:07:00 |
| 33 | Manoeuvring (on foot) | AO / OF | 0:01:30 | 3:09:30 | 10:08:00 |
| 34 | 1-on-1 | AO / OF | 0:01:00 | 3:10:30 | 10:09:30 |
| 35 | Manoeuvring (on foot) | AO / OF | 0:02:00 | 3:12:30 | 10:10:30 |
| 36 | Manoeuvring (on chariot) | AO / OC | 0:05:00 | 3:17:30 | 10:12:30 |
| 37 | Manoeuvring (on foot) | AO / OF | 0:01:00 | 3:18:30 | 10:17:30 |
| 38 | 1-on-1 | AO / OF | 0:01:00 | 3:19:30 | 10:18:30 |
| 39 | Manoeuvring (on foot) | AO / OF | 0:01:30 | 3:21:00 | 10:19:30 |
| 40 | 1-on-1 | AO / OF | 0:01:00 | 3:22:00 | 10:21:00 |

| **Table s5 cont.** Late Bronze Age combat simulation protocol with activities performed by elite warriors during one day described in the Iliad. | | | | | |
| --- | --- | --- | --- | --- | --- |
| **#** | **Activity description** | **Tactics** | **Duration**  **(hh:min:sec)** | **Cumulative time (hh:min:sec)** | **Real time (hh:min:sec)** |
| 41 | Manoeuvring (on foot) | AO / OF | 0:02:00 | 3:24:00 | 10:22:00 |
| 42 | Manoeuvring (on chariot) | AO / OC | 0:05:00 | 3:29:00 | 10:24:00 |
| 43 | Break | NA / OF | 0:05:00 | 3:34:00 | 10:29:00 |
| 44 | Manoeuvring (on chariot) | AO / OC | 0:05:00 | 3:39:00 | 10:34:00 |
| 45 | Manoeuvring (on foot) | AO / OF | 0:01:00 | 3:40:00 | 10:39:00 |
| 46 | Chariot vs chariot | AO / OF | 0:01:00 | 3:41:00 | 10:40:00 |
| 47 | Manoeuvring (on foot) | AO / OF | 0:01:30 | 3:42:30 | 10:41:00 |
| 48 | 1-on-1 combat | AO / OF | 0:01:00 | 3:43:30 | 10:42:30 |
| 49 | Manoeuvring (on foot) | AO / OF | 0:02:00 | 3:45:30 | 10:43:30 |
| 50 | Manoeuvring (on chariot) | AO / OC | 0:05:00 | 3:50:30 | 10:45:30 |
| 51 | Manoeuvring (on foot) | AO / OF | 0:01:00 | 3:51:30 | 10:50:30 |
| 52 | 1-on-1 combat | AO / OF | 0:01:00 | 3:52:30 | 10:51:30 |
| 53 | Manoeuvring (on foot) | AO / OF | 0:01:30 | 3:54:00 | 10:52:30 |
| 54 | 1-on-1 combat | AO / OF | 0:01:00 | 3:55:00 | 10:54:00 |
| 55 | Manoeuvring (on foot) | AO / OF | 0:02:00 | 3:57:00 | 10:55:00 |
| 56 | Manoeuvring (on chariot) | AO / OC | 0:05:00 | 4:02:00 | 10:57:00 |
| 57 | Break | NA / OF | 0:05:00 | 4:07:00 | 11:02:00 |
| 58 | Manoeuvring (on chariot) | AO / OC | 0:05:00 | 4:12:00 | 11:07:00 |
| 59 | Manoeuvring (on foot) | AO / OF | 0:01:00 | 4:13:00 | 11:12:00 |
| 60 | 1-on-1 combat | AO / OF | 0:01:00 | 4:14:00 | 11:13:00 |
| 61 | Manoeuvring (on foot) | AO / OF | 0:01:30 | 4:15:30 | 11:14:00 |
| 62 | 1-on-1 combat | AO / OF | 0:01:00 | 4:16:30 | 11:15:30 |
| 63 | Manoeuvring (on foot) | AO / OF | 0:02:00 | 4:18:30 | 11:16:30 |
| 64 | Manoeuvring (on chariot) | AO / OC | 0:05:00 | 4:23:30 | 11:18:30 |
| 65 | Manoeuvring (on foot) | AO / OF | 0:01:00 | 4:24:30 | 11:23:30 |
| 66 | 1-on-1 combat | AO / OF | 0:01:00 | 4:25:30 | 11:24:30 |
| 67 | Manoeuvring (on foot) | AO / OF | 0:01:30 | 4:27:00 | 11:25:30 |
| 68 | Chariot vs chariot | AO / OF | 0:01:00 | 4:28:00 | 11:27:00 |
| 69 | Manoeuvring (on foot) | AO / OF | 0:02:00 | 4:30:00 | 11:28:00 |
| 70 | Manoeuvring (on chariot) | AO / OC | 0:05:00 | 4:35:00 | 11:30:00 |
| 71 | Break | NA / OF | 0:05:00 | 4:40:00 | 11:35:00 |
| 72 | Manoeuvring (on chariot) | AO / OC | 0:05:00 | 4:45:00 | 11:40:00 |
| 73 | Manoeuvring (on foot) | AO / OF | 0:01:00 | 4:46:00 | 11:45:00 |
| 74 | 1-on-1 combat | AO / OF | 0:01:00 | 4:47:00 | 11:46:00 |
| 75 | Manoeuvring (on foot) | AO / OF | 0:01:30 | 4:48:30 | 11:47:00 |
| 76 | 1-on-1 combat | AO / OF | 0:01:00 | 4:49:30 | 11:48:30 |
| 77 | Manoeuvring (on foot) | AO / OF | 0:02:00 | 4:51:30 | 11:49:30 |
| 78 | Manoeuvring (on chariot) | AO / OC | 0:05:00 | 4:56:30 | 11:51:30 |
| 79 | Manoeuvring (on foot) | AO / OF | 0:01:00 | 4:57:30 | 11:56:30 |
| 80 | 1-on-1 combat | AO / OF | 0:01:00 | 4:58:30 | 11:57:30 |

| **Table s5 cont.** Late Bronze Age combat simulation protocol with activities performed by elite warriors during one day described in the Iliad. | | | | | |
| --- | --- | --- | --- | --- | --- |
| **#** | **Activity description** | **Tactics** | **Duration**  **(hh:min:sec)** | **Cumulative time (hh:min:sec)** | **Real time (hh:min:sec)** |
| 81 | Manoeuvring (on foot) | AO / OF | 0:01:30 | 5:00:00 | 11:58:30 |
| 82 | 1-on-1 combat | AO / OF | 0:01:00 | 5:01:00 | 12:00:00 |
| 83 | Manoeuvring (on foot) | AO / OF | 0:02:00 | 5:03:00 | 12:01:00 |
| 84 | Manoeuvring (on chariot) | AO / OC | 0:05:00 | 5:08:00 | 12:03:00 |
| 85 | Break | NA / OF | 0:05:00 | 5:13:00 | 12:08:00 |
| 86 | Manoeuvring (on chariot) | AO / OC | 0:30:00 | 5:43:00 | 12:13:00 |
| 87 | Manoeuvring (on foot) | AO / OF | 0:01:00 | 5:44:00 | 12:43:00 |
| 88 | 1-on-1 combat | AO / OF | 0:01:00 | 5:45:00 | 12:44:00 |
| 89 | Manoeuvring (on foot) | AO / OF | 0:01:30 | 5:46:30 | 12:45:00 |
| 90 | Chariot vs warrior on ship | AO / OF | 0:01:00 | 5:47:30 | 12:46:30 |
| 91 | Manoeuvring (on foot) | AO / OF | 0:02:00 | 5:49:30 | 12:47:30 |
| 92 | Manoeuvring (on chariot) | AO / OC | 0:05:00 | 5:54:30 | 12:49:30 |
| 93 | Manoeuvring (on foot) | AO / OF | 0:01:00 | 5:55:30 | 12:54:30 |
| 94 | 1-on-1 combat | AO / OF | 0:01:00 | 5:56:30 | 12:55:30 |
| 95 | Manoeuvring (on foot) | AO / OF | 0:01:30 | 5:58:00 | 12:56:30 |
| 96 | 1-on-1 combat | AO / OF | 0:01:00 | 5:59:00 | 12:58:00 |
| 97 | Manoeuvring (on foot) | AO / OF | 0:02:00 | 6:01:00 | 12:59:00 |
| 98 | Manoeuvring (on chariot) | AO / OC | 0:05:00 | 6:06:00 | 13:01:00 |
| 99 | Break | NA / OF | 0:06:00 | 6:12:00 | 13:06:00 |
| 100 | Manoeuvring (on chariot) | AO / OC | 0:05:00 | 6:17:00 | 13:12:00 |
| 101 | Manoeuvring (on foot) | AO / OF | 0:01:00 | 6:18:00 | 13:17:00 |
| 102 | 1-on-1 combat | AO / OF | 0:01:00 | 6:19:00 | 13:18:00 |
| 103 | Manoeuvring (on foot) | AO / OF | 0:01:30 | 6:20:30 | 13:19:00 |
| 104 | Foot warrior vs chariot | AO / OF | 0:01:00 | 6:21:30 | 13:20:30 |
| 105 | Manoeuvring (on foot) | AO / OF | 0:02:00 | 6:23:30 | 13:21:30 |
| 106 | Manoeuvring (on chariot) | AO / OC | 0:05:00 | 6:28:30 | 13:23:30 |
| 107 | Manoeuvring (on foot) | AO / OF | 0:01:00 | 6:29:30 | 13:28:30 |
| 108 | 1-on-1 combat | AO / OF | 0:01:00 | 6:30:30 | 13:29:30 |
| 109 | Manoeuvring (on foot) | AO / OF | 0:03:30 | 6:34:00 | 13:30:30 |
| 110 | Manoeuvring (on chariot) | AO / OC | 0:05:00 | 6:39:00 | 13:34:00 |
| 111 | Break | NA / OF | 0:06:00 | 6:45:00 | 13:39:00 |
| 112 | Manoeuvring (on chariot) | AO / OC | 0:05:00 | 6:50:00 | 13:45:00 |
| 113 | Manoeuvring (on foot) | AO / OF | 0:01:00 | 6:51:00 | 13:50:00 |
| 114 | Foot warrior vs chariot | AO / OF | 0:01:00 | 6:52:00 | 13:51:00 |
| 115 | Manoeuvring (on foot) | AO / OF | 0:03:30 | 6:55:30 | 13:52:00 |
| 116 | Manoeuvring (on chariot) | AO / OC | 0:05:00 | 7:00:30 | 13:55:30 |
| 117 | Manoeuvring (on foot) | AO / OF | 0:01:00 | 7:01:30 | 14:00:30 |
| 118 | 1-on-1 combat | AO / OF | 0:01:00 | 7:02:30 | 14:01:30 |
| 119 | Manoeuvring (on foot) | AO / OF | 0:03:30 | 7:06:00 | 14:02:30 |
| 120 | Manoeuvring (on chariot) | AO / OC | 0:05:00 | 7:11:00 | 14:06:00 |

| **Table s5 cont.** Late Bronze Age combat simulation protocol with activities performed by elite warriors during one day described in the Iliad. | | | | | |
| --- | --- | --- | --- | --- | --- |
| **#** | **Activity description** | **Tactics** | **Duration**  **(hh:min:sec)** | **Cumulative time (hh:min:sec)** | **Real time (hh:min:sec)** |
| 121 | Break | NA / OF | 0:06:00 | 7:17:00 | 14:11:00 |
| 122 | Manoeuvring (on chariot) | AO / OC | 0:05:00 | 7:22:00 | 14:17:00 |
| 123 | Manoeuvring (on foot) | AO / OF | 0:01:00 | 7:23:00 | 14:22:00 |
| 124 | 1-on-1 combat | AO / OF | 0:01:00 | 7:24:00 | 14:23:00 |
| 125 | Manoeuvring (on foot) | AO / OF | 0:03:30 | 7:27:30 | 14:24:00 |
| 126 | Manoeuvring (on chariot) | AO / OC | 0:05:00 | 7:32:30 | 14:27:30 |
| 127 | Manoeuvring (on foot) | AO / OF | 0:01:00 | 7:33:30 | 14:32:30 |
| 128 | 1-on-1 combat | AO / OF | 0:01:00 | 7:34:30 | 14:33:30 |
| 129 | Manoeuvring (on foot) | AO / OF | 0:03:30 | 7:38:00 | 14:34:30 |
| 130 | Manoeuvring (on chariot) | AO / OC | 0:05:00 | 7:43:00 | 14:38:00 |
| 131 | Break | NA / OF | 0:06:00 | 7:49:00 | 14:43:00 |
| 132 | Manoeuvring (on chariot) | AO / OC | 0:05:00 | 7:54:00 | 14:49:00 |
| 133 | Manoeuvring (on foot) | AO / OF | 0:01:00 | 7:55:00 | 14:54:00 |
| 134 | 1-on-1 combat | AO / OF | 0:01:00 | 7:56:00 | 14:55:00 |
| 135 | Manoeuvring (on foot) | AO / OF | 0:03:30 | 7:59:30 | 14:56:00 |
| 136 | Manoeuvring (on chariot) | AO / OC | 0:05:00 | 8:04:30 | 14:59:30 |
| 137 | Manoeuvring (on foot) | AO / OF | 0:01:00 | 8:05:30 | 15:04:30 |
| 138 | 1-on-1 combat | AO / OF | 0:01:00 | 8:06:30 | 15:05:30 |
| 139 | Manoeuvring (on foot) | AO / OF | 0:03:30 | 8:10:00 | 15:06:30 |
| 140 | Manoeuvring (on chariot) | AO / OC | 0:05:00 | 8:15:00 | 15:10:00 |
| 141 | Break | NA / OF | 0:06:00 | 8:21:00 | 15:15:00 |
| 142 | Manoeuvring (on chariot) | AO / OC | 0:05:00 | 8:26:00 | 15:21:00 |
| 143 | Manoeuvring (on foot) | AO / OF | 0:01:00 | 8:27:00 | 15:26:00 |
| 144 | Foot warrior vs chariot | AO / OF | 0:01:00 | 8:28:00 | 15:27:00 |
| 145 | Manoeuvring (on foot) | AO / OF | 0:03:30 | 8:31:30 | 15:28:00 |
| 146 | Manoeuvring (on chariot) | AO / OC | 0:05:00 | 8:36:30 | 15:31:30 |
| 147 | Manoeuvring (on foot) | AO / OF | 0:01:00 | 8:37:30 | 15:36:30 |
| 148 | 1-on-1 combat | AO / OF | 0:01:00 | 8:38:30 | 15:37:30 |
| 149 | Manoeuvring (on foot) | AO / OF | 0:03:30 | 8:42:00 | 15:38:30 |
| 150 | Manoeuvring (on chariot) | AO / OC | 0:05:00 | 8:47:00 | 15:42:00 |
| 151 | Break | NA / OF | 0:06:00 | 8:53:00 | 15:47:00 |
| 152 | Disengagement | AO / OC | 0:21:00 | 9:14:00 | 15:53:00 |
| 153 | Return to camp | NA / OC | 1:40:00 | 10:54:00 | 16:14:00 |
|  |  |  |  |  | 17:54:00 |
| Note: NA: warrior not wearing armour; OC: warrior on chariot; AO: warrior armour on; OF: warrior on foot. | | | | | |

## Section 4. Experimental study of the physiological stress during Late Bronze Age warfare (Study 3)

### Section 4.1. Concept and scope

Understanding the physical effort exerted and the physiological adaptations involved in the associated activities are essential to an analysis of the physiology involved in Late Bronze Age warfare. Therefore, we used the information from Studies 1 and 2 (Section 2) and the Late Bronze Age combat simulation protocol developed from them (Section 3) to conduct a human laboratory experiment where individuals underwent physiological measurements while performing the developed protocol (Table s5). The study was performed in controlled environmental settings (Figure s19) simulating the estimated ambient conditions (evaluated via a Kestrel 5400FW meter, Nielsen-Kellerman, Pennsylvania, USA) of the fighting described in the Iliad (Section 2.1), while participants wore a replica of the Dendra armour and carried replicas of Late Bronze Age weapons.

### Section 4.2. Study participants and ethics

A total of 13 males (Marines from the Hellenic Armed Forces; age: 29.2 ± 7.9 years; height: 1.73 ± 0.05 m; 74.1 ± 6.8 kg weight) volunteered. They were selected to fit as much as possible the age and anthropometric characteristics of the elite warriors described in the Iliad (Section 2.5). Prior to participating, they completed the Physical Activity Readiness Questionnaire to ensure that there were no health contraindications to performing extended strenuous physical exercise. They also read and signed an informed consent form approved by the institutional Ethics Review Board which approved the study, as well as an informed consent to publish the information/images/photographs taken during the study in an online open access publication. The experimental protocol adhered to the Declaration of Helsinki and was approved by the Bioethical Committee at the School of Exercise Science of the University of Thessaly (protocol number: 1098) and the Hellenic Army General Staff (Φ.300/74/449322 - Σ.2354 - Athens, Oct. 19, 2017). The individuals appearing in Figures 2, 7, s6, s16, and s17 in this manuscript have given written informed consent (as outlined in PLOS consent form) to publish these photographs. Extensive familiarization for all tests was undertaken prior to the study and all participants were free to cease their participation at any point before, during, or after the data collection and request that their data be removed.

### Section 4.3. Experimental protocol

Prior to undergoing any assessments, all participants were trained by the co-author SBP, a military officer and Licensed Instructor in Japanese fencing, martial arts, ancient combat tactics, and weapons (Figure s6). This 2-day training in groups was focused on familiarization and correct application of Late Bronze Age combat protection equipment and technology, correct and safe use of weaponry, as well as the different combinations of combat fighting techniques (close and middle range; Table s4) of the Late Bronze Age combat simulation protocol (Table s5). When necessary, additional individual training sessions were held. All participants underwent enough training to be deemed qualified by the Instructor for the unrestricted and safe use of the techniques, weapons, and armour used in the study.

Participants arrived at the laboratory for the main part of the investigation in the late afternoon (1730 – 1800 h) of the day prior to the day of testing. They were requested to abstain from caffeine since the morning of that day and they ate dinner (with *ad libitum* water) according to their weight-adjusted nutrition plan (see Section 2.4). Then, they toured the lab space and were provided with a step-by-step description of all procedures that would take place on the next day. The principal investigator and / or a senior member of the research team was present at all times to answer any questions. Participants were requested to retire to bed at 2200 h and were woken at 0530 h according to the procedures outlined in Section 2. At 0600 h, they were provided with *ad libitum* water and a breakfast according to their weight-adjusted nutrition plan (see Section 2.4). At 0630 h, volunteers were weighed and gave a urine sample for the determination of hydration

| **Figure s6.** Photographs taken during the training of the study participants in fencing, martial arts, military combat tactics, and weapons. | | | | |
| --- | --- | --- | --- | --- |
| 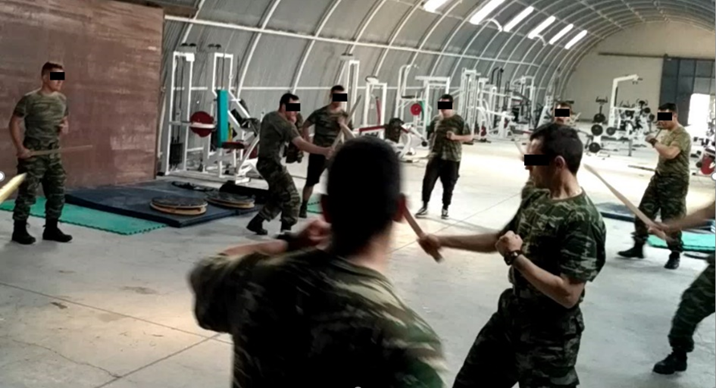 | 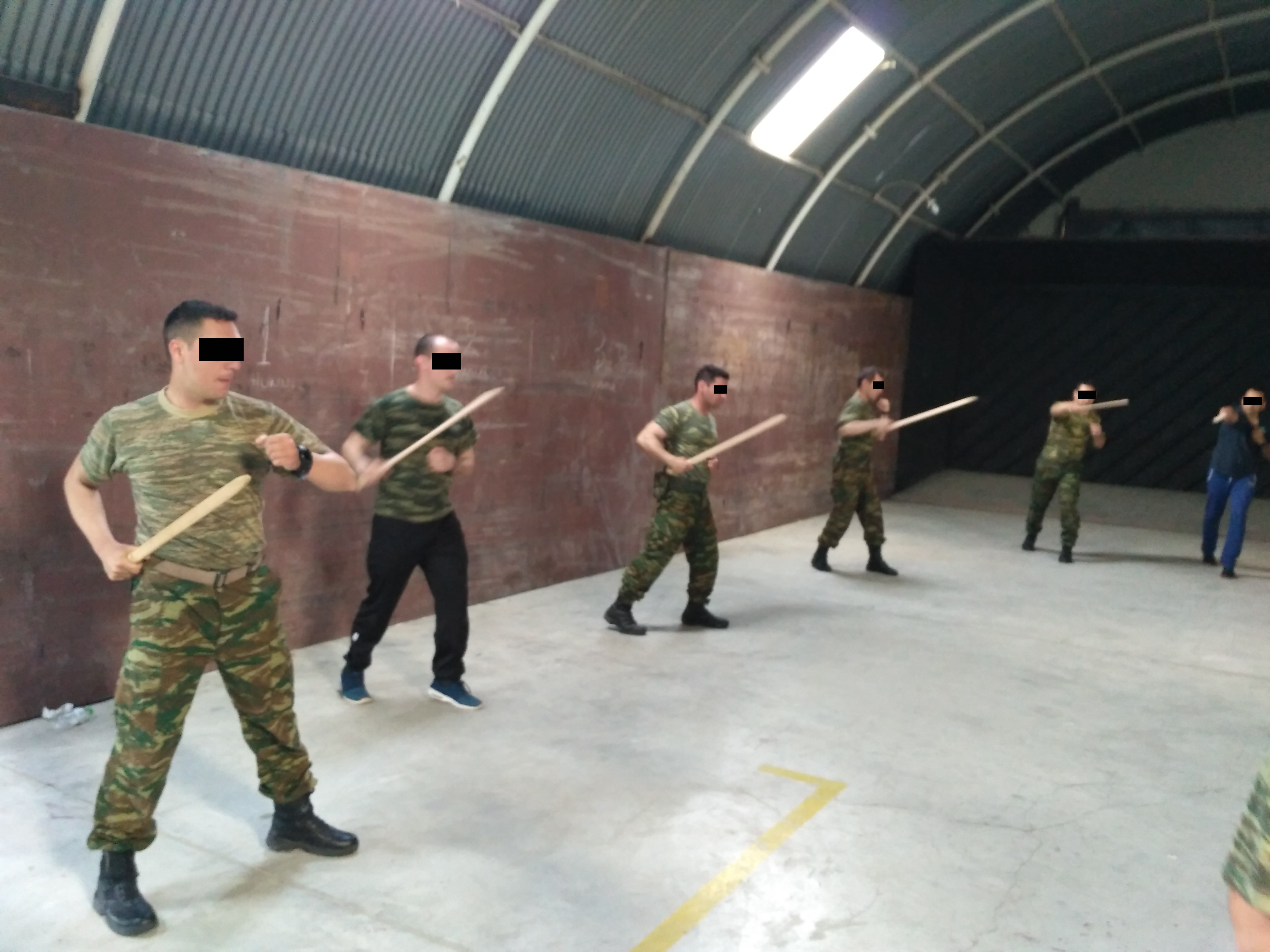 | | 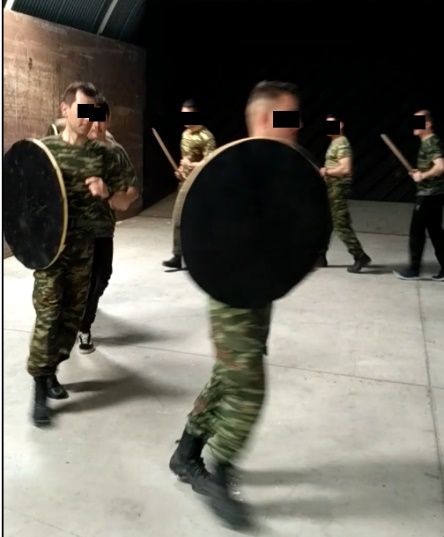 | 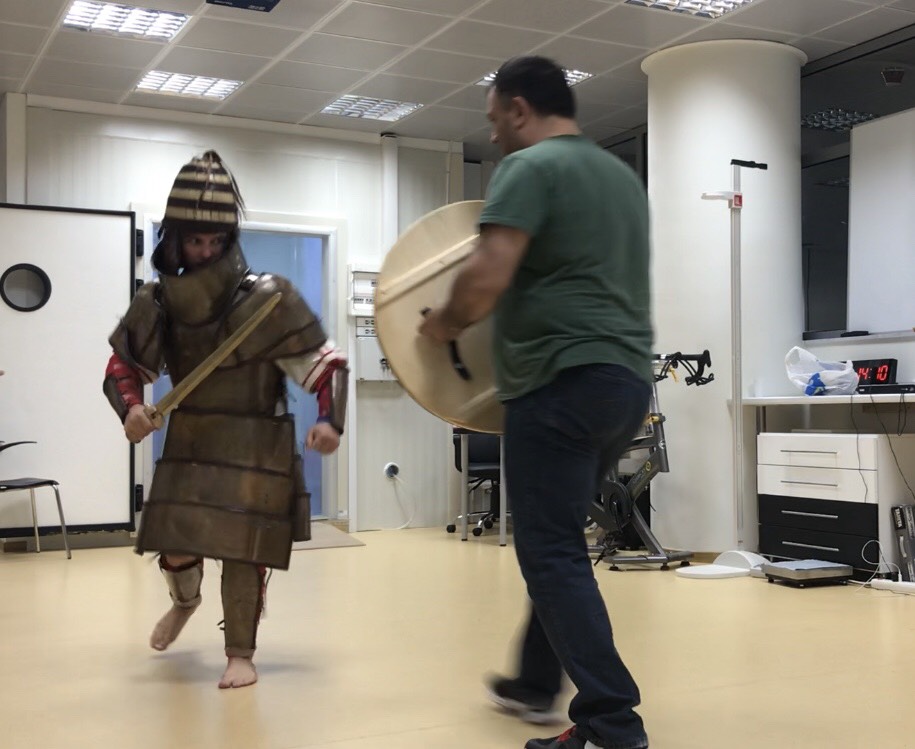 |
| 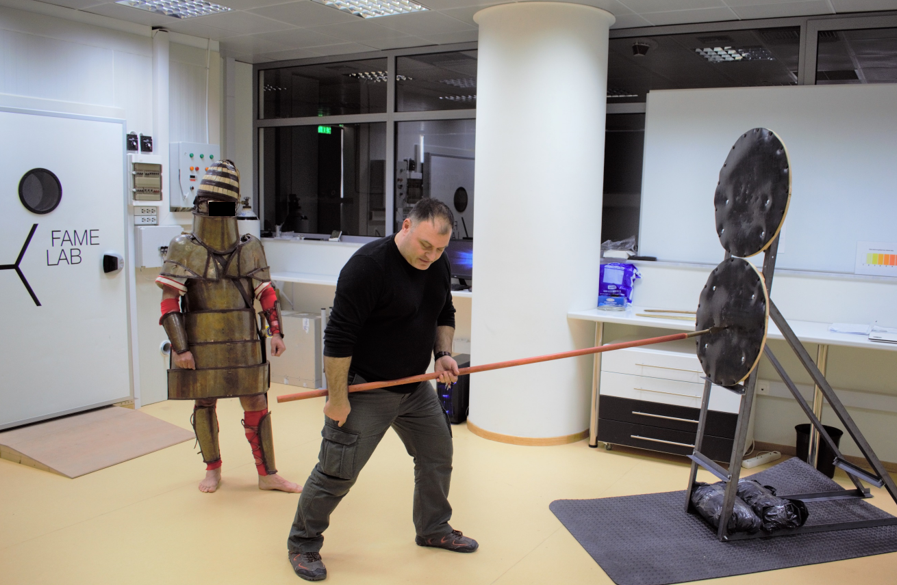 | | 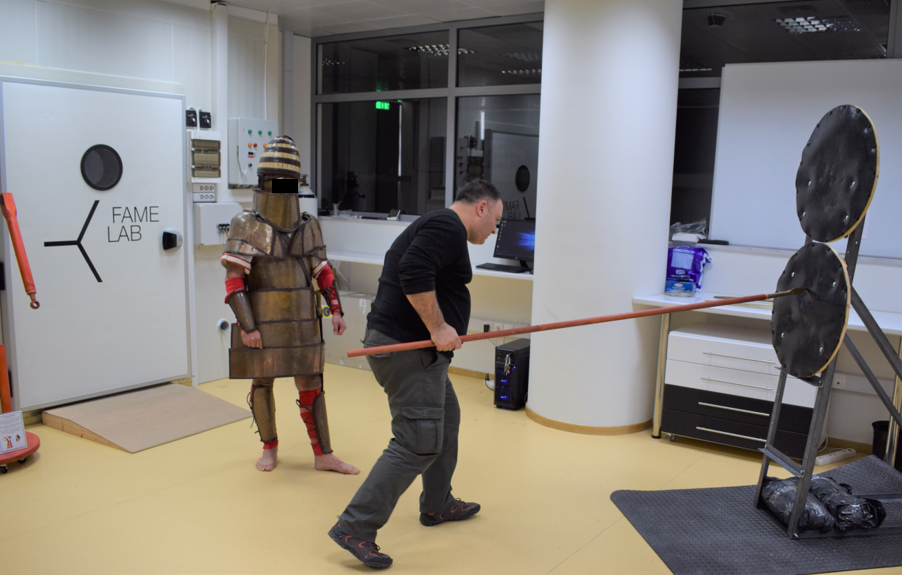 | | |

status. Thereafter, all sensors were placed and a blood sample was taken at 0655 h. The Late Bronze Age combat simulation protocol (Table s5) was initiated precisely at 07:00 and timing was followed very carefully throughout until its completion, at 17:54. During the various phases of the Late Bronze Age combat simulation protocol, participants performed the combat move combinations outlined in Table s4 and were continually encouraged to exert maximal effort. During the breaks included in the Late Bronze Age combat simulation protocol, participants were allowed to consume *ad libitum* water as well as snacks according to their weight-adjusted nutrition plan (see Section 2.4). Once the Late Bronze Age combat simulation protocol was completed, participants gave a urine sample and a second blood sample was taken.

In addition to the measurements described above, the following parameters were assessed before, within (during breaks at 0951, 1057, 1203, 1334, 1510, 1542 h; as shown in Table s5), and immediately after the Late Bronze Age combat simulation protocol: blood glucose concentration, blood lactate levels, hand grip strength, reaction time to visual and auditory stimuli, as well as self-perceived rate of exhaustion, thermal comfort, and thermal sensation. Heart rate, core body temperature, and mean skin temperature were continuously assessed throughout the Late Bronze Age combat simulation protocol. Finally, we assessed the energy cost of each activity via a portable gas analyser. For the latter measurement, we randomly evaluated small parts of the Late Bronze Age combat simulation protocol for some participants to avoid interrupting the flow of combat and thus limiting the participants’ maximum effort.

### Section 4.4. Experimental setup

Given that the Late Bronze Age combat simulation protocol included riding a chariot and walking for significant amounts of time, as well as performing predetermined scenarios of close combat with combinations of weapons and fighting techniques, we designed and prepared the equipment necessary for a valid laboratory simulation of these activities (Figure s7). A wooden chariot skeleton was created and connected to a 4-hp treadmill motor to simulate the variable speed and motion of a ‘real’ chariot for the participants to ride.[^77^](#_ENREF_77) As a result, participants were required to maintain some balance during their ride which, together with the (purposefully designed) constant rocking motion of the chariot and the noise of the wheels turning, generated a level of psychophysical fatigue that would have been close to that exerted by real chariot riding as described in the Iliad.[^77^](#_ENREF_77) In addition, a treadmill was placed near the chariot and, next to them, was a 30 m^2^ open area where participants performed the martial arts and combat moves prescribed by the Late Bronze Age combat simulation protocol (Figure s7). In this area, participants performed the combat phases of the combat simulation protocol and were instructed to hit the centre of a round target (65 cm diameter) made of thick beech wood (2 cm thick) faced with leather (0.5 cm thick). The target was mounted at a height of 1.7 m (centre of the target) onto a force plate (Bertec FP4060-15-2000, Bertec Corp USA, with load capacity of 10,000 N at the Vertical axis and 5,000 N at the Horizontal axis) which was, in turn, mounted to a wall.

| **Figure s7.** Construction of the chariot used (top) and experimental setup in the lab (bottom). |
| --- |
| 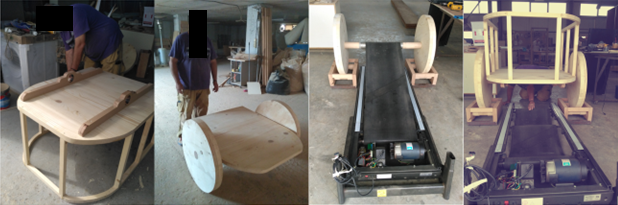 |
| 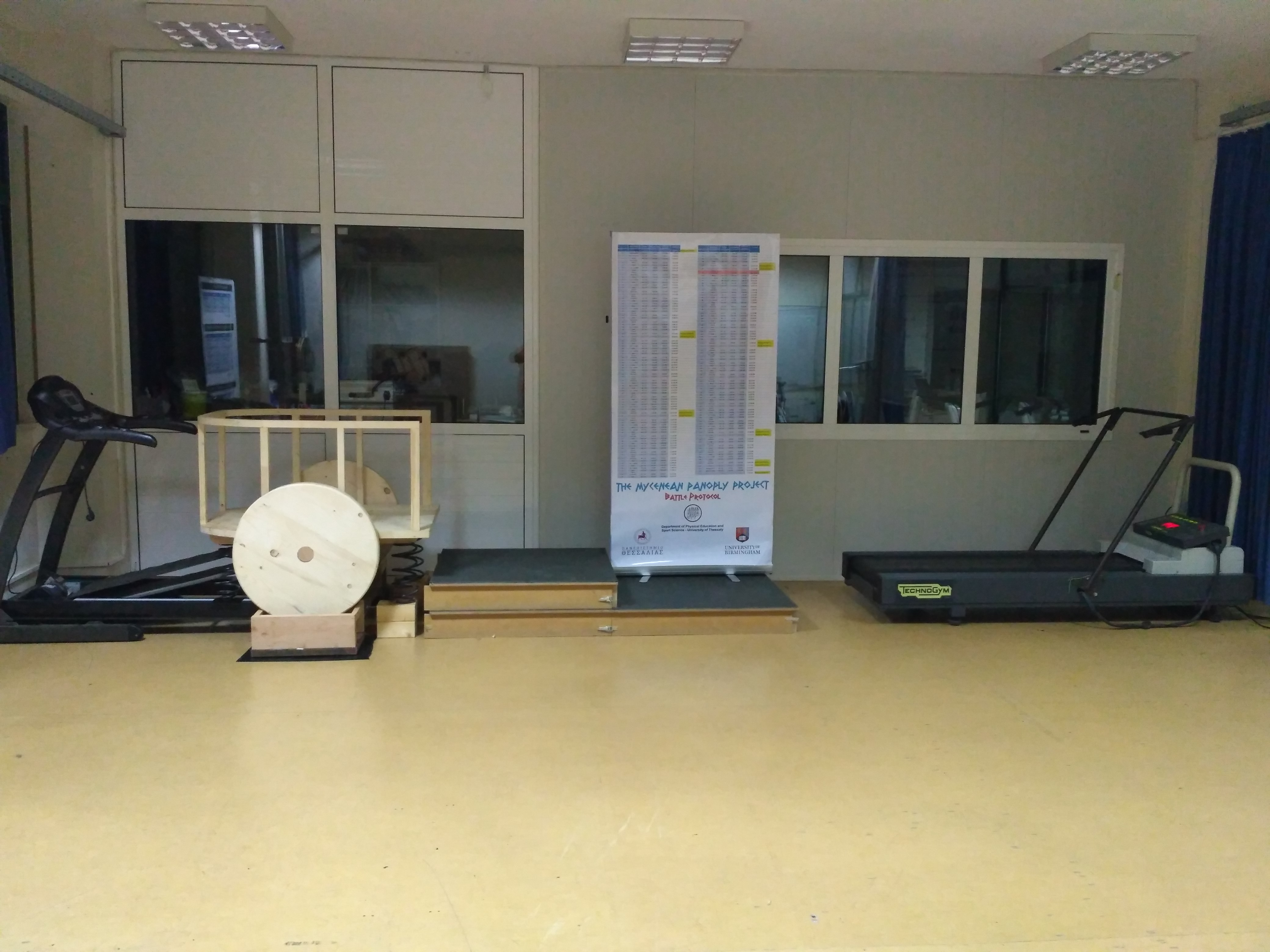 |

### Section 4.5. The Dendra armour replica

The Dendra armour is an artefact discovered in tomb no. 12 in the Mycenaean cemetery that was first explored in 1926 near the village of Dendra, in the Argolid, Greece.[^1^](#_ENREF_1) It is one of the earliest examples of hammered beaten bronze armour – dated to the Late Bronze Age c. 1450 BC. An attempt by looters to rob the tomb early in 1960 was foiled, though not before some items had been removed.[^1^](#_ENREF_1) The original Dendra armour is on exhibition in the Archaeological Museum of Nafplion, Greece. The panoply consisted of 18 or 19 parts (depending on whether a second arm guard was originally present) that were held together with leather thongs:

- Parts 1-2: a breast plate and a longer back plate: the right arm hole is cut deeper front and back and from this we infer that the owner used his sword or other weapon with his right hand. These plates were hinged/wired together on one side and fastened on the other with clips.
- Parts 3-8: three broad over-lapping bands front and back, hanging from the two main plates like a skirt: those in the front were made deeper to compensate for the shorter front plate which enabled the wearer to bend forward at the waist.
- Parts 9-10: two shoulder guards (pauldrons), each attached at a single point on the shoulders, which are elaborated with two refinements (parts 11-14).
- Parts 11-12: sliding extensions to cover the arms above the elbow and provide protection and ease of movement.
- Parts 13-14: small triangles of metal loosely fixed to the front of each shoulder guard provide vital protection when the arm is raised since they still cover the vulnerable armpit which, without them, would be unprotected. If these pieces were fixed as an elongation of the shoulder guard, they would overlap and catch when the arms were moved.
- Part 15: a cylindrical neck piece (gorget) covering the throat to the cheeks. A slight raise of the shoulders would reduce the gap between this and a helmet to a narrow eye-slit.
- Parts 16-17: a pair of greaves.
- Parts 18-19(?): only one lower arm guard (vambrace) was found, leading to the erroneous conclusion that the wearer was an archer. The second guard may have been lost or stolen since the tomb was partly robbed before the full excavation.

The whole panoply must have been made for a specific individual, as many of the pieces have been ‘tailor-made’, for example, the shaping on the back of the neck. Drilled holes in the metal for joining the plates indicate where they were attached to each other with leather thonging, presumably raw-hide, though the single pairs of attachment holes for the shoulder guards suggests at least some were wired in place since leather would chafe through too readily under the strain. Some traces of leather were preserved on the inside surface of the bronze pieces but it is not clear whether these were a lining or the traces of a leather jerkin or gambison. The armour could hardly have been worn without padding in some form, whether integral to it or worn as a separate garment.

X-ray fluorescence spectrometry analyses of two separate components of the armour were carried out in April 1985 by Dr Richard Jones of the British School at Athens Fitch Laboratory.[^104^](#_ENREF_104)^,^[^105^](#_ENREF_105) Both components had good metallic surfaces with some dark copper oxide visible and gave the following semiquantitative results, suggesting homogeneity in the composition of the bronze:

- Lower arm-guard[^1^](#_ENREF_1)^(Plate XXI)^ average: 10% Sn, trace Pb
- Greave[^1^](#_ENREF_1)^(Plate XXII)^ average: 10-11% Sn, trace Pb

A separate analysis of a different element gave similar results.[^106^](#_ENREF_106) Based on these analyses, there is no reason to suppose this composition does not apply to the whole suit of armour.

The thickness of metal recorded by the excavators is 1 mm,[^1^](#_ENREF_1) but no details have been reported about the weight of any of the pieces. Since the panoply is now mounted on an armature for display in the Nafplion Museum, it is no longer possible to obtain an accurate weight for the whole. An estimate of 18-20 kg seems reasonable, although on the basis of another replica made by Andrew Walpole, Molloy suggests a total weight of 15 kg.{Molloy, 2012 #147}

The Dendra panoply was completed with a boar’s tusk helmet of which over fifty plates were found.[^1^](#_ENREF_1) The two bronze cheek pieces found were, presumably, attached to this helmet. This type of helmet was common in the Mycenaean world from c 1650 – 1200 BC with a few as late as 1000 BC.[^107^](#_ENREF_107)^,^[^108^](#_ENREF_108) One of the few not found in a tomb context comes from below the sanctuary of Apollo at Thermon in Aetoloakarnania (Figure s8).[^109^](#_ENREF_109){Wardle, 2021 #175} In the Iliad such helmets were prized heirlooms, like the one given by Meriones to Odysseus (Il. 11.261-5).

**Figure s8.** Boars’ tusk plates from Thermon, Aetoloakarnania (Photo copyright: K.A.Wardle).


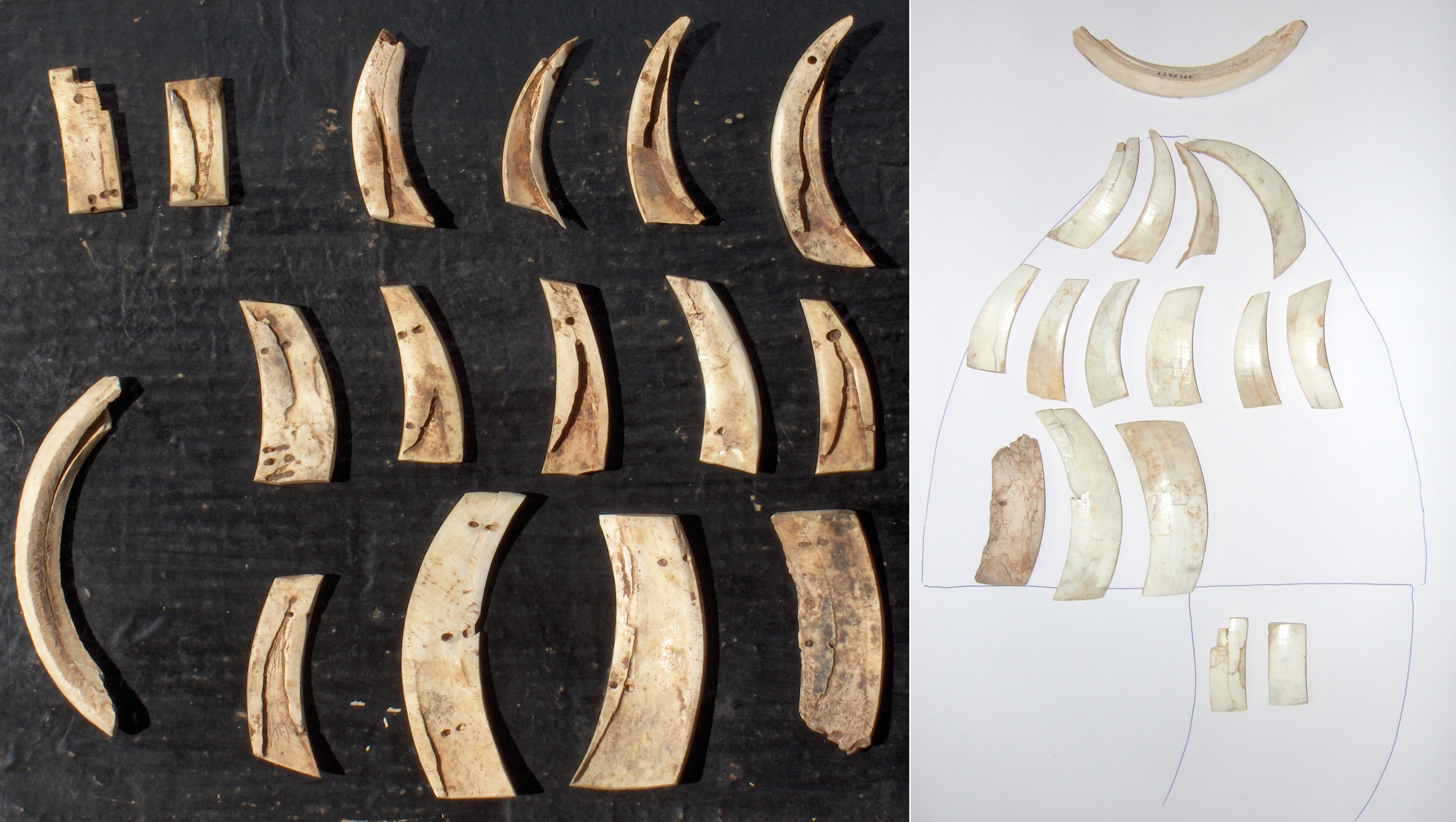


For the purposes of this study, we used a replica of the Dendra armour (Figure s9) made in Birmingham, UK.,[^110^](#_ENREF_110) in 1984 by staff (Keith Davis) and students (Cheryl Clarke and Stephen Thompson) of the Metal-working Department at the Bournville College of Art in Birmingham under the direction of Diana Wardle for the exhibition ‘Homer’s Heroes’ at the Birmingham City Museum and Art Gallery and later in Manchester, Durham, Belfast and other centres. The replica was made using gilding metal (95% copper, 5% zinc), which was the closest alloy to the original bronze available, but very slightly heavier (specific gravity of 8.8g/cc, compared to 8.72g/cc). Each of the plates of the armour followed exactly the dimensions, curvature and perforations of the original and was edged with leather strips and joined with leather thongs. The helmet plaques imitating the original boars’ tusk helmet were made from cast resin by H. Buglass of the former Department of Ancient History and Archaeology, University of Birmingham and the plaited leather inner cap by Ken Wardle,[^111^](#_ENREF_111) based on the description provided by Homer in the Iliad.{Homer, #165} The total (i.e., armour and helmet) weight of the replica was 23.32 kg [measured with a precision weight scale (Kern DE 150K2D, Kern & Sohn GmbH, Balingen, Germany)], as opposed to the original which, as noted above, is estimated to weigh about 18 kg (missing one handguard, parts of greaves, and after 3500 years of oxidisation). The weight of each piece of the replica is provided in Figure s9. Recent tests of the efficacy of such armour have revealed that it offered considerable protection against most blows.[^112^](#_ENREF_112) From this point forth, we refer to the replica as the Dendra armour.

| **Figure s9.** Photograph of the Dendra armour replica used for the purposes of this study (Photo copyright: S. B. Petmezas). | | | |
| --- | --- | --- | --- |
| 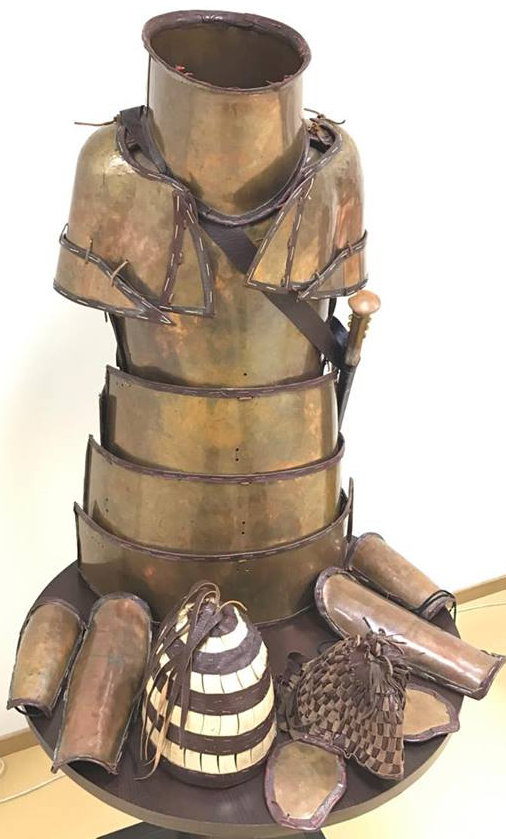 | **#** | **Description** | **Weight (kg)** |
|  | 1 | Helmet (outer) | 1.036 |
|  | 2 | Helmet (inner) with metal cheek guards | 0.644 |
|  | 3 | Collar | 1.634 |
|  | 4 | Corslet (front) | 6.526 |
|  | 5 | Corslet (rear) | 6.942 |
|  | 6 | Shoulder piece (Pauldron), right | 1.950 |
|  | 7 | Shoulder piece (Pauldron), left | 1.752 |
|  | 8 | Lower arm-guard (Vambrace), right | 0.338 |
|  | 9 | Lower arm-guard (Vambrace), left | 0.336 |
|  | 10 | Greave (right) | 0.596 |
|  | 11 | Greave (left) | 0.620 |
|  | 12 | Leather thongs | 0.550 |
|  | 13 | Shoulder pads | 0.400 |
|  |  | **Total** | **23.324** |

### Section 4.6. Weapons

The only weapon found *in situ* with the Dendra armour was a long knife.[^1^](#_ENREF_1) It would have been surprising if no other weapons had been present, but the tomb had been partly looted before full excavation.[^1^](#_ENREF_1) Two swords which appeared on the antiquities market shortly after the excavation and were sold at auction in Lucerne to the National Museum of Denmark in Copenhagen, are believed to be the ‘missing’ weapons. One is a ‘horned’ sword (Sandars’ type Ci)[^113^](#_ENREF_113)^(Plate 21)^ with an intricately worked gold-covered pommel. A fragment of similar gold work from an ivory pommel was recovered during the excavation. The second is a slightly later ‘cruciform’ sword (Sandars’ type Di)[^113^](#_ENREF_113)^(Plate 24),^ [^114^](#_ENREF_114) – the type on which the sword used in the experimental protocol is based (see below).

Scraps of bronze with traces of decayed leather may represent the remains of a shield but unfortunately not enough survives for certainty. Other finds include a range of pottery vessels which provide the dating evidence for the tomb and several bronze vessels of the kind regularly found in well-furnished tombs of the period in both mainland Greece and Crete.

For the purposes of this study, we created a replica Mycenaean cruciform sword (Sandars type Di)[^113^](#_ENREF_113) of the type reported to have been found in the Dendra tomb, which had blunt edges and point for safety reasons. Since bronze was not readily available, we used copper for the blade, chestnut wood for the handle and fish-based glue (Figure s10). The replica sword was 0.85 m long and weighed 1.2 kg (copper blade, wooden handle, copper ornamental nails and pins). Given that the specific gravity of 10% tin bronze is around 8.72g/cc and that of copper is 8.95g/cc, the original sword is slightly lighter than the replica used in the present study and, therefore, will have required less effort in Bronze Age combat contexts than in our experimental protocol.

| **Figure s10.** Development stages of the Mycenaean cruciform sword (Group Di) for this study. | | |
| --- | --- | --- |
| 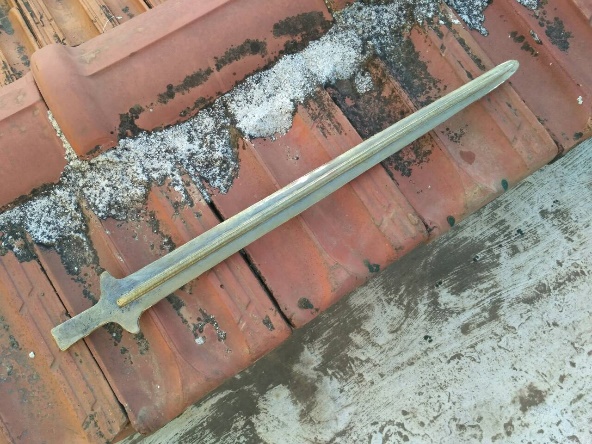 | 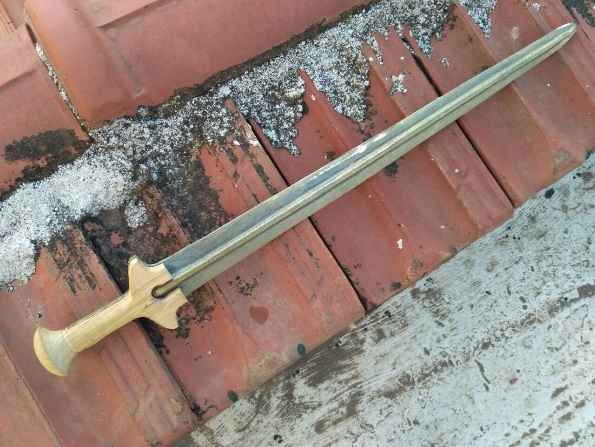 | 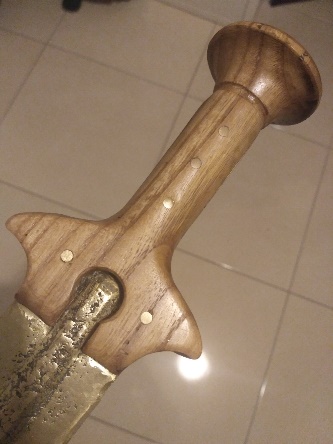 |

We used a 2.2 m ashwood spear with a copper head (blunt edges and point for safety reasons; 0.46 kg) similar to those described in the Iliad for the combat moves requiring spear throw or strike. Also, a medium-sized stone (23 x 15 cm; 1.3 kg weight) was used in the “foot warrior vs chariot” encounters (Table s4) and a composite recurve bow[^38^](#_ENREF_38) was used to shoot an arrow in a close-range in the “chariot vs warrior on ship” encounter (Table s4).

### Section 4.7. Anthropometric and physiological assessments

Weight was measured to the nearest 0.001 kg (as another measure of fluid loss) with a precision weight scale (Kern DE 150K2D, Kern & Sohn GmbH, Balingen, Germany). Height was measured using a stadiometer (Seca 213; Seca GmbH & Co. KG; Hamburg, Germany). Indirect calorimetry was used to evaluate the energy cost of the different activities using a portable gas analyser (Oxycon Mobile, CareFusion, San Diego, USA). Heart rate was assessed using wireless heart rate monitors (Polar Team2, Polar Electro Oy, Kempele, Finland). Core temperature was recorded using telemetric capsules (BodyCap, Caen, France). Skin temperature from four sites was measured using wireless thermistors (iButtons type DS1921H, Maxim/Dallas Semiconductor Corp., USA) and was expressed as mean skin temperature according to a well-established formula: 0.3(chest + arm) + 0.2(thigh + leg).[^115^](#_ENREF_115)

Force characteristics of the hits onto the target were evaluated using a force plate (Bertec FP4060-15-2000, Bertec Corp USA) sampled at 1000 Hz. The force plate was used to record the 3D reaction force generated during each hit as well as the centre of pressure position of the hit. The 3D force components from each hit, termed resultant force, was calculated and its peak value was inserted for analysis. Moreover, the absolute deviation of the centre of pressure of each hit from the centre of the target was calculated and processed further. For each hit, we evaluated the X,Y,Z forces generated by hits within each encounter, the resultant force, the coordinates of the centre of pressure of each hit as well as their average distance from the target centre.

Self-perceived thermal comfort (1 = comfortable; 5 = extremely uncomfortable), thermal sensation (-3 = cold; +3 = hot),[^116^](#_ENREF_116) and perceived exertion (6 = no exertion at all; 20 = maximal exertion)[^117^](#_ENREF_117) were assessed with standardised scales. Reaction time was assessed using computer software developed by our group (freely available at www.famelab.gr/research/downloads/). Participants were requested to place their finger on the screen of a tablet computer and to remove it as fast as possible after receiving a visual (i.e., screen turning from black to yellow) or acoustic (i.e., a loud beep) stimulus. Screen brightness and sound volume remained constant.

Urine samples were assessed for urine specific gravity using a handheld refractometer (ATAGO Ltd, Tokyo, Japan) and determined as either euhydrated (< 1.020) or dehydrated (≥ 1.020).[^118^](#_ENREF_118) Blood lactate concentration was assessed using a small blood sample from the tip of the finger and a portable lactic acid analyser (Lactate Scout, EKF-diagnostic GmbH, Barleben, Germany). A complete blood count assessment was performed using an automated haematology analyser (Mythic 18, Orphee SA, Geneva, Switzerland). ELISA was used to assess interleukin 10 (OriGene Technologies Inc., Rockville, MD, USA), as well as interleukin 6 and tumour necrosis factor alpha (Boster Biological Technology, Pleasanton, CA, USA).

### Section 4.8. Results

All participants successfully completed the 11-hour Late Bronze Age combat simulation protocol without serious issues other than a high level of fatigue, sore upper body due to the weight of the armour, and foot pain due to walking, running, riding a chariot and fighting barefoot. The “1-on-1” and “foot warrior vs chariot” encounters were the most demanding activities, while “manoeuvring on chariot” and the “chariot vs chariot” encounter were the least demanding activities (Figure 5, main text).

There was a minor increase in heart rate from the beginning until the end of the Late Bronze Age combat simulation protocol (r = 0.179, p < 0.001), paralleled by similar minor increases in core body temperature (r = 0.149, p < 0.001) and mean skin temperature (r = 0.119, p < 0.001) across time (Figure s11). Heart rate and mean skin temperature were higher in the “chariot vs warrior on ship,” “foot warrior vs chariot,” “chariot vs chariot,” “1-on-1,” “break,” “manoeuvring on foot,” and “manoeuvring on chariot” activities than at baseline (p < 0.05; Figure s11).

| **Figure s11.** Average heart rate (panels A-B), core body temperature (panels C-D), and mean skin temperature (panels E-F) during each Late Bronze Age combat simulation protocol stage (left-hand side graphs) and activity (right-hand side graphs). Shaded areas represent standard deviation. Brackets in activity graphs (B, D, E) indicate statistically significant differences compared to baseline (p<0.05). | |
| --- | --- |
| 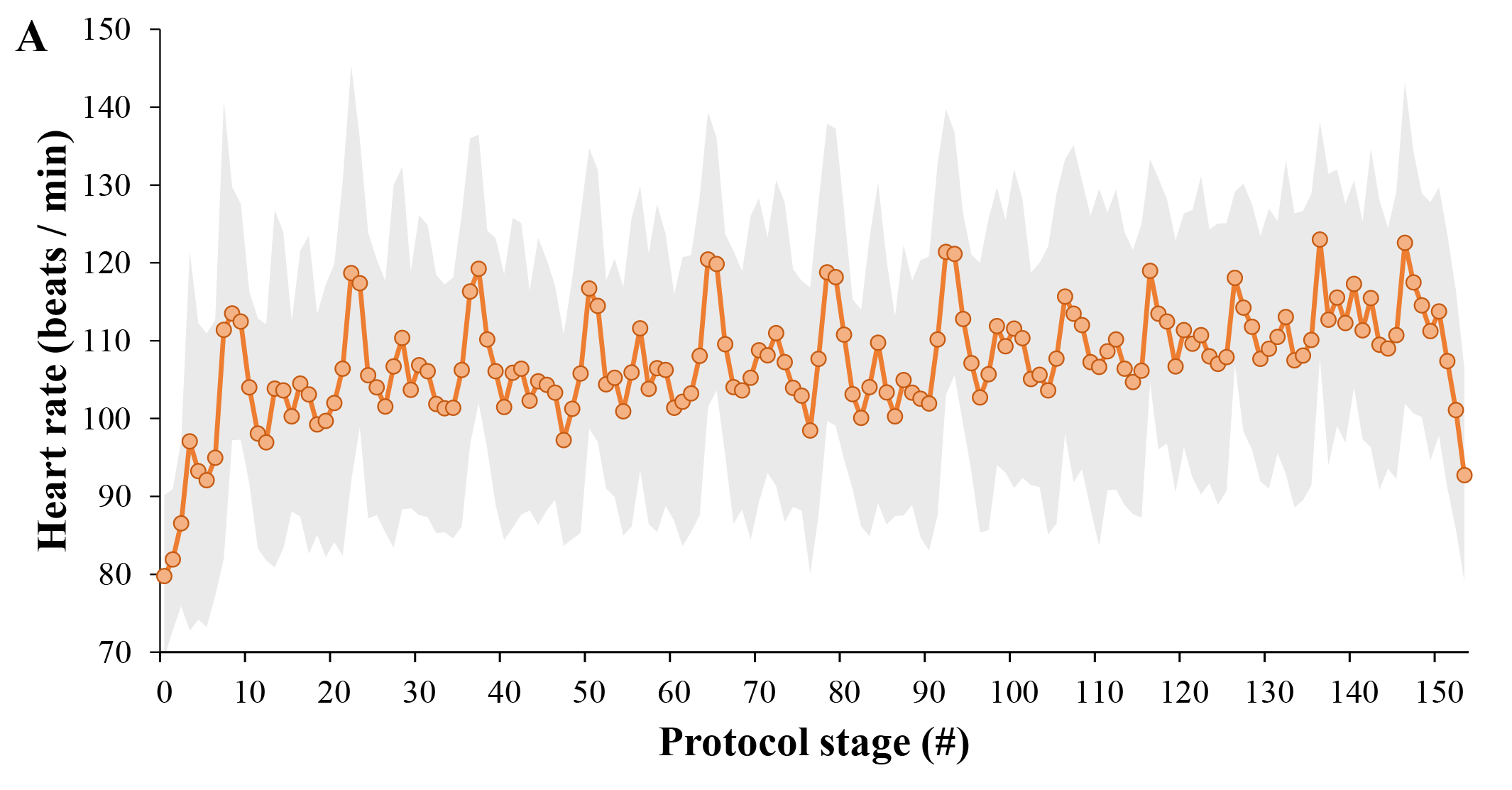 | 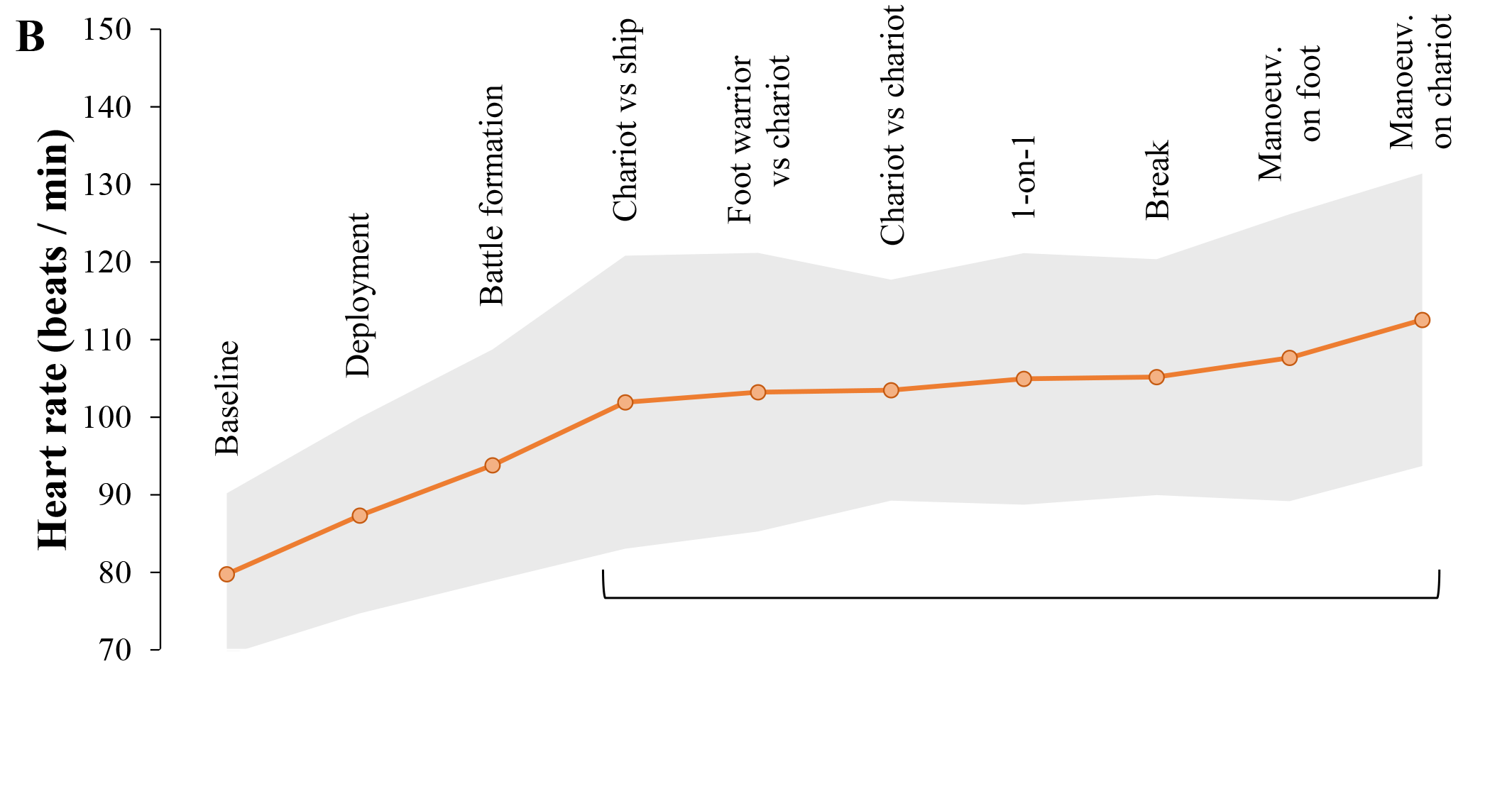 |
| 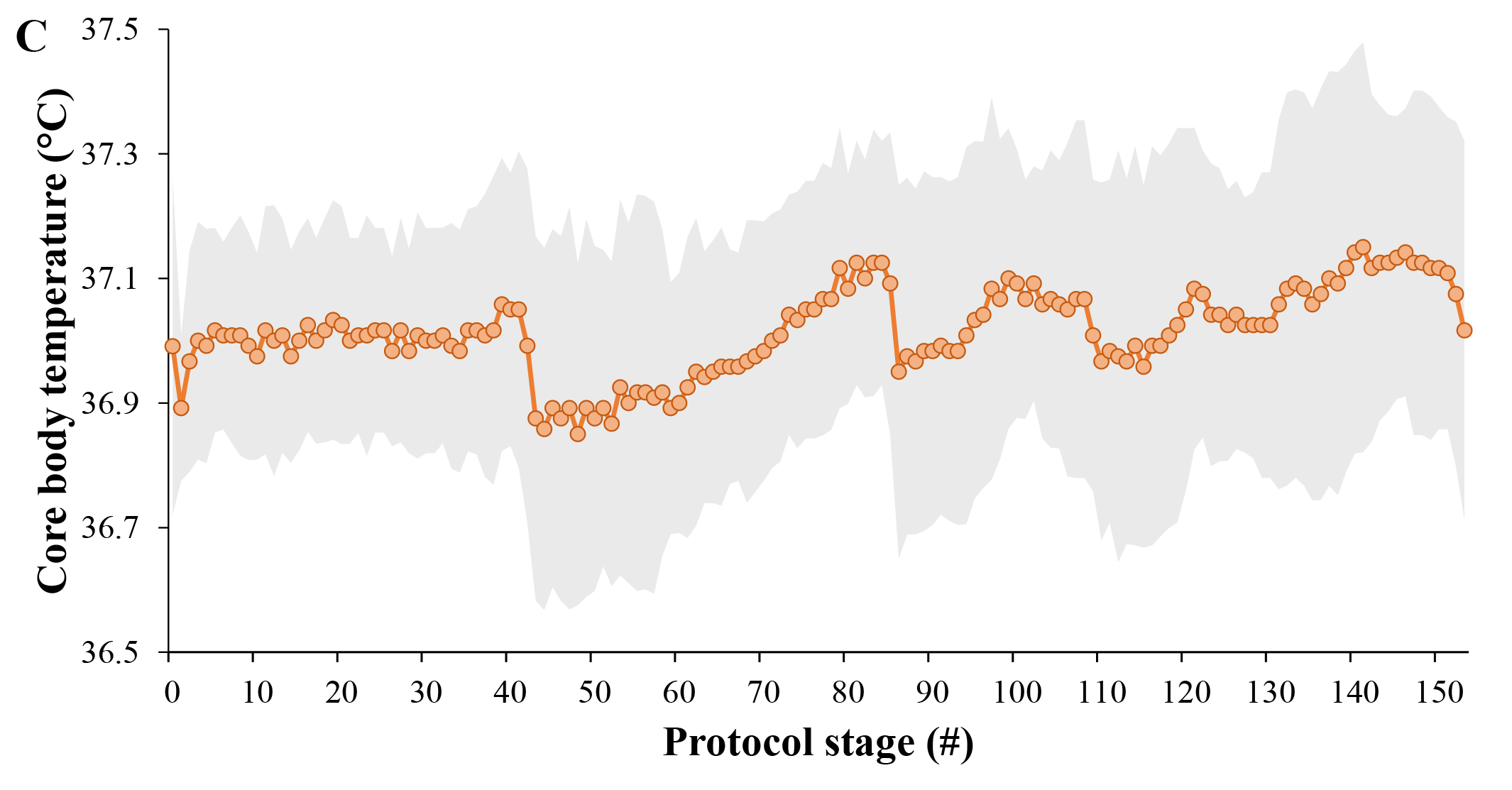 | 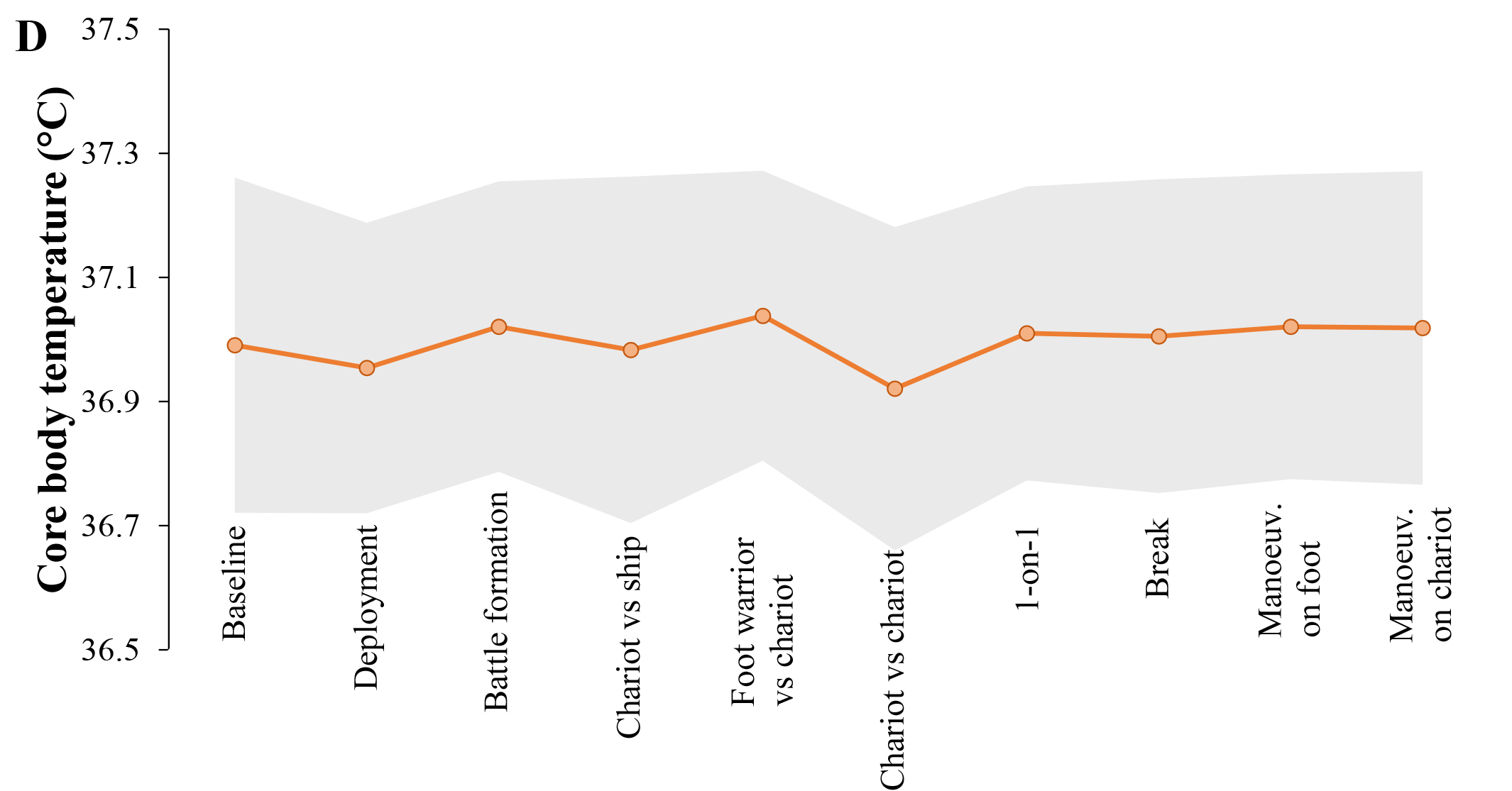 |
| 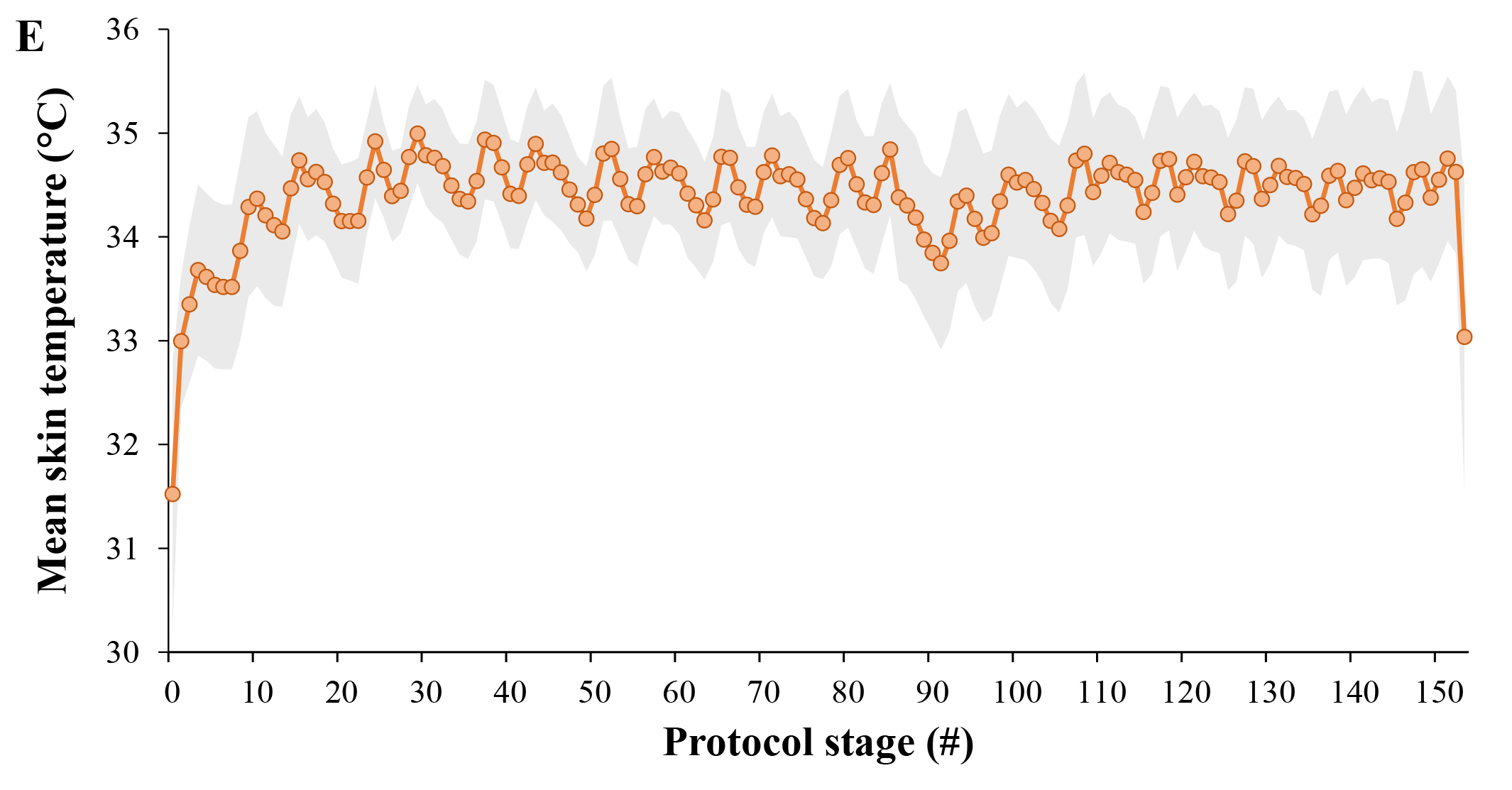 | 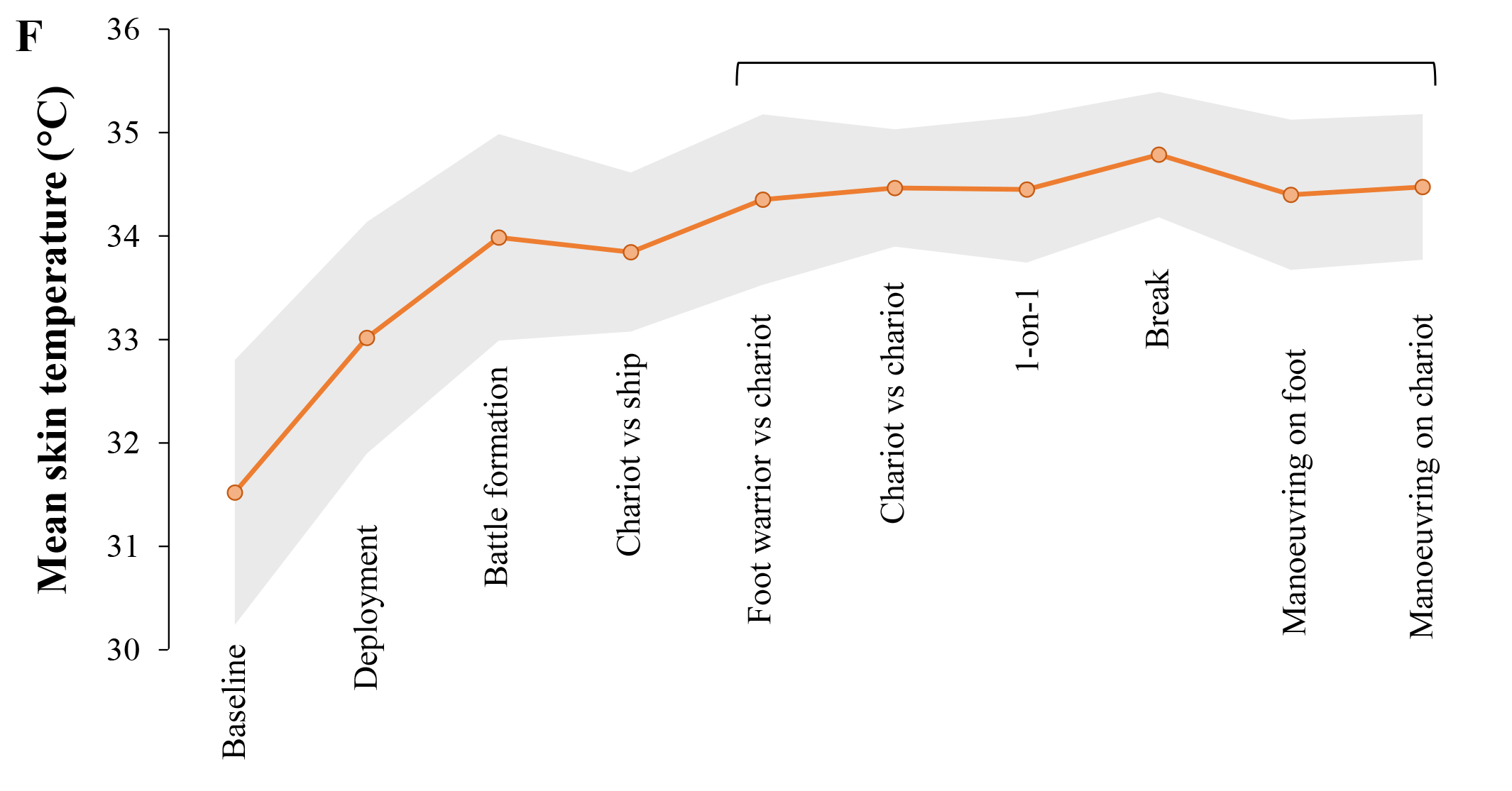 |

With regards to the force generated during combat hits, the average generated force per hit as well as the average deviation from the target centre per hit remained similar across encounter types (p > 0.05; Figure 6). Also, we found no significant reduction across time (which would indicate fatigue) in the force generated or the deviation from the target centre in any of the encounter types (p > 0.05). Figure s12 illustrates the relevant results for 1-on-1 which was the most frequent encounter type. This is the first time that this type of setting has been used to collect ground reaction data and therefore a direct comparison is not possible. A recent study of hammer hits to the head by a large group of men and women[^119^](#_ENREF_119) showed single hammer stroke impact forces as high as 14 kN but the setting was very different. We consider the forces generated in our study relatively high, meaning that the hits performed by our participants could potentially result in serious injury depending on the body part affected.

| **Figure s12.** Characteristics of the force generated and the deviation from the target centre during hits across time. Bars represent standard deviation. | |
| --- | --- |
| 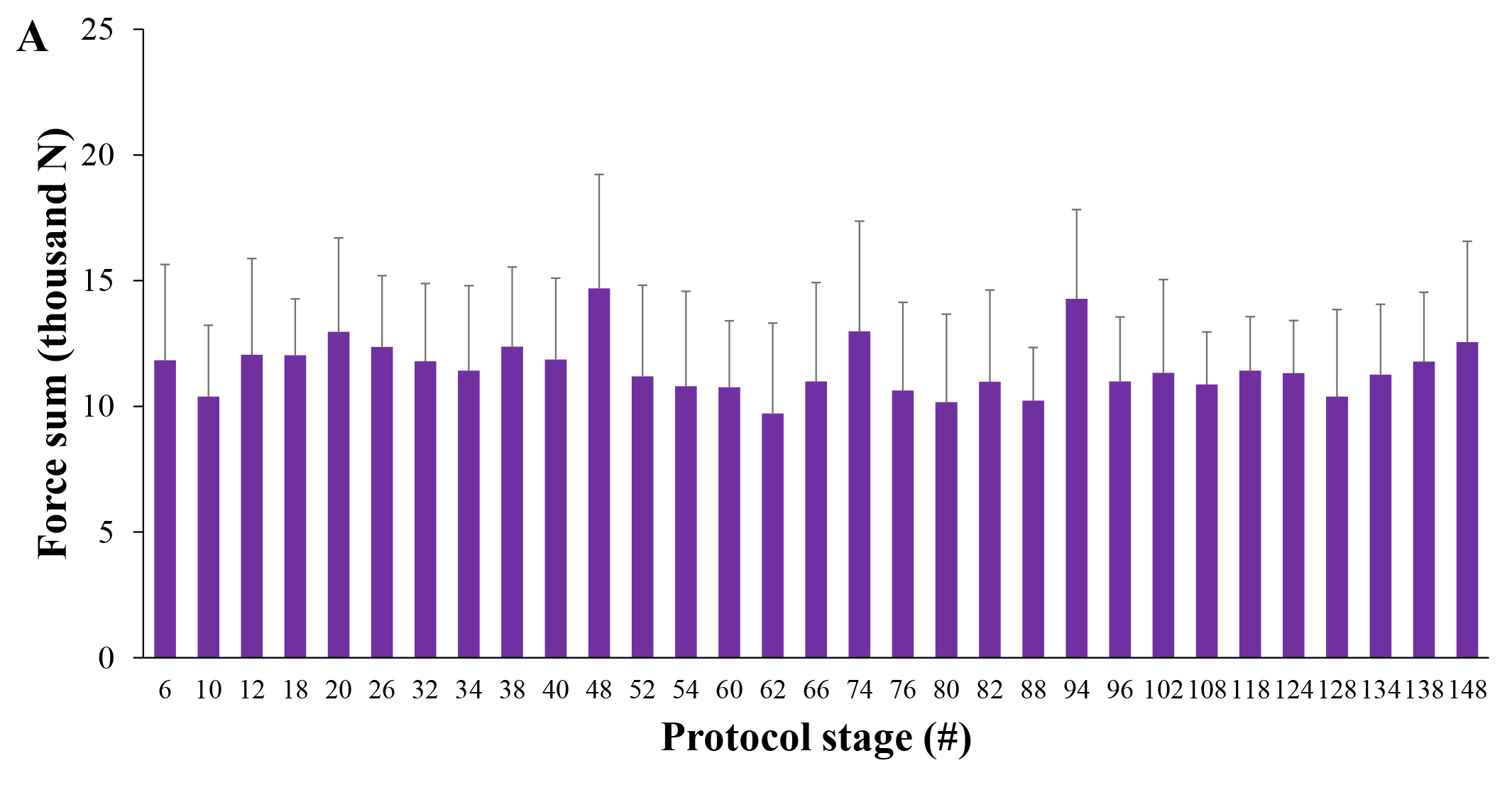 | 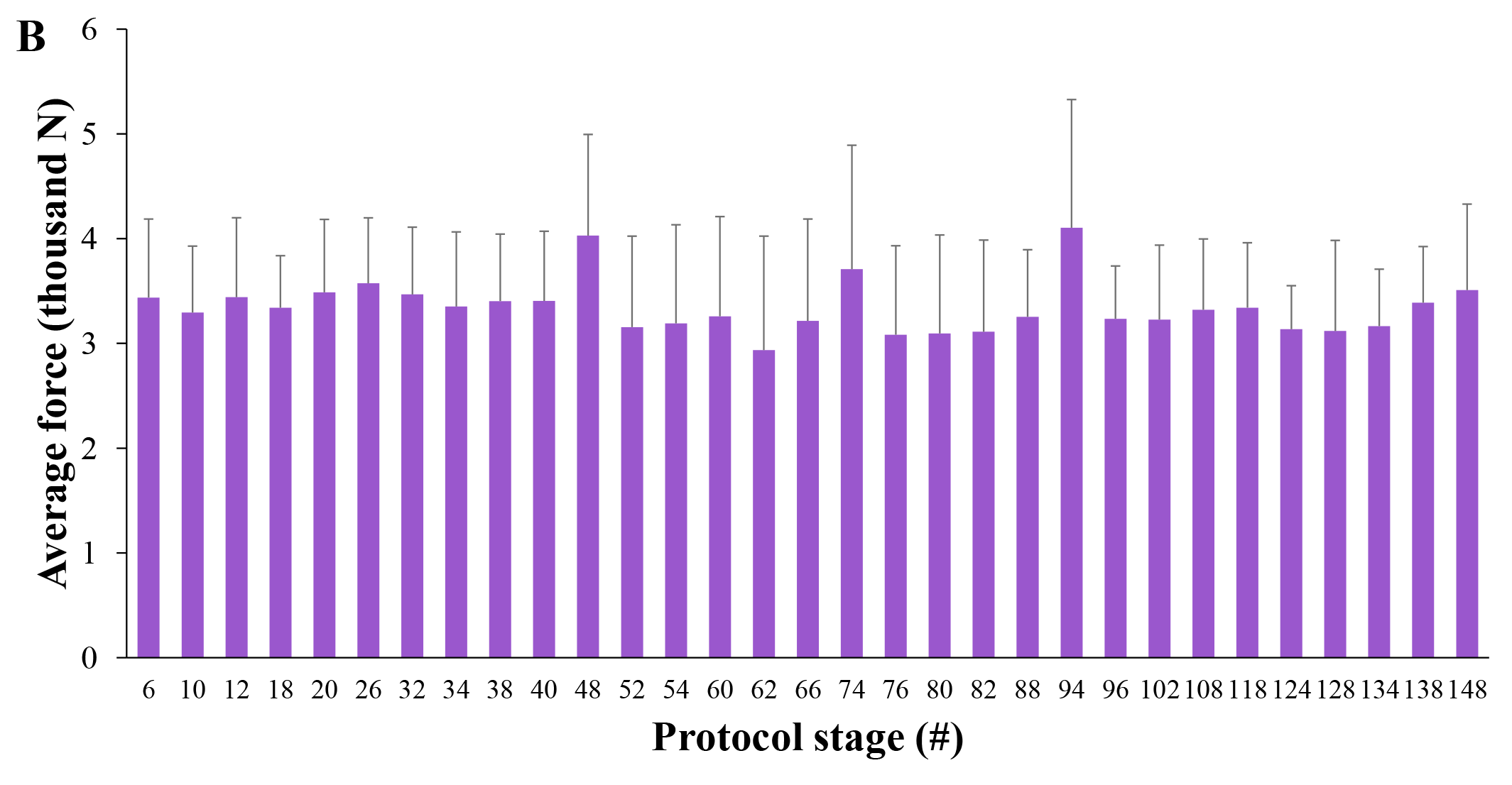 |
| 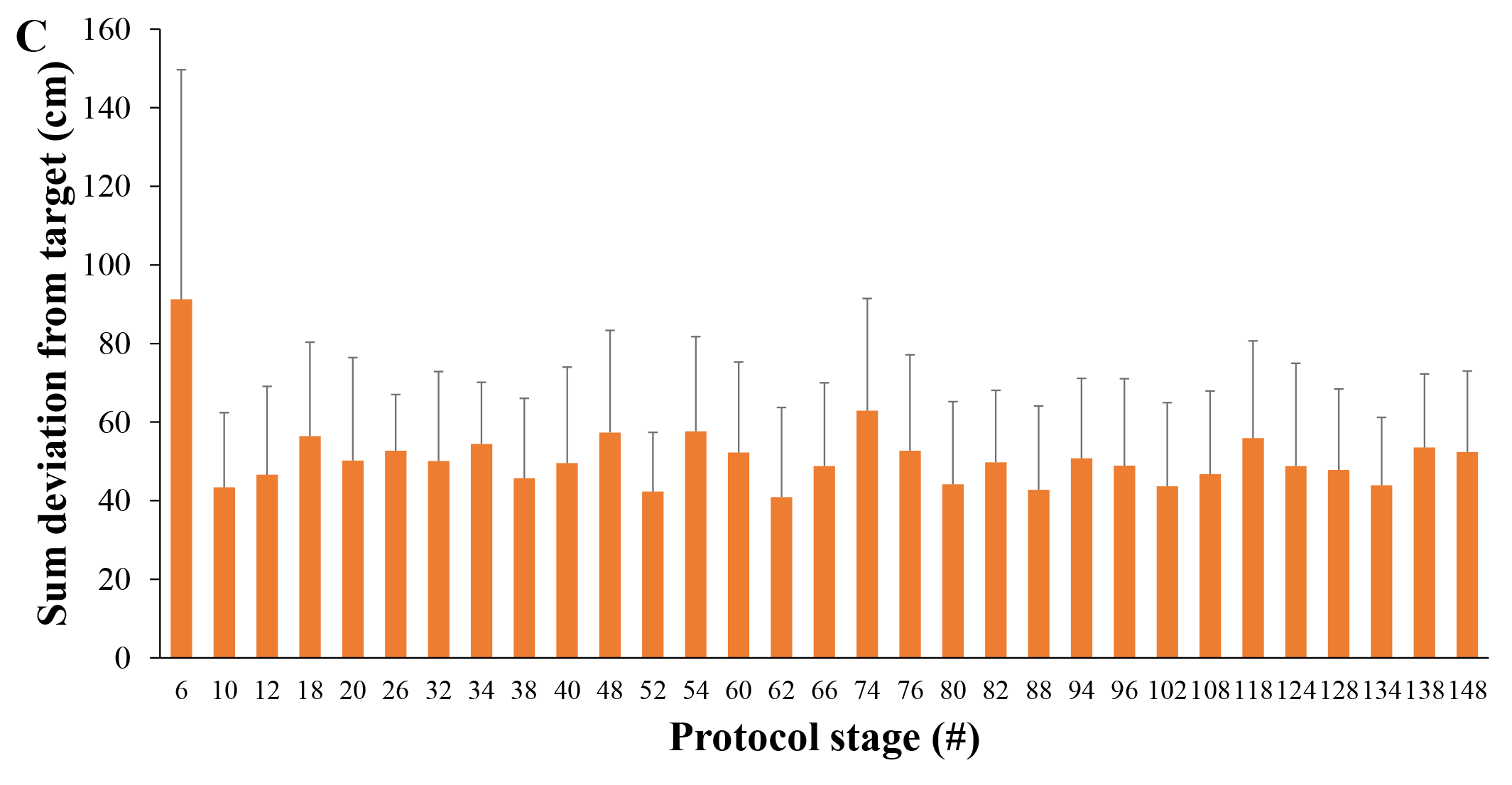 | 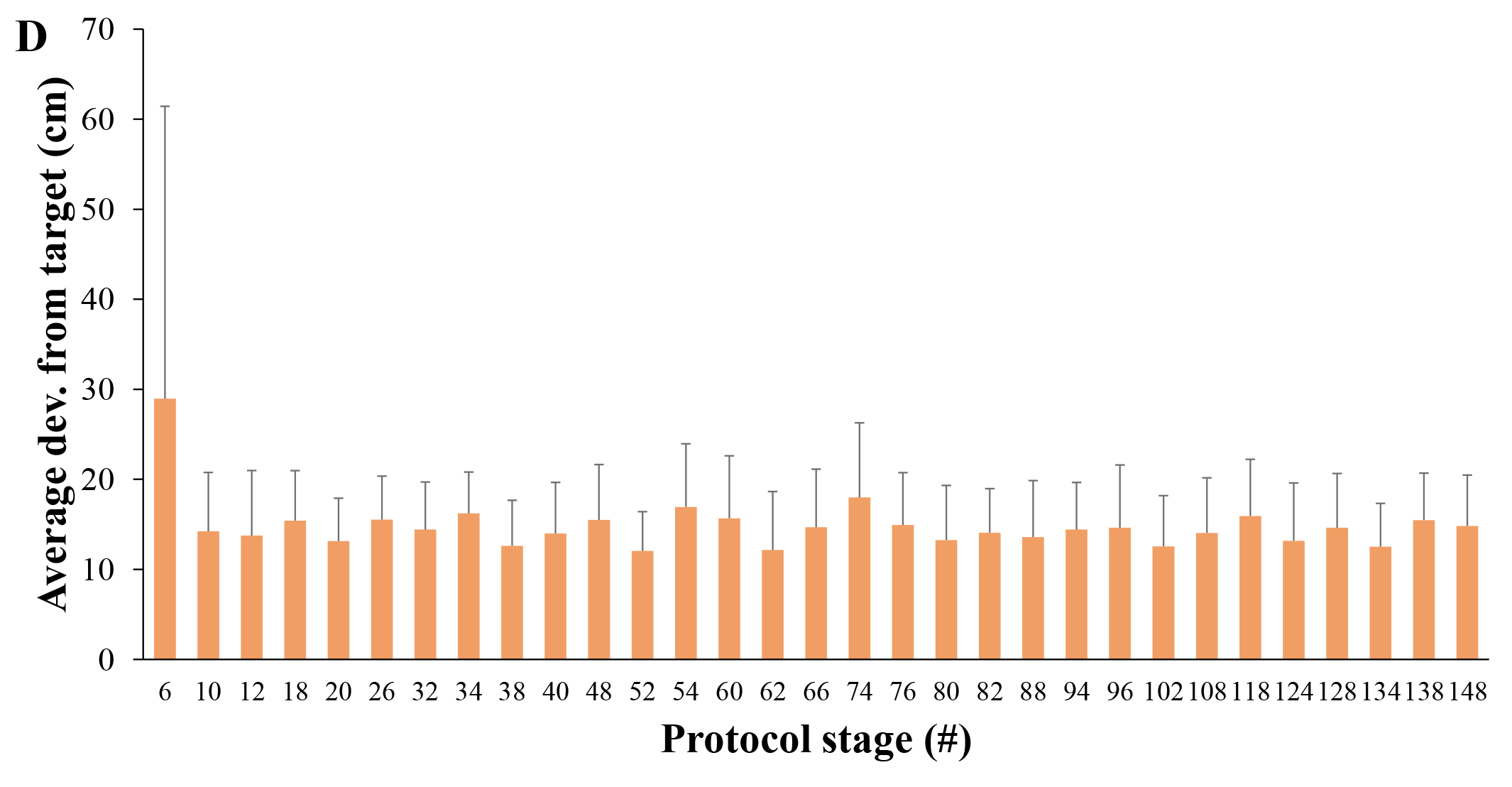 |

The self-perceived fatigue of the participants increased across time (r = 0.603, p < 0.001), and they reported significantly increased fatigue levels (p < 0.05) from the Late Bronze Age combat simulation protocol stage 56 onwards (i.e., after 4 hours of combat; Figure s13). The participants’ thermal sensation (r = 0.188, p = 0.05) and thermal comfort (r = 0.401, p < 0.001) increased across time (Figure s13). Thermal sensation was higher than baseline during stages 110 to 150 of the combat simulation protocol (p < 0.05), while thermal comfort was higher than baseline almost throughout (p<0.05). There was a decline in blood glucose levels across time (r = -0.234, p = 0.017), from an average of 105.5 ± 14.0 mg / dL at baseline to 89.4 ± 9.0 mg / dL at the end of the 11-hour Late Bronze Age combat simulation protocol (p < 0.05; Figure s13). Importantly, the participants demonstrated a reactive leucocytosis at the end of the combat simulation protocol (Table s6), indicating the increased level of physiological stress endured. Leucocytosis at the level seen in our post-combat measurement is a typical physiological reaction of healthy bone marrow to extreme stress and exercise, leading to catecholamine-induced demargination of white blood cells and increased release from the bone marrow storage pool.[^120^](#_ENREF_120)

| **Figure s13.** Results across time for perceived exertion, thermal sensation, thermal comfort, as well as blood glucose. Bars represent standard deviation. Note: a = different from stage 0 (p < 0.05). | | | | | | | | |  |
| --- | --- | --- | --- | --- | --- | --- | --- | --- | --- |
| 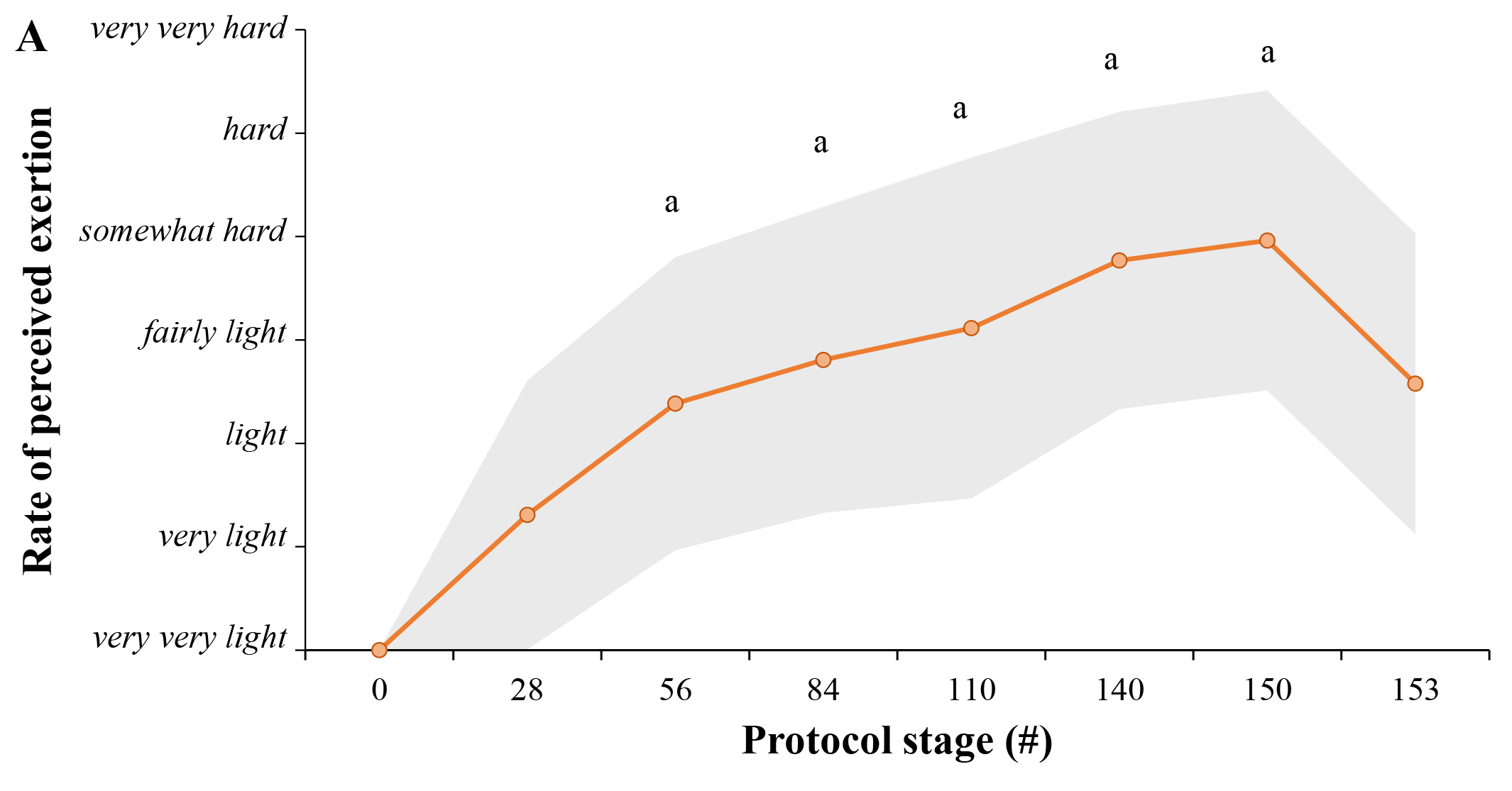 | | | | | 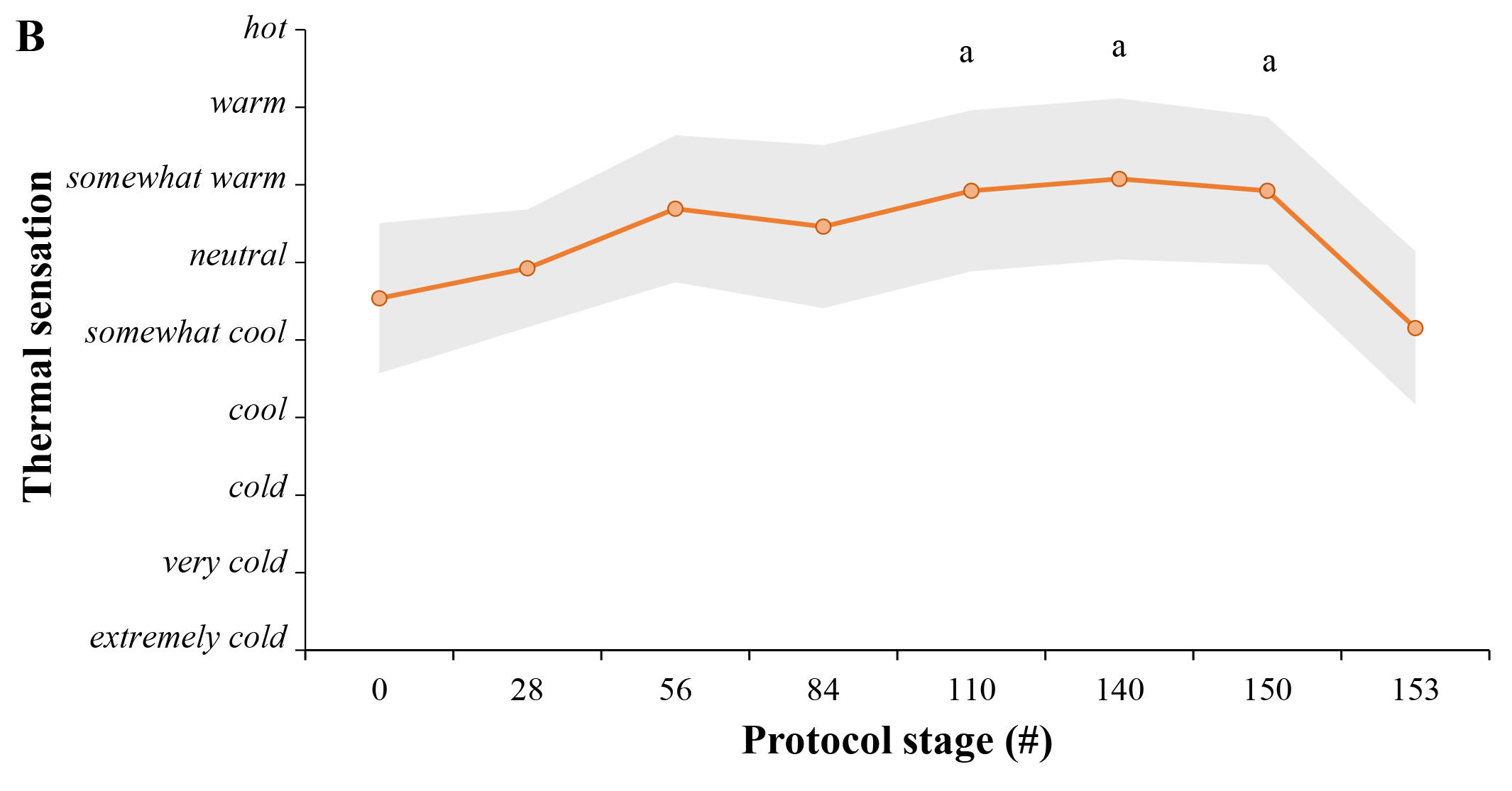 | | | |  |
| 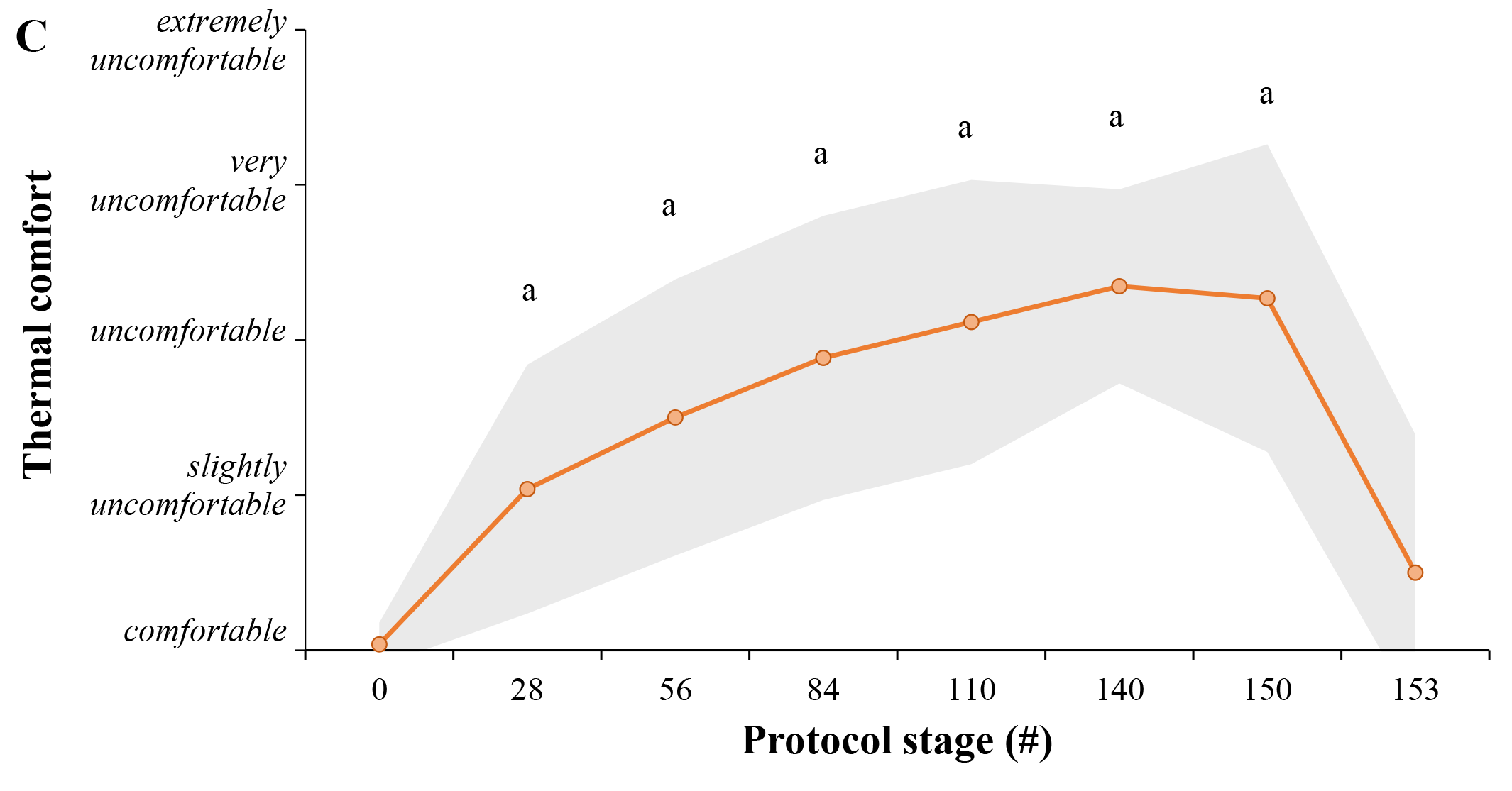 | | | | | 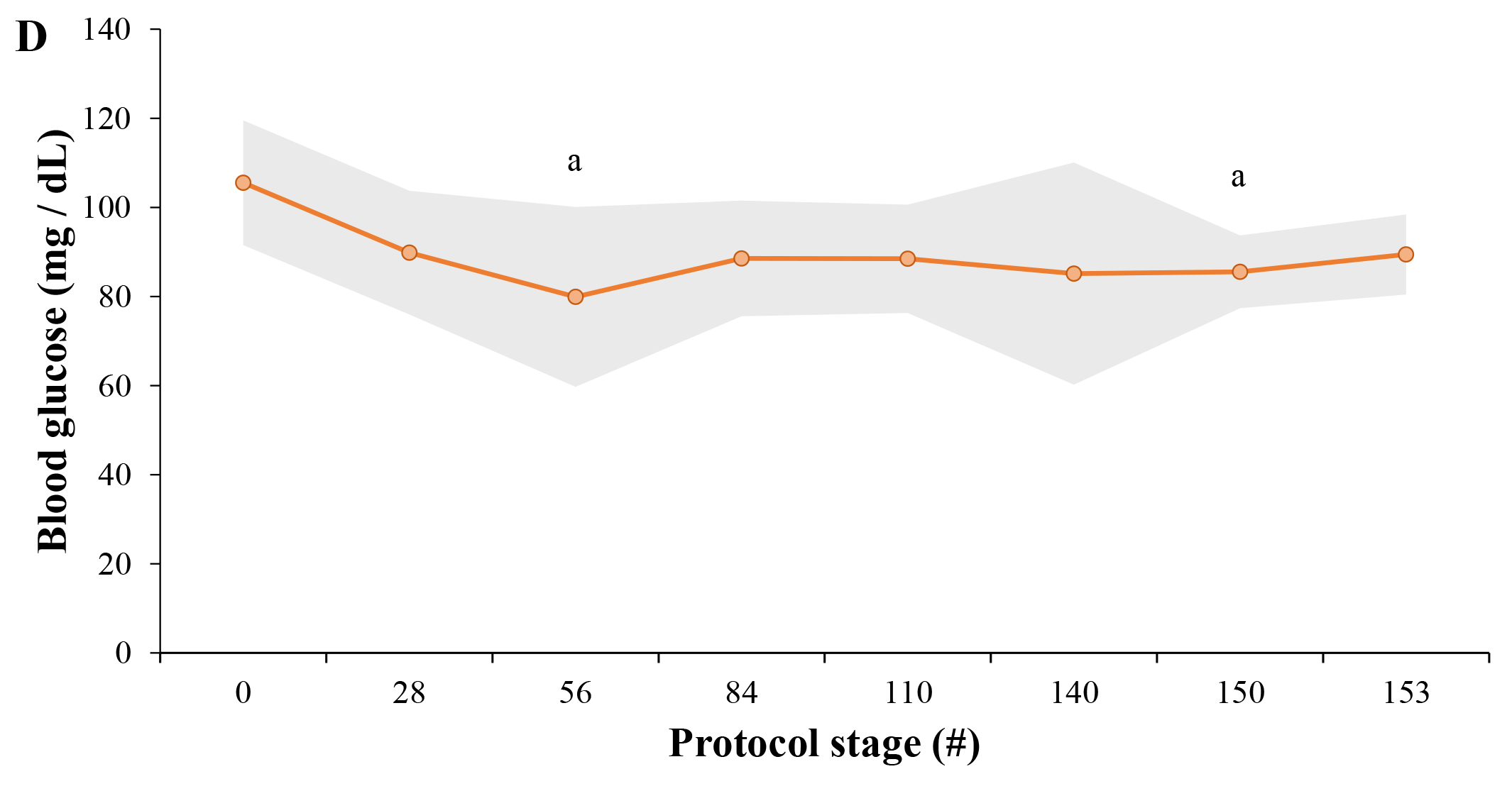 | | | |  |
| **Table s6.** Results (mean ± standard deviation) for the complete blood count assessments performed before and after the Late Bronze Age combat simulation protocol. | | | | | | | | | |
| **Time** | **WBC** | **LYM** | | **MON** | | | **GRA** | | |
|  |  | **#** | **%** | **#** | | **%** | **#** | **%** | |
| Pre | 6.3±0.7 | 2.4±0.4 | 38.3±4.9 | 0.3±0.1 | | 5.3±0.8 | 3.6±0.5 | 56.4±5.1 | |
| Post | 12.1±1.2 ^a^ | 2.3±0.9 | 18.9±5.9 ^a^ | 0.5±0.1 ^a^ | | 4.1±1.2 ^a^ | 9.3±1.3 ^a^ | 77.1±5.7 ^a^ | |
|  |  |  |  |  | |  |  |  | |
|  | **RBC** | **HGB** | **HCT** | **MCV** | | **MCH** | **MCHC** | **PLT** | |
| Pre | 5.4±0.6 | 15.3±1.1 | 45.6±1.9 | 85.8±8.6 | | 28.7±3.4 | 33.4±1.2 | 248.8±29.9 | |
| Post | 5.4±0.6 | 14.9±1.4 ^a^ | 44.0±3.1 ^a^ | 83.1±8.6 ^a^ | | 28.1±3.5 ^a^ | 33.8±1.6 | 225.9±56.9 | |
| Note: ^a^ = significantly different from “pre” at p < 0.05.  Key: WBC = white blood cells; LYM = lymphocytes; MON = monocyte count; GRA = granulocytes; RBC = red blood cells; HGB = haemoglobin; HCT = haematocrit; MCV = mean corpuscular volume; MCH = mean corpuscular haemoglobin; MCHC = mean corpuscular hemoglobin concentration; PLT = platelets; # = count; % = percent of WBC. | | | | | | | | | |

We found no statistically significant changes in hand grip strength, reaction time to visual or sound stimuli, urine specific gravity, blood lactate (which ranged from 4 to 9 mmol / L for most of the Late Bronze Age combat simulation protocol), interleukin 6, interleukin 10, as well as tumour necrosis factor alpha (p > 0.05).

### Section 4.9. Artistic photo shoot

Following the end of data collection, a photo shoot was organised for the purpose of creating a documentary visual account of the measurements and obtain images that can used for print/digital advertising and popularization of the experimental study and its findings. In this case, the sensors and measuring equipment were removed as the primary focus was on the warrior, the armour, the weapons, the body postures and the combat moves. Selected photos are illustrated in Figures 7 and s14-15.

| **Figure s14.** Selection of artistic photos taken after the end of the experimental study. | |
| --- | --- |
| 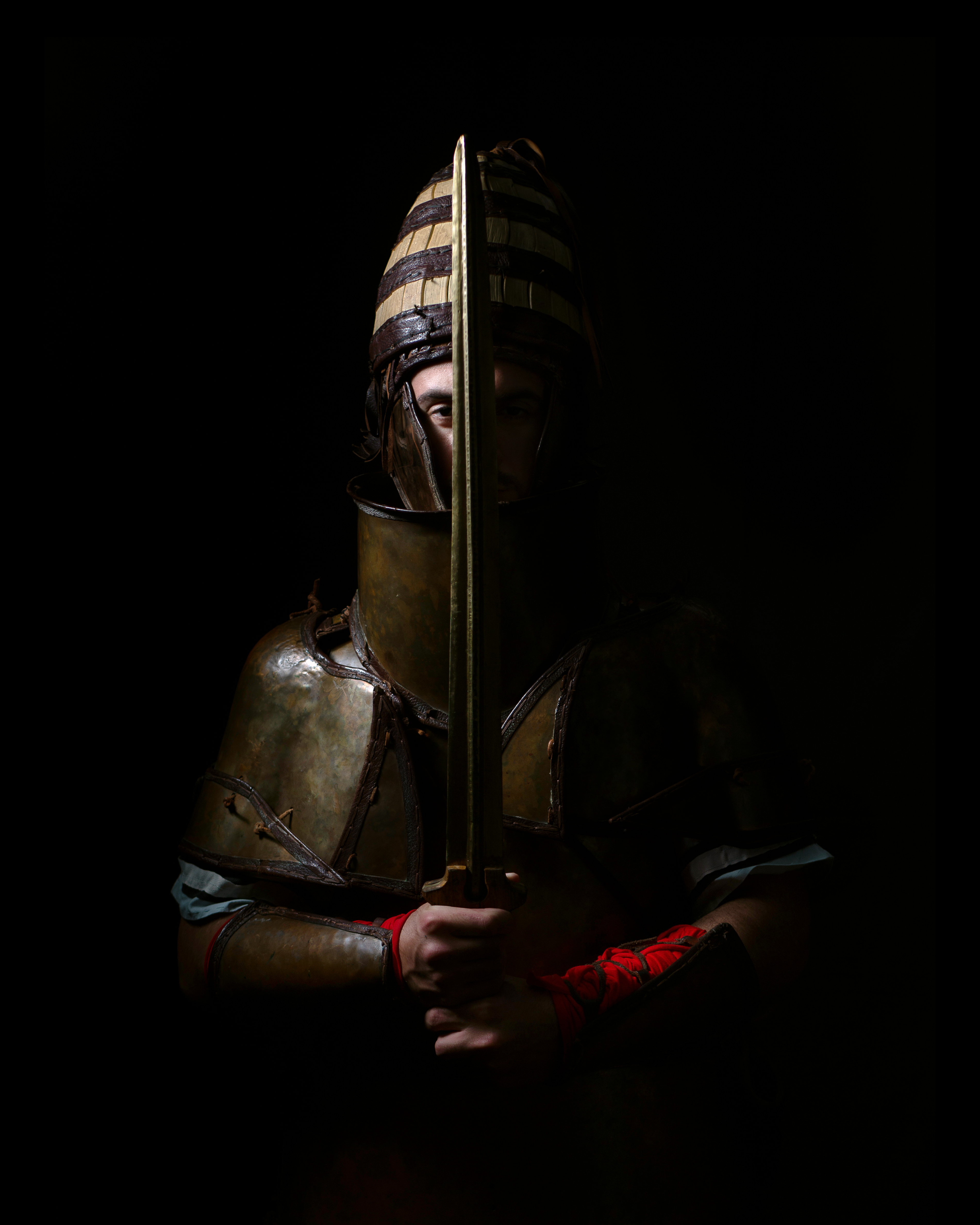 | 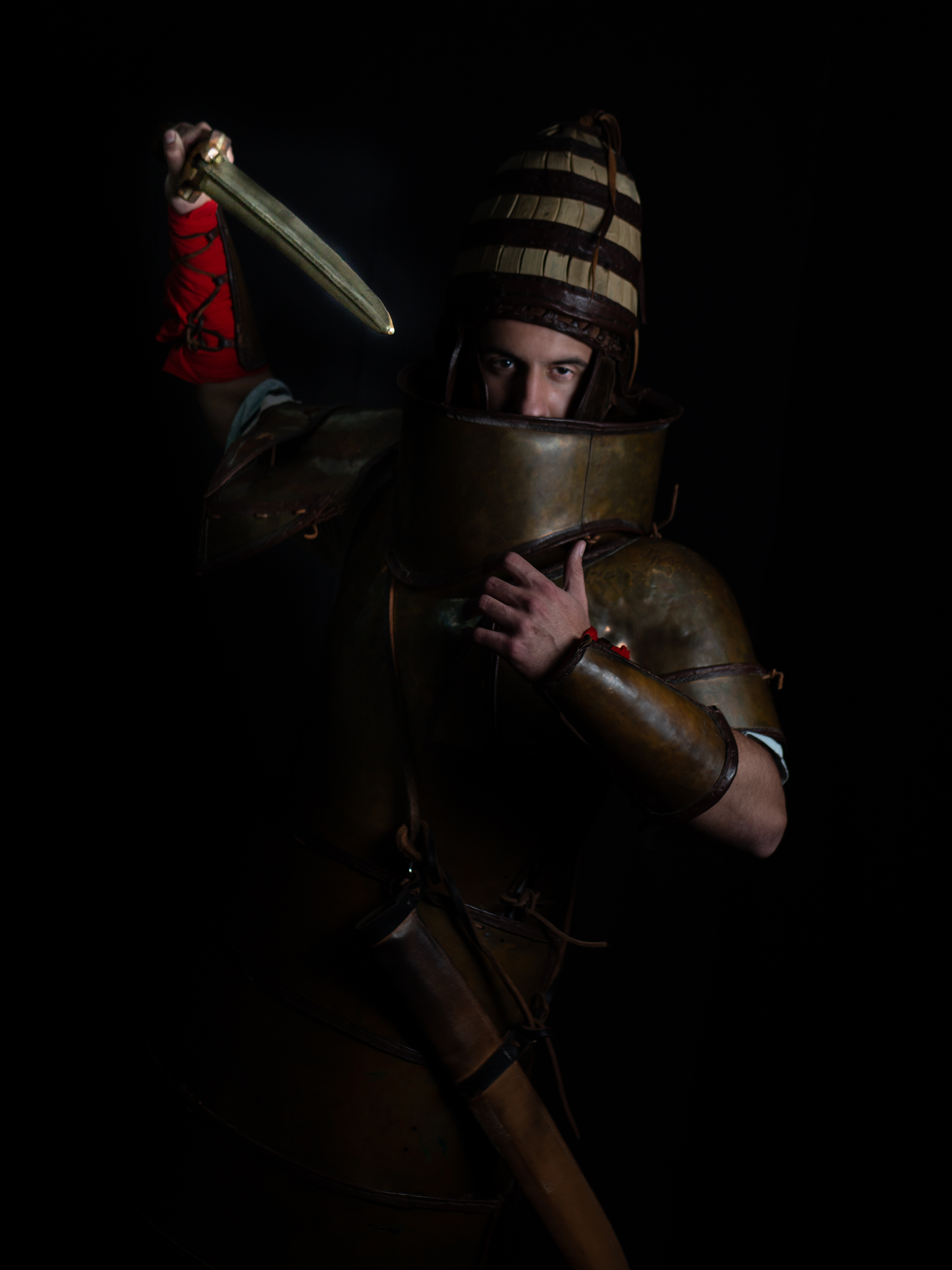 |
| 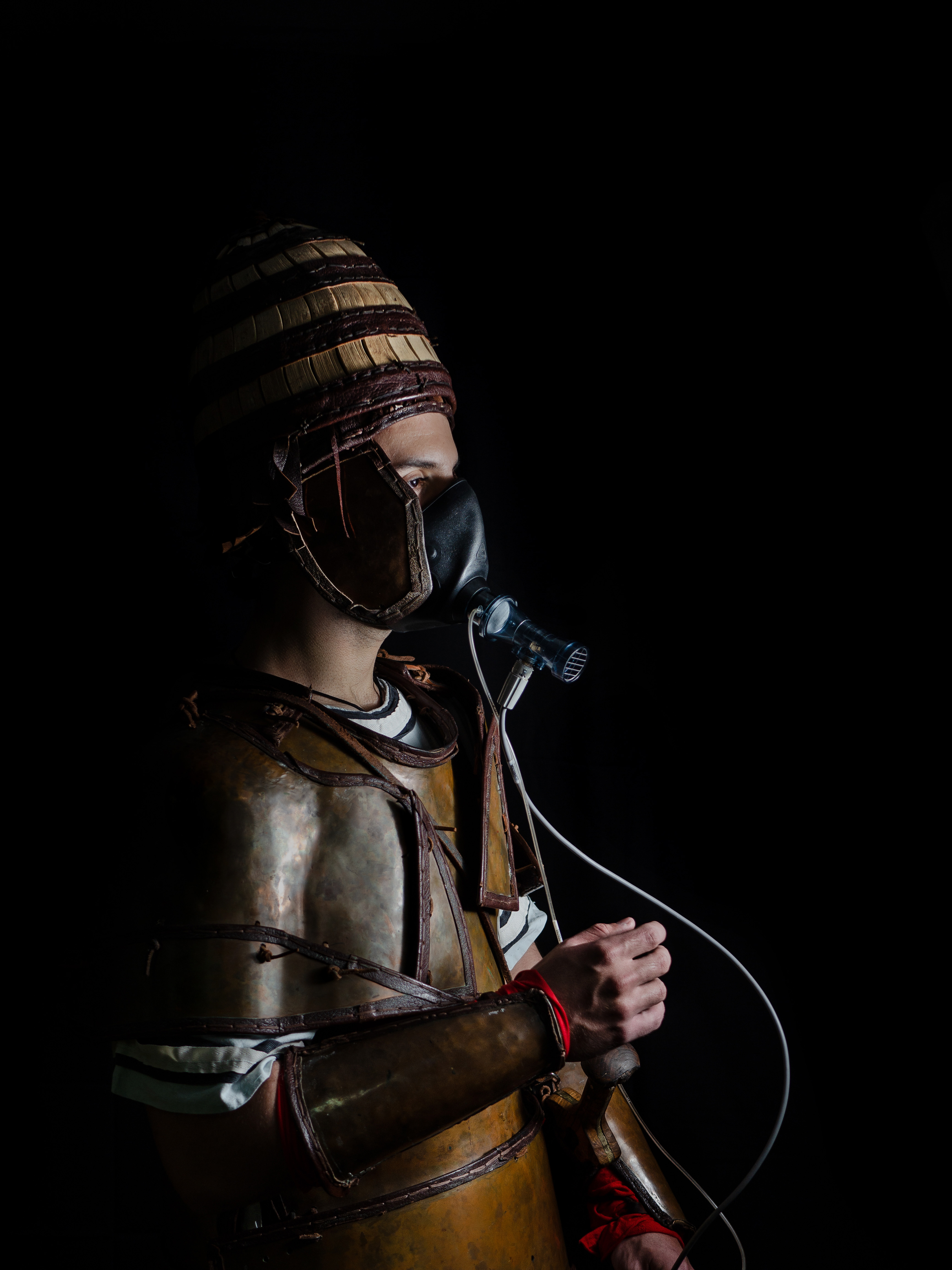 | 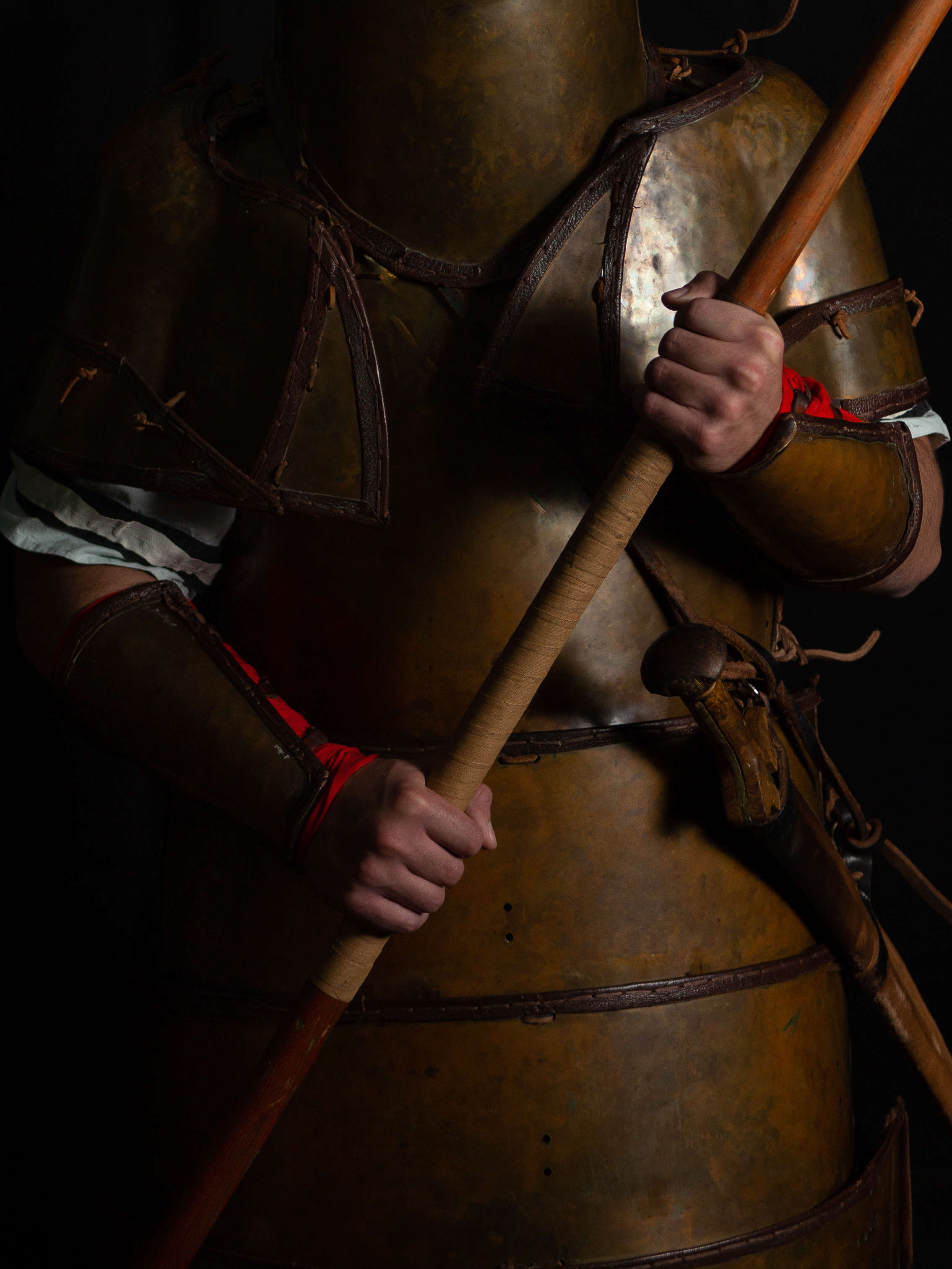 |

| **Figure s15.** Selection of artistic photos taken after the end of the experimental study. | |
| --- | --- |
| 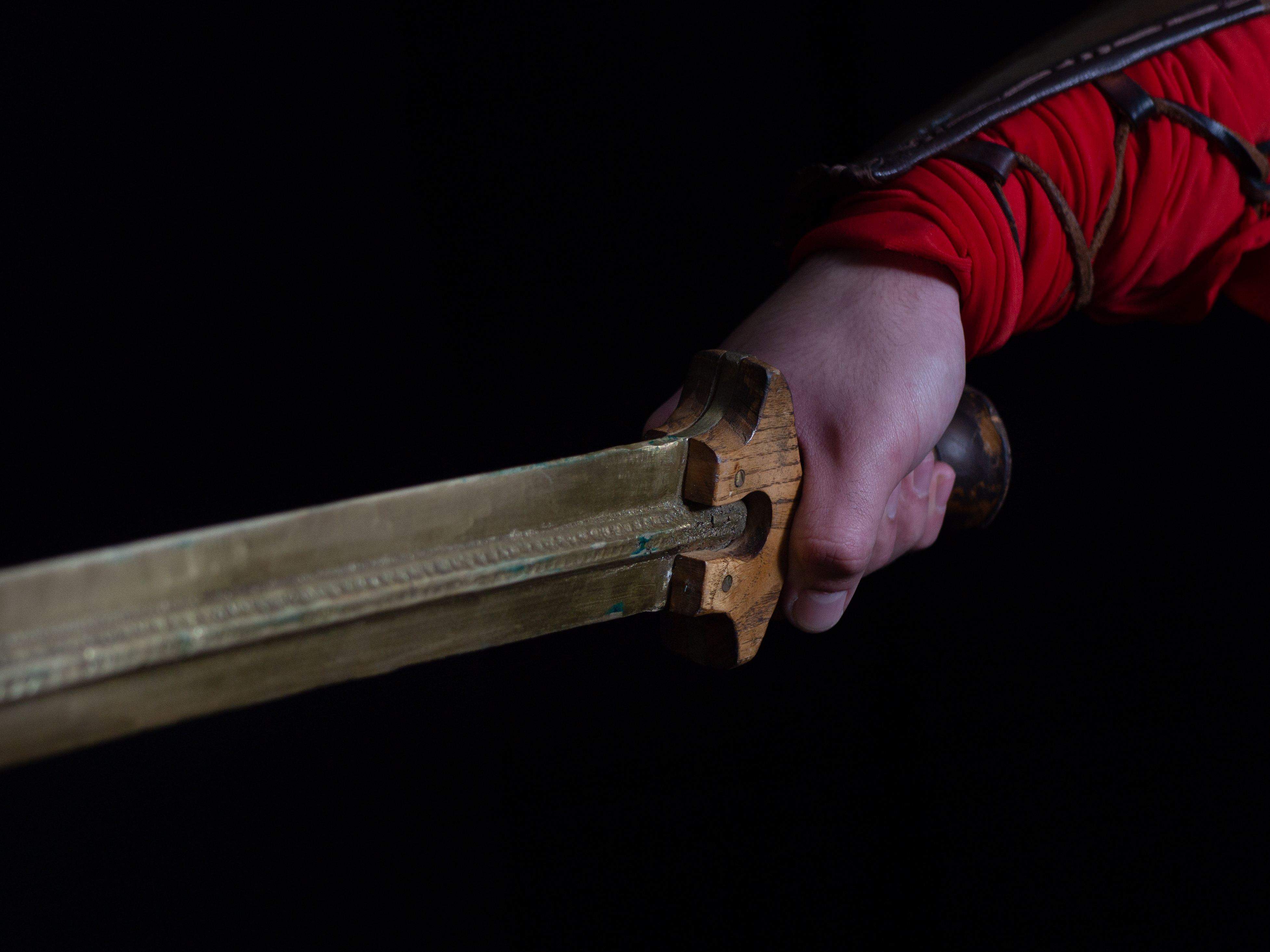 | 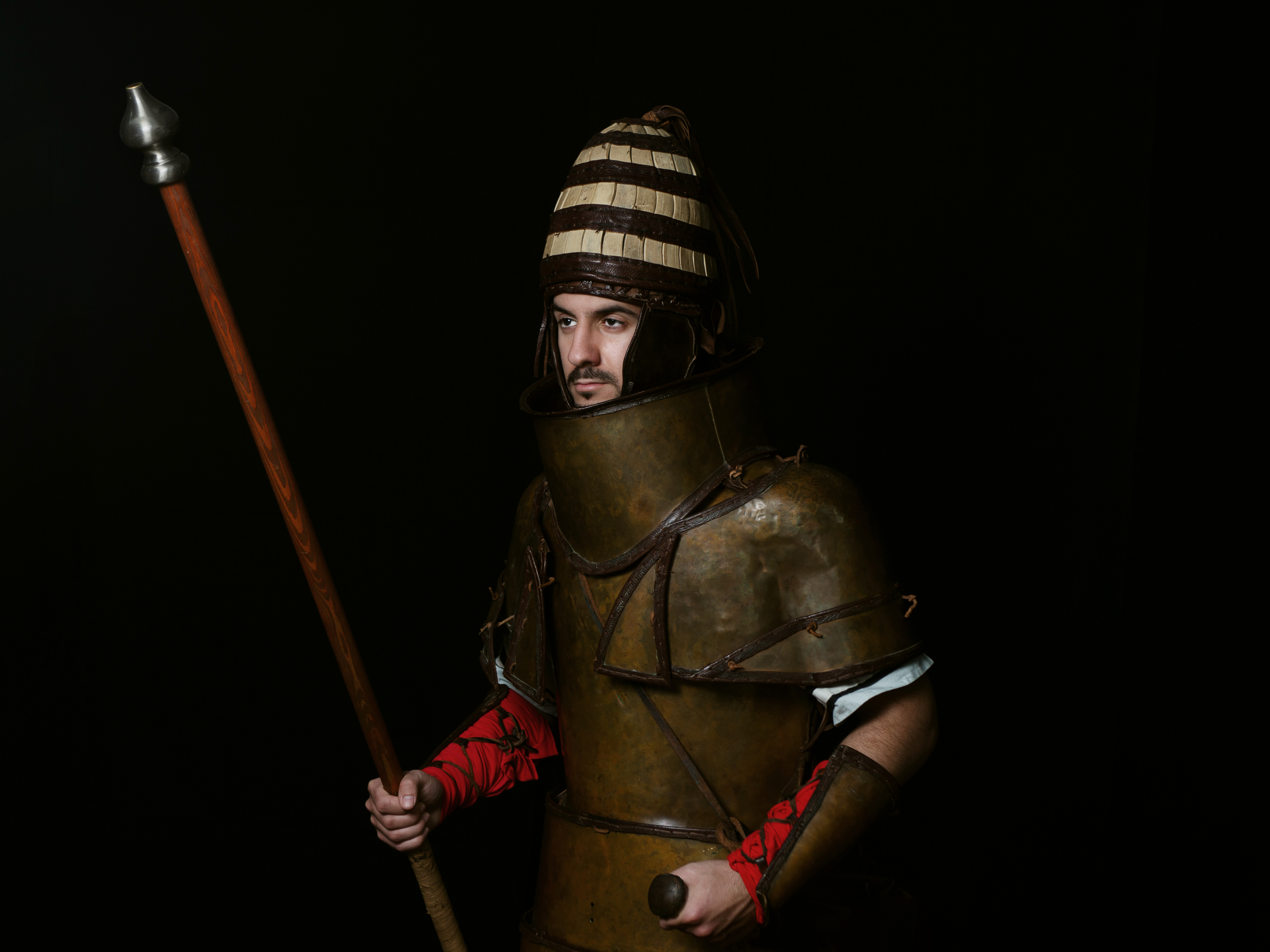 |
| 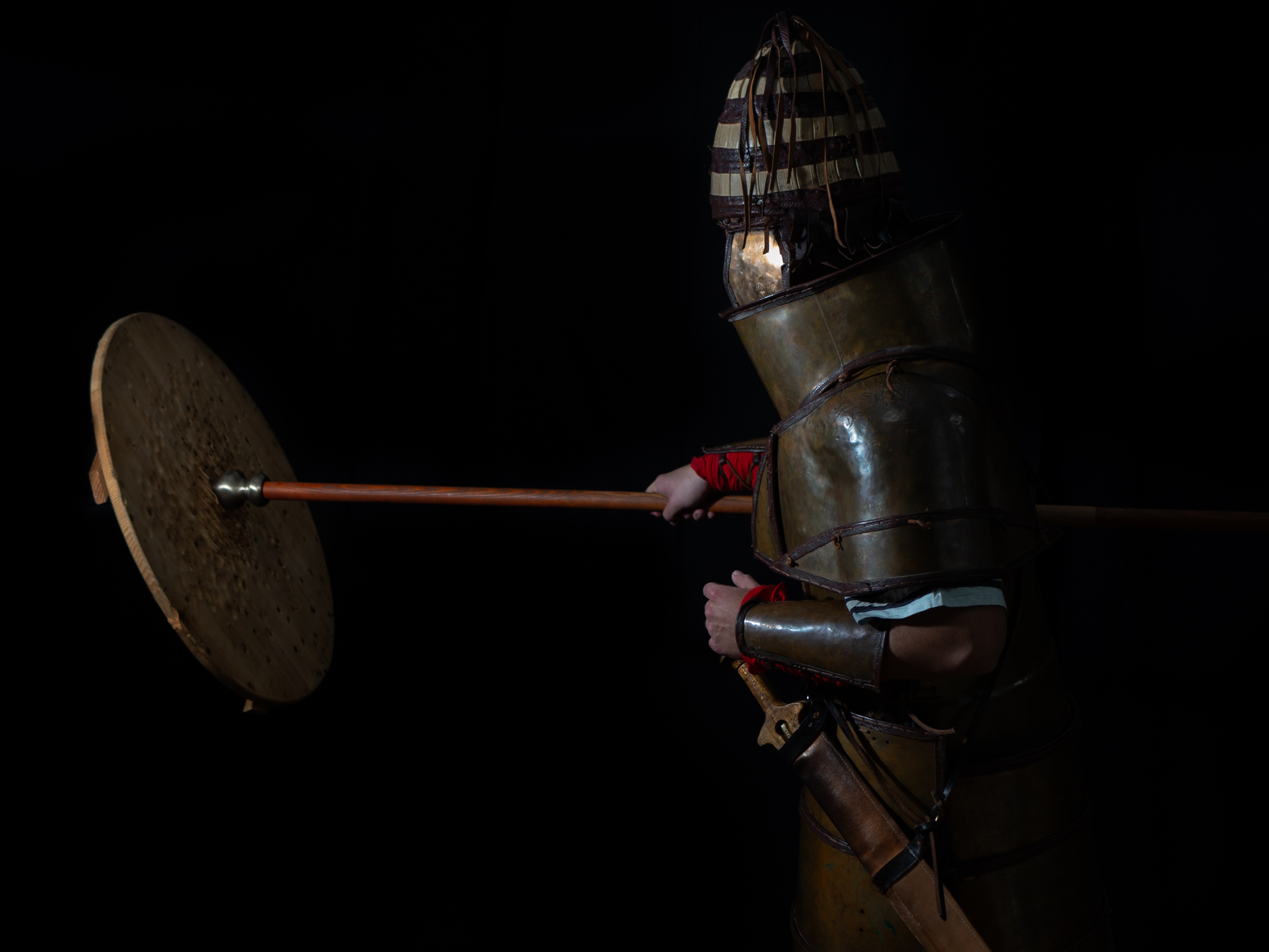 | 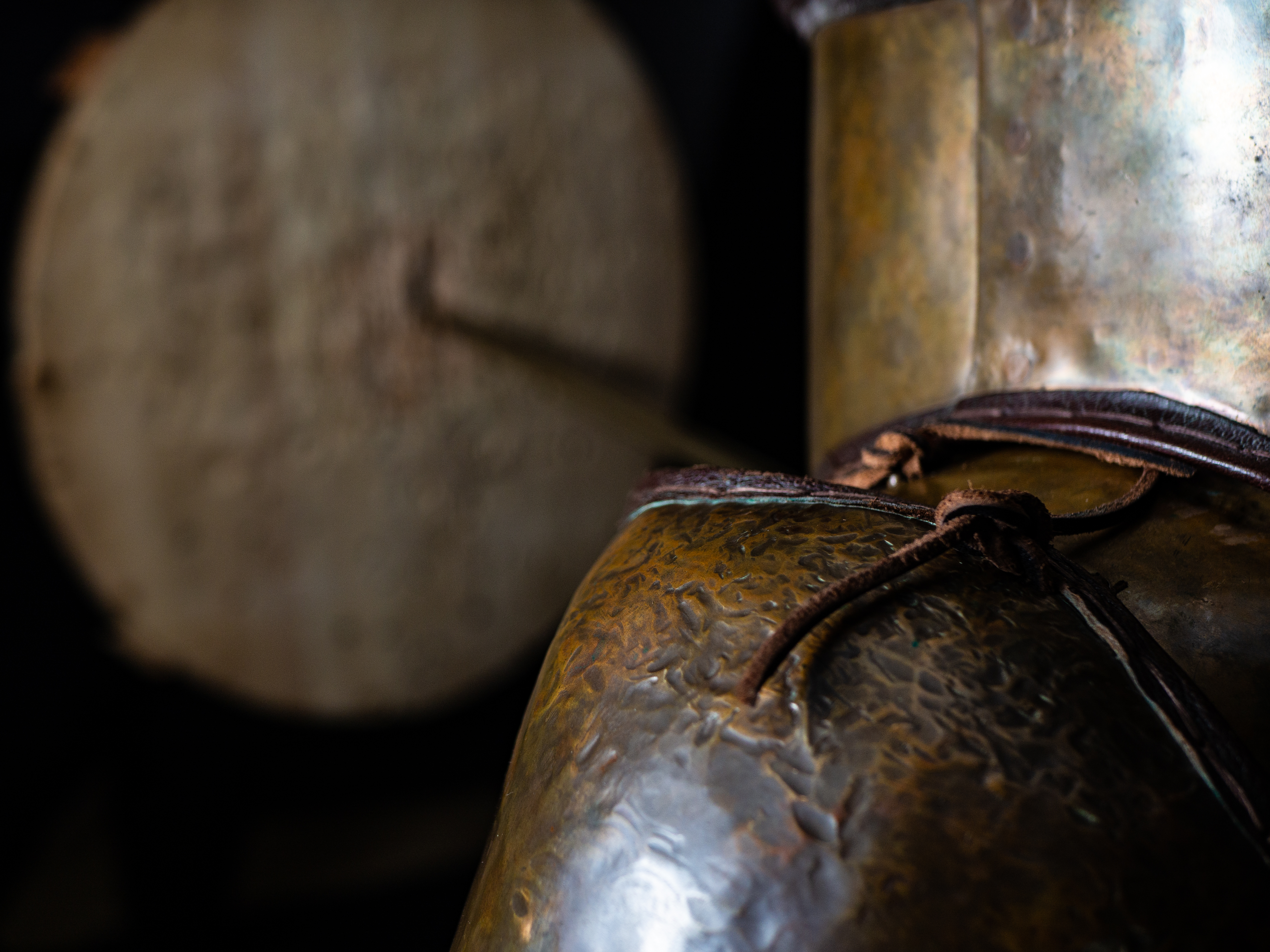 |
| 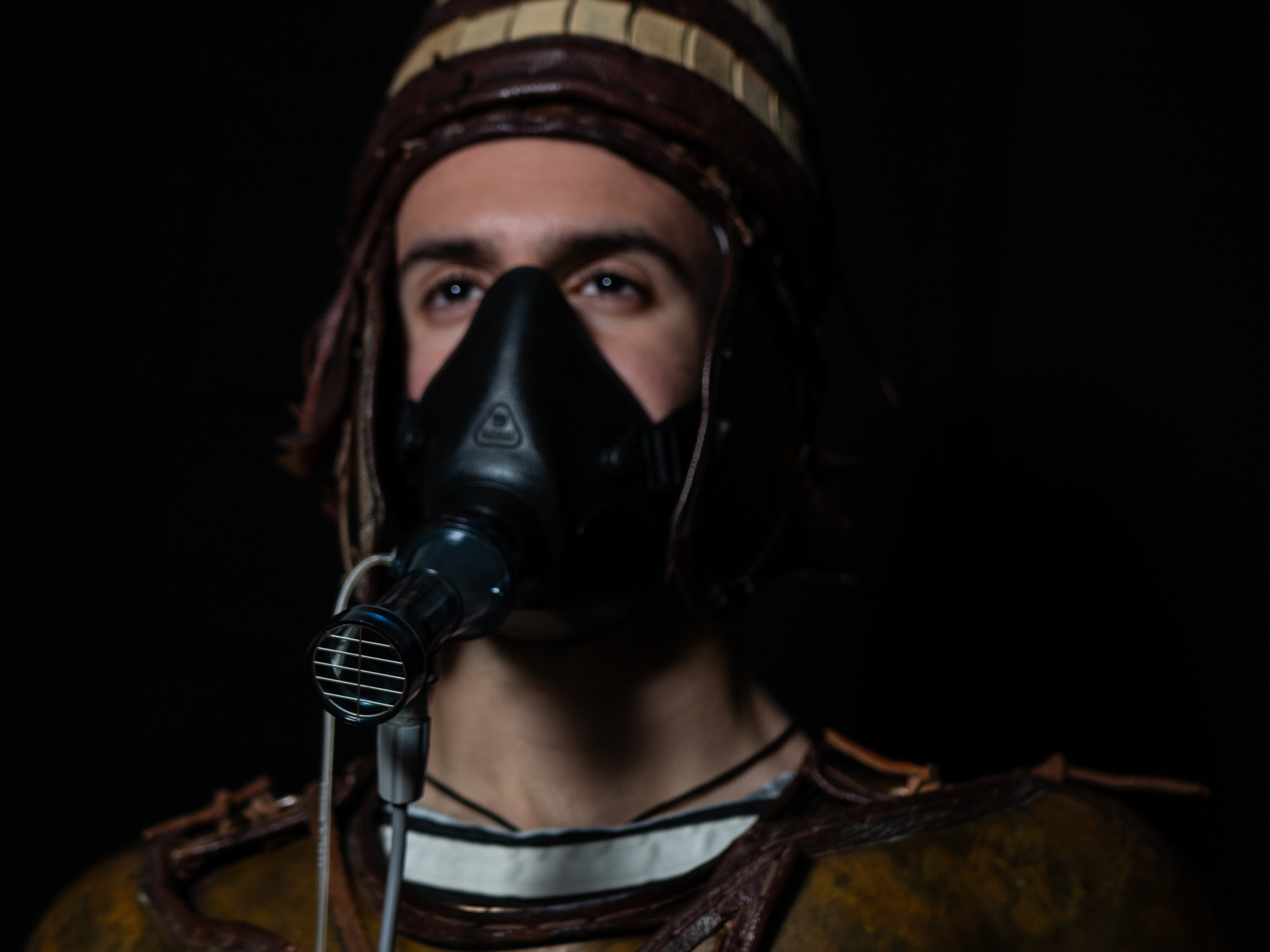 | 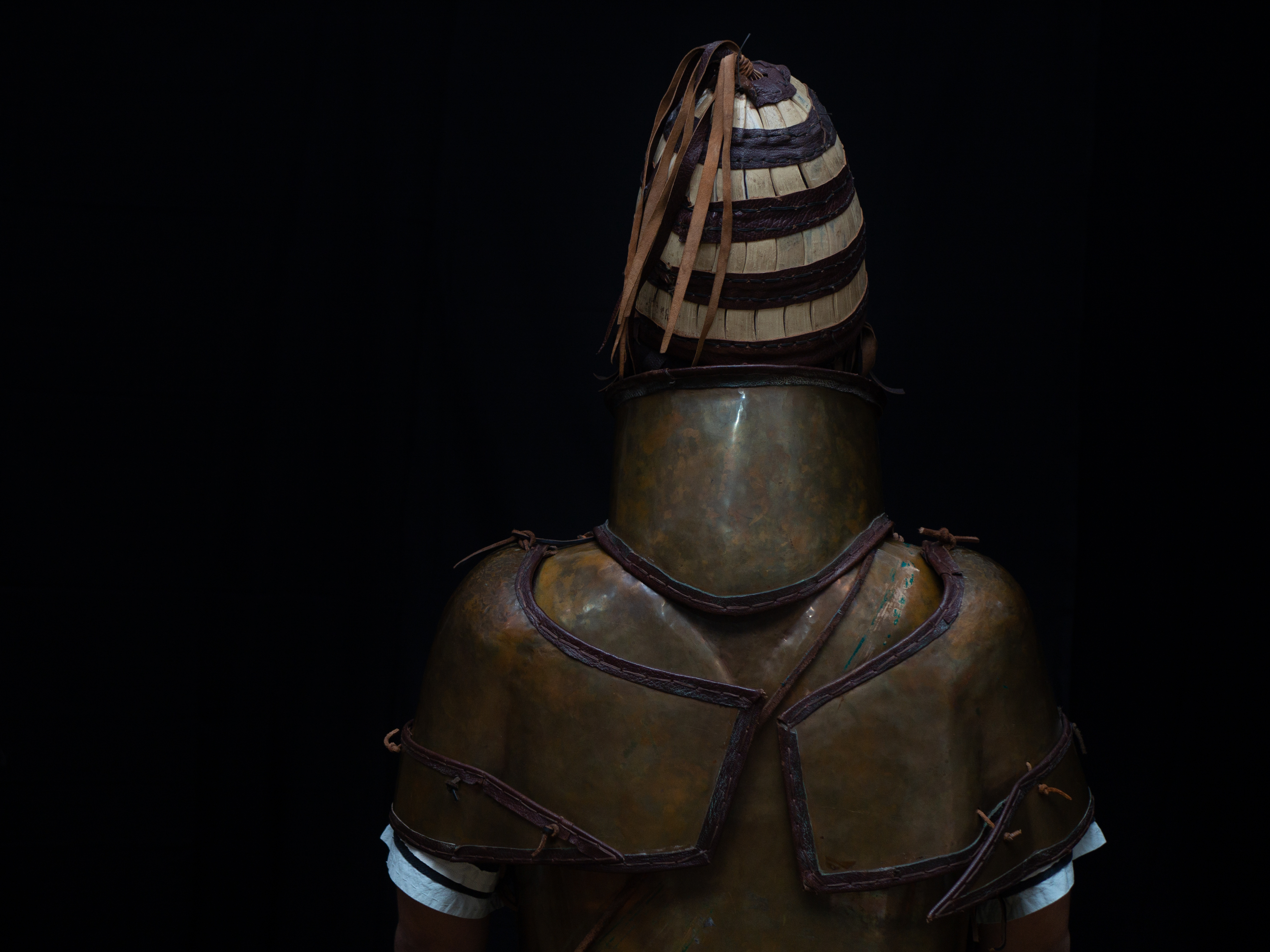 |

## Section 5. Numerical study using a Late Bronze Age warrior model (Study 4)

### Section 5.1. Concept and scope

As shown in Section 4, special forces personnel can complete the developed Late Bronze Age combat simulation protocol of elite warriors despite the significant fatigue and psychophysical stress caused by this exhausting 11-hour protocol. Our participants were continually encouraged to perform at a maximum effort and this performance was achieved to a large extent, as shown by the significant amount of fatigue reported as well as the reactive leucocytosis observed at the end of the combat simulation protocol. However, it remains almost impossible to create a life-threatening situation in a controlled laboratory setting. It is certain that our participants could have reached a higher level of effort, had their life depended on it, as evidenced by the measurement of heart rate during the combat simulation protocol. Furthermore, the above-mentioned tests were conducted in laboratory conditions, thus, without exposure to outdoor environmental conditions (e.g. solar radiation) that could further affect the thermal exchanges of the subjects and provide an extra thermal burden on their thermoregulatory system. To circumvent this limitation and address the question of whether it would have been physically possible to perform the developed Late Bronze Age combat simulation protocol in real rather than laboratory conditions, we used the information collected in the previous sections to develop a numerical model, henceforth called the Late Bronze Age Warrior model, simulating the thermoregulatory system of the elite Late Bronze Age warrior wearing Dendra-type armour.

The Late Bronze Age Warrior model was developed starting from the Tanabe 65-node model,[^121^](#_ENREF_121) i.e. a thermo-physiological model that has been used to predict the body temperature of virtual individuals as well as their the heat/mass exchanges with the surrounding environment, to which we incorporated several modifications to account for phenomena that are key for the goal of this study. The original Tanabe 65-node model calculates the temperature of 16 body parts (feet, legs, thighs, hands, arms, shoulders, pelvis, chest, back, head) – each divided in 4 layers (core, muscle, fat, skin), as well as the average temperature of the blood, for virtual subjects having a height and a weight of 172 cm and 74.4 kg, respectively, which is similar to the average height and weight of the participants of the experimental study.

Given that this study required detailed account of the sun-induced radiant exchanges of a virtual subject performing physical activity while wearing Dendra-like armour (instead of typical clothing considered in the Tanabe 65-node model), we incorporated modifications to the model code describing more accurately the effects of the clothing/armour and the radiant exchanges on the body thermal exchanges with the environment. Firstly, we added 16 new “clothing” nodes corresponding to the clothing/armour on each body part, because skin and clothing/armour temperatures are different, and it is important to calculate them separately since heat accumulation in the clothing/armour is relevant in transient scenarios. For that purpose, we modelled and implemented the heat and moisture transfer in the “clothing” node based on the approach of Voelker et al.[^122^](#_ENREF_122) Secondly, we modelled and implemented the calculation of the long-wave radiation exchanges in an explicit way using the Stefan-Boltzmann equation, instead of the simpler (and less accurate) approach based on an equivalent radiation heat transfer coefficient (as done in the original Tanabe 65-node model). We also incorporated a set of equations relating the angle of the sun (relative to the virtual subject) to the percentage of the body surface area that is, at that angle, irradiated.[^123^](#_ENREF_123) This is important to accurately estimate the variation of the solar loads as the sun position varies along the day (e.g. the back of an individual facing the sun is not irradiated). Finally, we added an efficiency parameter that accounts for the energy lost by work during physical activities, which is particularly important when the metabolic rate is high.[^124^](#_ENREF_124)

The modifications described above were implemented to develop the Late Bronze Age Warrior model and its predictions were compared, together with those obtained with the Tanabe 65-node model, with results from several independent experimental studies (Figure s16).

An implementation of the Late Bronze Age Warrior model has been placed in an online data repository and is freely available for research and educational purposes (<https://doi.org/10.6084/m9.figshare.12090831.v1>).[^125^](#_ENREF_125)

### Section 5.2. Validation of the Late Bronze Age Warrior Model against literature

In a study by Gavin et al.[^126^](#_ENREF_126) the rectal temperature of individuals wearing different clothing ensembles was measured during exercise. The experimental protocol was: 20 min of seated rest inside the chamber; 30 min of running at 70 % V̇O_2_max; 15 min of walking at 40 % V̇O_2_max; and 15 min of seated rest. The chamber conditions were: 30 °C, 35 % relative humidity, simulated wind velocity of 3, 6 and 11 km/h for rest, walking and running, respectively. We tested the accuracy of the original Tanabe model and the Late Bronze Age Warrior model against the experimental results of rectal temperature when the individuals were semi-nude (Figure s16a) and dressed in a light attire (Figure s16b). The results using the Late Bronze Age Warrior model were able to better predict the variation in the rectal temperature in both semi-nude and the dressed participants, than those obtained with the original Tanabe model.

A study by Zhao et al.[^127^](#_ENREF_127) assessed the heat recovery of individuals exercising until exhaustion. The clothed individuals exercised intermittently under a radiation lamp (450 W/m^2^) inside an environmental chamber (34 °C, 60 % relative humidity, 0.3 m/s air velocity), followed by a 6 min active recovery period, and a 30 min of passive recovery. The resulting core temperature (Figure s16c) obtained from experimental data (average core temperature of participants and limits of 95 % confidence interval) was well predicted both during the fighting and recovery sections of the Late Bronze Age combat simulation protocol, when the Late Bronze Age Warrior model was used.

In a study by Bourlai et al.[^128^](#_ENREF_128), subjects wearing thermal protective clothing (with high evaporative resistance) walked on a treadmill for 45 min while carrying a 20 kg backpack in heated conditions (38.8 °C and 19.2 % relative humidity). The average core temperature of all participants during the Late Bronze Age combat simulation protocol is shown in Figure s16d together with 95% confidence interval and the predictions by the Late Bronze Age Warrior model and the original Tanabe model. The core temperature predictions by the Late Bronze Age Warrior model follow more closely the obtained experimental temperatures.

The above results show that the Late Bronze Age Warrior model can accurately predict the core temperature of individuals exercising at high intensity, individuals performing intermittent work (Figure s16a-c), individuals exposed to solar radiation (Figure s16c), and individuals wearing different clothing ensembles (Figure s16a-d) including those with high evaporative resistance (Figure s16d) and for different environmental conditions.

**Figure s16.** Comparison between experimental data from several studies and the numerical prediction using the Tanabe model and the Bronze Age Warrior model; the shaded regions represent the 95% confidence interval.

| 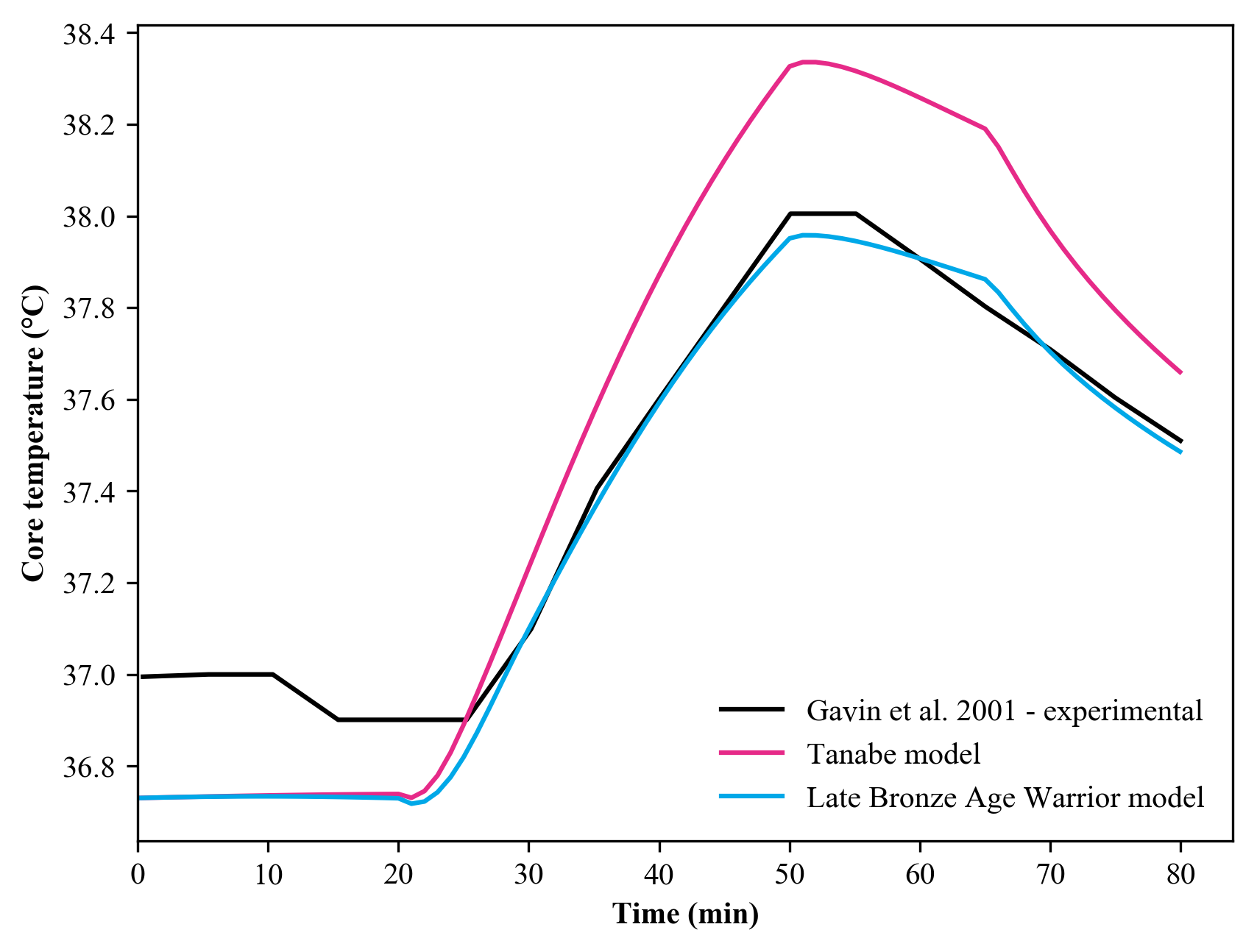 | 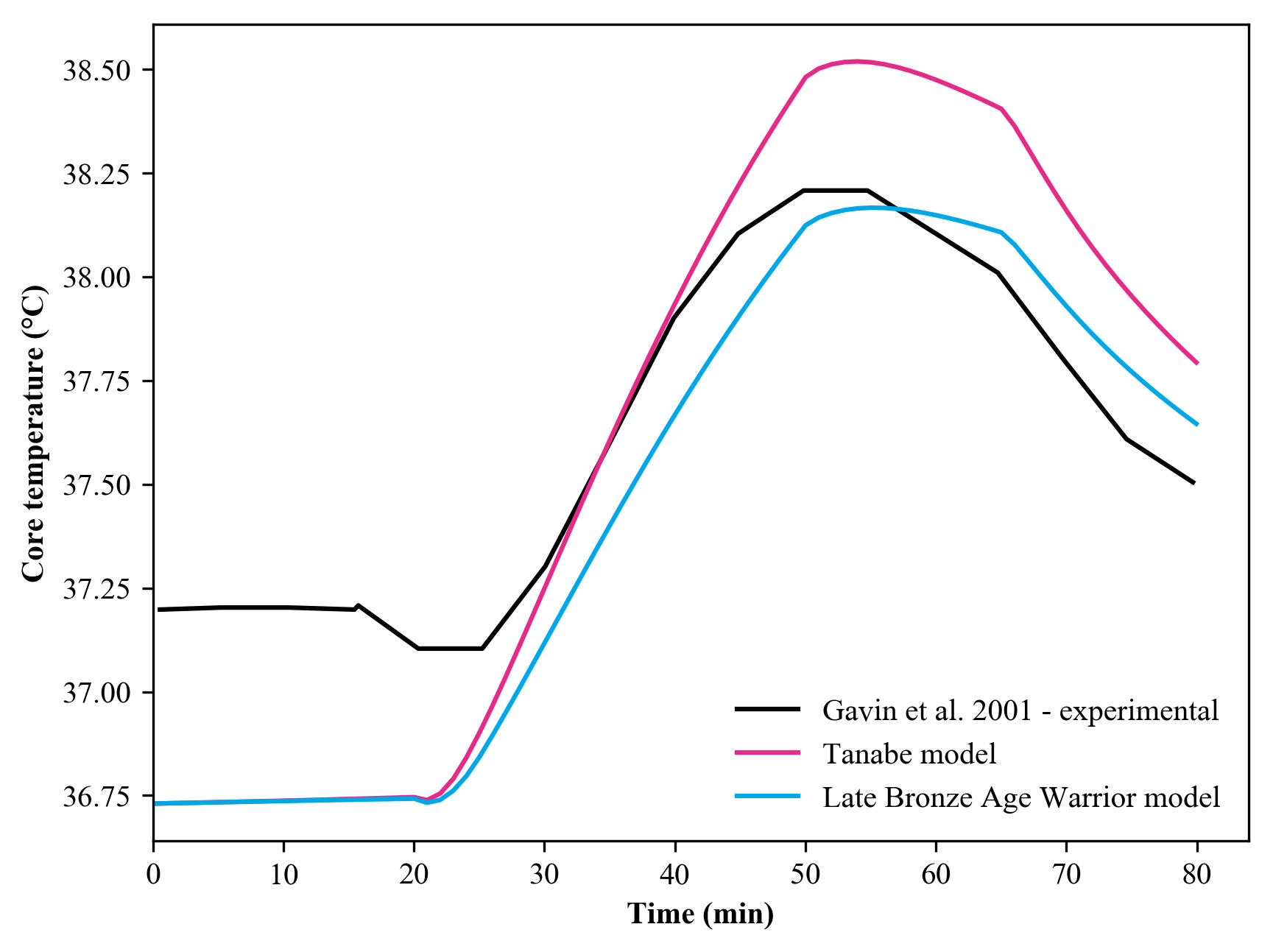 |
| --- | --- |
| (a) | (b) |
| 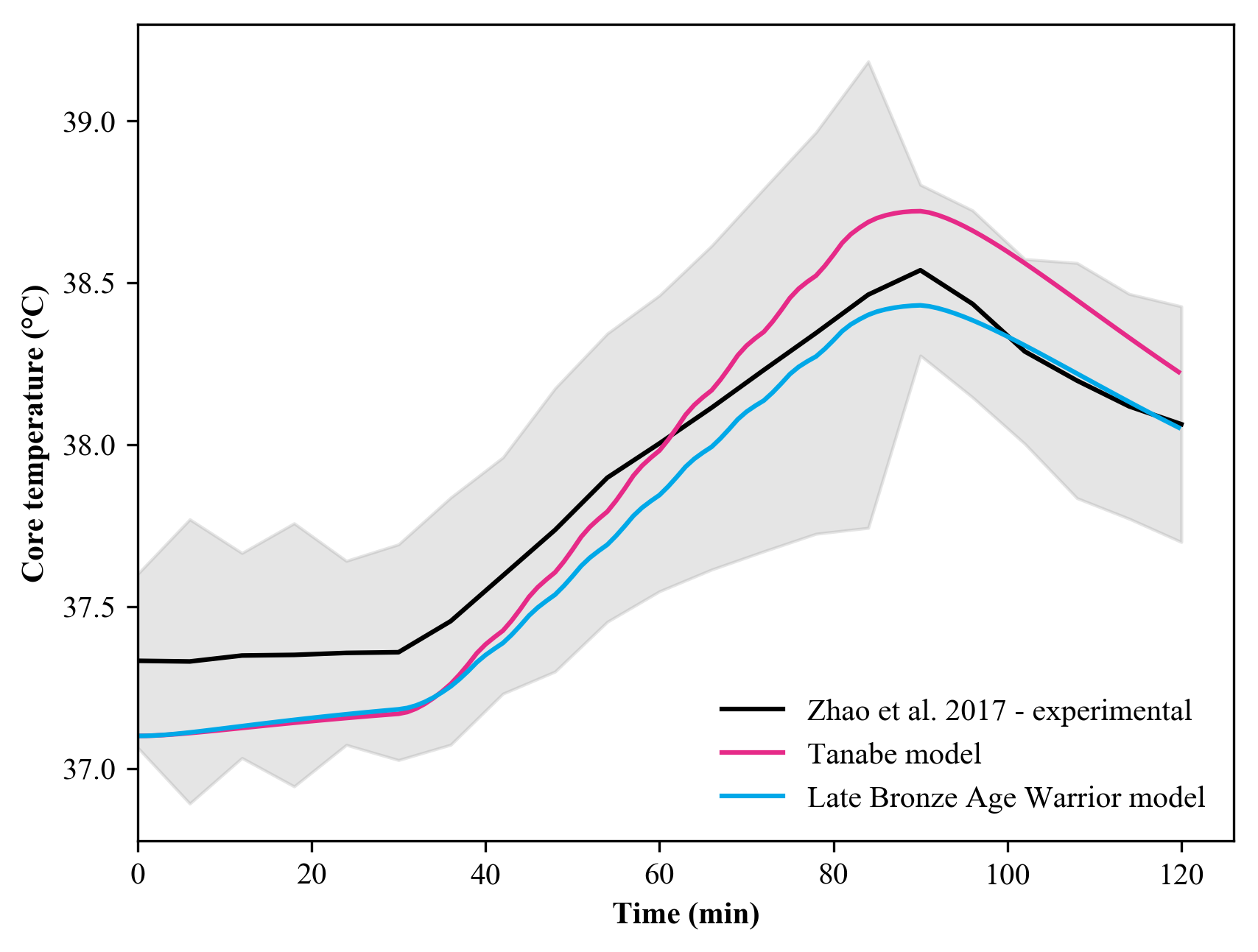 | 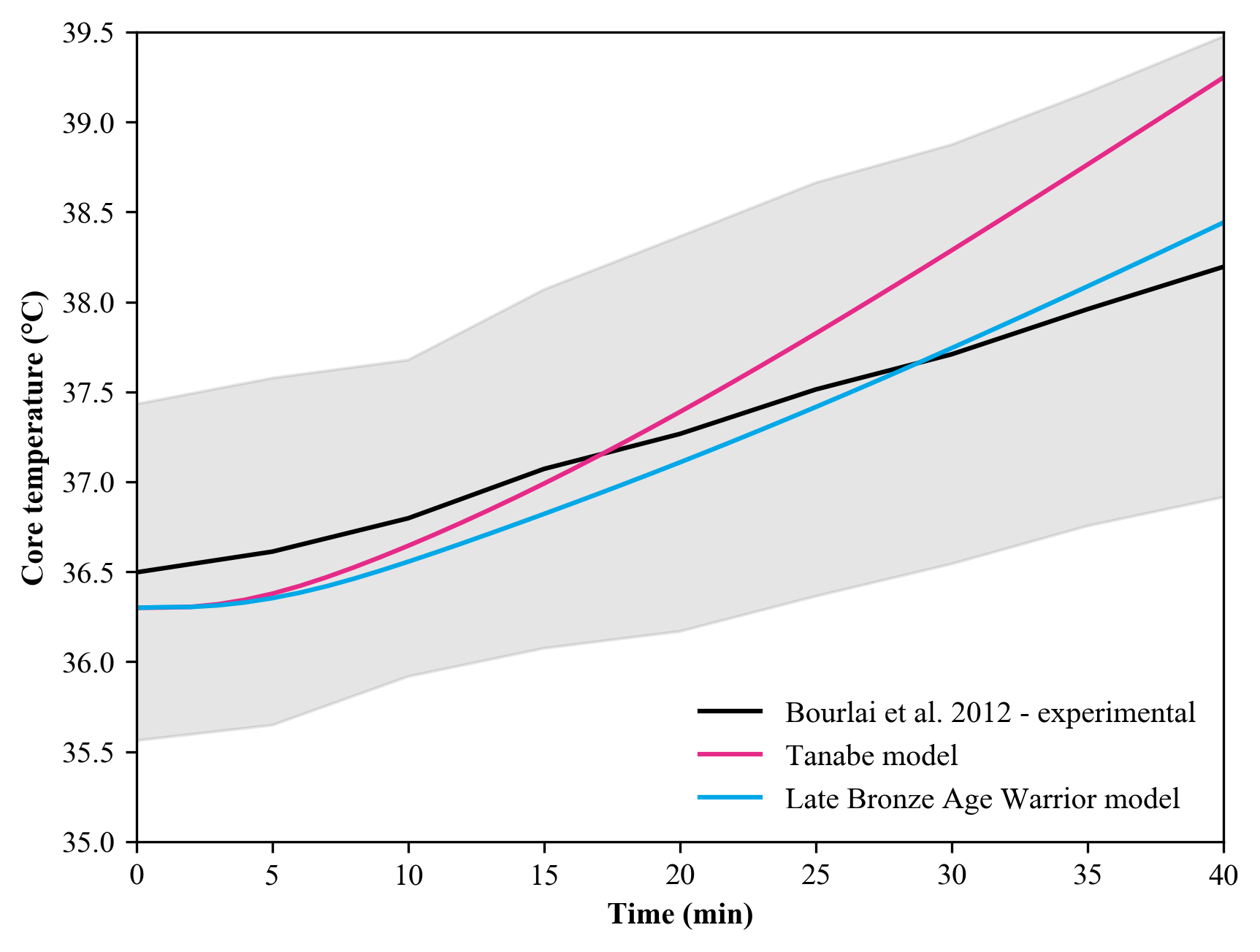 |
| (c) | (d) |

### Section 5.3. Assumptions for the numerical simulations

XRF analyses of the metal of the armour (see Section 4.5 above) indicate a composition similar to that of alloy C52400[^129^](#_ENREF_129), and as such, we assumed that the thermal properties of the original Dendra armour were similar to those of this alloy. However, the composition of the Dendra armour replica (Section 4.5) is different from that of the original armour, being instead similar to that of alloy C21000.[^129^](#_ENREF_129) For that reason, we estimated the thermal properties of the replica based on those of this alloy. The optical properties of both the replica and the original armours were estimated based on common properties for non-polished alloys (Table s7).[^130^](#_ENREF_130)^,^[^131^](#_ENREF_131)

| **Table s7.** Properties of the replica and original Dendra-type armours. | | |
| --- | --- | --- |
| **Variable** | **Replica** | **Original** |
| Specific heat [J·kg^-1^·K^-1^] | 380 | 380 |
| Density [kg·m^-3^] | 8860 | 8780 |
| Thermal conductivity [W·m^-1^·K^-1^] | 234 | 50 |
| Absorptivity [-] | 0.3 | 0.3 |
| Emissivity [-] | 0.22 | 0.22 |
| Thickness [m] | 0.0015 | 0.0015 |

The helmet of the original Dendra armour is mostly made of leather and wild boar tusks, therefore, we based the thermal properties of this helmet on those of ivory.[^132^](#_ENREF_132)^,^[^133^](#_ENREF_133) On the other hand, the helmet of the replica is made of cast resin and, thus, its thermal properties (Table s8) are different from those of the original helmet.[^130^](#_ENREF_130) The optical properties for both helmets were assumed to be similar.

| **Table s8.** Properties of the replica and original Dendra-type helmets. | | |
| --- | --- | --- |
| **Variable** | **Replica** | **Original** |
| Specific heat [J·kg^-1^·K^-1^] | 1000 | 1150 |
| Density [kg·m^-3^] | 1200 | 1700 |
| Thermal conductivity [W·m^-1^·K^-1^] | 0.15 | 0.35 |
| Absorptivity | 0.45 | 0.45 |
| Emissivity | 0.9 | 0.9 |
| Thickness [m] | 0.01 | 0.01 |

The warriors (and the participants in the experimental work) wore a clothing layer under the armour, whose properties were estimated based on typical undergarment data.[^134^](#_ENREF_134) Moreover, it is possible that warriors used a cloth surcoat over their armour similar to that used by the Knights Templar, which may have been important not only to show a symbol (e.g. the Templar cross) but also to reduce the thermal load on the body. Therefore, the effect of a white surcoat on the core temperature of elite warriors was also numerically investigated, considering optical properties (Table s9) based on those of cotton.[^135^](#_ENREF_135) Furthermore, since the white surcoat is an additional clothing layer, it causes an increase in the total thermal resistance. We assumed that the surcoat and the undergarment were made from similar materials and that the surcoat was half as thick as the undergarment (i.e. the surcoat thermal resistance if half of that from the undergarment).

| **Table s9.** Optical properties of the different materials and surfaces used in the model. | | |
| --- | --- | --- |
|  | **Emissivity (-)** | **Absorptivity (-)** |
| Skin | 0.95 | 0.6 |
| Underclothes | 0.9 | 0.78 |
| White surcoat | 0.77 | 0.3 |

The evaporative resistance of permeable clothing layers is calculated, in the thermoregulation model used in this work, using an equation (i.e. eq 9 and 10 in a study by McCullough and Kenney[^136^](#_ENREF_136)) that relates the evaporative resistance to a permeability coefficient. However, this is only appropriate for permeable clothing layers, which is not directly applicable to armour, which includes non-permeable regions (e.g. made from metal parts) separated by multiple openings (i.e. between the different metal parts) that allow for evaporative exchanges. We thus based the estimation of the armour’s evaporative resistance on data from existing clothing ensembles. Figure s17 shows the evaporative resistance for different clothing ensembles, measured using the mass and the heat methods.[^137^](#_ENREF_137) We consider the evaporative resistance of a climber’s ensemble (CLM bar in Figure s17), i.e. 70 Pa·m^2^·W^-1^, to be a reasonable estimate of the armour’s evaporative resistance because: (1) it is 2-3 times higher than that of a light attire (HV bar, T-shirt and trousers, in Figure s17), (2) it is lower than that for a full firefighter outfit (the multiple openings of the armour allow convective mass transfer which decreases the apparent evaporative resistance), and (3) it is still a relatively high evaporative resistance which makes our analysis conservative.

| **Figure s17.** Evaporative resistance of multiple ensembles, determined by two different methods and adapted from a study by Wang et al.[^137^](#_ENREF_137); Nude – underwear only, L – light clothing, HV – high-visibility clothing, MIL – military clothing, CLM – climber overall, FIRE – firefighter. |
| --- |
| 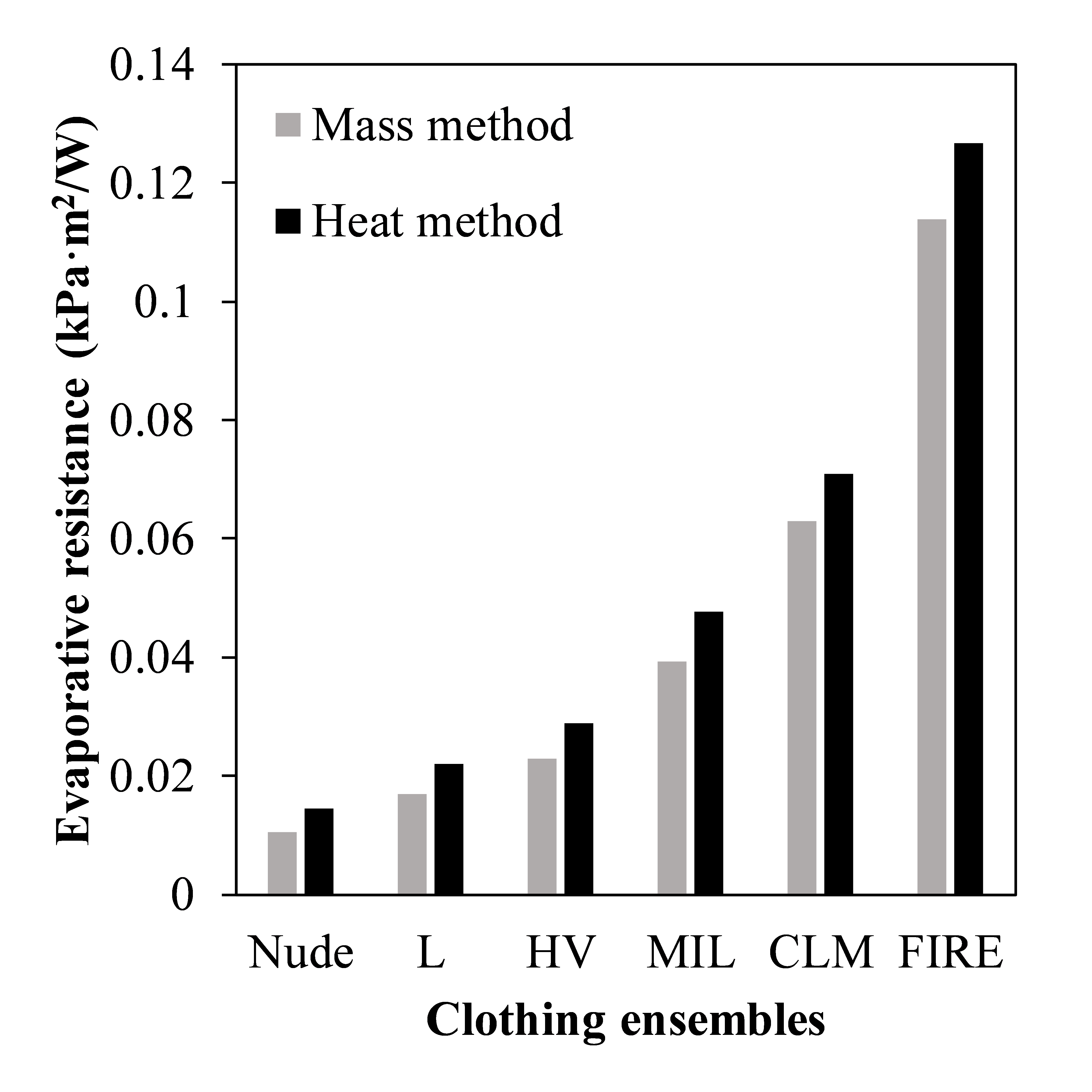 |

To account for the increase in body surface area when the armour is worn, a clothing factor (fcl) of 1.5, usual for winter ensembles and protective clothing (ISO 9920:2003), was used in this study. When the armour was not used, a clothing factor of 1.2 was assumed, because the participants wore an additional clothing layer underneath.

The metabolic rate of some of the activities performed during the Late Bronze Age combat simulation protocol experiments were measured (Figure 5, main text). Then, we assumed that the metabolic rate of the “baseline” and “break” activities were the same as the typical resting metabolic rate (58 W/m^2^), and that the “deployment” and “battle formation” activities caused the same metabolic rate as the “chariot” activity (due to similarities). This was needed because the metabolic rate for those activities was not measured.

| **Table s10.** Average metabolic rate calculated for each activity; the metabolic rates of “baseline” and “break” were assumed to correspond to that of a resting individual (58 W/m^2^), and the metabolic rates of “deployment” and “battle formation” were assumed to be similar to that of “Manoeuvring (on chariot)”. | | |
| --- | --- | --- |
|  | **Activity** | **M (W / m^2^)** |
| 0 | Baseline | 58 |
| 1 | Deployment | 80 |
| 2 | Battle formation | 80 |
| 3 | Manoeuvring (on foot) | 178 |
| 4 | Foot warrior vs chariot | 227 |
| 5 | 1-on-1 | 213 |
| 6 | Manoeuvring (on chariot) | 80 |
| 7 | Break | 58 |
| 8 | Chariot vs chariot | 118 |
| 9 | Chariot vs ship | 171 |

An air speed of 0.5 m/s (corresponding roughly to an indoor scenario, without wind) was used to simulate the experimental conditions in the laboratory. Natural convection around a seated human body generates an air motion of around 0.2 m/s around the individual.[^138^](#_ENREF_138) We used an air speed slightly higher than that because there were one or more individuals moving inside the room during the experiments, which increases the air motion. An alternative would be to use a convective coefficient equation derived for running on treadmills indoors which uses the treadmill velocity as an input, instead of the air velocity.[^139^](#_ENREF_139) However, this yields the same coefficient for all the body parts, and the coefficients are known to vary greatly between body parts.[^140^](#_ENREF_140) That would also be inadequate for the tactical/combat parts of the Late Bronze Age combat protocol where the treadmill was not used. Therefore, we opted to estimate the air speed and use the equations specific for each body part that were reported by de Dear et al.[^140^](#_ENREF_140) to calculate the corresponding convective coefficients.

The NOAA solar position calculator was used to predict the position of the sun for the intended day and location, during the course of the day. Regarding the solar intensity I_dir_, the ASHRAE clear sky equation was used to predict the solar irradiance for June.[^141^](#_ENREF_141) This is appropriate because there are very few reports of clouds during the battle. In Figure s18, the parameters A and B, which depend on the month, were selected for June, and *β*, which is the elevation angle of the sun, was calculated by the NOAA solar position calculator throughout the day. Then, the sky temperature was estimated based on the sky emissivity *ε* (approximately 0.8 at the top of the atmosphere[^142^](#_ENREF_142)) and the ambient temperature.[^143^](#_ENREF_143)

**Figure s18.** (a) Solar irradiation during the day, (b) equation used to calculate the solar irradiation, (c) equation that calculates sky temperature based on ambient temperature and (d) solar elevation along the day that was considered in this study.

| 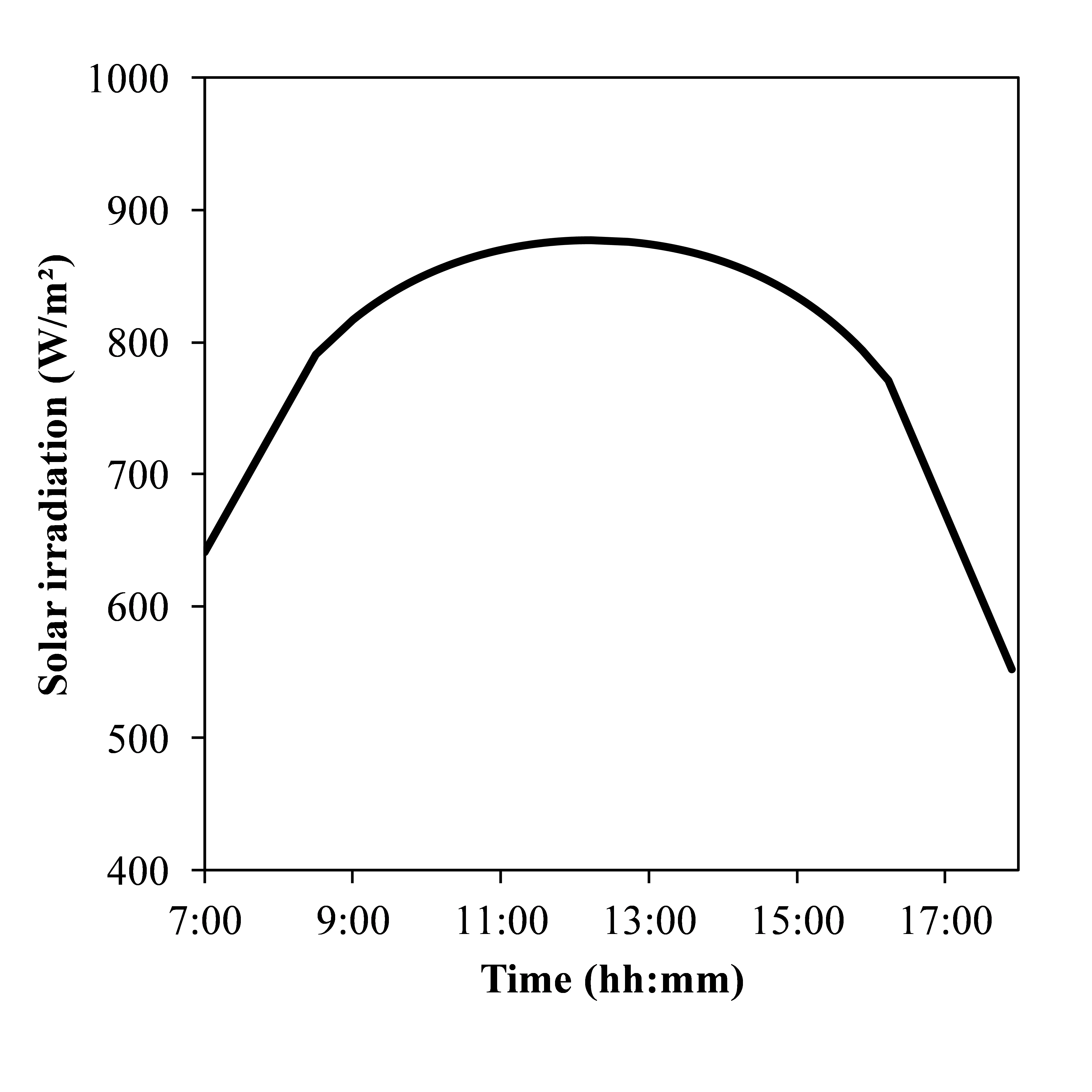 | $I_{dir}=Ae^{\frac{-B}{\sin\beta}}$ (b) |
| --- | --- |
|  | $T_{sky}={(\varepsilon_{sky}\times{T_{amb}}^{4})}^{1/4}$ (c) |
|  | 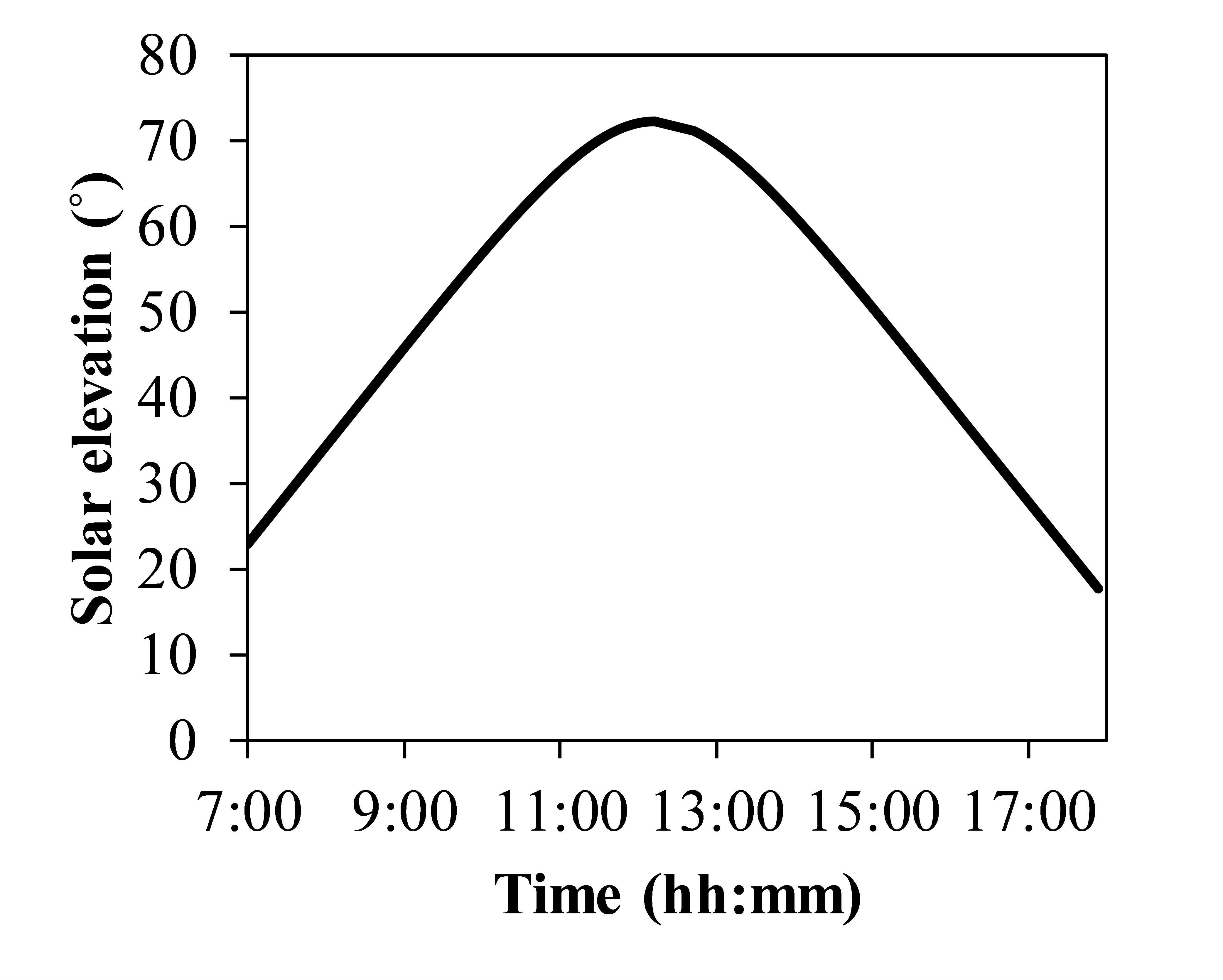 |
| (a) | (d) |

During the comparison of the predictions of the Late Bronze Age Warrior model against the data obtained in the experimental laboratory tests, the environmental conditions were chosen to match those used experimentally (see the experimental data in Figure s19 as the average plus 95 % confidence range for the participants).

**Figure s19.** Experimental data of air temperature vs time (left) and relative humidity vs time (right) for all the participants, expressed as average and 95% confidence interval.

| 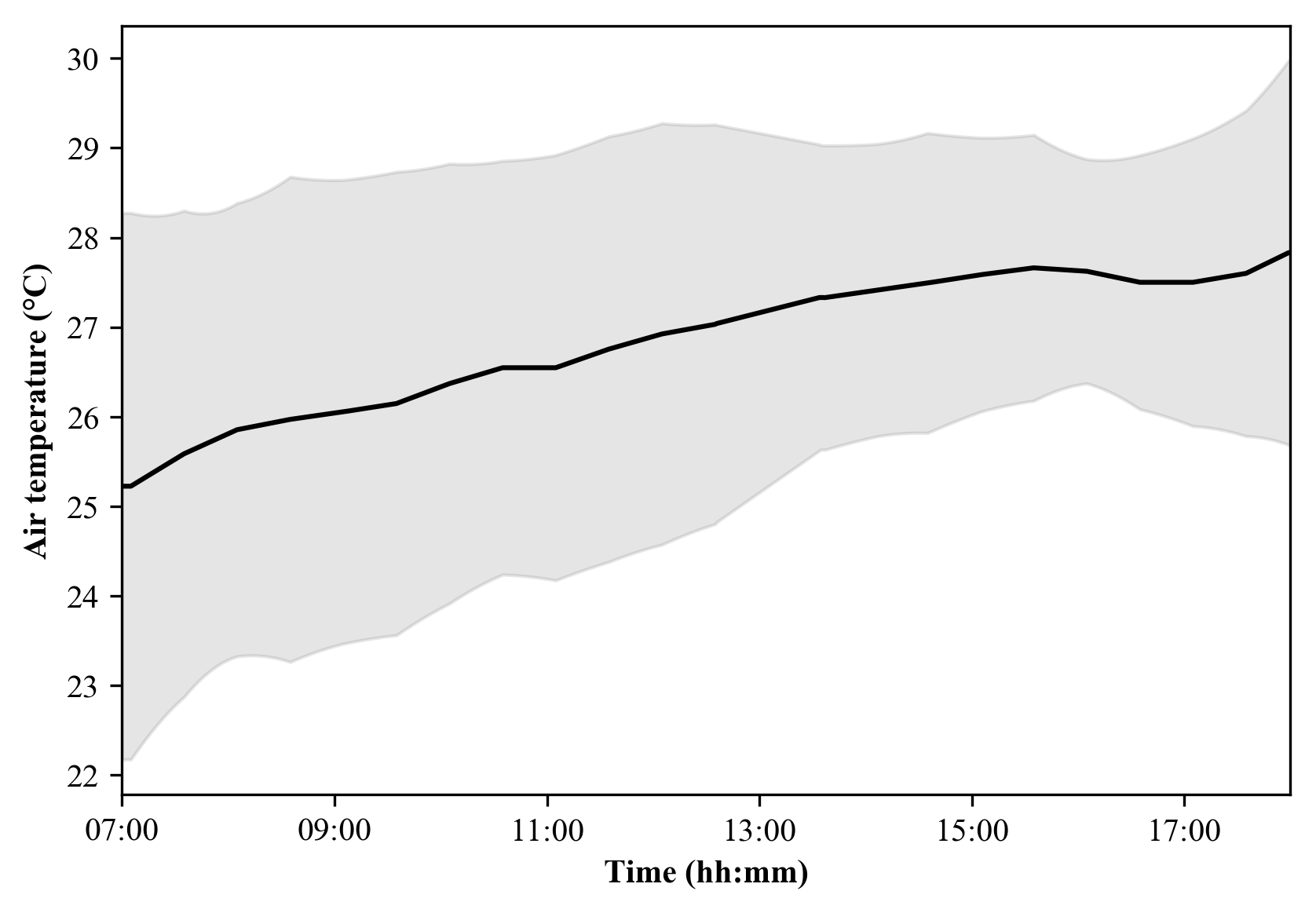 | 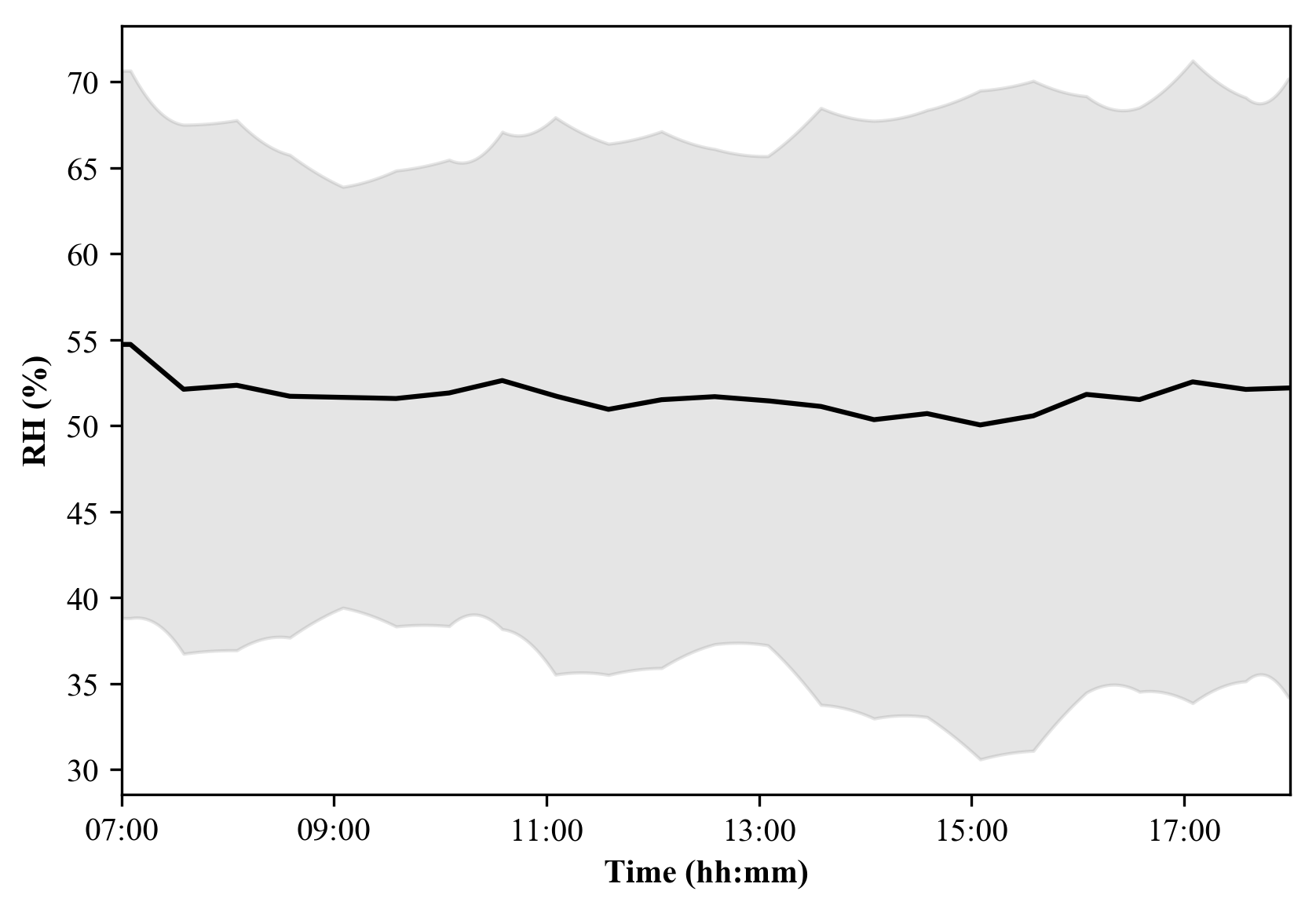 |
| --- | --- |

With this in mind, we selected a relative humidity of 50 % for the simulations (assumed constant throughout the day). In addition, since air temperature during the experimental tests increased throughout the day at an approximately linear rate, we used linear regression to obtain an equation relating the air temperature to the time of the day:

$$T_{air}=0.236t+25.2$$

These parameters were used in an Excel sheet developed for the purpose, which automatically updates the air velocity and metabolic rate in each step based on the description code of each Late Bronze Age combat simulation protocol activity.

### Section 5.4. Simulation cases and goals

In the first step, we simulated a case similar to the experimental setup, whose assumptions were explained in the previous Section. Our goal with this (case 1) was to check if the model adequately predicted the average core temperature of the participants during the experimental tests of this study. For that reason, in case 1 the virtual subject wore the replica of the Dendra-type armour and helmet, as to mimic as closely as possible the experimental setup.

After the first simulation case, we wanted to study how different variables might change the variation of core temperature along the day while wearing the original Dendra-type armour and helmet. For instance, we changed the ambient temperature and wind speed, to mimic the ranges that are typical for outdoors at the same location. Besides this, we added the effect of the radiant exchange (i.e. short-wave radiation absorbed from the sun and long-wave radiation lost to the sky), increased metabolic rate and use of a white surcoat over the armour.

| **Table s11.** Numerical cases tested in this study. | | | | | |
| --- | --- | --- | --- | --- | --- |
| **Num. case** | **Solar exposure^1^** | **Air temperature [°C]^1^** | **Wind^3^** | **Metabolic rate^4^** | **White cloth over armour^5^** |
| 1 | Absent | 25-27 | Absent | Regular | Absent |
| 2 | Present | 25-27 | Absent | Regular | Absent |
| 3 | Present | 25-27 | Absent | Increased | Absent |
| 4 | Present | 25-27 | Present | Increased | Absent |
| 5 | Present | 15-30 | Present | Increased | Absent |
| 6 | Present | 15-30 | Absent | Increased | Absent |
| 7 | Present | 15-30 | Absent | Increased | Present |
| Note: ^1^: The presence of solar exposure is modelled by the ASHRAE equation and the NOAA solar position calculator; ^2^: Air temperature is either 25-27 °C (as in the experimental protocol), or 15-30 °C (from weather station data, Figure s21); ^3^: “Absent” refers to still air with 0.5 m/s, and “Present” refers to varying velocity from weather station data (Figure s20); ^4^: “Regular” refers to the metabolic rates based on the experimental tests, and ”Increased”, refers to a 2.3 times higher metabolic rates for the combat simulation protocol phases that included fighting (Table s12); ^5^: “Present” refers to the cases implying the use of a white surcoat used over the armour. | | | | | |

With this in mind, we considered a case 2 with conditions similar to those of case 1 but including the effect of the solar exposure occurring outdoors. It is noteworthy that in outdoor scenarios the virtual subject gains heat from the sun and loses heat to the sky (whose temperature is estimated based on the sky temperature). This is in contrast with indoor scenarios, where there is no solar exposure and there are radiation exchanges with the walls of the room (which are assumed to be at ambient temperature).

In case 3, we considered an increased metabolic rate relative to the experimental tests because the heart rate of the participants during the tests (100-120 beats per minute; Figure s11) was quite low for young, healthy and physically active individuals. A literature search indicated that the typical metabolic rate for wrestling is around 500 W/m^2^,[^144^](#_ENREF_144)^,^[^145^](#_ENREF_145) thus we considered this rate as the increased metabolic rate for the “1v1 combat” activity. This metabolic rate is 2.3 times higher than the observed experimental metabolic rate. Therefore, because activities 4, 8 and 9 also included fighting (although in different conditions) the corresponding metabolic rates were also increased by the same factor (i.e. 2.3). The increased metabolic rates of these four activities together with those for the remaining steps of the combat simulation protocol are shown in Table s12 and were considered in case 3. The total energy cost using the regular and increased metabolic rates is also shown in the aforementioned table. This was calculated by multiplying the energy cost of each section of the combat simulation protocol by its duration (Metabolic rate x duration) and converting the sum of the energy cost for all combat simulation protocol steps from Joule to kcal. The increase in total energy cost is small when we compare the increased and regular metabolic rates, because the activities that had their intensity increased correspond to a small portion of the whole combat simulation protocol.

| **Table s12.** Metabolic rates of different activities corresponding to an increase of 2.3 times in fighting intensity (rows in grey correspond to the steps with increased metabolic rate) and the total energy cost of the combat simulation protocol with regular and increased metabolic rates. | | | |
| --- | --- | --- | --- |
|  | **Activity** | **Increased M (W / m^2^)** | **Regular M (W / m^2^)** |
| 0 | Baseline | 58 | 58 |
| 1 | Deployment | 80 | 80 |
| 2 | Battle formation | 80 | 80 |
| 3 | Manoeuvring (on foot) | 178 | 178 |
| 4 | Foot warrior vs chariot | 531 | 227 |
| 5 | 1-on-1 | 500 | 213 |
| 6 | Manoeuvring (on chariot) | 80 | 80 |
| 7 | Break | 58 | 58 |
| 8 | Chariot vs chariot | 276 | 118 |
| 9 | Chariot vs ship | 401 | 171 |
| **Total energy cost of protocol [kJ]** | | **8996** | **7740** |
| **Total energy cost of protocol [kcal]** | | **2150** | **1850** |

Since the air speed considered in case 1 (0.5 m/s) mimicked the conditions of the experimental tests, in case 4, we considered the effect of typical outdoor wind conditions. For this purpose, we used weather station data for the period between the 3^rd^ of June and the 8^th^ of June of 2019 (as June is best estimate for the period in which the described actions took place) for a location close to that of the battle and used it to define typical wind speeds. The recorded wind speed (Figure s20) fluctuates mostly in the range 0-3 m/s with differences between the morning and the afternoon periods. We calculated and plotted the average wind speed based on the mentioned daily data and used a 2^nd^ order regression to obtain a fitting equation representing the variation in wind speed along the day, for the period between 07:00 and 18:00. This wind speed curve specific for the dates and location in question was used in case 4 to assess the effect of wind on the convective losses of the virtual subjects and, therefore, on their core temperatures. All the other simulation parameters were similar to those of case 3 (i.e. they included solar exposure and increased metabolic rate).

| **Figure s20.** Weather station data of wind speed between the 3^rd^ and 8^th^ of June 2019 near the location of the battle. |
| --- |
| 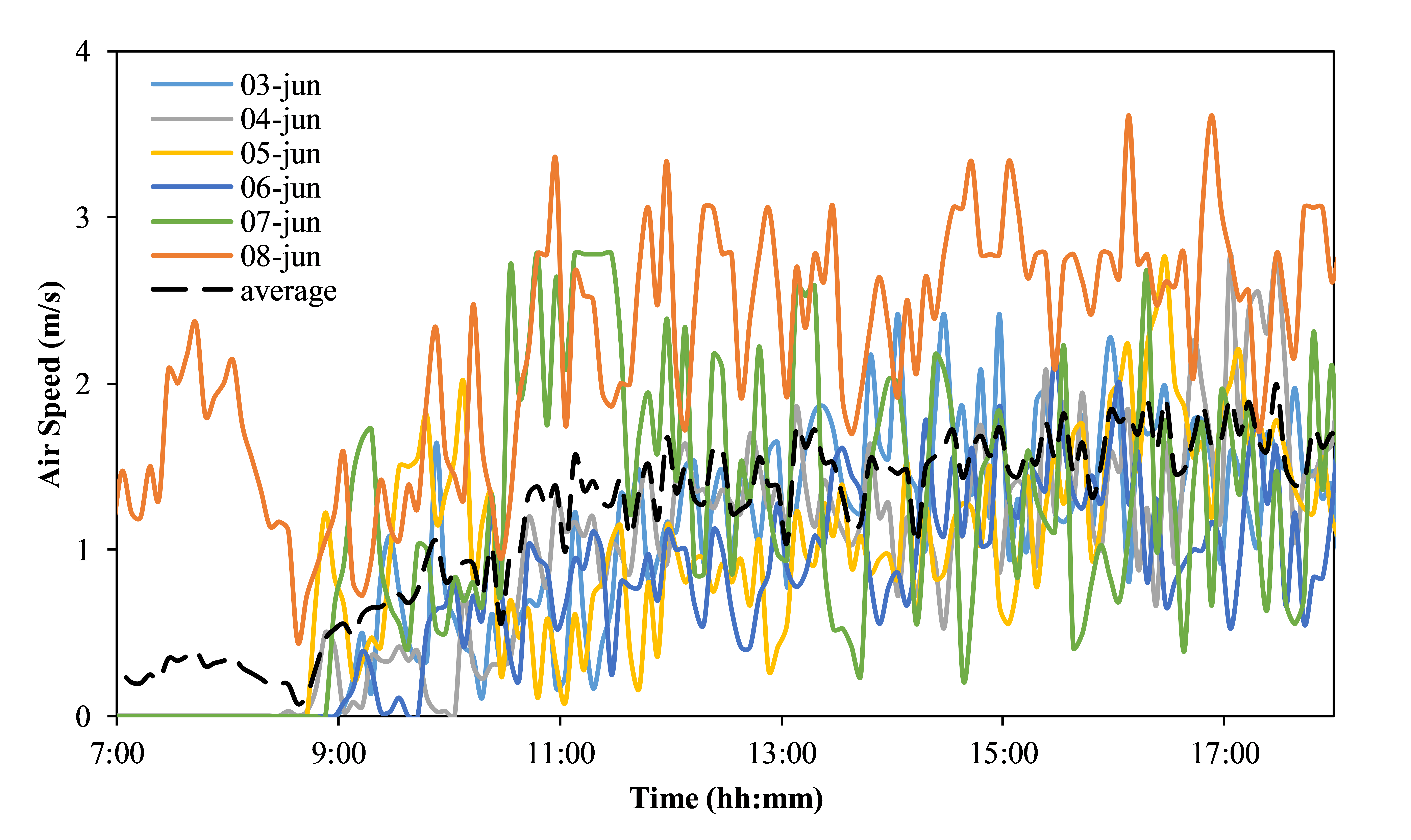 |

The air temperature considered during the experimental tests (in the range of 25-27 °C) does not represent typical outdoor temperatures during a day in June at the location in question. For that reason, we used temperature data from a weather station near the battle location for the same June days as considered for the wind speed case, and used a 2^nd^ order polynomial regression to extract the average curve relating the air temperature to the time of day (Figure s21). This curve shows that the typical outdoor temperature ranges from about 15 °C at the sunrise to about 30 °C around 14:00-15:00. Although there are some reports[^146^](#_ENREF_146) indicating that temperatures were lower during the later phases of the Late Bronze Age, it is not clear how much lower they were. For that reason, we chose to base our analysis on the current temperature variation along the day (i.e. the fitting shown in Figure s22), accepting that this makes our analysis conservative (as the higher air temperatures imply lower heat losses by convection and thus higher core temperature of the virtual subject).

| **Figure s21.** Air temperature along the day for a weather station near the location of the battle. Data corresponds to measurements between the 3^rd^ of June and 8^th^ of June 2019. |
| --- |
| 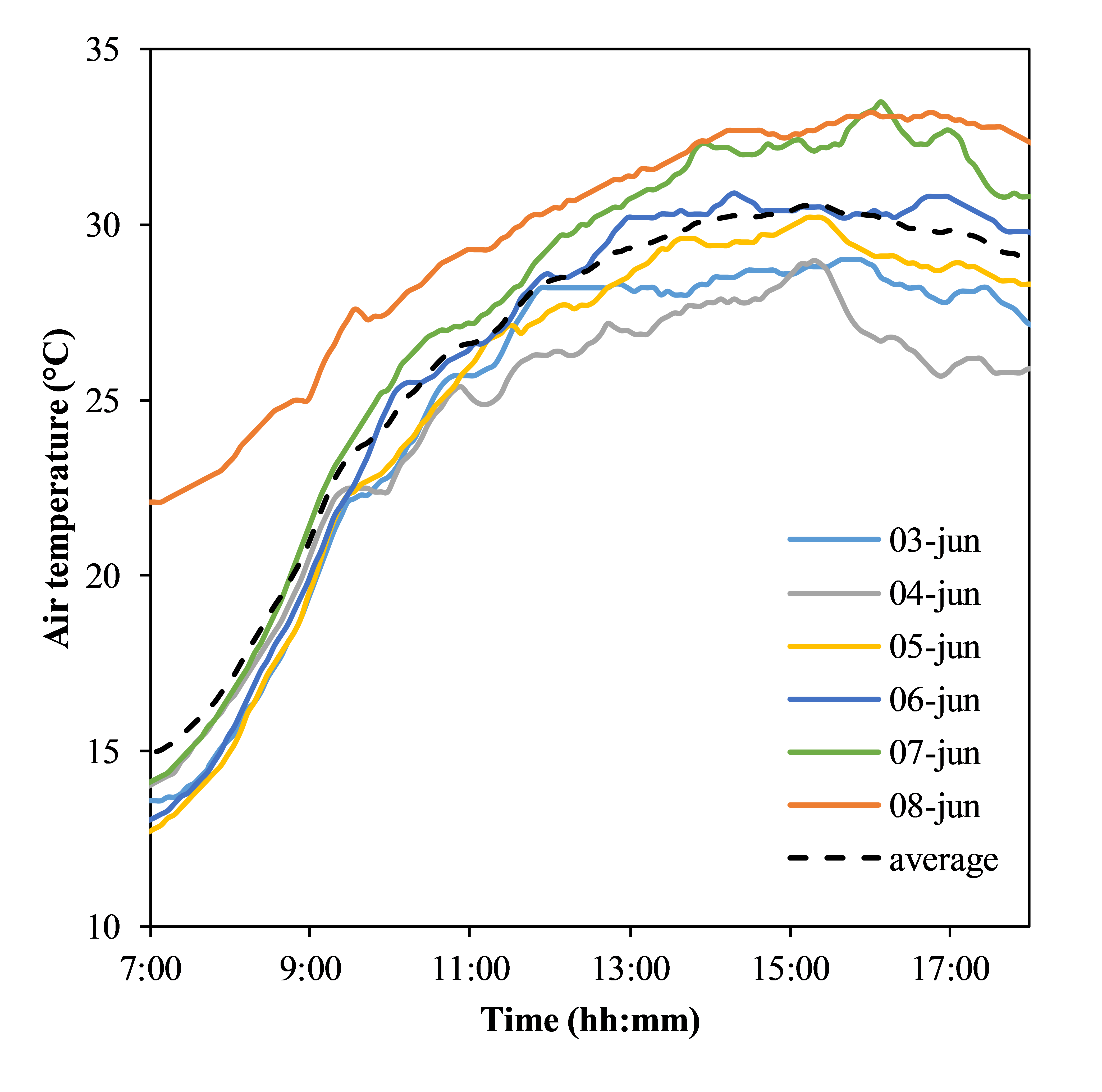 |

| **Figure s22.** Average air temperatures (blue) and wind speeds (orange) along the day and 2^nd^ order curves that fit the data. |
| --- |
| 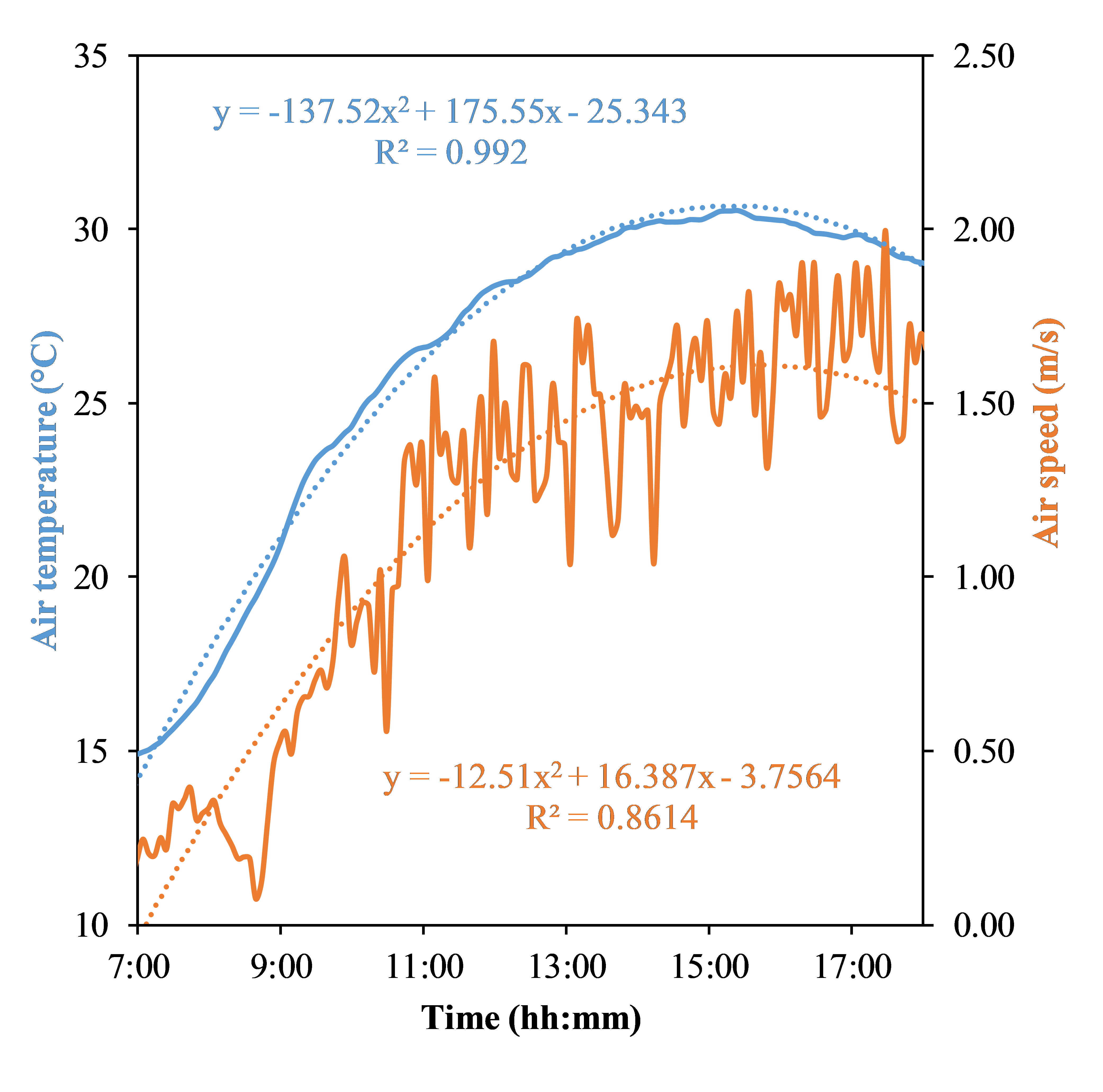 |

We studied the effect of considering the typical outdoor air temperatures and wind in the core temperatures of virtual subjects exposed to solar radiation and having increased metabolic rates (in case 5). In case 6, we simulated a scenario similar to that of case 5 but without the effect of external wind (i.e. we assumed an air speed of 0.5 m/s which mimics indoor or still air conditions). This is a hypothetical scenario intended to push the virtual warrior to the limit in terms of heat accumulation. Despite these conditions being unlikely, they were considered in case 6 as a worst-case scenario.

Finally, in case 7, we considered that the virtual subject wore a white surcoat over the armour during conditions that result in maximum heat gain (i.e case 6, where the subject is under the sun, has increased metabolic rate, there is no external wind, and the air temperature is that of typical outdoor conditions). On one hand, the white surcoat has higher emissivity than the armour (Table s7 and s9), which increases heat losses by long-wave radiation to the surrounding environment. On the other hand, as aforementioned, the use of a white surcoat increases the total thermal resistance of the clothing, which decreases the heat loss through the clothing. Case 7 allowed us to check whether a white cloth over the armour could have been used by warriors to keep their core temperature low.

Our goal was to assess if the armour could have been worn to battle during the reported time and location. To that end, we defined a core temperature threshold (38.5 °C) which, if crossed, would imply that the warrior could not have sustained the defined Late Bronze Age combat simulation protocol for the conditions considered in each simulation case. In those cases, we assumed that it was not possible to wear the armour for combat.

### Section 5.5. Results

In general, the predicted core temperatures of the virtual subject exposed to the conditions considered in case 1 (which closely mimic the experimental conditions) follow the core temperatures of the subjects participating in the experimental tests (Figure s23). The first two hours of experiment are very well predicted by the thermoregulation model with predicted core temperatures within 0.1°C of the measured temperatures. The same cannot be said of the last two hours of the experiments, where predicted and measured temperatures seem to follow different patterns (the temperature is predicted to decrease but observed to increase over time, Figure s23). However, it is unclear why the experimental core temperature increases in last two hours of the test when the participants are simply returning to camp (note that, in this last step, while the measured core temperature increases, skin temperature decreases, which seems counterintuitive). In opposition to this, the core temperature predicted by the thermoregulation model gradually decreases over time, in line with the lower metabolic rate of the activity in question (Table s10). The increased temperatures during the last two hours of the experimental tests were probably due to a combination of dehydration and diet-induced thermogenesis (as participants typically chose to eat their food rations during the latter parts of the Late Bronze Age combat simulation protocol, especially once all simulated encounters were completed), which were considered beyond the scope of this numerical work.

| **Figure s23.** Comparison between measured (experimental) and predicted (numerical case 1) core temperature during the combat simulation protocol, done to validate the Late Bronze Age Warrior model against our experimental tests. |
| --- |
| 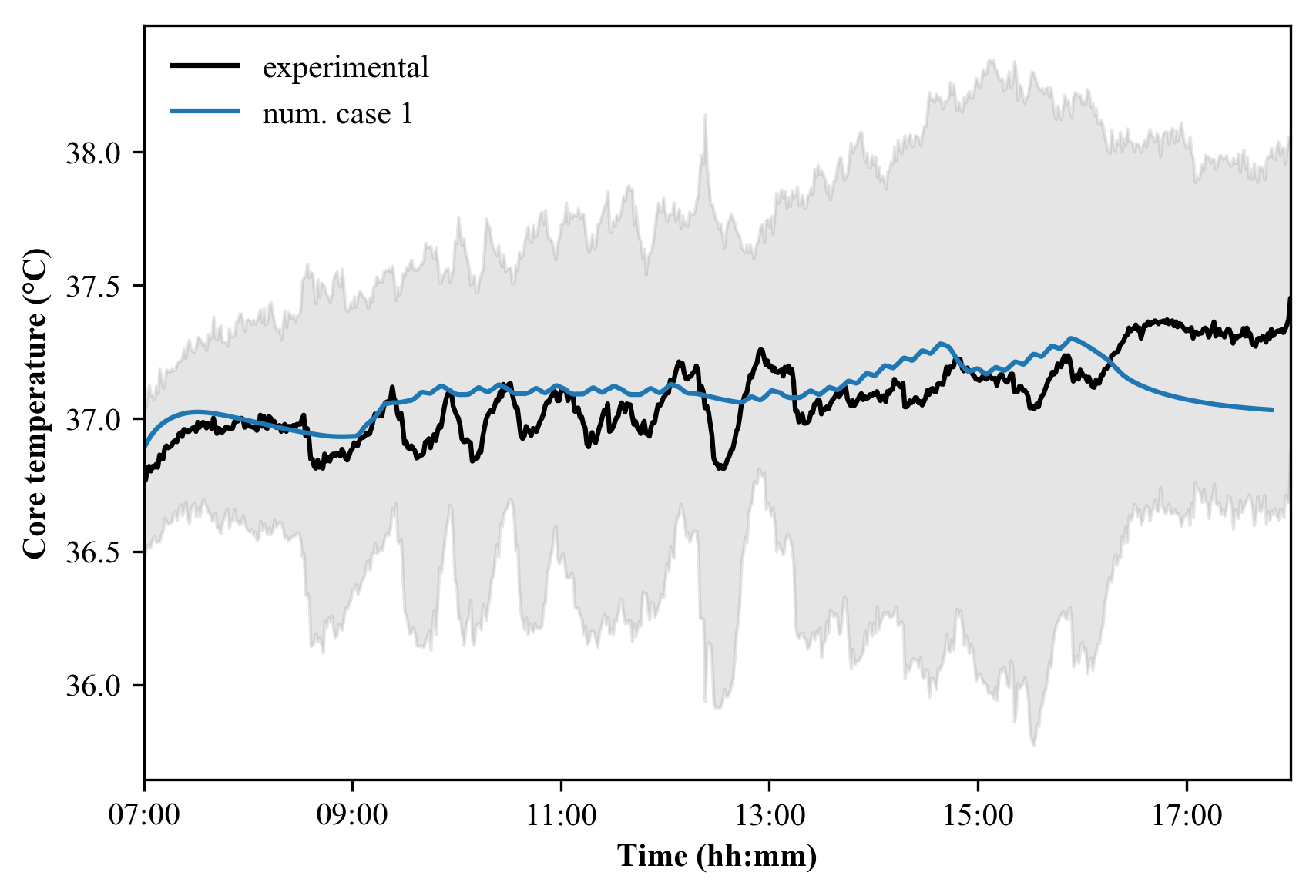 |

During the middle section of the combat simulation protocol (i.e. from 09:00 to 16:00; Figure s23), we see that the predicted core temperature is fairly similar to the measured temperature, with deviations of only 0.1-0.2 °C, which is quite reasonable. However, the measured core temperature fluctuates during the combat simulation protocol, and the predicted temperature does not. This may be due to the measurements being performed using a gastrointestinal pill, which is reported[^147^](#_ENREF_147) to produce highly variable temperatures during the pill transit (>9h, a relevant time interval for our study). On the contrary, the rectal temperature, against which the thermoregulation model was validated for various conditions (Figure s16), is known to produce more “thermally” stable curves than those resulting from gastrointestinal pill recordings. Therefore, we believe that the fluctuations in the measured temperature could be due to the measuring technique and not the result of the changing metabolic rates in the different steps of the combat simulation protocol. In other words, the obtained different temperature curves are most likely due to the pill transit and the fact that the (predicted) rectal temperature is more stable than the (measured) gastrointestinal temperature.

The results discussed above show that, in general, the thermoregulation model predicts fairly well the core temperature of the virtual subjects performing the activities considered in the combat simulation protocol. Furthermore, although it would be interesting to assess the validity of the model predictions for the core temperature of virtual warriors wearing the armour for environmental conditions beyond those considered in the experimental tests (maybe more thermally demanding scenarios leading to increasing core temperatures over time), that is not possible as the experimental tests did not include other conditions beyond those reported above. However, given the previous validation of this model against multiple independent studies and various thermal scenarios (Figure s16), we believe it can be used to predict the core temperature of virtual subjects exposed to different environmental conditions.

Figure s24 provides an overview of core temperature during the combat simulation protocol for all cases simulated. Only one case (i.e. case 6 in Table s11) implied core temperature above the defined threshold (38.5 °C), with most of them leading to, at most, 38 °C.

| **Figure s24.** Experimental and predicted core temperature throughout the combat simulation protocol for every case of Table s11. The dashed red line is the assumed core temperature threshold which, if exceeded, implies that the protocol could not be completed under those conditions. |
| --- |
| 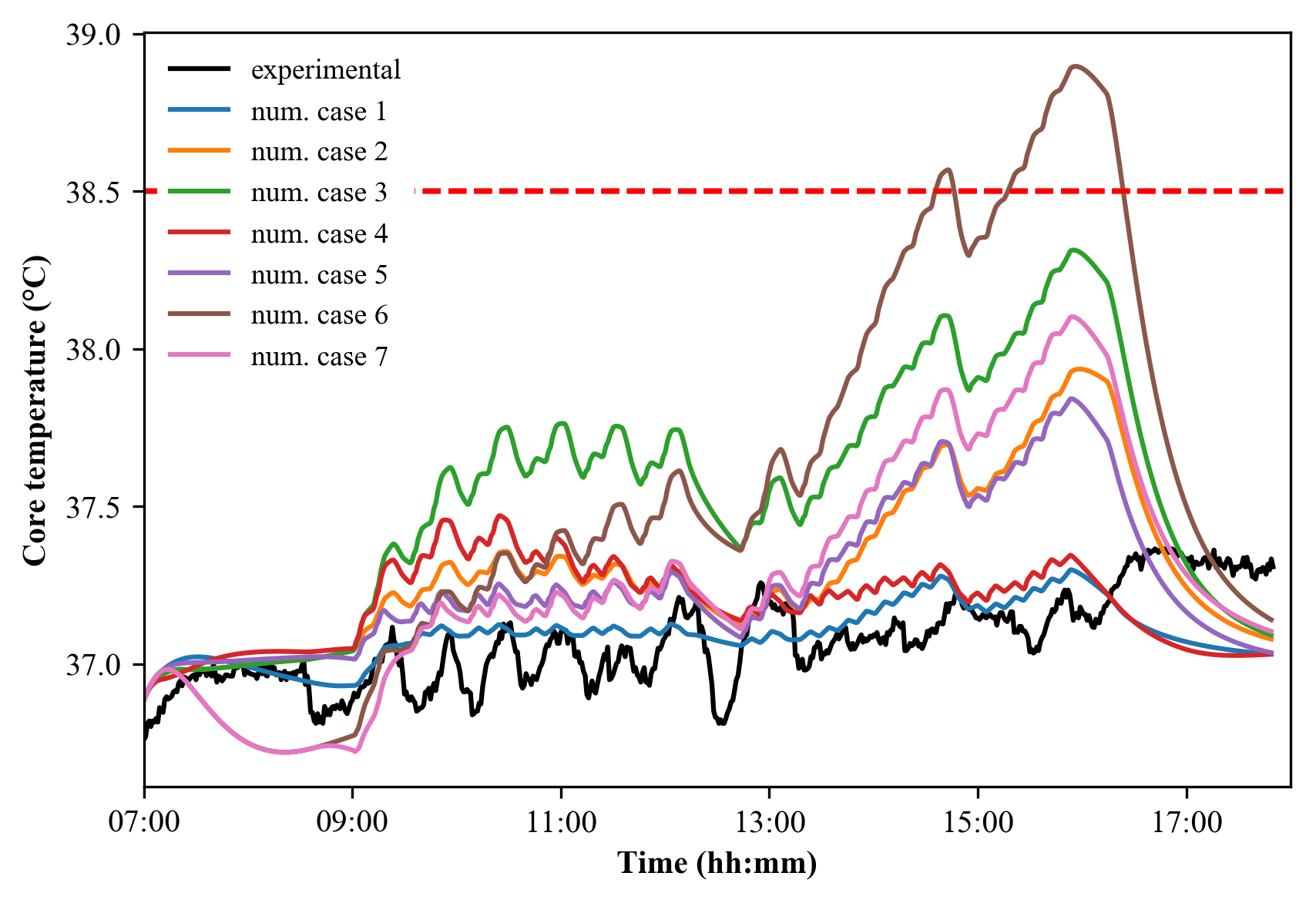 |

The results from the simulation of case 1 (which replicates the experiments) and case 2 (which adds solar exposure) are quite different (Figure s25) because the absorbed solar radiation in the latter case heats the body throughout the entire combat simulation protocol (Figure s26b). Note that heat gain by solar radiation is actually higher when the armour is not worn because the armour has a small absorptivity (0.3) compared to that of typical clothing (0.78, Table s7 and s9). Therefore, the absorbed radiation curve (Figure s26b) shows much higher absorptions (i.e. peaks in the absorbed radiation curve) when the participant takes the armour off to rest or during the first and last activities of the combat simulation protocol. Similar oscillations can be observed in the emitted radiation curve (in this case because the emissivity of the armour (0.22) is much lower than that of the undergarment (0.9), Table s9). Overall, there is a positive net heat gain by radiant exchange as the heat gain from the sun (i.e. absorbed radiation, Figure s26b) is higher than the heat loss to the sky (i.e. emitted radiation, Figure s26b). This is why the core temperature of the virtual subject in case 2 is up to almost 1°C higher than that of case 1 (where there is no solar exposure and the body exchanges heat by radiation to the walls rather than the colder sky in case 2), but still below the threshold of 38.5°C.

| **Figure s25.** Comparison between the predicted core temperature resulting from case 1 and case 2. While case 1 mimics the experimental study, case 2 has the presence of solar irradiation. The dashed red line is the core temperature threshold which, if it is exceeded, implies that the combat simulation protocol could not be completed under those conditions. |
| --- |
| 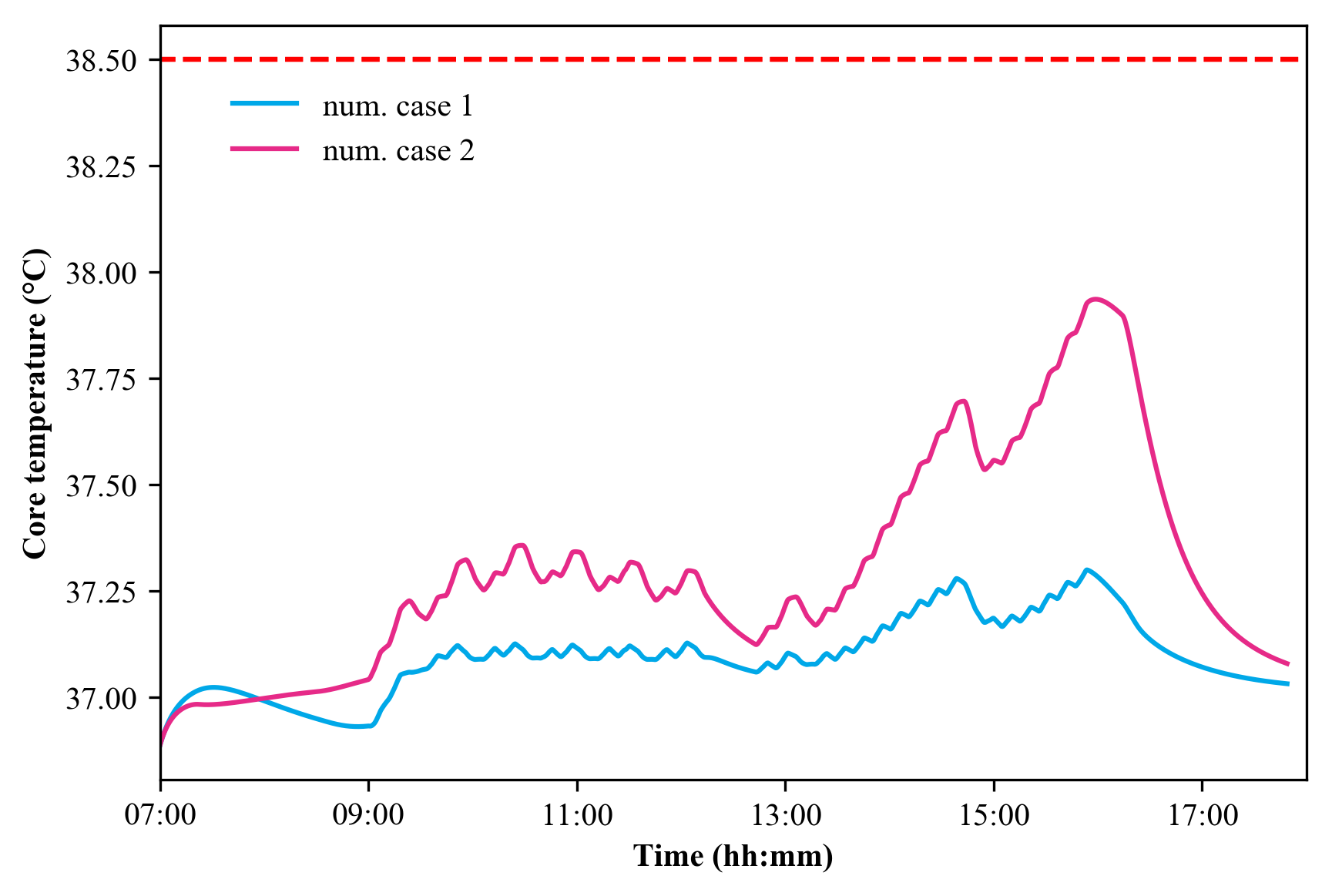 |

Figure s27 compares the core temperatures in case 2 and case 3, the latter with increased metabolic rates (to match high intensity fighting, Table s12). As expected, the increased heat production results in core temperatures in case 3 about 0.5°C higher than those in case 2, but still below the defined threshold (38.5°C). This means that it would be possible for warriors to complete the Late Bronze Age combat simulation protocol at high intensity under the sun and without wind (case 3, i.e. considering a constant air speed of 0.5 m/s). Furthermore, when considering typical wind conditions for the date and location in question (Figure s22), which is represented by case 4, there is a clear change in the core temperature variation over time (Figure s28) relative to the scenario without wind (case 3). The higher wind speeds in the afternoon (Figure s22) imply convective losses during that period that are almost 80% higher than those without wind (Figure s29a vs Figure s29b), resulting in up to 1 °C lower core temperatures in case 4 (Figure s28).

**Figure s26.** Average heat gain from different phenomena obtained from the simulation of case 1 (a) and case 2 (b).

| 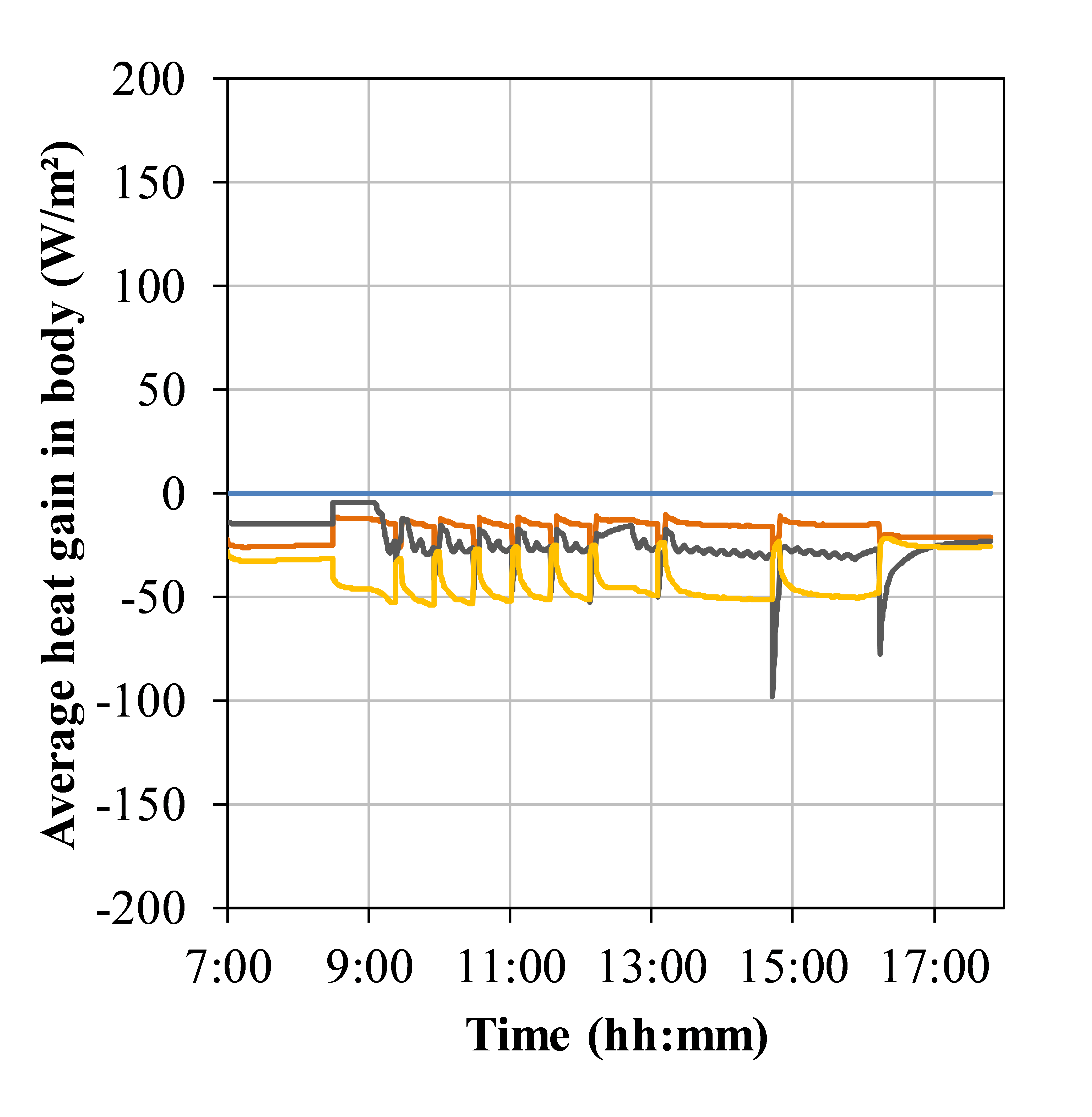 | 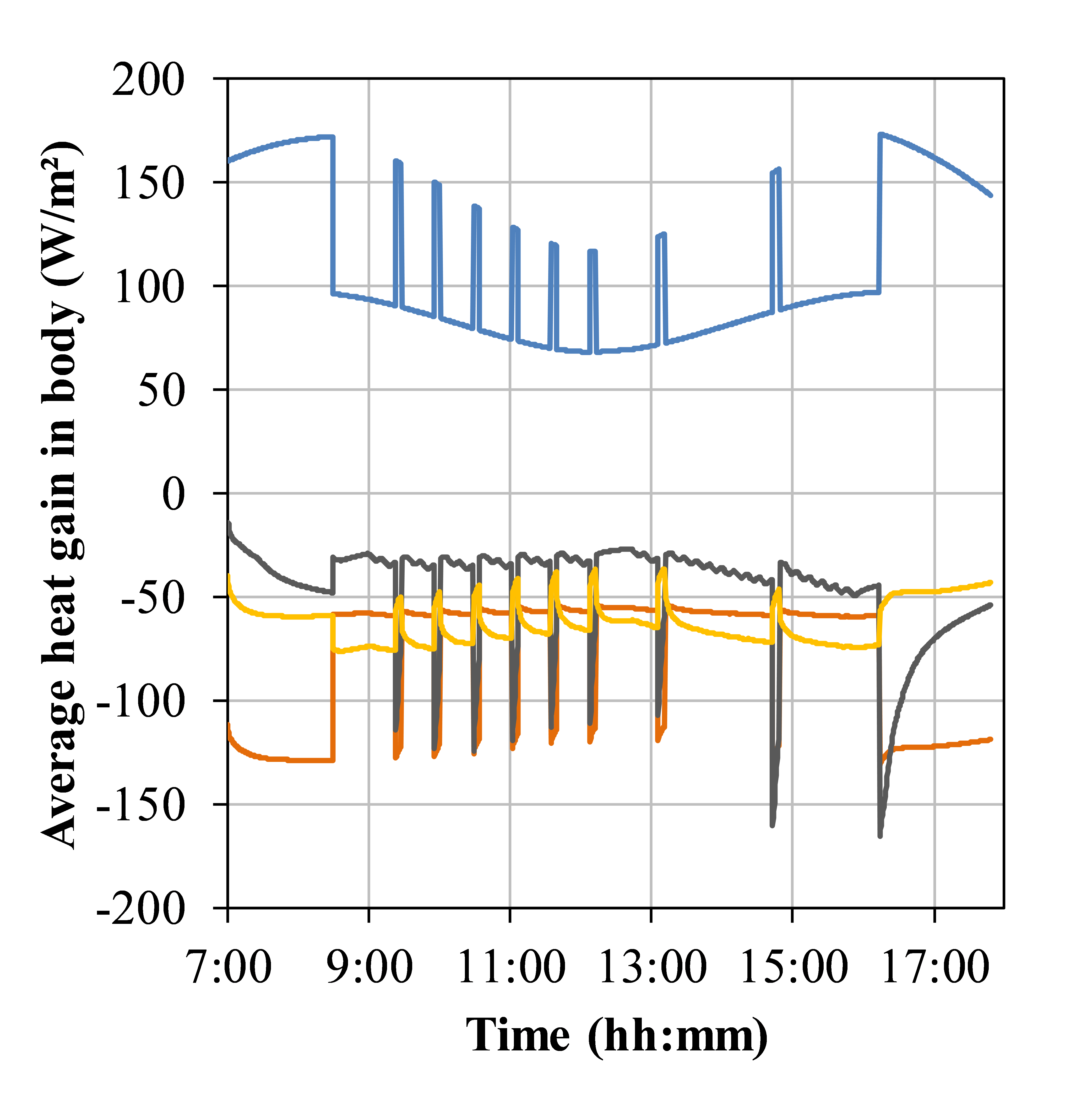 | 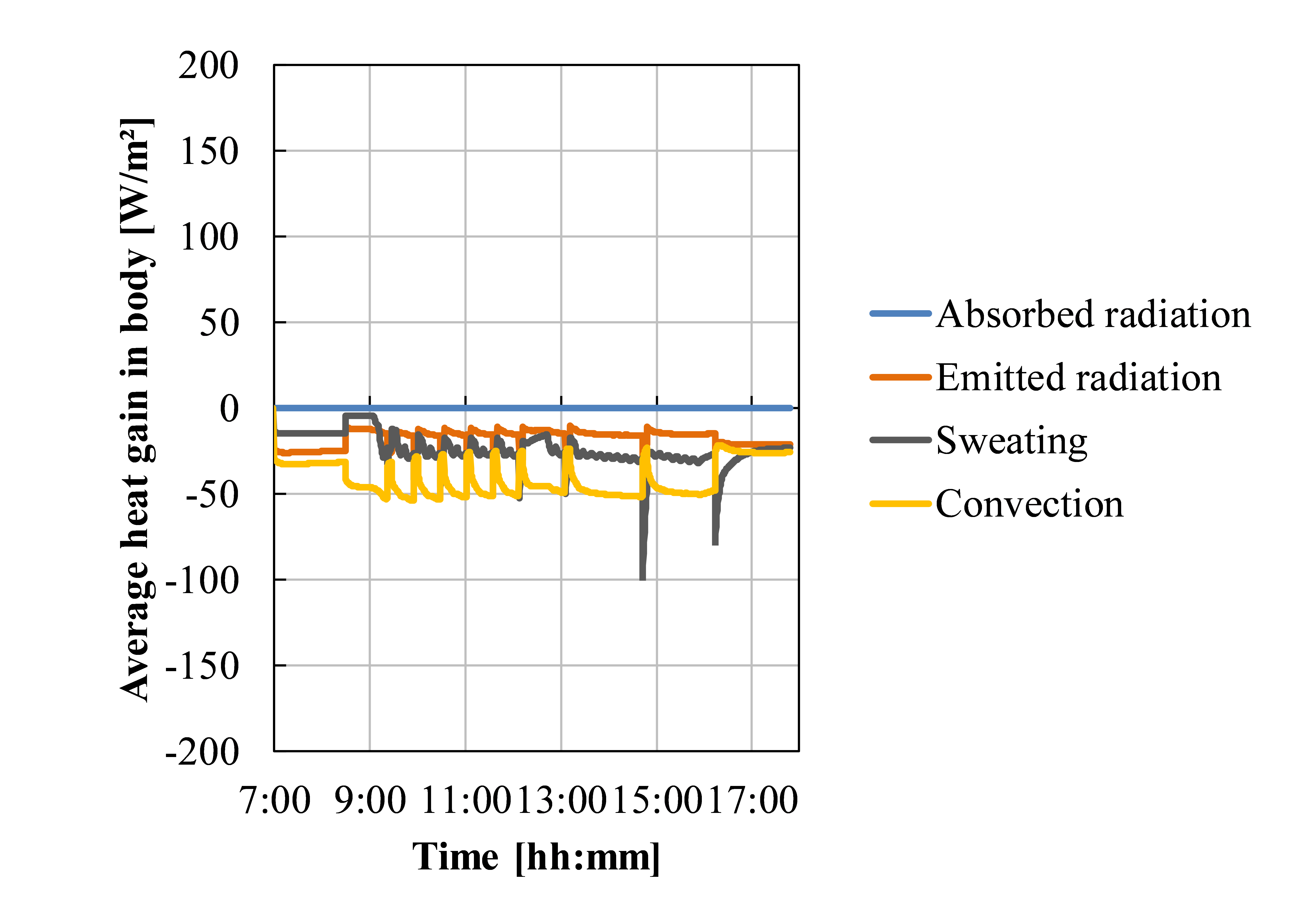 |
| --- | --- | --- |
| (a) | (b) |  |

**Figure s27.** Comparison between the predicted core temperatures obtained from case 2 and case 3 (the difference between them being that, in case 3, the metabolic rates are increased compared to case 2). The dashed red line is the core temperature threshold which, if it is exceeded, implies that the combat simulation protocol could not be completed under those conditions.


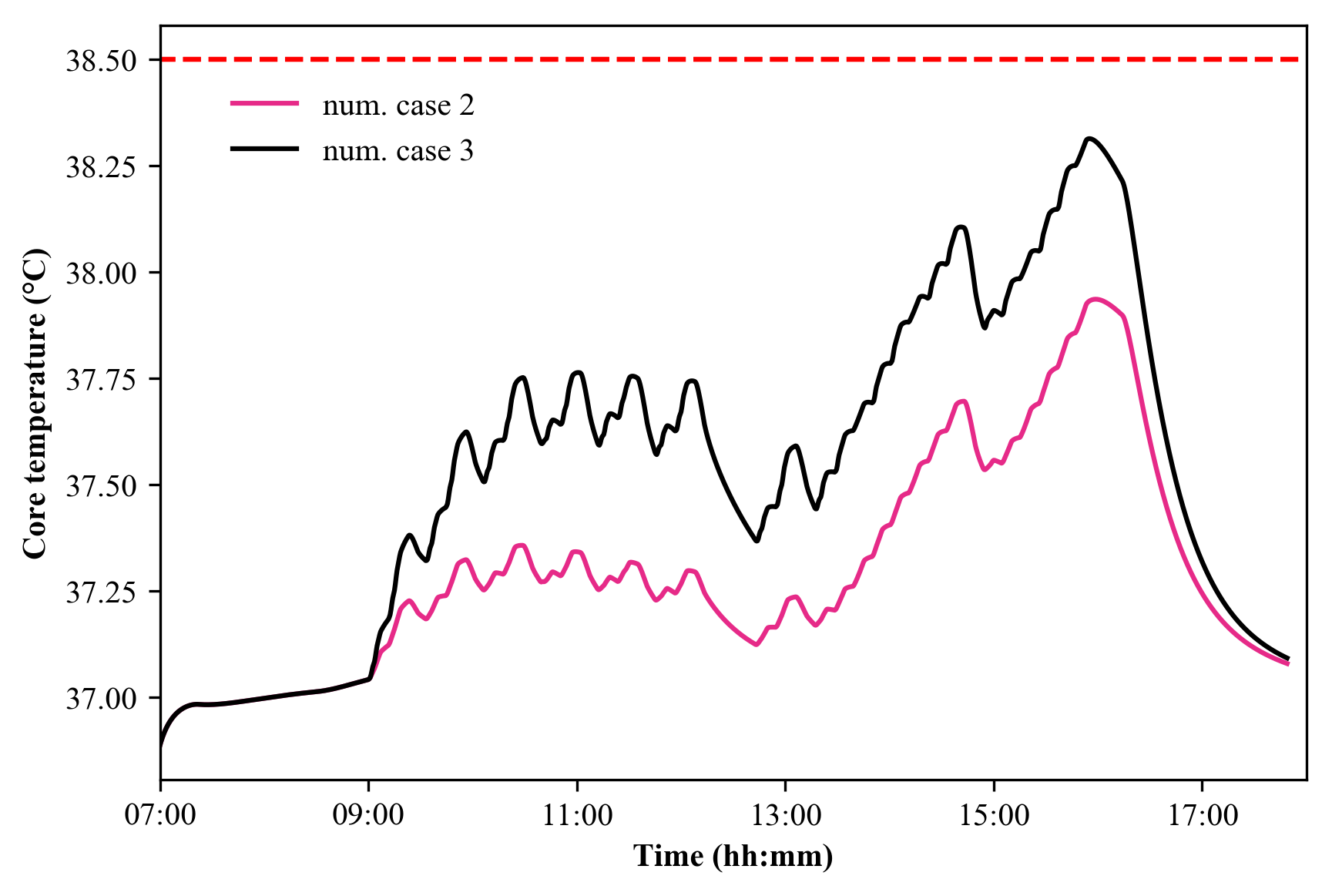


| **Figure s28.** Comparison between the predicted core temperatures obtained from case 3 and case 4. Contrary to case 3, the wind is present in case 4. The dashed red line is the core temperature threshold which, if it is exceeded, implies that the combat simulation protocol could not be completed under those conditions. |
| --- |
| 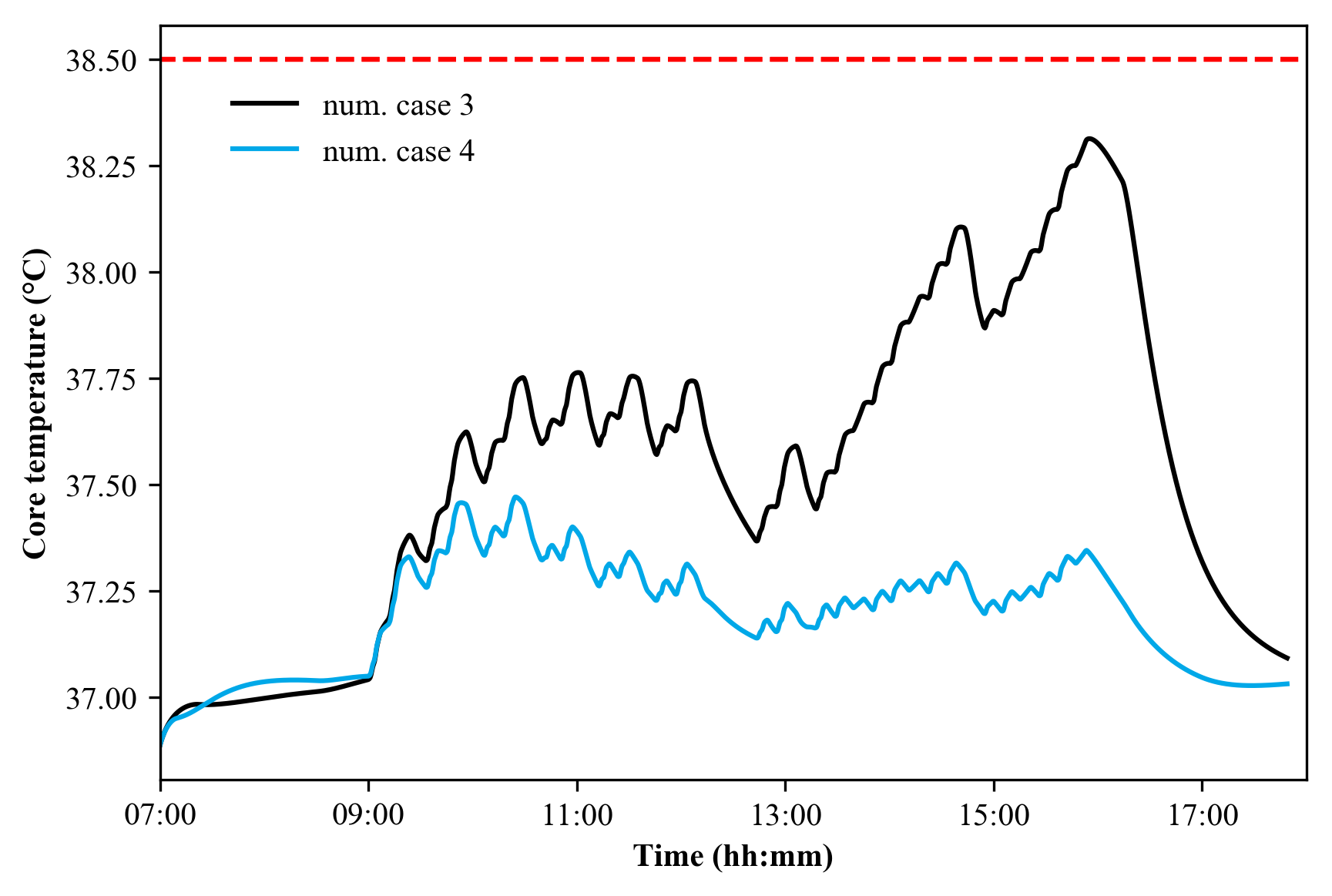 |

**Figure s29.** Average heat gain/loss from different phenomena obtained from the simulation of case 3 (a) and case 4 (b).

| 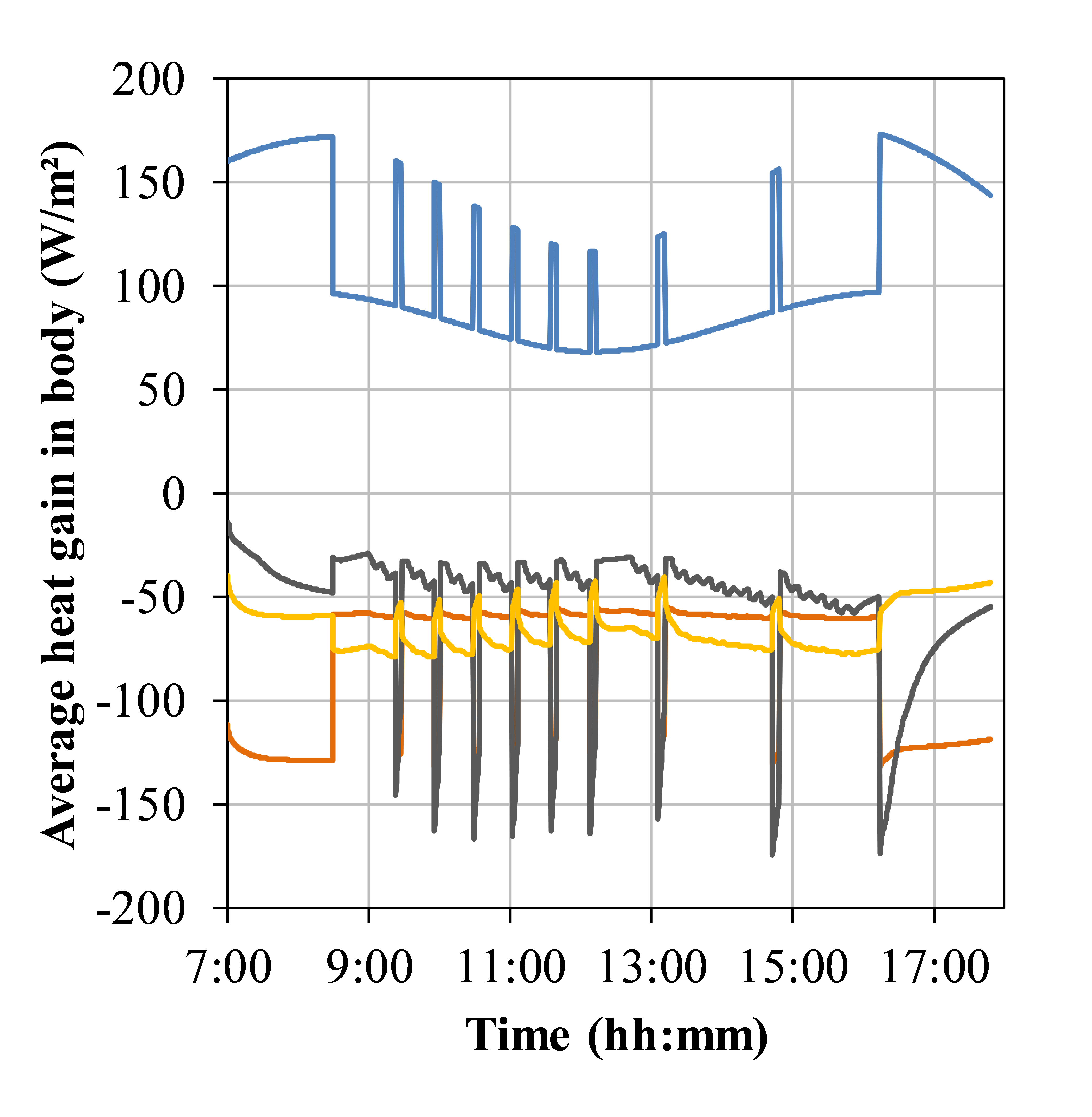 | 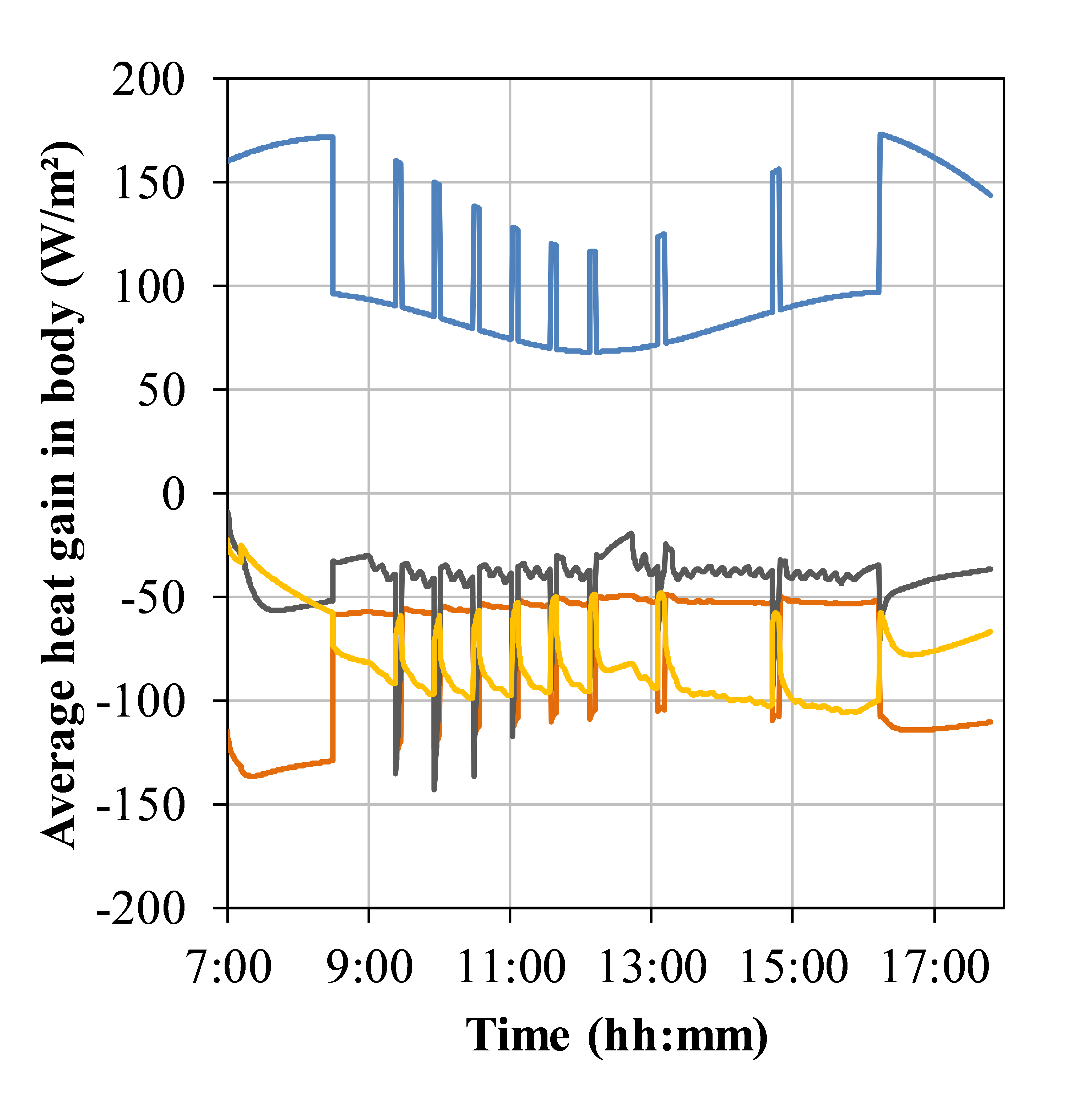 | 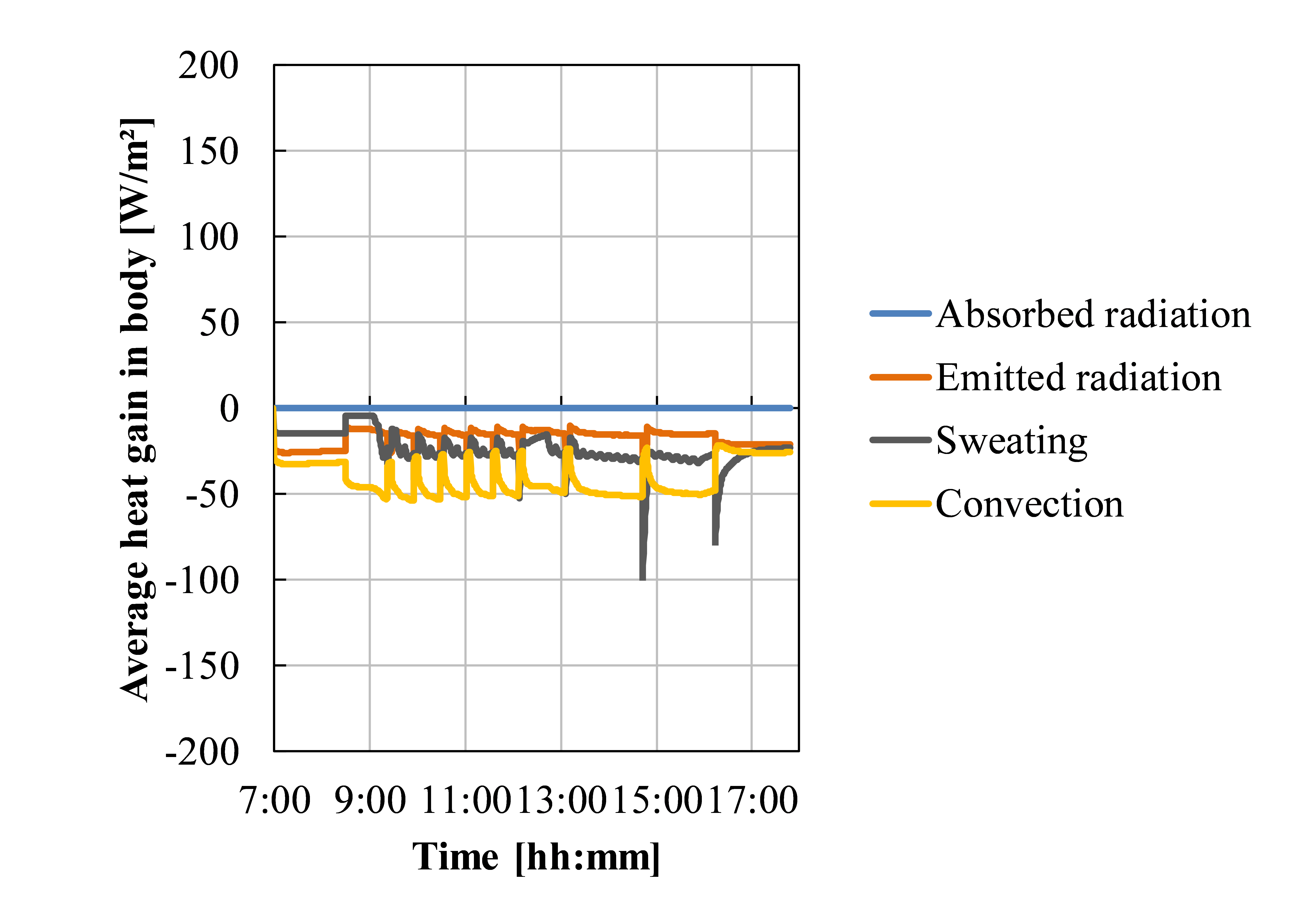 |
| --- | --- | --- |
| (a) | (b) |  |

In Figure s30a, we assess the effect of the considered air temperature on the core temperature variation over time. In the afternoon, the core temperatures are up to 0.5 °C higher for the typical air temperatures (15-30 °C; case 5) than for the experimental air temperatures (25-27 °C; case 4). This is due to higher air temperatures in the afternoon (Figure s22), which decrease the heat loss by convection (Figure s31a vs Figure s31b). However, since the minimum temperature during the morning in typical conditions is lower than that of the experimental conditions, the core temperature is lower in the former. Based on these results, namely that the core temperature is below 38.5 °C, we conclude that, for the tested conditions, the warrior could successfully complete the combat simulation protocol while wearing the armour.

**Figure s30.** Comparison between the predicted core temperatures obtained from case 4 and case 5 (a), showing the effect of typical air temperatures instead of those used experimentally on core temperature. (b) Case 6 represents conditions similar to case 5 but when wind is absent. The dashed red line is the core temperature threshold which, if it is exceeded, implies that the combat simulation protocol could not be completed under those conditions.

| 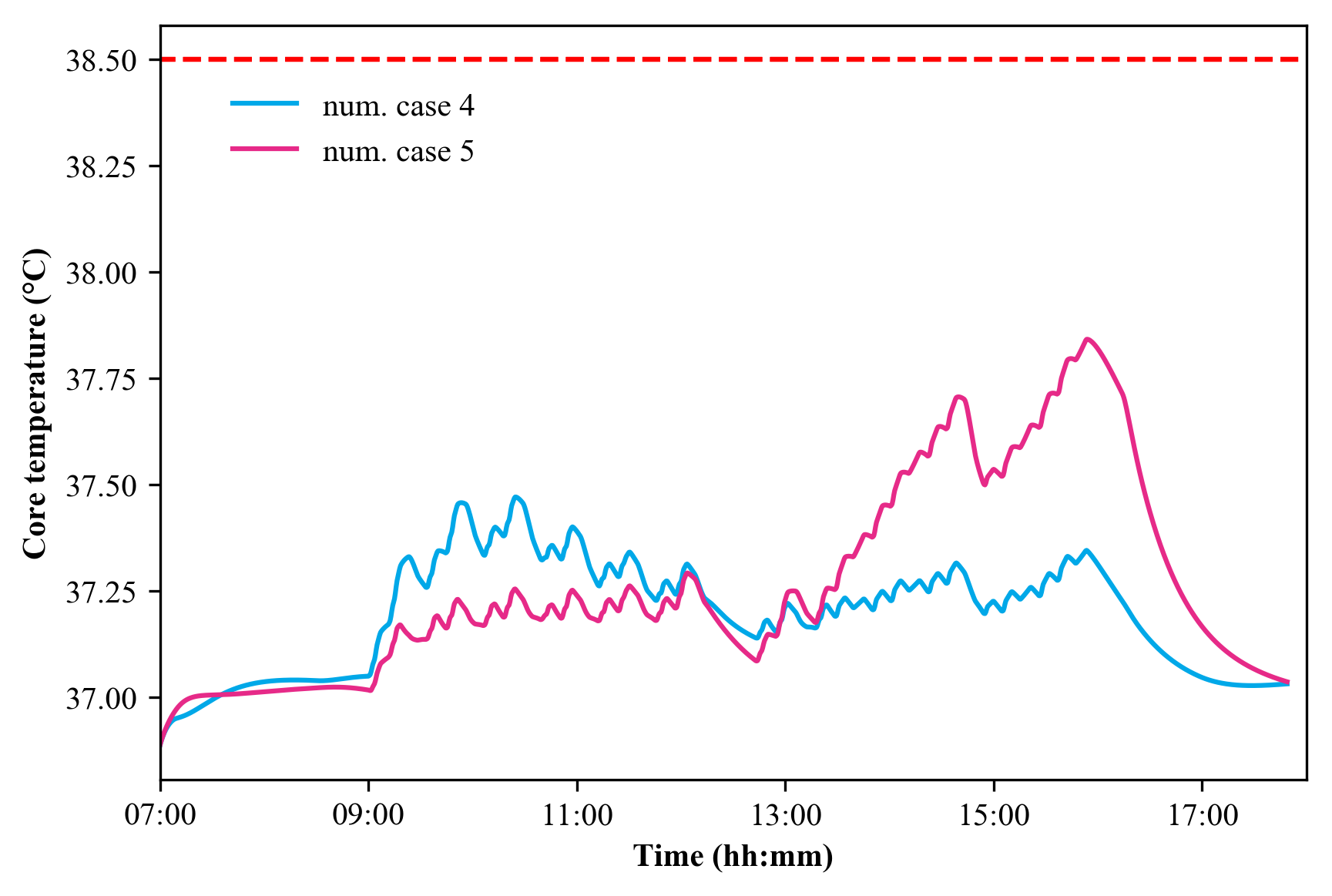 | 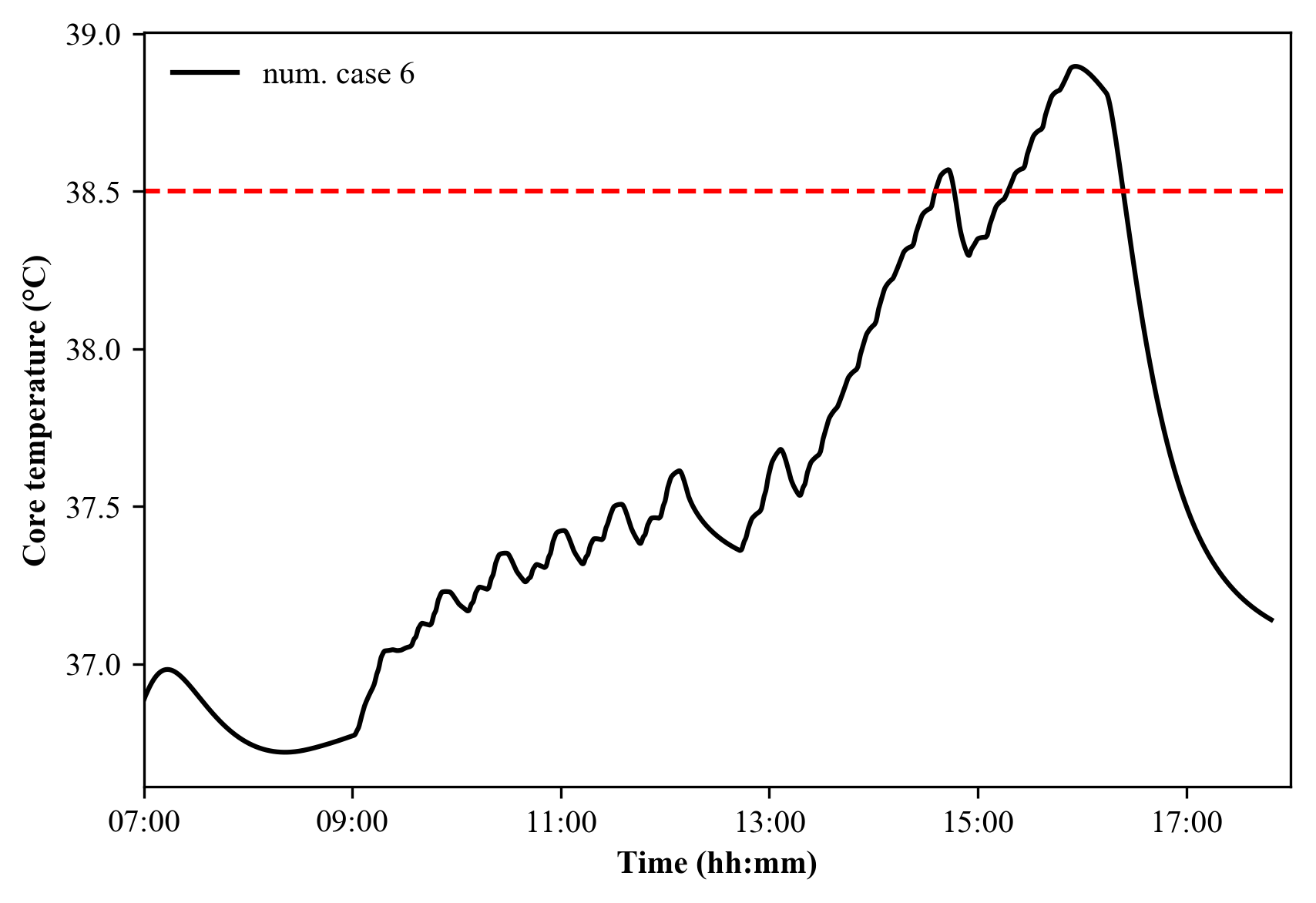 |
| --- | --- |
| (a) | (b) |

However, note that if the wind speeds were lower than the typical speeds, e.g. 0.5 m/s as assumed to match the experimental test conditions, the convective losses would be lower (Figure s31b), which would increase the core temperatures of the subjects. This is clearly seen in Figure s30b, where the core temperatures of the subject in case 6 (like case 5 but with fixed wind speed at 0.5 m/s) reaches 39 °C. In this case we would have to conclude against the armour being used in battle without the addition of a surcoat (case 7, see below). We believe, however, that such a low wind speed (i.e. 0.5 m/s) is not likely as the minimum daily wind speed recorded in the modern weather station data (Figure s20) is around 1 m/s. Therefore, if the assumed typical wind speeds are indeed a good representation of the actual wind speed at the time of the battle, then we must consider the use of the armour in battle as possible from the thermoregulation point of view, in all but extreme conditions.

**Figure s31.** Average heat gain/loss from different phenomena obtained from the simulation of case 5 (a) and case 6 (b).

| 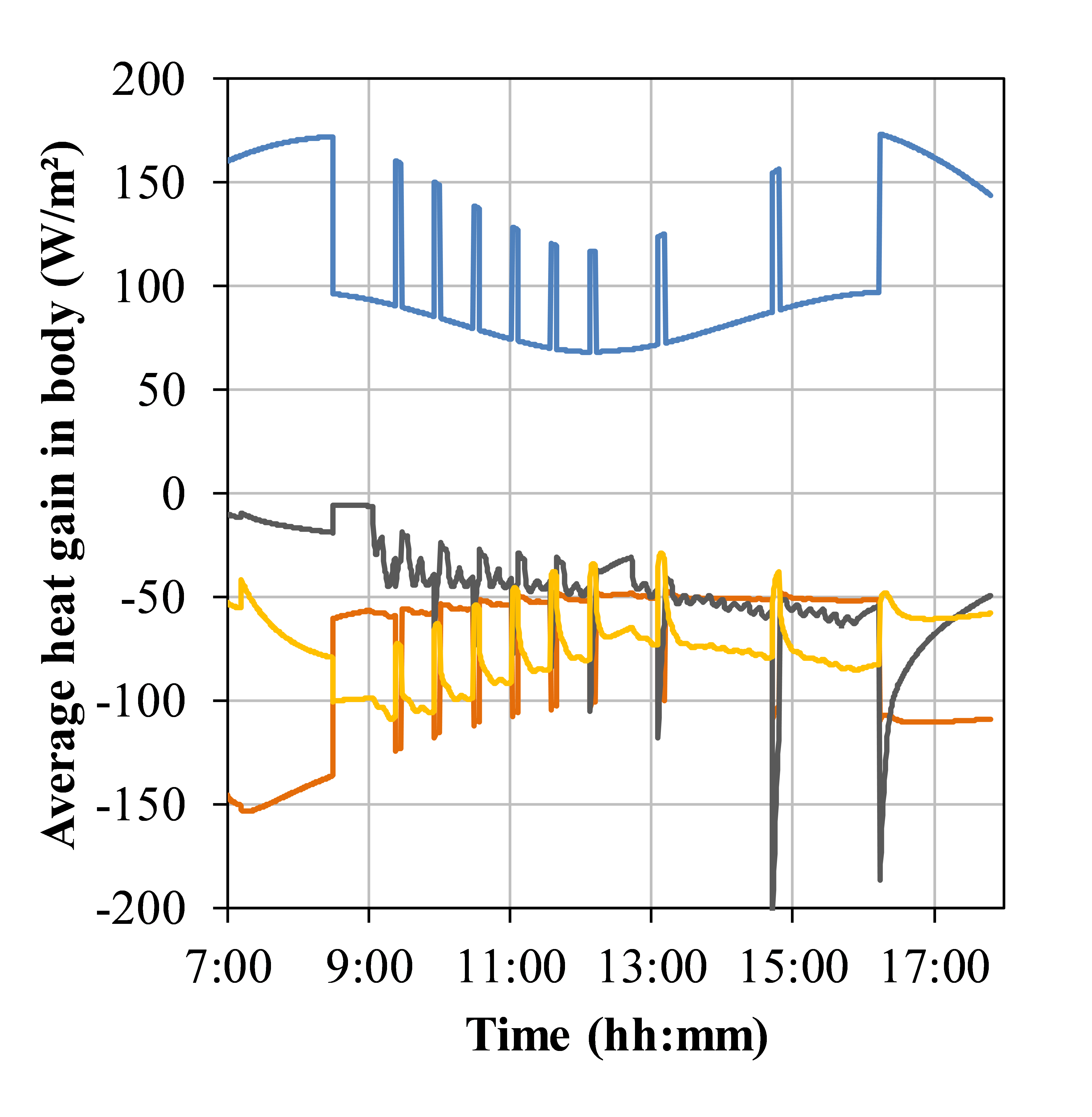 | 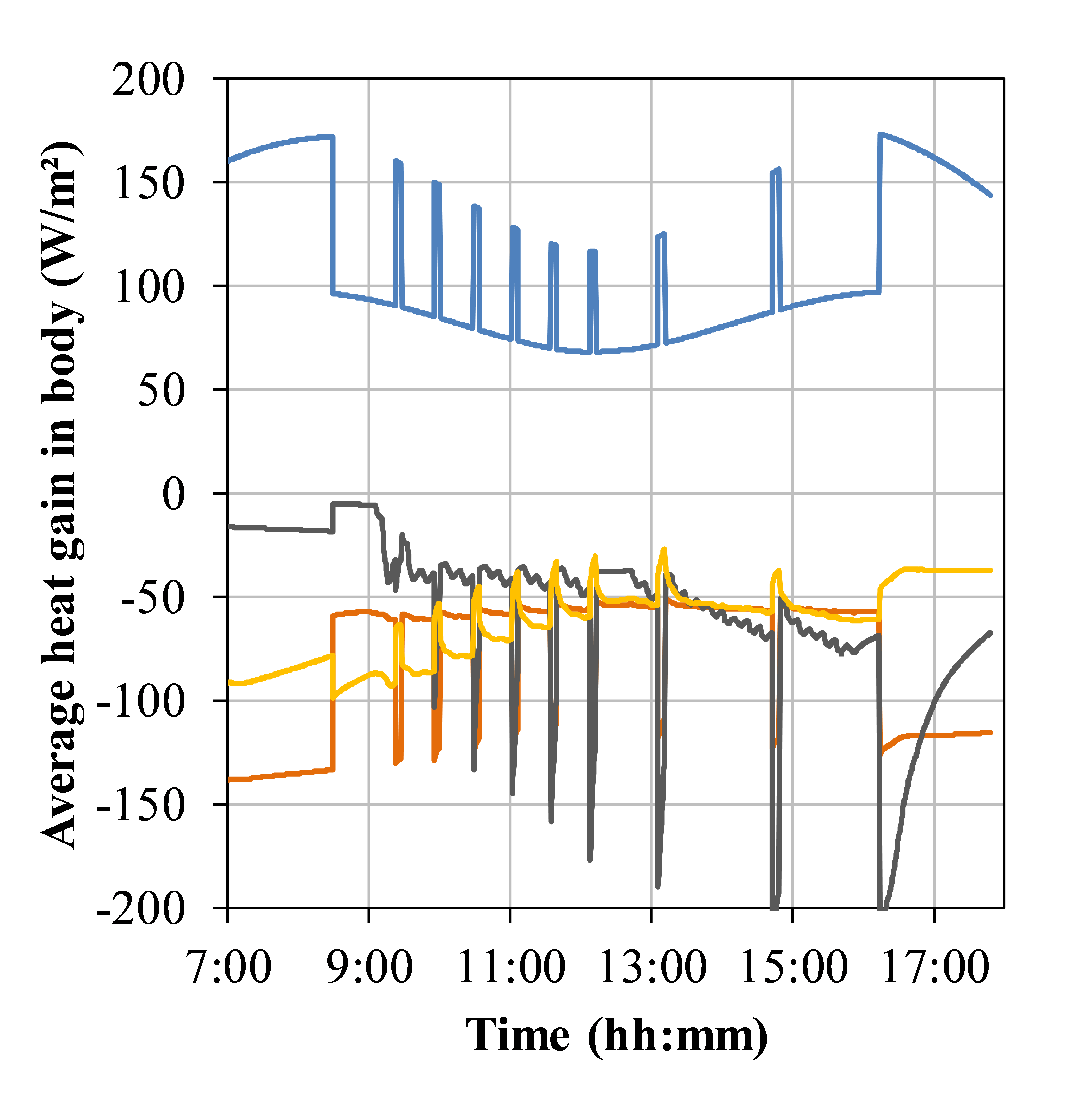 | 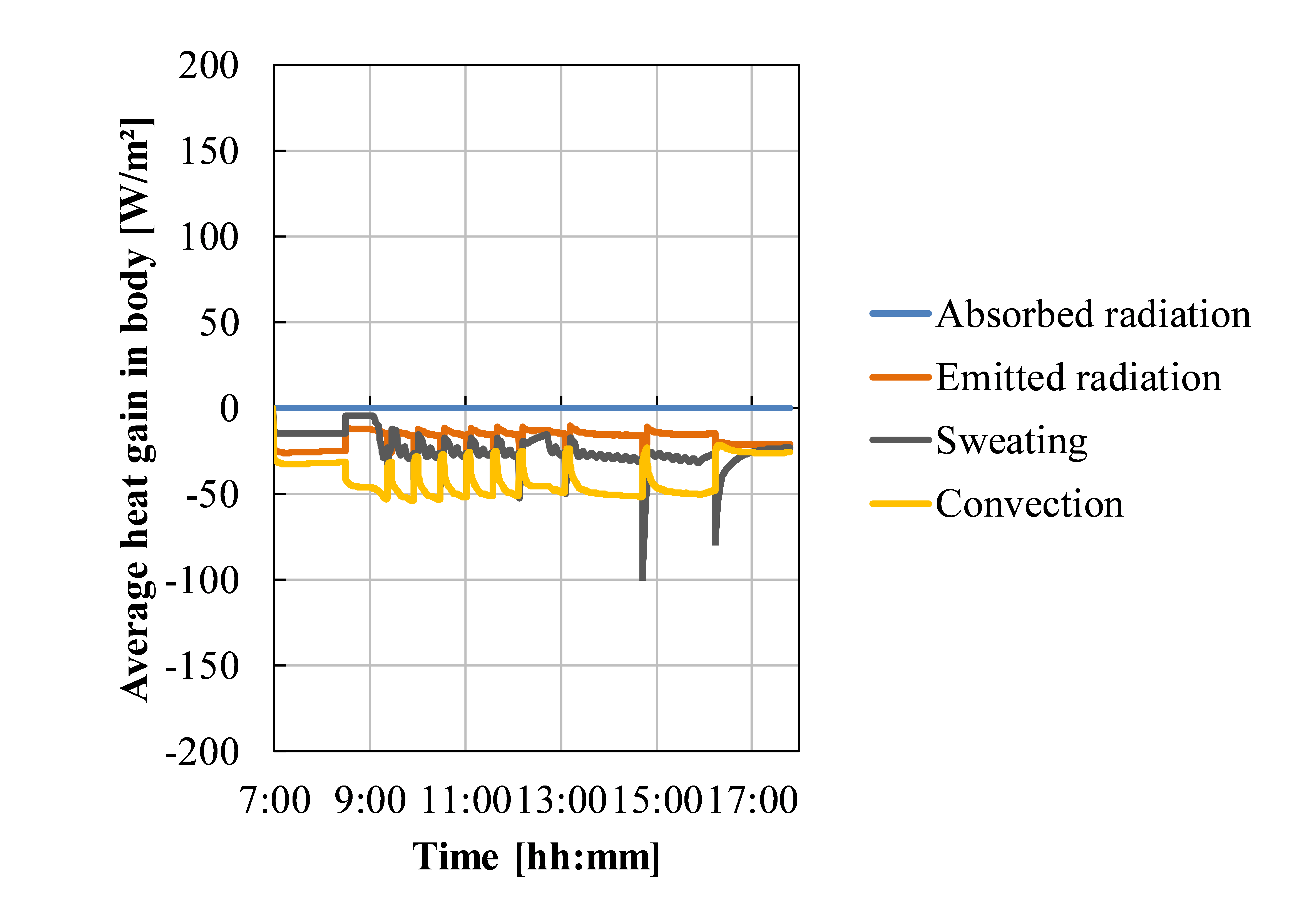 |
| --- | --- | --- |
| (a) | (b) |  |

Figure s32 shows the core temperatures for the simulation case leading to the higher thermal burden on the subjects (case 6, which resulted in core temperatures above the defined threshold, thus not compatible with combat simulation protocol completion) and the same conditions but with addition of a white surcoat over the armour (case 7). We see that the core temperature is almost 0.75 °C lower when the white surcoat is added over the armour. The surcoat greatly increases the heat loss by long-wave radiation because of its higher emissivity (compared to that of the armour), and decreases the convective losses because of the lower temperature of the surcoat (Figure s33 versus Figure s31b). Overall, there is an increase in the dry heat losses (long-wave radiant + convection) when wearing a surcoat, which lowers the core temperature of the subjects (case 7) below the defined threshold of 38.5 °C. For that reason, the use of a white surcoat over the armour would allow the warrior to complete the combat simulation protocol.

| **Figure s32.** Comparison between the predicted core temperatures obtained from case 6 and case 7. The dashed red line is the core temperature threshold which, if it is exceeded, implies that the combat simulation protocol could not be completed under those conditions. |
| --- |
| 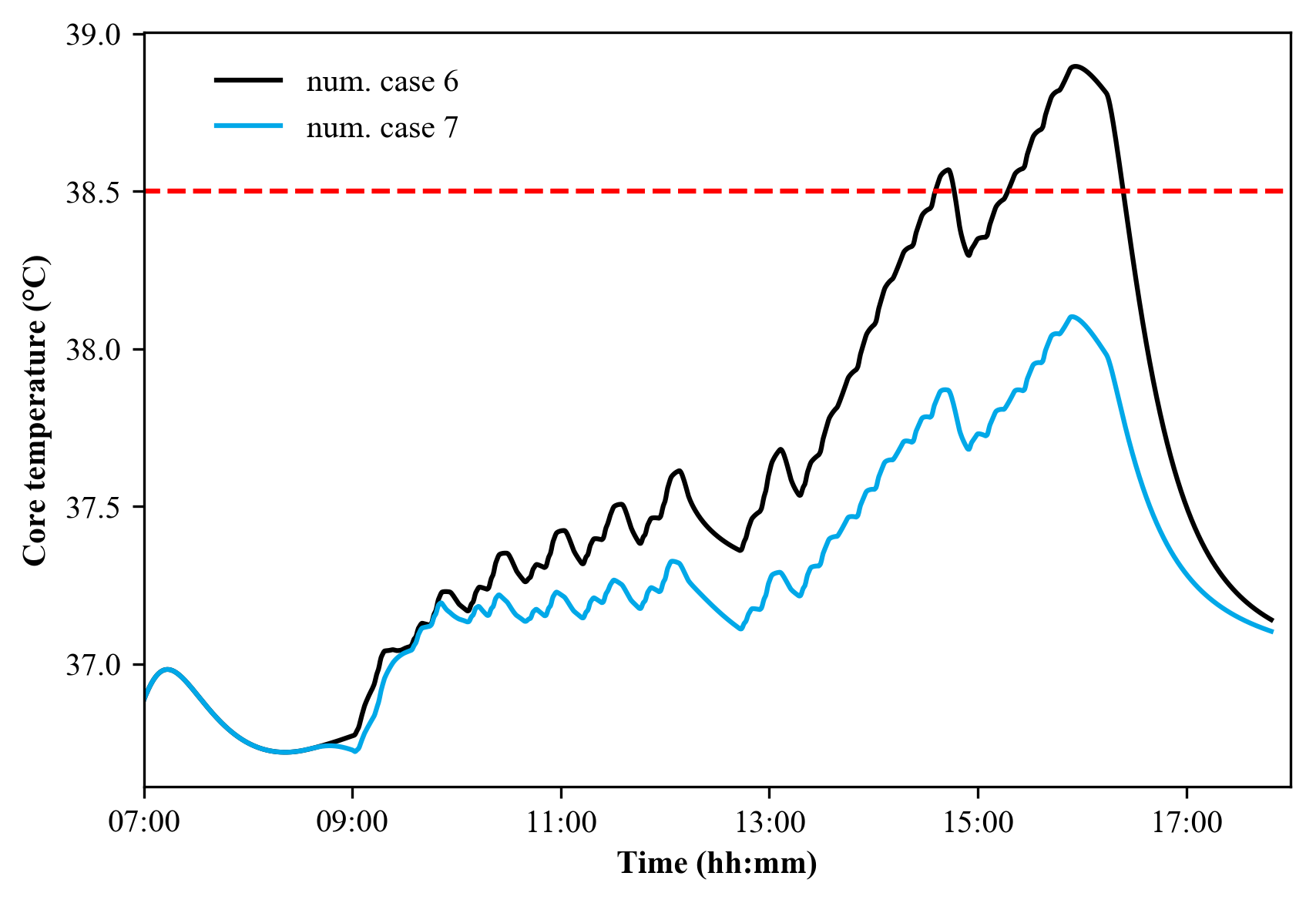 |

**Figure s33.** Average heat gain/loss from different phenomena obtained from the case 7 simulation.


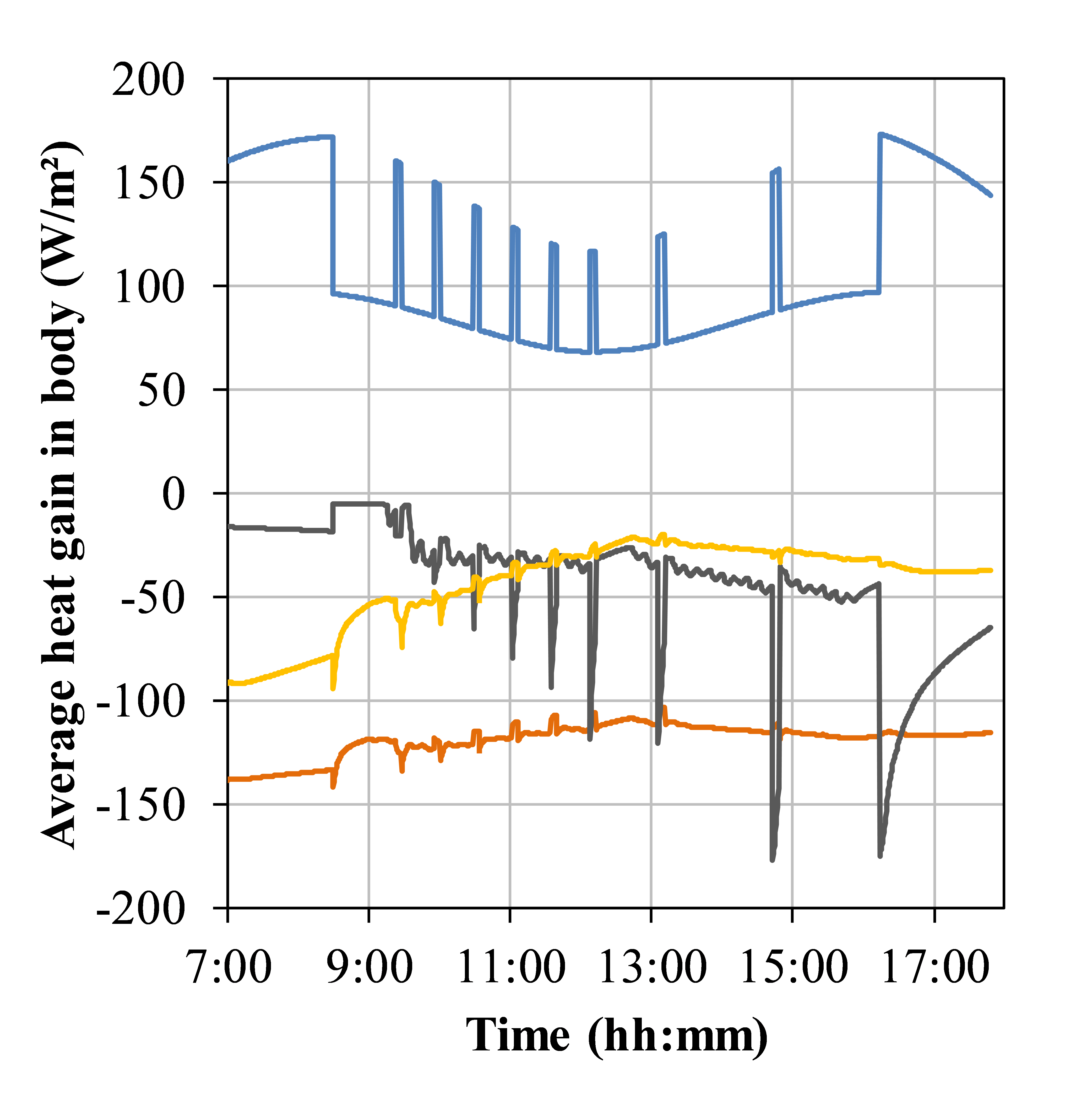

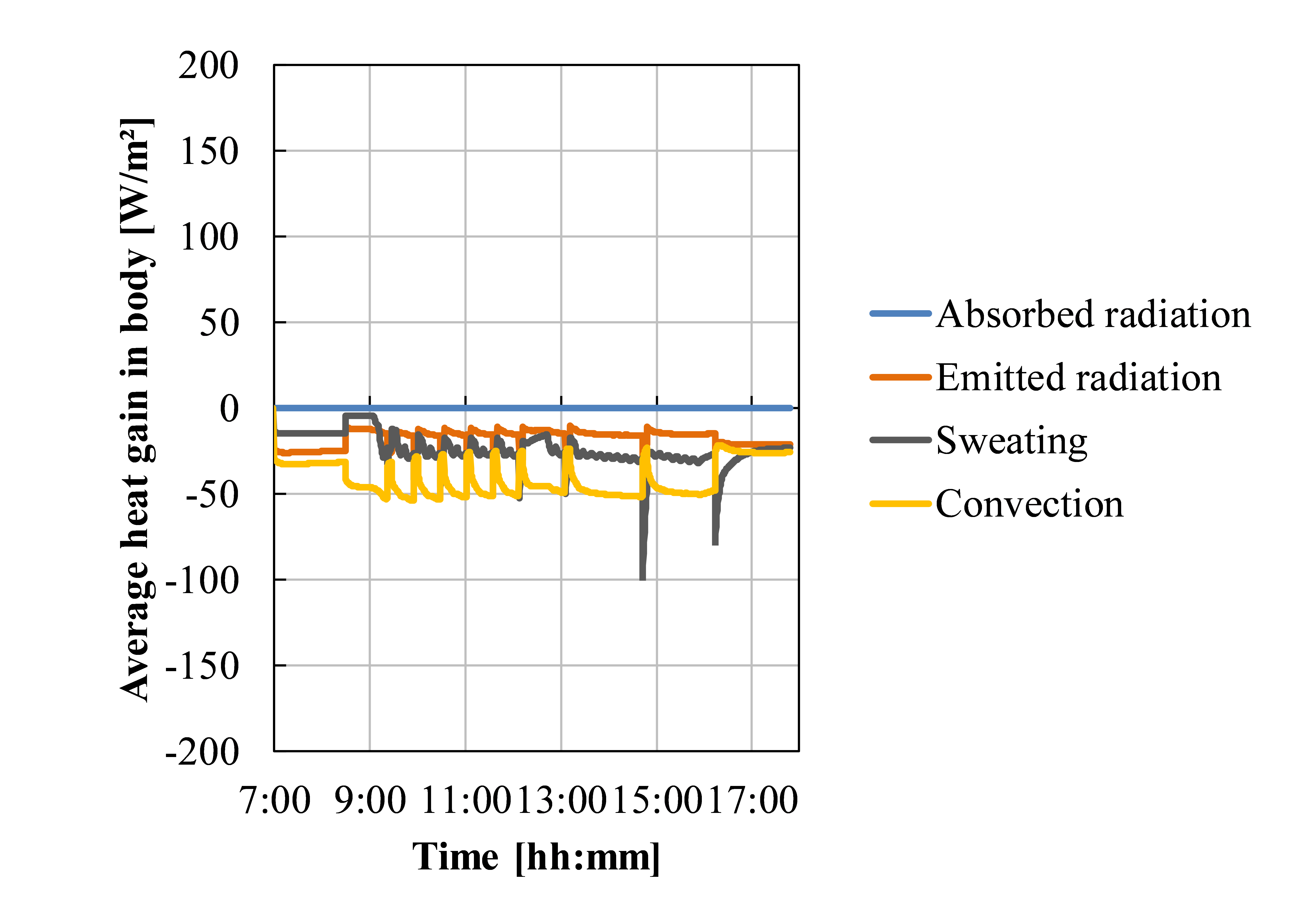


### Section 5.6. Conclusion of numerical study

We considered several simulation cases to investigate the influence of multiple parameters (air temperature, wind, solar exposure, metabolic rate, and use of a white surcoat over the armour) on the variation of the core temperature of individuals following the combat simulation protocol while wearing armour, and whether, in each case, they could successfully complete the Late Bronze Age combat simulation protocol. The simulations indicated that, for all but one of the tested conditions, the combat simulation protocol could be completed when using the armour. The exception (case 6) was a thermal scenario with unrealistic low wind speed and high temperatures that was considered highly improbable. Accordingly, these simulations support the hypothesis that the Dendra armour could have been worn to battle in the Late Bronze Age, as surely indicated by the large number of suits recorded in the Linear B tablets (see Section 1 above).

# REFERENCES

1 Åstrom, P. *Studies in Mediterranean Archaeology, IV. The Cuirass Tomb and other Finds at Dendra. Part I: The Chamber Tombs. In collaboration with Verdelis N. M., Gejvall N. -G., and Hjelmqvist H.*, (Bloms Boktryckeri A.-B., 1977).

2 Verdelis, N. M. Neue Funde von Dendra, Mitteilungen des Deutschen Archäologischen Institutes. *Athenische Abteilung* **82**, 1-53 (1967).

3 Mödlinger, M. *Protecting the body in war and combat: metal body armour in Bronze Age Europe. Oriental and European Archaeology, Volume 6*. (Austrian Academy of Sciences Press, 2017).

4 Molloy, B. in *Talanta XLIV (2012)* (ed A. Papadopoulos) 273-294 (Dutch Archaeological and Historical Society, 2013).

5 Obert, J. & Molloy, B. in *Brill’s Companion to Bronze Age Warfare in the Aegean* (eds L. Kvapil & K. Shelton) 245-267 (Brill, 2023).

6 Persson, A. W. *New tombs at Dendra near Midea*. (Gleerup, 1942).

7 Andrikou, E. New evidence on Mycenaean bronze corselets from Thebes in Boeotia and the Bronze Age sequence of corselets in Greece and Europe. Between the Aegean and the Baltic Seas. Prehistory across Borders. Proceedings of the International Conference Bronze and Early Iron Age Interconnections and Contemporary Developments between the Aegean and the Regions of the Balkan Peninsula. Edited by: Galanaki I., Tomas H., Galanakis Y., and Laffineur R. Central and Northern Europe University of Zagreb, 11–14 April 2005. *Aeaaeum* **27**, 401-409 (2007).

8 Savignoni, L. Scavi dela missione italiana a Phaestos 1902-1903: raporto preliminare. *Monumenti Antichi* **14**, 537-666 (fig. 522) (1904).

9 McDonald, W. A. & Wilkie, N. *Excavations at Nichoria in Southwest Greece, Volume II, The Bronze Age Occupation, 253, Table 5-8, 333, fig. 5.35, 853, pl. 5.113*. (University of Minnesota Press, 1992).

10 Georganas, I. & Kvapil, L. in *Brill’s Companion to Bronze Age Warfare in the Aegean* (eds L. Kvapil & K. Shelton) 104 (Brill, 2023).

11 Yalouris, N. Mykenische Bronzeschutzwaffen. *Mitteilungen des Deutschen Archäologischen Instituts. Athenische Abteilung* **75**, 42-76 (1960).

12 Tsountas, C. Excavations of graves in Mycenae (Ανασκαφαί τάφων εν Μυκήναις). *Archaiologike Ephemeris*, 119-180 (1888).

13 Stubbings, F. H. Mycenae 1939-1953, Part VII. A Bronze Founder’s Hoard. *The Annual of the British School at Athens* **49**, 292-296 (1954).

14 Hutchinson, R. W. A Late Minoan Tomb at Knossos. *The Annual of the British School at Athens* **51**, 68-73 (1956).

15 Ventris, M. & Chadwick, J. *Documents in Mycenaean Greek*. 374-380 (Cambridge University Press, 1959).

16 Chadwick, J. *The Mycenaean World*. (Cambridge University Press, 1976).

17 Driessen, J. in *Mykenaïka. Actes du IXe colloque international sur les textes mycéniens et égéens* (ed J.-P. Olivier) 197-214 (De Boccard, 1992).

18 Shelmerdine, C. in *Brill’s Companion to Bronze Age Warfare in the Aegean* (eds L. Kvapil & K. Shelton) 135 (Brill, 2023).

19 Godart, L., Killen, J. T. & Olivier, J.-P. Ausgrabungen in Tiryns 1981: Eighteen More Fragments of Linear B Tablet from Tiryns. *Archäologische Anzeiger*, 413-426 (1983).

20 Berger, D. *et al.* Isotope systematics and chemical composition of tin ingots from Mochlos (Crete) and other Late Bronze Age sites in the eastern Mediterranean Sea: An ultimate key to tin provenance? *PLoS One* **14**, e0218326 (2019). <https://doi.org:10.1371/journal.pone.0218326>

21 Bass, G. F., Pulak, C., Collon, D. & Weinstein, J. The Bronze Age Shipwreck at Ulu Burun (Kas): 1986 Campaign. *American Journal of Archaeology* **93**, 1-29 (1989).

22 Hauptmann, A., Maddin, R. & Prange, R. *On the Structure and Composition of Copper and Tin Ingots Excavated from the Shipwreck of Uluburun. Bulletin of the American Schools of Oriental Research, No. 328*. (University of Chicago Press, 2002).

23 Wace, A. J. B. & Stubbings, F. H. *A companion to Homer*. 506-510 (Macmillan, 1962).

24 Aldrete, G. S., Bartell, S. & Aldrete, A. *Reconstructing ancient linen body armour: Unraveling the Linothorax mystery*. (John Hopkins University Press, 2013).

25 Mylonas, G. E. *Mycenae rich in gold*. (Ekdotike Athenon, 1983).

26 Lang, M. L. *The Palace of Nestor in Messenia II: The Frescoes*. (Princeton University Press, 1969).

27 Rodenwaldt, G. *Tiryns II. Die Fresken des Palastes, nos. 113-93, 96-137, Taf. XII*. (Eleutheroudakis and Barth, 1912).

28 Doumas, C. *The wall-painting of Thera*. (Kapon, 1992).

29 Snodgrass, A. M. *Early Greek armour and weapons: from the end of the bronze age to 600 B.C.*, (University Press, 1964).

30 Dickinson, O. *The Aegean from Bronze Age to Iron Age*. (Routledge, 2006).

31 Morris, I. Circulation, deposition and the formation of the Greek Iron Age. *Man* **24**, 502-519 (1989).

32 Catling, H. W. Bronze cut-and-thrust swords in the Eastern Mediterranean. *Proceedings of the Prehistoric Society* **22**, 102-125 (1957).

33 Molloy, B. in *SOMA 2002: Symposium on Mediterranean Archaeology. Proceedings of the Sixth Annual, Meeting of Postgraduate Researchers, University of Glasgow, Department of Archaeology, 15-17 February, 2002* (eds A. Brysbaert *et al.*) S1142 (BAR Publishing, 2003).

34 Courbin, P. Une tombe géometrique d'Argos. *Bulletin de Correspondance Hellénique* **81**, 322-386 (1957).

35 Reichel, W. *Über homerische waffen: archäologische untersuchungen*. (A. Holder, 1894).

36 Lorimer, H. *Homer and the monuments*. (Macmillan, 1950).

37 Hall, J. M. *A history of the Archaic Greek World, ca. 1200-479 BCE*. (Blackwell Publishing, 2007).

38 Drews, R. *The end of the Bronze Age: Changes in warfare and the catastrophe ca. 1200 B.C.*, (Princeton University Press, 1993).

39 Litthauer, M. A. The military use of the chariot in the Aegean in the Late Bronze Age. *American Journal of Archaeology* **76**, 145-157 (1972).

40 Travis, H. & Travis, J. *Roman helmets*. (Amberley Publishing, 2014).

41 Phang, S. E., Spence, I., Kelly, D. & Londey, P. in *Conflict in Ancient Greece and Rome: the definitive political, social, and military encyclopedia* Vol. 1 3-16 (Bloomsbery Publishing, 2016).

42 Wardle, D. E. H. in *Problems in Greek prehistory. Papers presented at the Centenary Conference of the British School of Archaeology at Athens, Manchester* (eds E. B. French & K. A. Wardle) (Bristol Classical Press, 1986).

43 Jablonka, P. in *The Oxford Handbook of the Bronze Age Aegean* (ed E. H. Cline) Ch. 63, 1070-1085 (Oxford University Press, Inc., 2010).

44 Meier-Brügger, M. in *Ancient Greece: From the Mycenaean Palaces to the Age of Homer* (eds S. Deger-Jalkotzy & I. S. Lemos) Ch. 21, 417-426 (Edinburgh University Press Ltd, 2006).

45 Dickinson, O. in *The Aegean from Bronze Age to Iron Age: Continuity and change between the twelfth and eighth centuries bc* (ed O. Dickinson) Ch. 2, 24-57 (Routledge, 2006).

46 Mee, C. in *The Cambridge Companion to the Aegean Bronze Age* (ed C. W. Shelmerdine) Ch. 14, 362-386 (Cambridge University Press, 2008).

47 Cline, E. H. *The Oxford handbook of the Bronze Age*. (Oxford University Press, 2012).

48 van Wees, H. Kings in combat: battles and heroes in the Iliad. *Classical Quarterly* **38**, 1-24 (1988).

49 Vermeule, E. D. T. in *Troy and the Trojan War: A Symposium Held at Bryn Mawr College, October 1984* (ed M. J. Mellink) (Bryn Mawr Commentaries, 1986).

50 Molloy, B. Swords and swordsmanship in the Aegean Bronze Age. *American Journal of Archaeology* **114**, 403-428 (2010).

51 Müller-Karpe, H. Zur spätbronzezeitlichen Bewaffnung in Mitteleuropa und Griechenland (On Late Bronze Age armament in Central Europe and Greece). *Germania* **40** (1962).

52 Maronitis, D. *Homer's Iliad. Rhapsodies 1-24 [Translation in Modern Greek]*. (Agra Publishing, 2012).

53 Flouris, A. D., Petmezas, S. & Asimoglou, P. *Analysis of combat fighting in Homer's Iliad. figshare. Dataset.* [*https://doi.org/10.6084/m9.figshare.12961499.v1*](https://doi.org/10.6084/m9.figshare.12961499.v1). (2020).

54 Kraft, J. C., Rapp, G., Kayan, I. & Luce, J. V. Harbor areas at ancient Troy: Sedimentology and geomorphology complement Homer’s Iliad. *Geology* **31**, 163-166 (2003).

55 Hamilton, H. C. & Falconer, W. *The Geography of Strabo. Literally translated, with notes, in three volumes. Accessed freely at:* [*http://www.perseus.tufts.edu/hopper/text?doc=urn:cts:greekLit:tlg0099.tlg001.perseus-eng2:1*](http://www.perseus.tufts.edu/hopper/text?doc=urn:cts:greekLit:tlg0099.tlg001.perseus-eng2:1)*. Accessed on 23/1/2020*. (George Bell & Sons, 1903).

56 Luce, J. V. *Celebrating Homer’s landscapes*. 260 (Yale University Press, 1998).

57 Flouris, A. D. *3D map of Troy in the time of Homer’s Iliad:* [*https://doi.org/10.6084/m9.figshare.12961463.v1*](https://doi.org/10.6084/m9.figshare.12961463.v1). (2020).

58 Mylonas, G. E. Priam's Troy and the date of its fall. *Hesperia: The Journal of the American School of Classical Studies at Athens* **33**, 352-380 (1964).

59 Korfmann, M. in *Troy and the Trojan War: A Symposium Held at Bryn Mawr College, October 1984* (ed M. J. Mellink) (Bryn Mawr Commentaries, 1986).

60 Wood, M. *In search of the Trojan War*. (Penguin Books USA Inc., 1985).

61 Godley, A. D. *Herodotus, The Histories. Book 2, Chapters 53 and 145. Accessed freely at:* [*http://data.perseus.org/citations/urn:cts:greekLit:tlg0016.tlg001.perseus-eng1:1*](http://data.perseus.org/citations/urn:cts:greekLit:tlg0016.tlg001.perseus-eng1:1)*. Accessed on 1/2/2020*. (Harvard University Press, 1920).

62 Blegen, C. W., Caskey, J. L., Rawson, M. & Sperling, J. *Troy: Excavations Conducted By the University of Cincinnati 1932-1938. Vol 1-4 [published between 1950-1958]*. (Princeton University Press for the University of Cincinnati, 1950).

63 Papamarinopoulos, S. *et al.* The exact date of the deaths of Patroclus & Hector. *Mediterranean Archaeological Archaeometry* **14**, 93-102 (2014).

64 Papamarinopoulos, S., Preka-Papadema, P., Gazeas, K., Nastos, P. & Kiriakopoulos, K. G. Extreme physical phenomena during the Trojan War. *Mediterranean Archaeological Archaeometry* **16**, 135-155 (2016).

65 Papamarinopoulos, S. *et al.* The anatomy of a complex astronomical phenomenon described in the Odyssey. *Mediterranean Archaeological Archaeometry* **13**, 68-81 (2013).

66 Papamarinopoulos, S. P. in *Science and Technology in Homeric Epics* Vol. History of Mechanism and Machine Science, vol 6 (ed S. A. Paipetis) 341-356 (Springer Science, 2008).

67 Roisman, J. in *A companion to Greek warfare* (eds W. Heckel, F.S. Naiden, E.E. Garvin, & J. Vanderspoel) 147-157 (John Wiley & Sons, Inc., 2021).

68 Finné, M., Holmgren, K., Sundqvist, H. S., Weiberg, E. & Lindblom, M. Climate in the eastern Mediterranean, and adjacent regions, during the past 6000 years - A review. *Journal of Archaeological Science* **38**, 3153-3173 (2011).

69 Casford, J. S. L. *et al.* Circulation changes and nutrient concentrations in the late quaternary Aegean sea: a non-steady state concept for sapropel formation. *Paleoceanography* **17**, 024-040 (2002).

70 Casford, J. S. L. *et al.* A dynamic concept for Eastern Mediterranean circulation and oxygenation during sapropel formation. *Palaeogeography, Palaeoclimatology, Palaeoecology* **190**, 103-119 (2003).

71 Rohling, E. J., Hayes, A., Mayewski, P. A. & Kucera, M. in *Forces of transformation: The end of the Bronze Age in the Mediterranean. Proceedings of an international symposium held at St. John's College, University of Oxford. 25-26th March 2006. Themes from the Ancient Near East BANEA Publication Series, Vol. 1* (eds C. Bachhuber & R. G. Roberts) (Oxbow Books, 2009).

72 United States National Oceanic and Atmospheric Administration. *Global Surface Summary of the Day. Available at:* [*https://data.nodc.noaa.gov/cgi-bin/iso?id=gov.noaa.ncdc:C00516*](https://data.nodc.noaa.gov/cgi-bin/iso?id=gov.noaa.ncdc:C00516).

73 United States National Oceanic and Atmospheric Administration. *Sunrise/Sunset and Solar Position Calculators. Earth System Research Laboratory, Global Monitoring Division. Available at:* [*https://www.esrl.noaa.gov/gmd/grad/solcalc*](https://www.esrl.noaa.gov/gmd/grad/solcalc).

74 Pritchett, W. K. *The Greek State at War, Part IV*. (University of California Press, 1985).

75 Holoka, J. P. Marathon and the myth of the same-day march. *Greek, Roman and Byzantine Studies* **38**, 329-353 (1997).

76 Kyriazis, N. & Paparrigopoulos, X. War and democracy in ancient Greece. *Eur J Law Econ* **38**, 163-183 (2014).

77 Crouwel, J. H. & Morel, J. *Chariots and other means of land transport in Bronze Age Greece*. (Allard Pierson Museum, 1981).

78 Deyaert, J., Harms, T., Weenas, D., Gershuny, J. & Glorieux, I. Attaching metabolic expenditures to standard occupational classification systems: perspectives from time-use research. *BMC Public Health* **17**, 620 (2017). <https://doi.org:10.1186/s12889-017-4546-7>

79 Ainsworth, B. E. *et al.* 2011 Compendium of Physical Activities: a second update of codes and MET values. *Med Sci Sports Exerc* **43**, 1575-1581 (2011). <https://doi.org:10.1249/MSS.0b013e31821ece12>

80 Poulianiti, K. P., Havenith, G. & Flouris, A. D. Metabolic energy cost of workers in agriculture, construction, manufacturing, tourism, and transportation industries. *Ind Health* **57**, 283-305 (2019). <https://doi.org:10.2486/indhealth.2018-0075>

81 Ober, J. in *Hoplites: The classical Greek battle experience* (ed V. D. Hanson) Ch. 7, 176-198 (Routledge, 1990).

82 Lattimore, R. *The Iliad of Homer. Rhapsody 9: 209-217*. (Chicago, IL, USA, 1961).

83 Dalby, A. *Food in the Ancient World from A to Z*. (Routledge, 2003).

84 Minchin, E. Food fiction & food fact in Homer's Iliad. *Petis Propos Culinaires* **25**, 42-49 (1987).

85 Sherratt, S. Feasting in Homeric epic. *Hesperia* **73**, 301-337 (2004).

86 Pritchett, W. K. *The Greek State at War, Part I*. (University of California Press, 1985).

87 Petroutsa, E. I. & Manolis, S. K. Reconstructing Late Bronze Age diet in mainland Greece using stable isotope analysis. *Journal of Archaeological Science* **37**, 614-620 (2010).

88 Halstead, P. & Isaakidou, V. in *Food, Cuisine and Society in Prehistoric Greece, vol. 5* (eds P. Halstead & J. C. Barrett) 136-154 (Oxbow Books, 2004).

89 Blegen, C. *Troy and the Trojans*. (Praeger, 1963).

90 Angel, J. L. in *Troy and the Trojan War: A Symposium Held at Bryn Mawr College, October 1984* (ed M. J. Mellink) (Bryn Mawr Commentaries, 1986).

91 Paul, A. A. & Southgate, D. A. T. *The composition of foods*. (Her Majesty's Stationery Office, 1979).

92 House of Commons Science and Technology Committee. Alcohol guidelines. Eleventh Report of Session 2010-12. (House of Commons, UK Parliament, The Stationery Office Limited, London, 2012).

93 Hatch, J. W. & Geidel, R. A. Tracing status and diet in prehistoric Tennessee. *Archaeology* **36**, 56-59 (1983).

94 Angel, J. L. Paleodemography and evolution. *American Journal Physical Anthropology* **31**, 343-353 (1969).

95 Huber, N. M. in *The skeletal biology of earlier human populations* (ed D. R. Brothwell) 73-75 (Pergamon Press Ltd., 1968).

96 Angel, J. L. Skeletal material from Attica. *Hesperia* **14**, 279-363 (1945).

97 Castleden, R. in *Mycenaeans* (ed R. Castleden) Ch. 3, 66-103 (Routledge, 2005).

98 Albracht, F. *Kampf und Kampfschilderung bei Homer. Ein Beitrag zu den Kriegsaltertümern. Beilage zum Jahresbericht der Königl. Landesschule Pforta 1886. Naumburg an der Saale 1886. pp. 27-40. Translated in 2005 by Jones P., Willcock M., and Wright G. Battle and Battle Description in the Iliad: A Contribution to the History of War. London.*, (1886).

99 Lang, A. *The World of Homer*. 54-59 (Longmans, Green, and Co., 1910).

100 Kirk, G. S. *War and the Warrior in the Homeric Poems (Problemes de la guerre en Greece ancienne)*. 111 (Mouton & Co., 1968).

101 van Wees, H. J. in *Homer: critical assessments. Vol 2.* (ed I. J. F. de Jong) 221-238 (Routledge, 2004).

102 Gibala, M. Molecular responses to high-intensity interval exercise. *Appl Physiol Nutr Metab* **34**, 428-432 (2009). <https://doi.org:10.1139/H09-046>

103 Gaebel, R. E. *Cavalry operations in the ancient Greek world*. 138 (University of Oklahoma Press, 2002).

104 Demakopoulou, K., Mangou, E., Jones, R. E. & Photos-Jones, E. Mycenaean black inlaid metalware in the National Archaeological Museum, Athens: a technical examination. *Annual British School at Athens* **90**, 137-153 (1995).

105 Photos, E., Jones, R. E. & Papadopoulos, T. The black inlay decoration on a Mycenaean bronze dagger. *Archaeometry* **36**, 267-275 (1994).

106 Taratori, P., Moschona-Katsarou, A. & Karydas, A. in *Proceedings of the 5th Symposium of the Hellenic Society of Archaeometry* (eds N. Zacharias *et al.*) 857-866 (Hellenic Society for Archaeometry, 2008).

107 Dakoronia, P., Deger-Jalkotzy, S. & Fabrizii-Reuer, S. in *DORON: Timetikos tomos yia ton kathegete Spyro Iakovide* (ed D. Danielidou) 211-229 (Athens Academy of Science, 2009).

108 Borchhardt, J. *Homerische Helme. Helmformen der Ägäis in ihren Beziehungen zu orientalischenund europäischen Helmen in der Bronze- und frühen Eisenzeit*. (Von Zabern, 1972).

109 Papapostolou, I. A. *Το Ιερό του Θέρμου στην Αιτολία*. 193, fig. 118β (Archaeological Society of Athens, 2014).

110 Wardle, D. E. H. in *Problems in Greek Prehistory* (eds E.B. French & K.A. Wardle) 469-476 (Bristol Classical Press, 1988).

111 Wardle, K. A. & Wardle, D. *Cities of legend: the Mycenaean world (Classical World Series)*. (Bristol Classical Press, 1998).

112 Molloy, B. Men of Bronze: Experimental approaches to the first body armour in the Aegean and Europe. *Talanta, Proceedings of the Dutch Archaeological and Historical Society* **44**, 273-294 (2012).

113 Sandars, N. K. Later Aegean bronze swords. *American Journal of Archaeology* **67**, 117-153 (1963).

114 Dietz, S., Papadopoulos, T. J. & Kontorli-Papadopoulou, L.

115 Ramanathan, N. L. A New Weighting System for Mean Surface Temperature of the Human Body. *J Appl Physiol* **19**, 531-533 (1964). <https://doi.org:10.1152/jappl.1964.19.3.531>

116 ASHRAE. Standard 55-2004—thermal environmental conditions for human occupancy. *ASHRAE Inc., Atlanta, GA* (2004).

117 Borg, G. Psychophysical scaling with applications in physical work and the perception of exertion. *Scand J Work Environ Health* **16**, 55-58 (1990).

118 American College of Sports, M. *et al.* American College of Sports Medicine position stand. Exercise and fluid replacement. *Med Sci Sports Exerc* **39**, 377-390 (2007). <https://doi.org:10.1249/mss.0b013e31802ca597>

119 Ramsthaler, F. *et al.* Hammer blows to the head. *Forensic Sci Int* **301**, 358-370 (2019). <https://doi.org:10.1016/j.forsciint.2019.05.045>

120 Riley, L. K. & Rupert, J. Evaluation of patients with leukocytosis. *Am Fam Physician* **92**, 1004-1011 (2015).

121 Tanabe, S.-i., Kobayashi, K., Nakano, J., Ozeki, Y. & Konishi, M. Evaluation of thermal comfort using combined multi-node thermoregulation (65MN) and radiation models and computational fluid dynamics (CFD). *Energy and Buildings* **34**, 637-646 (2002).

122 Voelker, C. *et al.* in *Eleventh International IBPSA Conference* 1360-1366 (Scotland, 2009).

123 Park, S. & Tuller, S. E. Human body area factors for radiation exchange analysis: standing and walking postures. *Int J Biometeorol* **55**, 695-709 (2011). <https://doi.org:10.1007/s00484-010-0385-2>

124 Fiala, D. *Dynamic Simulation of Human Heat Transfer and Thermal Comfort*, (1998).

125 Vale, J. P. & Mayor, T. S. Late Bronze Age Warrior Model. figshare. Software. <https://doi.org/10.6084/m9.figshare.12090831.v1>. (2020).

126 Gavin, T. P. *et al.* Clothing fabric does not affect thermoregulation during exercise in moderate heat. *Medicine & Science in Sports & Exercise* **33**, 2124-2130 (2001). <https://doi.org:10.1097/00005768-200112000-00023>

127 Zhao, Y., Yi, W., Chan, A. P. C. & Chan, D. W. M. Comparison of heat strain recovery in different anti-heat stress clothing ensembles after work to exhaustion. *J Therm Biol* **69**, 311-318 (2017). <https://doi.org:10.1016/j.jtherbio.2017.09.004>

128 Bourlai, T., Pryor, R. R., Suyama, J., Reis, S. E. & Hostler, D. Use of thermal imagery for estimation of core body temperature during precooling, exertion, and recovery in wildland firefighter protective clothing. *Prehosp Emerg Care* **16**, 390-399 (2012). <https://doi.org:10.3109/10903127.2012.670689>

129 A. S. M. International Handbook Committee. *ASM Speciality Handbook: Copper and Copper Alloys*. (ASM International, 2001).

130 *Engineering ToolBox:* [*https://www.engineeringtoolbox.com*](https://www.engineeringtoolbox.com), 2001).

131 Annino, A., Grasso, F., Musumeci, F. & Triglia, A. Spectral absorptivity of rough copper and brass surfaces. *Applied Physics A Solids and Surfaces* **35**, 115-118 (1984). <https://doi.org:10.1007/bf00620640>

132 Jakubinek, M. B., Samarasekera, C. J. & White, M. A. Elephant ivory: A low thermal conductivity, high strength nanocomposite. *Journal of Materials Research* **21**, 287-292 (2006). <https://doi.org:10.1557/jmr.2006.0029>

133 Feldmann, A. & Zysset, P. Experimental determination of the emissivity of bone. *Med Eng Phys* **38**, 1136-1138 (2016). <https://doi.org:10.1016/j.medengphy.2016.06.019>

134 Thellier, F., Monchoux, F., Bonnis-Sassi, M. & Lartigue, B. Modeling additional solar constraints on a human being inside a room. *Solar Energy* **82**, 290-301 (2008). <https://doi.org:10.1016/j.solener.2007.10.004>

135 Watanabe, S., Horikoshi, T., Ishii, J. & Tomita, A. The measurement of the solar absorptance of the clothed human body – The case of Japanese, college-aged male subjects. *Building and Environment* **59**, 492-500 (2013). <https://doi.org:10.1016/j.buildenv.2012.09.018>

136 McCullough, E. A. & Kenney, W. L. Thermal insulation and evaporative resistance of football uniforms. *Med Sci Sports Exerc* **35**, 832-837 (2003). <https://doi.org:10.1249/01.MSS.0000064998.48130.22>

137 Wang, F., Gao, C., Kuklane, K. & Holmer, I. Determination of clothing evaporative resistance on a sweating thermal manikin in an isothermal condition: heat loss method or mass loss method? *Ann Occup Hyg* **55**, 775-783 (2011). <https://doi.org:10.1093/annhyg/mer034>

138 Oh, W. & Kato, S. The effect of airspeed and wind direction on human's thermal conditions and air distribution around the body. *Building and Environment* **141**, 103-116 (2018). <https://doi.org:10.1016/j.buildenv.2018.05.052>

139 Nishi, Y. & Gagge, A. P. Direct evaluation of convective heat transfer coefficient by naphthalene sublimation. *J Appl Physiol* **29**, 830-838 (1970). <https://doi.org:10.1152/jappl.1970.29.6.830>

140 de Dear, R. J., Arens, E., Hui, Z. & Oguro, M. Convective and radiative heat transfer coefficients for individual human body segments. *Int J Biometeorol* **40**, 141-156 (1997). <https://doi.org:10.1007/s004840050035>

141 ASHRAE Inc. *2007 ASHRAE Handbook - Heating, Ventilating and Air-Conditioning Applications*. (2007).

142 Pandey, D. Effects of atmospheric emissivity on clear sky temperatures. *Atmospheric Environment* **29**, 2201-2204 (1995). <https://doi.org:10.1016/1352-2310(94)00243-e>

143 Gliah, O., Kruczek, B., Etemad, S. G. & Thibault, J. The effective sky temperature: an enigmatic concept. *Heat and Mass Transfer* **47**, 1171-1180 (2011). <https://doi.org:10.1007/s00231-011-0780-1>

144 Horswill, C. A. Applied physiology of amateur wrestling. *Sports Med* **14**, 114-143 (1992). <https://doi.org:10.2165/00007256-199214020-00004>

145 ASHRAE Inc. in *ASHRAE Handbook - Fundamentals* (ed Robert A. Parsons) 8.12 (1997).

146 Finné, M., Holmgren, K., Sundqvist, H. S., Weiberg, E. & Lindblom, M. Climate in the eastern Mediterranean, and adjacent regions, during the past 6000 years – A review. *Journal of Archaeological Science* **38**, 3153-3173 (2011). <https://doi.org:10.1016/j.jas.2011.05.007>

147 Taylor, N. A., Tipton, M. J. & Kenny, G. P. Considerations for the measurement of core, skin and mean body temperatures. *J Therm Biol* **46**, 72-101 (2014). <https://doi.org:10.1016/j.jtherbio.2014.10.006>
