# Supplementary material for: Analysis of Greek prehistoric combat in full body armour based on physiological principles: A series of studies using thematic analysis, human experiments, and numerical simulations
Source: PLoS One. 2024 May 22;19(5):e0301494. doi: 10.1371/journal.pone.0301494 (PMC11111059; doi:10.1371/journal.pone.0301494)
Supplement: Attachment — Submitted filename: Response to Reviewers.docx [file pone.0301494.s002.docx]

Ref.: Ms. PONE-D-23-17949

Dear Editor and Reviewers,

We wish to thank you for reviewing our manuscript and for your constructive comments. We understand that the short comings identified during the review process were important and we appreciate the fact that you valued the interest of our paper and have given us a chance to revise it. We made appropriate changes in the paper based on the comments that we received, and we believe that the revisions have markedly improved the paper’s quality. Also, we reviewed both the main text and the online supplement and made corrections to improve language and clarity.

The appropriate responses to each one of the points that you raised appear below. Where possible, red font is used to indicate revised parts of the text. As instructed, we have uploaded a marked-up copy of our manuscript that highlights changes made to the original version as a separate file labeled 'Revised Manuscript with Track Changes'. Also, we have uploaded an unmarked version of our revised paper without tracked changes as a separate file labeled 'Manuscript'. We wish to note that the line numbers appearing in our responses to your comments are based on the copy of the revised manuscript that includes track changes.

We would like to apologise for not being able to submit our revision sooner. Thessaly, the region in Greece where most of the authors reside in, was hit by major floods in September and there were considerable damages to our Department buildings. Until mid December our Department buildings we were without electricity and internet, and we only managed to return to our offices in early 2024.

Regrettably, one of our esteemed co-authors, Diana Wardle, passed away during Christmas. Dr Wardle made significant contributions to this work, and in recognition of her invaluable input and as a tribute to her memory, we, the remaining authors, have collectively decided to change the order of authors in our manuscript. We propose to list Dr Wardle as the last author to honour her contributions and to memorialize her impact on this research. We understand that changes in authorship can be sensitive and require clear justification, especially during the revision process. Please be assured that this decision was made with the utmost respect for academic integrity and reflects our desire to appropriately acknowledge Diana's contributions to our work. The revised version of the manuscript reflects the updated author order. All co-authors have agreed to this change and we can provide written consent from each if required. We trust that you will understand the circumstances that have led to this decision and we appreciate your support in this matter. If there are any further requirements or procedures we need to follow to formalize this change, please let us know.

On behalf of all authors,

Andreas Flouris

**Academic Editor:**

Comment No. 1:

Apart from deleting the last sentence of your paper ("…and set the origins of Western Civilization…"), as noted by one of the reviewers, I further recommend to delete the sentence “Eratosthenes provided…13th century BC” (lines 220–224). Later narratives cannot be taken as reliable evidence for the dating of the Trojan War. The Trojan War cannot be taken as a historical event, at least in the form described in the Homeric epics. There is quite a long list of literature on the historicity of the Trojan War and the historical implications of early Greek poetry. There is also no way to know if it reflects a single event or a turmoil period that was triggered during an alleged migration from Greece to the eastern Aegean, a narrative that has been also put into question. Additionally, I recommend to avoid dating the event in the 13th (throughout the paper) or any other century and replace that with relative dates (e.g. in the later phases of the Late Bronze Age, etc.). The above mentioned flaws undermine the paper‘s value.

*Response to comment No. 1:*

Thank you for highlighting this important issue. Dating the Trojan War was outside the scope of our study, but setting a hypothesized time period and season in which the events described in the Iliad took place was essential for deriving information about the physical environment (temperature, day-night cycle, etc.). Following your comment, we removed all instances of mentioning the 13^th^ century BC, except for once in the main manuscript and once in the Online Supplement. In these instances, we made every effort to highlight that this hypothesis is only used to calibrate the environmental conditions of our experiments and should not be considered as part of the general discussion on the dating of the Trojan War. Also, we added an extensive discussion on this issue in the Limitations Section of the main text.

Main text Limitations Section (L: 607-613):

“Our hypothetical date for the Trojan War (1300-1200 BC) was needed to enable estimates of the environmental conditions (temperature, day-night cycle, etc.) but should not be considered as a contribution to the continuing discussions about the date of the War. Moreover, the Trojan War cannot be taken as a historical event, at least in the form described in the Homeric epics [24]. There is no way to know whether it reflects a single event or a period of turmoil that was triggered during an alleged migration from Greece to the eastern Aegean, a narrative that has been also put into question [19, 24, 55].”

Main text Methodology Section (L: 359-367):

“Dating the Trojan War was outside the scope of the present study, yet setting a hypothesized time period and season was needed to calibrate the environmental conditions for our experiments (temperature, day-night cycle, etc.). Our thematic analyses of the Iliad suggested that the events described took place in the summertime. Assigning a date for the Trojan War is a complex challenge (see Limitations section) which has been a subject of frequent discussion since antiquity. Ancient authors have placed the Trojan War within a long period between 1334 and 1129 BC [19-23], yet it is not clear whether later narratives are reliable evidence for dating the event [24, 25]. Considering this information, we hypothesised that the events described in the Iliad took place in June during the later phases of the Late Bronze Age.”

Online Supplement Section 2.1 (L: 325-343):

“Dating the Trojan War was outside the scope of the present study. However, determining the time period and the month in which the events described in the Iliad took place was essential to deriving information about the physical environment (season, temperature, day-night cycle, etc.). Our thematic analysis of the Iliad suggested that the events described took place in the summertime, as Homer provides many references to increased heat, especially during mid-day. Yet, providing a date for the Trojan War is a complex challenge which has been a subject of frequent discussion since antiquity.^58^ Eratosthenes provided an estimate of ca. 1184 BC, Douris of Samos proposed that the War took place in 1334 BC,^59,60^ while an estimate of ca. 1250 BC was provided by both Herodotus61 and Blegen.^62^ In total, ancient authors have placed the Trojan War within a long period between 1334 and 1129 BC.^58-62^ A recent series of studies analysing in detail some of Iliad’s descriptions of physical phenomena covered by “divine interventions” (considering that physics at that age was entangled within mythos and theology) suggested that the events described in the Iliad may have taken place in June, 1218 BC.^63-66^ However, this conclusion is predicated on the assumption that, despite the distance in time, the described phenomena are precise and accurate memories of events that occurred, rather than being introduced for dramatic effect. Overall, it is not clear whether later narratives are reliable evidence for the dating of the Trojan War.^67^ Taking all this information and approaches into account, we hypothesised that the events described in the Iliad took place in June during the later phases of the Late Bronze Age (between 1300 and 1200 BC).”

Comment No. 2:

It is not clear how information provided by Strabo can contribute to the reconstruction of the Trojan landscape during the Late Bronze Age period (lines 214, 238). Please explain or rephrase the sentence.

*Response to comment No. 2:*

We inserted additional information to improve clarity, as follows (L: 392-393):

“We used these assumptions – together with geographic indicators and distances provided by Strabo [16], lithosome distributions and…”

Comment No. 3:

It is advisable to avoid “accurate” in any description of replicas (lines 299-300).

*Response to comment No. 3:*

The term “accurate” has been removed from any description of the replicas throughout the main text and the Online Supplement.

Comment No. 4:

Please explain why you use methods of the Archaic period to reconstruct a Late Bronze Age sword (line 182). Additionally, there were no cooper but bronze weapons as one of the reviewers already noticed. This does not certainly affect the – indeed innovative – results of your study but you have to comment on that.

*Response to comment No. 4:*

We opted for copper because bronze was not readily available. This is now explained in the Online Supplement. We also made various corrections in the relevant parts of the main text and the Online Supplement to improve clarity. Please, see our detailed response to comment No. 16.:

Comment No. 5:

Please remove the photo from page 8. You can either include it in the figures cited in the text or delete it. My suggestion would be to include more photos from the experimental study in the text (as you do in the supporting information document).

*Response to comment No. 5:*

As recommended, we removed the photo from page 8 and inserted it in the main text as Figure 7. Also, we moved Figures s11 and s13 to the main text, as Figures 5 and 6.

Comment No. 6:

I finally suggest that you include more recent literature on early Aegean warfare (see, e.g., Heckel et al. A Companion to Greek Warfare, 2021, with further literature).

*Response to comment No. 6:*

Throughout the main text and the Online Supplement, we added citations for several chapters of the suggested book. We also cited additional recent literature on early Aegean, such as different chapters from Brill’s Companion to Bronze Age Warfare in the Aegean, published during 2023.

**Journal Requirements:**

Comment No. 7:

Please ensure that your manuscript meets PLOS ONE's style requirements, including those for file naming. The PLOS ONE style templates can be found at

https://journals.plos.org/plosone/s/file?id=wjVg/PLOSOne_formatting_sample_main_body.pdf and

https://journals.plos.org/plosone/s/file?id=ba62/PLOSOne_formatting_sample_title_authors_affiliations.pdf

*Response to comment No. 7:*

We have revised the file names and other properties of our paper to meet PLOS ONE’s style requirements.

Comment No. 8:

We note that Figures 1 and S9 in your submission contain copyrighted images. All PLOS content is published under the Creative Commons Attribution License (CC BY 4.0), which means that the manuscript, images, and Supporting Information files will be freely available online, and any third party is permitted to access, download, copy, distribute, and use these materials in any way, even commercially, with proper attribution. For more information, see our copyright guidelines: http://journals.plos.org/plosone/s/licenses-and-copyright.

We require you to either (1) present written permission from the copyright holder to publish these figures specifically under the CC BY 4.0 license, or (2) remove the figures from your submission:

a. You may seek permission from the original copyright holder of Figures 1 and S9 to publish the content specifically under the CC BY 4.0 license.

We recommend that you contact the original copyright holder with the Content Permission Form (http://journals.plos.org/plosone/s/file?id=7c09/content-permission-form.pdf) and the following text:

“I request permission for the open-access journal PLOS ONE to publish XXX under the Creative Commons Attribution License (CCAL) CC BY 4.0 (http://creativecommons.org/licenses/by/4.0/). Please be aware that this license allows unrestricted use and distribution, even commercially, by third parties. Please reply and provide explicit written permission to publish XXX under a CC BY license and complete the attached form.”

Please upload the completed Content Permission Form or other proof of granted permissions as an ""Other"" file with your submission.

In the figure caption of the copyrighted figure, please include the following text: “Reprinted from [ref] under a CC BY license, with permission from [name of publisher], original copyright [original copyright year].”

b. If you are unable to obtain permission from the original copyright holder to publish these figures under the CC BY 4.0 license or if the copyright holder’s requirements are incompatible with the CC BY 4.0 license, please either i) remove the figure or ii) supply a replacement figure that complies with the CC BY 4.0 license. Please check copyright information on all replacement figures and update the figure caption with source information. If applicable, please specify in the figure caption text when a figure is similar but not identical to the original image and is therefore for illustrative purposes only.

*Response to comment No. 8:*

Figure 1 has been re-drawn to remove any copyrighted material, such as vases. This image now contains no copyrighted material. Regarding Figure s9, we confirm that it does not contain copyrighted images. It is a photograph of the Dendra armour replica used for the purposes of this study, and was taken by the authors. This is now indicated in the figure legend.

Comment No. 9:

We note that Figures 2 and S7 in your submission contain map/satellite images which may be copyrighted. All PLOS content is published under the Creative Commons Attribution License (CC BY 4.0), which means that the manuscript, images, and Supporting Information files will be freely available online, and any third party is permitted to access, download, copy, distribute, and use these materials in any way, even commercially, with proper attribution. For these reasons, we cannot publish previously copyrighted maps or satellite images created using proprietary data, such as Google software (Google Maps, Street View, and Earth). For more information, see our copyright guidelines: http://journals.plos.org/plosone/s/licenses-and-copyright.

We require you to either (1) present written permission from the copyright holder to publish these figures specifically under the CC BY 4.0 license, or (2) remove the figures from your submission:

a. You may seek permission from the original copyright holder of Figures 2 and S7 to publish the content specifically under the CC BY 4.0 license.

We recommend that you contact the original copyright holder with the Content Permission Form (http://journals.plos.org/plosone/s/file?id=7c09/content-permission-form.pdf) and the following text:

“I request permission for the open-access journal PLOS ONE to publish XXX under the Creative Commons Attribution License (CCAL) CC BY 4.0 (http://creativecommons.org/licenses/by/4.0/). Please be aware that this license allows unrestricted use and distribution, even commercially, by third parties. Please reply and provide explicit written permission to publish XXX under a CC BY license and complete the attached form.”

Please upload the completed Content Permission Form or other proof of granted permissions as an ""Other"" file with your submission.

In the figure caption of the copyrighted figure, please include the following text: “Reprinted from [ref] under a CC BY license, with permission from [name of publisher], original copyright [original copyright year].”

b. If you are unable to obtain permission from the original copyright holder to publish these figures under the CC BY 4.0 license or if the copyright holder’s requirements are incompatible with the CC BY 4.0 license, please either i) remove the figure or ii) supply a replacement figure that complies with the CC BY 4.0 license. Please check copyright information on all replacement figures and update the figure caption with source information. If applicable, please specify in the figure caption text when a figure is similar but not identical to the original image and is therefore for illustrative purposes only.

The following resources for replacing copyrighted map figures may be helpful:

USGS National Map Viewer (public domain): http://viewer.nationalmap.gov/viewer/

The Gateway to Astronaut Photography of Earth (public domain): http://eol.jsc.nasa.gov/sseop/clickmap/

Maps at the CIA (public domain): https://www.cia.gov/library/publications/the-world-factbook/index.html and https://www.cia.gov/library/publications/cia-maps-publications/index.html

NASA Earth Observatory (public domain): http://earthobservatory.nasa.gov/

Landsat: http://landsat.visibleearth.nasa.gov/

USGS EROS (Earth Resources Observatory and Science (EROS) Center) (public domain): http://eros.usgs.gov/#

Natural Earth (public domain): http://www.naturalearthdata.com/

*Response to comment No. 9:*

The maps included in Figures 2 and s1 (now Figure s3) were created with Azgaar's Fantasy Map Generator, a free web application, under a CC BY license, with permission from Max Haniyeu, original copyright 2017-2021 (<https://github.com/Azgaar/Fantasy-Map-Generator/blob/master/LICENSE#start-of-content>). This information is now included in the legend of each figure, as well as in the Results section and the Data Availability Statement.

Main text Figure 2 legend (L: 346-351):

“Figure 2. Top: geomorphology of the area surrounding Troy in the later phases of the Late Bronze Age (labels indicate the locations of the two army encampments and the geographic features of the area). The map was created using Azgaar's Fantasy Map Generator, a free web application, under a CC BY license, with permission from Max Haniyeu, original copyright 2017-2021. Bottom: Volunteer marine soldiers in simulated combat wearing the Dendra armour replica during the empirical study (right) and an artistic photo shoot (left).”

Online Supplement Figure s3 legend (L: 436):

“Figure s3. Characteristics of the march between the Greek camp and Troy (panels A and B) and analysis of the battle tactics used in the four days of battle (out of the 10 days in total) described in the Iliad (panels C-F). The maps were created using Azgaar's Fantasy Map Generator, a free web application, under a CC BY license, with permission from Max Haniyeu, original copyright 2017-2021.”

Main text Results and Discussion Section (L: 340-341):

“…and the geographic indicators and distances provided by Strabo [17], we used Azgaar's Fantasy Map Generator, a free web application (under a CC BY license, with permission from Max Haniyeu, original copyright 2017-2021), to create our interpretation of the geomorphologies in the area surrounding Troy at the time of the events described in the Iliad (Figure 2).”

Main text Data Availability Statement (L: 781-783):

“The map was created using Azgaar's Fantasy Map Generator, a free web application, under a CC BY license, with permission from Max Haniyeu, original copyright 2017-2021.”

Finally, regarding Figure s7 (now Figure s1), the image has been re-drawn to remove any copyrighted material. This image now contains no copyrighted material.

Comment No. 10:

Please include captions for your Supporting Information files at the end of your manuscript, and update any in-text citations to match accordingly. Please see our Supporting Information guidelines for more information: http://journals.plos.org/plosone/s/supporting-information.

*Response to comment No. 10:*

We have included captions for our Supporting Information files at the end of the manuscript to meet PLOS ONE’s Supporting Information guidelines.

Comment No. 11:

We note that Figures 3, S4 and S17 includes an image of a participant in the study.

As per the PLOS ONE policy (http://journals.plos.org/plosone/s/submission-guidelines#loc-human-subjects-research) on papers that include identifying, or potentially identifying, information, the individual(s) or parent(s)/guardian(s) must be informed of the terms of the PLOS open-access (CC-BY) license and provide specific permission for publication of these details under the terms of this license. Please download the Consent Form for Publication in a PLOS Journal (http://journals.plos.org/plosone/s/file?id=8ce6/plos-consent-form-english.pdf). The signed consent form should not be submitted with the manuscript, but should be securely filed in the individual's case notes. Please amend the methods section and ethics statement of the manuscript to explicitly state that the patient/participant has provided consent for publication: “The individual in this manuscript has given written informed consent (as outlined in PLOS consent form) to publish these case details”.

If you are unable to obtain consent from the subject of the photograph, you will need to remove the figure and any other textual identifying information or case descriptions for this individual.

*Response to comment No. 11:*

We confirm that the individuals appearing in Figures 2, s4, s16, and s17 in this manuscript have signed the Consent Form for Publication in a PLOS Journal and these are securely filed in their individual case notes. The manuscript has been adapted accordingly:

Main text Methods Section (L: 219-221):

“The individuals appearing in Figures 2, 7, s6, s16, and s17 in this manuscript have given written informed consent (as outlined in PLOS consent form) to publish these photographs.”

Online Supplement Section 4.2 (L: 686-687):

“The individuals appearing in Figures 2, 7, s6, s16, and s17 in this manuscript have given written informed consent (as outlined in PLOS consent form) to publish these photographs.”

Comment No. 12:

Please review your reference list to ensure that it is complete and correct. If you have cited papers that have been retracted, please include the rationale for doing so in the manuscript text, or remove these references and replace them with relevant current references. Any changes to the reference list should be mentioned in the rebuttal letter that accompanies your revised manuscript. If you need to cite a retracted article, indicate the article’s retracted status in the References list and also include a citation and full reference for the retraction notice.

*Response to comment No. 12:*

We confirm that, to the best of our knowledge, our reference list is complete and correct and does not include papers that have been retracted.

**Reviewer #1:**

Comment No. 13:

This is an interesting and important study. Previous interpretations of the Dendra Corselet have been speculative and devoid of any real analysis. Therefore the literature about the Dendra armour has tended to be full of "truisms". The main "truism" is that the Dendra armour is essentially ceremonial and could not have been used practically. The author of this article sets out to demonstrate this is not so. Using a series of practical scientific experiments he establishes that the Dendra armour is practical and could have worn in actual combat. This is the primary aim of the project, and is discussed at the beginning of the article. The methodology for the project is described as a series of practical experiments wherein an accurate replica of the armour is worn by a group of volunteers (fit young men from the Greek army), and they undertook physical actions appropriate to combat. They were then subjected to a series of the tests to establish their physiological response to the stresses of the actions. In the article the tests are described fully and the results presented (additional detail is also provided through links to online databases). Consequently the primary of aim of the project is proved: that the Dendra armour could be used practically in combat and did not impeded the warrior's mobility or combat effectiveness.

That said: the article as presented is essentially quantitative in its discussion. This is one of its strengths. It is written seemingly for a scientifically minded audience, with appropriate "hard" evidence. The author however, in part of the online material includes a supplementary discussion of the project which is primarily qualitative in form and style. It is therefore more accessible to those of us with less scientific minds. It manages to address the sorts of questions which might occur to archaeologists and scholars of ancient combat. It also elaborates more on the interpretative implications of the study. Therefore I strongly urge the author to consider incorporating some of the discussion from the qualitative version into the quantitative. Or he could design the qualitative version to stand as a Part 2. It will definitely have a wider accessibility. I am happy to recommend publication of this article.

*Response to comment No. 13:*

Thank you for your supportive comments. We would be happy to re-design the way the information is presented so that the qualitative and quantitative versions can stand as Parts 1 and 2. Given that we are unsure if the Academic Editor agrees with this approach, in this revision we have only addressed the remaining comments and suggestions. If the Academic Editor agrees with this approach, we would be happy to present a final version of the manuscript, to be submitted as one part of the work together with a separate submission which would stand as the other part.

**Reviewer #2:**

Comment No. 14:

I congratulate the authors on a most original, thorough, and thought-provoking piece of research, which adds significantly to our knowledge and understanding of Mycenaean combat tactics. The research conclusively demonstrates that the Dendra armour was a functional, and probably much used, piece of defensive kit. What's more, the article and supplementary materials are written in a fluent and sophisticated language that does not require any further editing. I warmly recommend publishing the manuscript once the authors have carried out the minor corrections recommended below.

*Response to comment No. 14:*

Thank you for your supportive comments. We have made all the revisions suggested by you, Reviewer 1, and the Academic Editor and we agree that the paper has been drastically improved.

Comment No. 15:

line 78ff. The authors give the impression that the Iliad provides a coherent account of fighting tactics from the late Mycenaean period. As I'm sure they are aware, the Iliad is palimpsest of contents created in different periods, as shown in particular by mismatching or otherwise problematic descriptions of weapons, armour and tactics (e.g., the well-known example of chariots being used in the Iliad as 'battlefield taxis'). They should discuss, in at least a paragraph, how they navigated the problem and resolved the many contradictions posed by the text.

*Response to comment No. 15:*

Thank you for raising this important point. We added the following text in the Methods section (L: 148-183), and we further included a discussion on this issue in the Limitations section (L: 598-606).

“Although Homer provides a detailed description of the weapons and combat moves in his epics [8], it is still unclear whether the Iliad provides a robust account of fighting tactics from the late Mycenaean period as it is a palimpsest of contents created in different periods. This results in, for example, problematic descriptions of armour and tactics in some cases. We navigated this problem and resolved contradictions by complementing our thematic analyses with an extensive review of the scholarly literature (Online Supplement, Sections 2.1-2.7). This ensured that the findings from the thematic analyses were in line with the best available evidence. For the literature review, we searched the Google Scholar and PubMed databases from their date of inception to January 2020 using search terms related to each of the above-mentioned seven warfare and battle tactics topics. We included all types of peer-reviewed papers, conference proceedings, and books. Manuscript titles and abstracts were subsequently screened for relevance. Thereafter, full texts (retrieved via the University of Thessaly library) were screened independently by three reviewers (ADF, SP, and PA). The results from the two thematic analyses of the Iliad combined with the results from the review of the literature are summarised in the Online Supplement (Section 2) and were used to create a Late Bronze Age combat simulation protocol (Section 3), while the data has been placed in an online data repository and are freely available [9].”

“First, the Iliad is undoubtedly the most appropriate text to use for developing a Late Bronze Age combat simulation protocol [8, 25]. However, it is not clear whether it provides a robust account of fighting tactics from the late Mycenaean period, as it is a palimpsest of contents created in different periods and can lead, for example, to misinterpretation or oversimplification of complex military practices. Therefore, we complemented our thematic analyses with an extensive review of the scholarly literature to ensure that our findings were in line with the best available evidence.”

Comment No. 16:

line 181. It's not clear why the replica sword was manufactured from pure copper considering that late Mycenaean swords were made of tin-bronze. This should be corrected or explained.

*Response to comment No. 16:*

We opted for copper because bronze was not readily available. This is now explained in the Online Supplement. We also made various corrections in the relevant parts of the main text and the Online Supplement to improve clarity, as follows:

Main text Methods Section (L: 283-299):

“For the purposes of this study, we created a replica Mycenaean cruciform sword (Sandars type Di) [12] of the type reported to have been found in the Dendra tomb, which had blunt edges and point for safety reasons using methods and materials of the archaic period (Online Supplement, Section 4.6 and Figure s10). The sword was 0.85 m long with a weight of 1.2 kg (copper blade, wooden handle, copper ornamental nails and pins), being slightly heavier than the original. For the combat moves requiring spear throw or strike, we used a 2.2 m replica wooden (ashwood) spear with a copper head blunt edges and point for safety reasons (0.46 kg), similar to those described in the Iliad. Also, a medium-sized stone (23 x 15 cm; 1.3 kg weight) was used in the “foot warrior vs chariot” encounters (Table s4) and a composite recurve bow [13] was used to shoot an arrow at close-range in the “chariot vs warrior on ship” encounter (Table s4).”

Online Supplement Section 4.6 (L: 891-898):

“For the purposes of this study, we created a replica Mycenaean cruciform sword (Sandars type Di)^113^ of the type reported to have been found in the Dendra tomb, which had blunt edges and point for safety reasons. Since bronze was not readily available, we used copper for the blade, chestnut wood for the handle and fish-based glue (Figure s10). The replica sword was 0.85 m long and weighed 1.2 kg (copper blade, wooden handle, copper ornamental nails and pins). Given that the specific gravity of 10% tin bronze is around 8.72g/cc and that of copper is 8.95g/cc, the original sword is slightly lighter than the replica used in the present study and, therefore, will have required less effort in Bronze Age combat contexts than in our experimental protocol.”

Comment No. 17:

line 383: I strongly recommend that the problematic closing "and set the origins of Western Civilization" is deleted. The article closes rather well, and in fact better, with "the beginning of the Age of Iron".

*Response to comment No. 17:*

As recommended, that part of the sentence was deleted.
